# Reductive Transamination of Pyridinium Salts to N-Aryl Piperidines

**DOI:** 10.1021/acs.joc.4c00493

**Published:** 2024-06-13

**Authors:** Zhenyu Chen, Geyang Song, Leiming Qi, Ramachandran Gunasekar, Christophe Aïssa, Craig Robertson, Alexander Steiner, Dong Xue, Jianliang Xiao

**Affiliations:** † Department of Chemistry, University of Liverpool, Liverpool L69 7ZD, U.K.; ‡ Key Laboratory of Applied Surface and Colloid Chemistry, Ministry of Education and School of Chemistry and Chemical Engineering, Shaanxi Normal University, Xi’an 710119, China

## Abstract

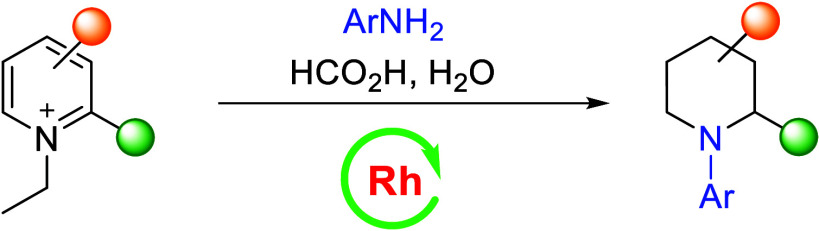

Saturated N-heterocycles are found in numerous bioactive natural products and are prevalent in pharmaceuticals and agrochemicals. While there are many methods for their synthesis, each has its limitations, such as scope and functional group tolerance. Herein, we describe a rhodium-catalyzed transfer hydrogenation of pyridinium salts to access N-(hetero)aryl piperidines. The reaction proceeds via a reductive transamination process, involving the initial formation of a dihydropyridine intermediate via reduction of the pyridinium ion with HCOOH, which is intercepted by water and then hydrolyzed. Subsequent reductive amination with an exogenous (hetero)aryl amine affords an N-(hetero)aryl piperidine. This reductive transamination method thus allows for access of N-(hetero)aryl piperidines from readily available pyridine derivatives, expanding the toolbox of dearomatization and skeletal editing.

## Introduction

**Figure 1:**
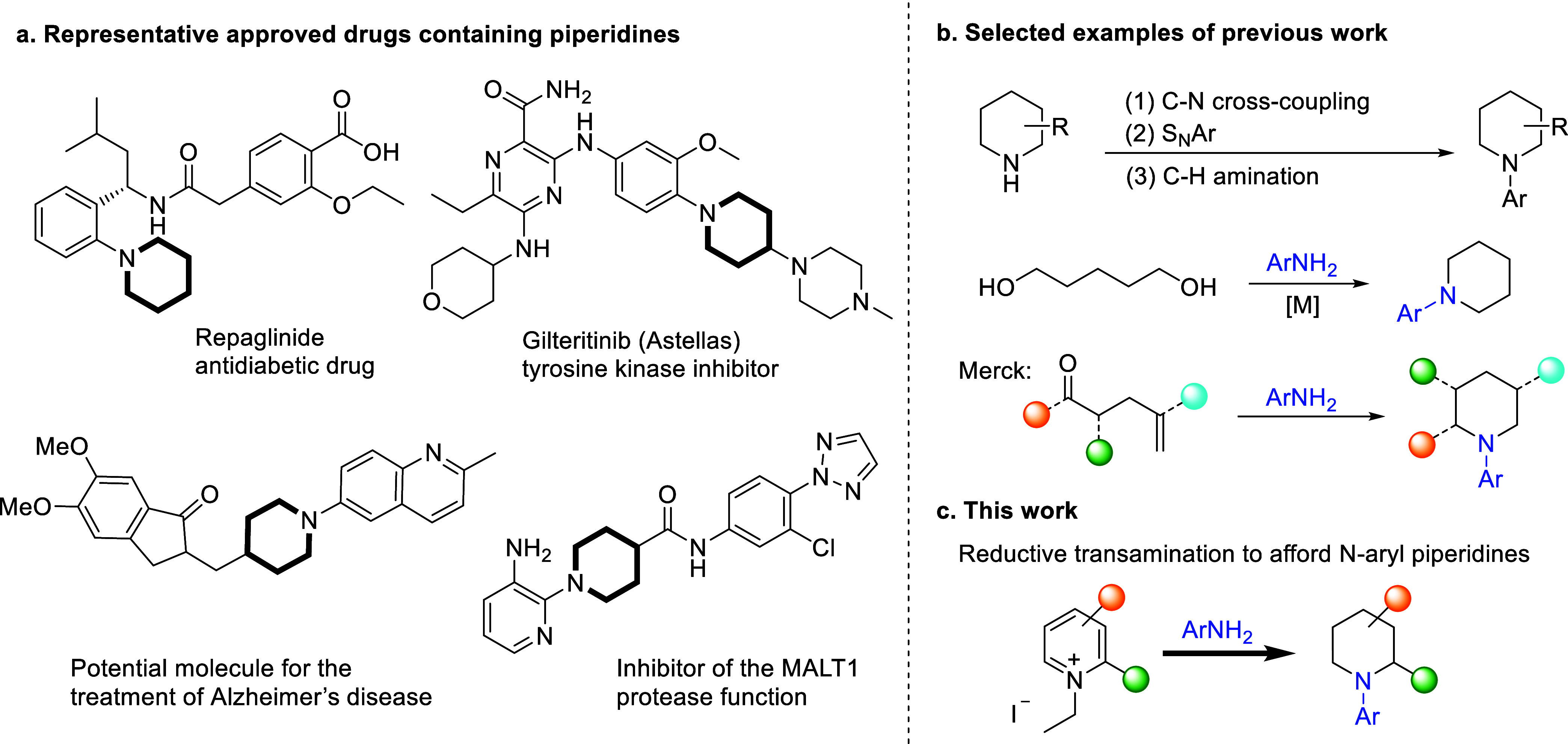
Bioactive N-(hetero)aryl piperidines and strategies for their synthesis. (a) Examples of FDA-approved drugs containing N-(hetero)aryl piperidines. (b) Examples of known synthetic approaches to N-aryl piperidines. (c) This work: rhodium-catalyzed reductive transamination leading to N-(hetero)aryl piperidines.

Saturated nitrogen heterocycles, like piperidines, are significant structural motifs in natural products and pharmaceuticals.^1^ In fact, around 60% of the US Food and Drug Administration (FDA)-approved drugs contain at least one N-heterocyclic structural unit, of which piperidines are the most frequently seen ring systems.^1c^ The N-arylated piperidines are also attractive structures due to their prevalence as scaffolds in approved and potential drug molecules (Figure 1a).^2^ The most versatile methods to access such compounds are the palladium-catalyzed Buchwald–Hartwig^3^ and copper-mediated Ullmann–Goldberg C–N coupling reactions.^4^ Although well-developed and widely used, these amination methods may encounter some difficulties with less-reactive electron-rich aryl chlorides,^4e,5^ heteroaryl substrates,^6^ and the use of strong bases,^7^ which could render the reaction incompatible with functional groups (Figure 1b). Base-promoted nucleophilic aromatic substitution (S_N_Ar) reactions provide another popular approach to N-aryl piperidines.^8^ However, the approach is hampered by the necessity for highly electron-deficient aryl halides. More recently, examples of aromatic C–H amination with aliphatic amines have been reported.^1f,9^ Alternatively, N-aryl piperidines can be accessed via the reaction of aryl amines with 1,5-difunctionalized compounds via S_N_2 substitution, reductive amination, or “borrowing hydrogen” strategies (Figure 1b).^6b,10^ A notable recent example is from Merck researchers, who devised a novel reductive amination/aza-Michael cyclization strategy that enables the synthesis of challenging N-(hetero)aryl piperidines from 2-methylene-5-oxohexanoates (Figure 1b).^6b^ However, 1,5-difunctionalized substrates with additional functionalities in the chain are limited in commercial availability or challenging to prepare in general.^11^

The importance of N-aryl piperidines and the problems in accessing them prompted us to search for an alternative method for their preparation. We recently reported a new catalytic approach to produce chiral piperidines via asymmetric reductive transamination (ART) of pyridinium salts with a chiral aliphatic amine.^12^ Building on this work, we herein present a reductive transamination synthesis of functionalized N-aryl piperidines from easily available pyridinium salts, including particularly those that may be difficult to access by conventional methods (Figure 1c).

## Results and Discussion

**Scheme 1:**
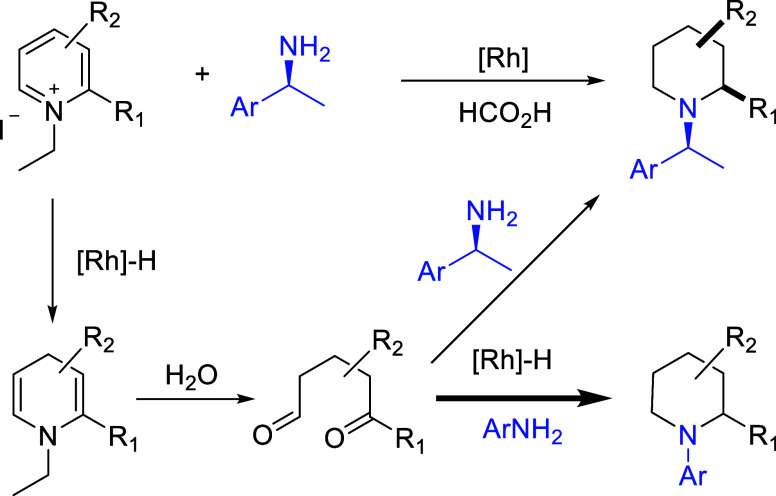
Simplified Pathway of the ART and Proposed Arylation Reactions Catalyzed by the Precatalyst [Cp*RhCl_2_]_2_

The reported ART reaction converts a pyridinium salt into a chiral piperidine (Scheme 1). Under reducing conditions in the presence of water, a chiral amine introduced undergoes transamination to replace the original nitrogen moiety in the pyridinium ion, thereby affording a piperidine with high diastereoselectivity. The reaction proceeds via a pathway that involves two key intermediates. As shown in Scheme 1, a Rh-catalyzed transfer hydrogenation with formic acid first produces a dihydropyridine intermediate, which is hydrolyzed in situ by water, affording a dicarbonyl intermediate. Subsequent reductive amination with an exogenous chiral amine under Rh catalysis leads to the cyclized product, an enantiomerically enriched piperidine.^12^

Given the prominence of N-(hetero)aryl heterocycles in drug development, we thought that it should be beneficial to replace the aliphatic amine used in the ART with an aryl, albeit less nucleophilic, variant, ArNH_2_, thus affording an N-aryl piperidine (Scheme 1). In a previous study,^12^ we obtained chiral piperidines using a mixture of an aliphatic amine (10 equiv) and formic acid (24 equiv) as the amine and hydrogen source, respectively, and a catalyst generated *in situ* from [Cp*RhCl_2_]_2_ in CH_2_Cl_2_/H_2_O. We commenced our exploration of transamination of pyridinium salts with *p*-anisidine, starting with this condition (Table 1). Delightfully, after optimization, the target N-aryl piperidine **1** was obtained with a remarkable yield of 86% using 10 equiv of the aryl amine. This was achieved by performing the reaction in a mixture solvent of MeOH/H_2_O (entry 1), and we found that the choice of solvent is critical to the success of the reaction. Thus, a much lower yield was noted when the reaction was performed in CH_2_Cl_2_/H_2_O (entry 4), the solvent of choice in the original ART reaction, while significantly increased yields were obtained in polar, protic solvents, with MeOH/H_2_O (15:1 v/v) being the most effective (see the Supporting Information for more details). We also found that triethylamine (NEt_3_) could be used to balance the basicity of the reaction system and, thus, replace part of the aryl amine without affecting the product yield (entry 2). Under such conditions, the reaction is feasible even with only 1 equiv of *p*-anisidine, albeit with a significantly decreased yield of **1** (entry 3). This should particularly benefit reactions where expensive aryl amines are used. The reaction became sluggish at low temperatures, with the yield decreasing to 30% when run at ambient temperature. However, little change was observed when the temperature was increased to 60 °C. All of the reagents, including the Rh(III) catalyst, are stable to air and moisture in solution, leading to reproducible results under either an air or N_2_ atmosphere (entries 5 vs 1). Changing the counteranion to bromide and ethyl to a benzyl substituent was found to decrease the yield (entry 6). Notably, switching to the noncoordinating anions, PF_6_^–^ and BF_4_^–^, led to no reaction (entries 7–8). This is in line with what we found in the previous studies, which showed that the iodide anion plays an important role in promoting the transfer hydrogenation.^13^

**Table 1:**
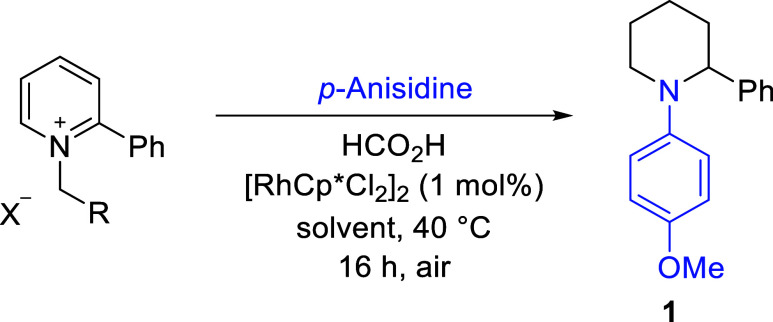
Optimization of Reaction Conditions^a^

entry	X, R	conditions	yield (%)^b^
1	I, Me	MeOH/H_2_O	86
2	I, Me	MeOH/H_2_O *p*-anisidine (5 equiv) + NEt_3_ (5 equiv)	84^c^
3	I, Me	MeOH/H_2_O *p*-anisidine (1 equiv) + NEt_3_ (9 equiv)	55
4	I, Me	CH_2_Cl_2_/H_2_O	11
5	I, Me	MeOH/H_2_O, N_2_	85
6	Br, Ph	MeOH/H_2_O	51
7	PF_6_, Me	MeOH/H_2_O	NA^d^
8	BF_4_, Me	MeOH/H_2_O	NA^d^

a Reaction conditions: 0.5 mmol of pyridinium salt, 10 equiv of *p*-anisidine, 24 equiv of HCO_2_H, CH_2_Cl_2_/H_2_O = 15:1 (4.0 mL), 1 mol % [Cp*RhCl_2_]_2_, 40 °C, 16 h, in air, unless otherwise indicated.

b Isolated yields using flash column chromatography.

c Optimized (standard) condition.

d No reaction observed.

With the optimized reaction conditions in hand, we first explored the scope of pyridinium salts in the reaction with *p*-anisidine (Table 2). The substrates were readily prepared from bromopyridines via the Suzuki–Miyaura coupling followed by quaternization, which activates pyridines toward nucleophilic attack by a metal hydride.^14^ The reductive transamination worked well for a wide range of 2-aryl and 2-alkyl substituted pyridinium salts, affording the N-arylated piperidines in good yields in general. The N-arylated piperidine **1** was isolated with a yield of 84% under standard conditions. A slightly lower yield, 75%, was recorded when the same reaction was performed on a gram scale (1.09 g) for 24 h. Substrates bearing electron-withdrawing groups appear to afford slightly lower yields compared with those bearing electron-donating ones, e.g., **5** vs **9**. The steric effect is more pronounced, as seen in **14** and **15**, where the sterically more demanding pyridinium precursor to **15** furnished a much lower yield. Furthermore, when the 2-aryl group is 2,6-dimethoxyphenyl, no reaction was observed, most likely due to a high energy barrier in the ring closure step (vide infra). In the reaction of a nitrile-bearing pyridinium, the piperidine product **7** reacted further with *p*-anisidine, leading to the formation of a secondary amine byproduct (see the Supporting Information). To avoid the nucleophilic addition of excessive amines to the nitrile group, the amount of *p*-anisidine was reduced to 1.2 equiv. This improved the yield of **7** from 35 to 58%, while again indicating the reaction to be feasible even with near-stoichiometric aryl amines. Notably, potentially reactive functional groups were well-tolerated, such as halides (**2**, **3**, **4**, **17**, **33**, **37**), nitro (**6**), ketone (**13**), and ester (**25**), some of which might not survive common C–N coupling conditions. The preservation of the functionalities in these piperidine products opens the possibility of further functionalization.

**Table 2:**
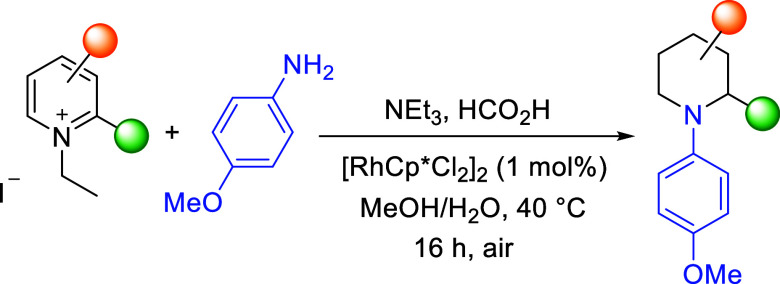

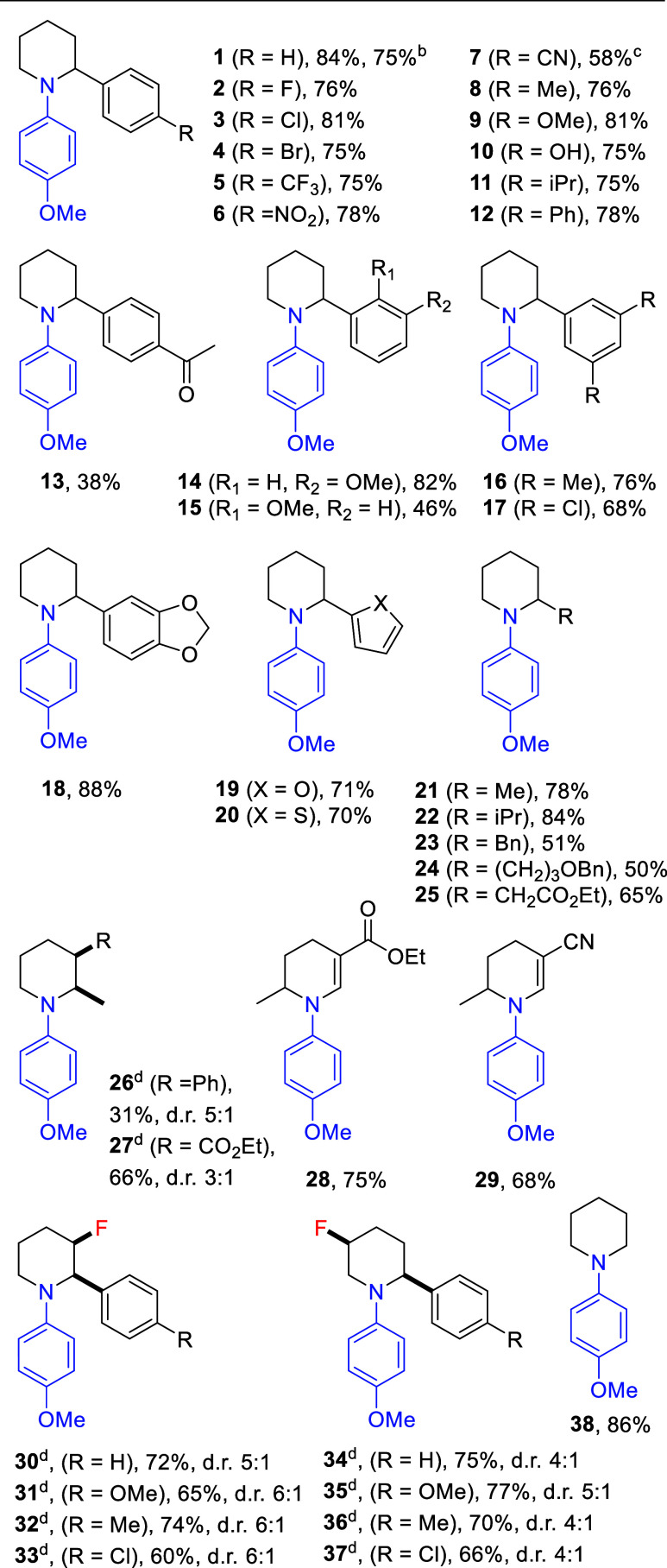
Reductive Transamination of Pyridinium Salts with *p*-Anisidine^a^

a Reaction conditions: 0.5 mmol of pyridinium salt, 5 equiv of *p*-anisidine, 5 equiv of NEt_3_, 24 equiv of HCO_2_H, MeOH/H_2_O = 15:1 (4.0 mL), 1 mol % [Cp*RhCl_2_]_2_, 40 °C, 16 h, in air. Isolated yields are reported.

b Reaction was performed on a 3.5 mmol (1.09 g) scale for 24 h.

c 1.2 equiv of *p*-anisidine was used.

d Yields of the isolated major *cis* diastereomers are reported. The d.r. (*cis*/*trans*) was determined by analysis of the ^1^H NMR spectra of crude mixtures.

The reductive transamination also worked for some disubstituted pyridinium salts. Thus, 2-methylpiperidines **26** with a 3-phenyl motif and **27** with 3-carboxylate were obtained in moderate to good yields, demonstrating the tolerance of the reaction to different substituents at the C3 position of the pyridinium ring. These compounds were isolated as single *cis* products; however, they were formed as a pair of *cis* and *trans* diastereomers, with the diastereomeric ratios (d.r.) being 5:1 for **26** and 3:1 for **27** according to the ^1^H NMR measurement of the crude product (vide infra). Surprisingly somehow, moving the methyl group from the C2 to C6 position resulted in the formation of the tetrahydropyridine **28**. This partial hydrogenated product is likely to be stabilized by the extensive conjugation involving the nitrogen lone pair, the C=C bond, the carboxylate group, and the aromatic ring.^15^ When the reaction time was prolonged to 48 h, the olefin moiety remained mostly intact, with only ca. 20% conversion to the fully hydrogenated piperidine. Similarly, the 2-cyano substituted product **29** could be obtained in a good yield. The reduction of the C=C bond in the precursors to **26** and **27** may be attributed to easier enamine and iminium isomerization; the final product results from the reduction of the latter.

**Figure 2:**
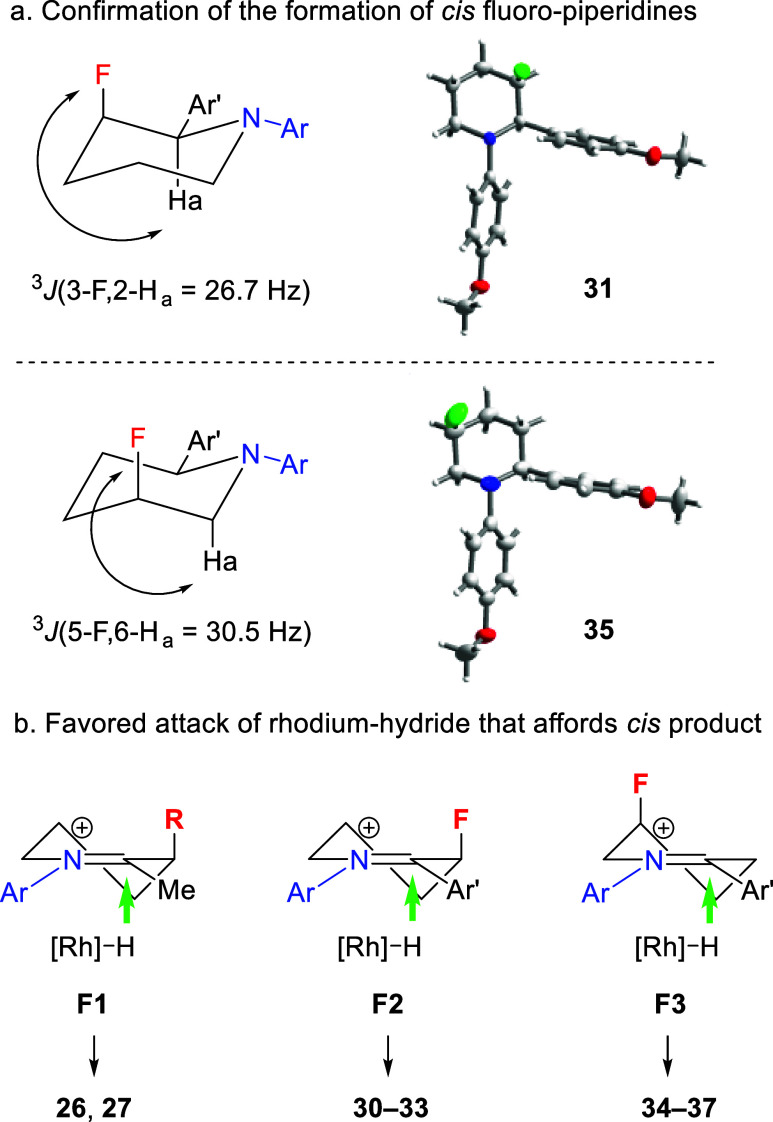
(a) Axial orientation of the fluorine substituent in the X-ray structures of **31** and **35**. (The structures are disordered; see the Supporting Information for details. Thermal ellipsoids are shown at the 50% probability level.) (b) Illustration of steric and stereoelectronic effects in directing the formation of the *cis* product (the cyclic iminium ion results from the reaction of a dicarbonyl intermediate with an aryl amine; see Scheme 2).

Fluorine-containing molecules exhibit unique properties in material science and pharmaceuticals.^16^ In particular, around 20% of all approved medicines contain fluorine atoms.^17^ However, the direct synthesis of fluoropiperidines via the hydrogenation of fluoropyridine precursors remains rare, largely due to the hydrodefluorination side reaction.^18^ The mild conditions of the reductive transamination reaction make one-step access to N-aryl fluorinated piperidines possible. As shown in Table 2, a range of 2-aryl-3-fluoropiperidines and the 5-fluoro analogues were obtained in good yields under the standard conditions (**30–37**). These compounds were isolated as single *cis* diastereomers; however, as in the cases of **26** and **27**, they were formed as a mixture of two diastereomers, with the *cis* isomer accounting for the major products. The X-ray structures of **31** and **35** were determined and are consistent with the NMR analysis, showing the aryl and fluorine to be *cis*, with the latter being axial (Figure 2a).

The formation of the *cis* isomers as the major products may be attributed to the reduced allylic strain and/or favored electrostatic interactions when the hydride adds to the C=N bond, as illustrated in Figure 2b. In the case of a phenyl or an ester group at position 3 of the pyridine, minimization of the allylic strain would favor the conformer **F1** to give the *cis* isomer of **26** preferentially. When a fluorine atom is present at position 3 or 5, Coulombic attraction favors conformers **F2** and **F3**, which leads to the dominant formation of the *cis* isomer of **30**–**33** and **34**–**37**, respectively.^16g^ Finally, it is noted that the reaction worked well for a nonsubstituted pyridinium substrate, affording **38**.

We next extended this approach to other aniline derivatives. As shown in Table 3, various amines, including those that are very electron-rich and hence rarely featured in C–N coupling reactions, can be brought into the reductive transamination reaction to afford the corresponding N-aryl piperidines. Notably, phenylamines bearing *para*-bromine and iodine substituents were tolerated in this catalytic system to give piperidines **40** and **41** accordingly, albeit in lower yields, possibly due to the lower nucleophilicity of the halogenated anilines. Such amines are prone to undergoing homocoupling in the conventional C–N coupling reactions.^3d^ Delightfully, the highly electron-donating 4-amino (σ = 0.57) and 4-hydroxy (σ = 0.38) substituted anilines went through the reaction smoothly to give amine products **43** and **44** in good yields, so did the electron-rich 2,4-dimethoxyaniline that gave rise to **51**. 2-Naphthylamine also worked well (**54**, 77%); however, the sterically more demanding 1-naphthylamine showed no reaction, as was the case for 2,6-dimethoxyaniline. As may be expected, there appears to be a correlation between the p*K*_a_ of the attacking amines and their reactivity, with those of higher p*K*_a_ being more active, although the p*K*_a_ values of amines do not necessarily correlate with their nucleophilicity (see the Supporting Information for more details).^19^ The sluggishness of benzene-1,4-diamine in forming **43** is likely due to the protonation of one of the amines (p*K*_a_: 6.3 c.f. p*K*_a_: 3.7 formic acid).

**Table 3:**
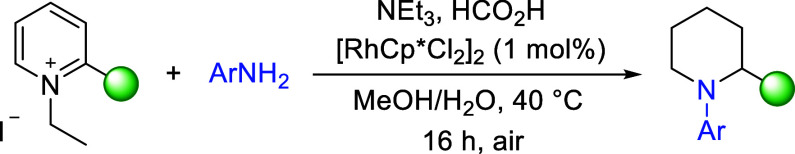

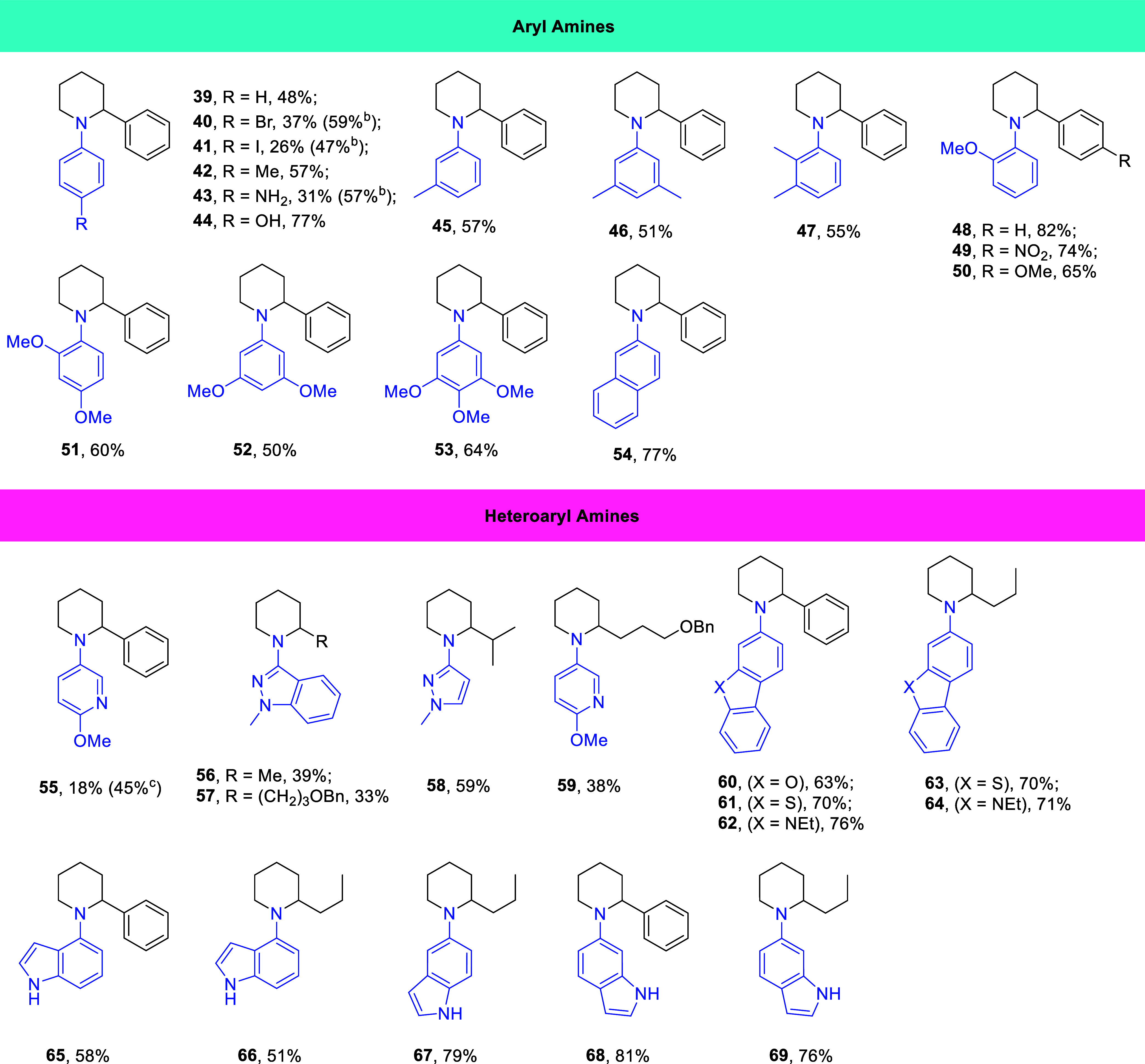
Reductive Transamination with Various (Hetero)aryl Amines^a^

a Reaction conditions were the same as in Table 2. Isolated yields are reported.

b Reaction was carried out for 30 h.

c 12 equiv of HCO_2_H was used.

N-Heteroarylation of piperidine has become an essential strategy for the preparation of potential drug molecules.^2,20^ However, engaging heterocycles in C–N coupling reactions can be challenging.^6^ Delightfully, a range of N-, O-, and S-containing heteroaromatics underwent the reductive amination with 2-aryl and 2-alkylpyridinium salts, affording N-heteroarylated piperidines **55–69**. The yields of these products varied, again with those with higher p*K*_a_ generally affording higher yields (see the Supporting Information). The N-heteroaryl piperidine **55** was obtained only in 18% yield under the standard conditions (24 equiv of formic acid); the yield increased to 45% when 12, instead of 24, equiv of formic acid was used. A lower concentration of acid is expected to give rise to a higher concentration of neutral, attacking amines. However, there are N-heterocyclic amines that showed very low reactivities under the current conditions, likely due to their low nucleophilicity (see the Supporting Information).

**Scheme 2:**
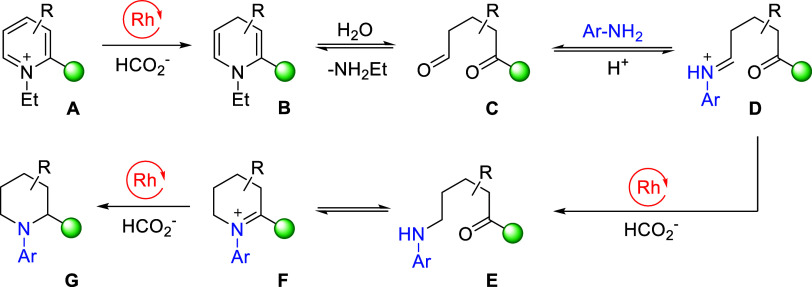
Proposed Mechanism for the Formation of N-Aryl Piperidines via Reductive Transamination For the mechanism of how [Cp*RhCl_2_]_2_ generates the active Rh(III)-H from HCOOH and catalyzes the reduction of iminium ions, see ref (13).

Based on the previous mechanistic studies of the asymmetric reduction of pyridinium salts^13b,21^ and our recent research on ART,^12^ a plausible mechanism is proposed and shown in Scheme 2. The Rh-catalyzed transfer hydrogenation of pyridinium **A** first affords a dihydropyridine **B**, which is intercepted by water, leading to its ring-opening to give **C**. Reductive amination of the dicarbonyl intermediate with the exogenous amine then follows, affording the amino ketone **E** via reduction of the iminium ion **D**. Finally, an intramolecular reductive amination occurs, converting **E** to the N-aryl piperidine product **G** via the tetrahydropyridinium ion **F**. Interestingly, a recent study has shown that **F** can be exploited for accessing functionalized N-(hetero)aryl piperidines.^22^

## Conclusions

In conclusion, a reductive transamination-based catalytic approach for the preparation of N-(hetero)aryl piperidines from readily available pyridinium salts has been established. The method demonstrates broad substrate tolerance, particularly toward substrates that feature functionalities that may interfere with other catalytic processes and operate under simple reaction conditions, requiring neither elaborate ligands nor inert gas protection. The reductive transamination is triggered by rhodium-catalyzed transfer hydrogenation of the pyridinium ring with formic acid with the intermediate dihydropyridine intercepted by water and an exogenous amine. Subsequent ring closure leads to an N-arylated piperidine. Offering a new pathway for converting pyridines to piperidines, the reaction should be of value to synthetic chemistry and enrich the toolbox of dearomatization and skeletal editing.^23^

## Data Availability

The data underlying this study are available in the published article and its Supporting Information.

## Supporting Information Available

Experimental details and procedures, compound characterization data, ^1^H and ^13^C{^1^H} NMR spectra, and the X-ray structure data of compounds **31** and **35** (PDF)
