# Supplementary material for: Reductive Transamination of Pyridinium Salts to N-Aryl Piperidines
Source: J Org Chem. 2024 Jun 13;89(13):9352–9. doi: 10.1021/acs.joc.4c00493 (PMC11232014; doi:10.1021/acs.joc.4c00493)
Supplement: Supplementary file 1 — jo4c00493_si_001.pdf [file jo4c00493_si_001.pdf]

Reductive Transamination of Pyridinium Salts to N-Aryl Piperidines

Zhenyu Chen,[1] Geyang Song,[2] Leiming Qi,[1] Ramachandran Gunasekar,[1] Christophe Aïssa,[1] Craig Robertson,[1] Alexander Steiner,[1] Dong Xue[2] and Jianliang Xiao*[1]

[1] Department of Chemistry, University of Liverpool, Liverpool, L69 7ZD, UK

[2] Key Laboratory of Applied Surface and Colloid Chemistry, Ministry of Education and School of Chemistry and Chemical Engineering, Shaanxi Normal University, Xi'an, 710119, China

Table of Contents

1. General information 2
2. Preparation of pyridinium salts 3
3. General procedure for transamination of pyridiniums 3
4. Optimization of reaction conditions with *p*-anisidine 3
5. Effect of pK_a of amines on product yield 5
6. Analytic data of isolated products 6
7. NMR spectra of isolated products 36
8. X-Ray crystallographic data 116
9. References 118

1. General information

Reagents: Unless otherwise specified, the chemicals were purchased from commercial suppliers (Sigma-Aldrich, Alfa Aesar, Fluorochem, Apollo Scientific and TCI) and used without further purification.

Solvents: Acetonitrile and methanol were used as purchased and water was distilled.

Chromatography: Thin layer chromatography (TLC) was carried out on silica gel plates (GF_{254}) and silica gel (230-400 mesh) was used for flash column chromatography.

NMR spectroscopy: ^{1}H NMR, $^{13}C\{^{1}H\}$ NMR and $^{19}F\{^{1}H\}$ NMR spectra were recorded on a Bruker Avance 400 spectrometer. The solvent signals were used as references for ^{1}H and $^{13}C\{^{1}H\}$ spectra ($CDCl_3$: $\delta^{1}H = 7.26$, $\delta^{13}C = 77.0$ ppm; DMSO-d_6: $\delta^{1}H = 2.50$, $\delta^{13}C = 39.5$ ppm). Coupling constants (J) were reported in hertz (Hz). The following abbreviations are used to indicate the multiplicity of the signals: s = singlet, d = doublet, t = triplet, m = multiplet, and associated combinations, e.g. dd = doublet of doublets.

Mass spectroscopy: High resolution mass spectra (HRMS) were recorded on the Agilent 6540A Accurate-Mass Q-ToF MS with Agilent Jetstream Source (ESI); m/z values were calculated using the software Agilent MassHunter Qualitative Analysis Navigator for the molecular formula of the product (M) with an additional positive ion (H^+).

Crystallography: Single crystals of **31** and **35** suitable for X-ray diffraction were grown by carefully layering n-hexane over a dichloromethane solution of the compound. The diffraction data of crystals were collected on a Bruker D8 Venture diffractometer equipped with a Photon100 CMOS APS detector and a microfocus sealed tube using MoKa radiation (l = 0.71073 Å). The crystal structures were refined with SHELX by full-matrix least squares against F2 using all data.[1] Both structures are disordered as explained in Figure S3. All atoms apart from the methoxy group were split on two positions and refined with enhanced rigid-bond restraints (RIGU) and similar distance restraints for chemically equivalent bonds (SAME).

For the 3-fluoro-substituted piperidines, a minor diastereomer, in which the fluorine is *trans* to the 2-substitutent, could be observed, where the d.r. can be easily determined by integrating the singlets in the $^{19}F\{^{1}H\}$ NMR spectra. The configurations of selected products were assigned by X-ray diffraction and/or NMR analysis and those of the rest by analogy.

2. Preparation of pyridinium salts

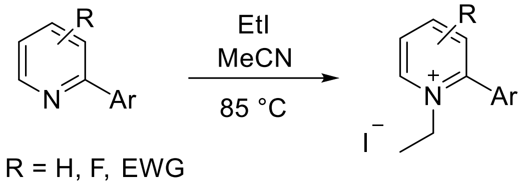

Standard alkylation procedures were followed according to the literature.[2] An example is given below. Synthesis of 1-ethyl-2-phenylpyridin-1-ium iodide: To a carousel reaction tube containing 2-phenylpyridine (620.8 mg, 4.0 mmol) was added ethyl iodide (1.25 g, 8.0 mmol) followed by introducing acetonitrile (4.0 mL). The mixture was stirred in an oil bath at 85 °C in the dark for 18-24 h. After completion, the solvent was removed under reduced pressure, followed by addition of ethyl acetate. The resulting suspension was filtered, and the solid was washed with diethyl ether and dried under vacuum to give 1-ethyl-2-phenylpyridin-1-ium iodide as a yellow crystalline solid.

3. General procedure for transamination of pyridiniums

Synthesis of **1**: To a carousel reaction tube containing a magnetic stirring bar, *p*-anisidine (184.7 mg, 1.5 mmol) and trimethylamine (0.21 mL, 1.5 mmol) was added formic acid (0.27 mL, 7.2 mmol) dropwise at room temperature. After stirring the amine/acid mixture for 10 min, a pyridinium salt, N-ethyl-2-phenylpyridinium iodide (94.2 mg, 0.3 mmol), $[Cp^*RhCl_2]_2$ (1.9 mg, 3 μmol), 3.75 mL of MeOH and 0.25 mL of distilled H_2O were introduced into the mixture. The reaction tube was placed in a carousel parallel reactor. The mixture was stirred in the parallel reactor at 40 °C for 18 h, cooled to room temperature and then basified with an aqueous solution of KOH. The resulting mixture was extracted with ethyl acetate (3×10 mL), dried over Na_2SO_4, filtered and concentrated under reduced pressure The residue was purified by column chromatography (hexane/EtOAc = 10:1) to give the desired product **1** as a colourless oil in 84% yield (112.2 mg).

4. Optimization of reaction conditions with *p*-anisidine

Table S1. Conditions screened in the optimization

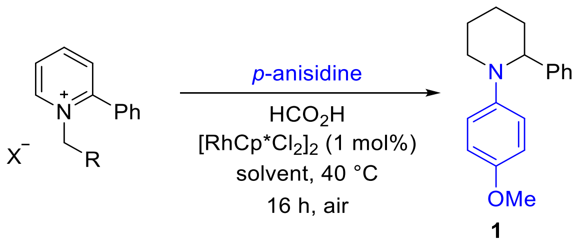

Entry	X, R	Conditions[a]	Yield (%)[b]
1	I, Me	MeOH/H_2O	**86**
2	I, Me	MeOH/H_2O<br>*p*-anisidine (5 equiv)<br>+ NEt_3 (5 equiv)	**84**[c]
3	I, Me	MeOH/H_2O<br>*p*-anisidine (1 equiv)<br>+ NEt_3 (5 equiv)	38
4	I, Me	MeOH/H_2O<br>*p*-anisidine (1 equiv)<br>+ NEt_3 (9 equiv)	55
5	I, Me	CH_2Cl_2/H_2O	11
6	I, Me	THF/H_2O	38
7	I, Me	MeCN/H_2O	74
8	I, Me	EtOH/H_2O	71
9	I, Me	iPrOH/H_2O	59
10	I, Me	MeOH/H_2O, 25 °C	30
11	I, Me	MeOH/H_2O, 60 °C	82
12	I, Me	MeOH/H_2O, N_2	85
13	Br, Ph	MeOH/H_2O	51
14	PF_6, Me	MeOH/H_2O	NA[d]
15	BF_4, Me	MeOH/H_2O	NA[d]

a. Reaction conditions: 0.5 mmol pyridinium salt, 10 equiv of *p*-anisidine, 24 equiv of HCO_2H, CH_2Cl_2/H_2O = 15:1 (4.0 mL), 1 mol% $[Cp^*RhCl_2]_2$, 40 °C, 16 h, in air, unless otherwise indicated. **b.** Isolated yields using flash column chromatography. **c.** Optimized (standard) condition. **d.** No reaction observed.

On examining various reaction conditions while bearing in mind the cost of amines, those shown in entry 2 were selected as the standard conditions for subsequent reactions.

5. Effect of pK_a of amines on product yield

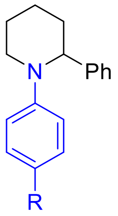

	pK_a value[a]	Nucleophilicity[b]	Yields of piperidines
R = I	3.8		26%
R = Br	3.9		37%
R = Cl	4.0	12.92 (in MeCN)	NA
R = H	4.6	12.64 (in MeCN), 12.99 (in H_2O)	48%
R = Me	5.1	13.19 (in MeCN), 13.00 (in H_2O)	57%
R = OMe	5.3	13.42 (in MeCN), 16.53 (in H_2O)	84%

Figure S1. Effect of the pK_a of the attacking amines on the yields of their corresponding piperidine products (Yields taken from Table 3 in the Article. NA: no desired product observed). **a.** For the anilines, the pK_a values refer to those of the anilinium ions. The pK_a values are taken from the literature.[3] **b.** Nucleophilicity values taken from the literature.[4]

The data above show that the pK_a of amines exerts a significant effect on the product yield of the reductive transamination reaction – the higher the pK_a, the higher the yield. Note that pK_a does not always correlate with nucleophilicity,[4] and it is the latter that presumably dictates the reactivity of an amine (see the proposed mechanism in the Article).

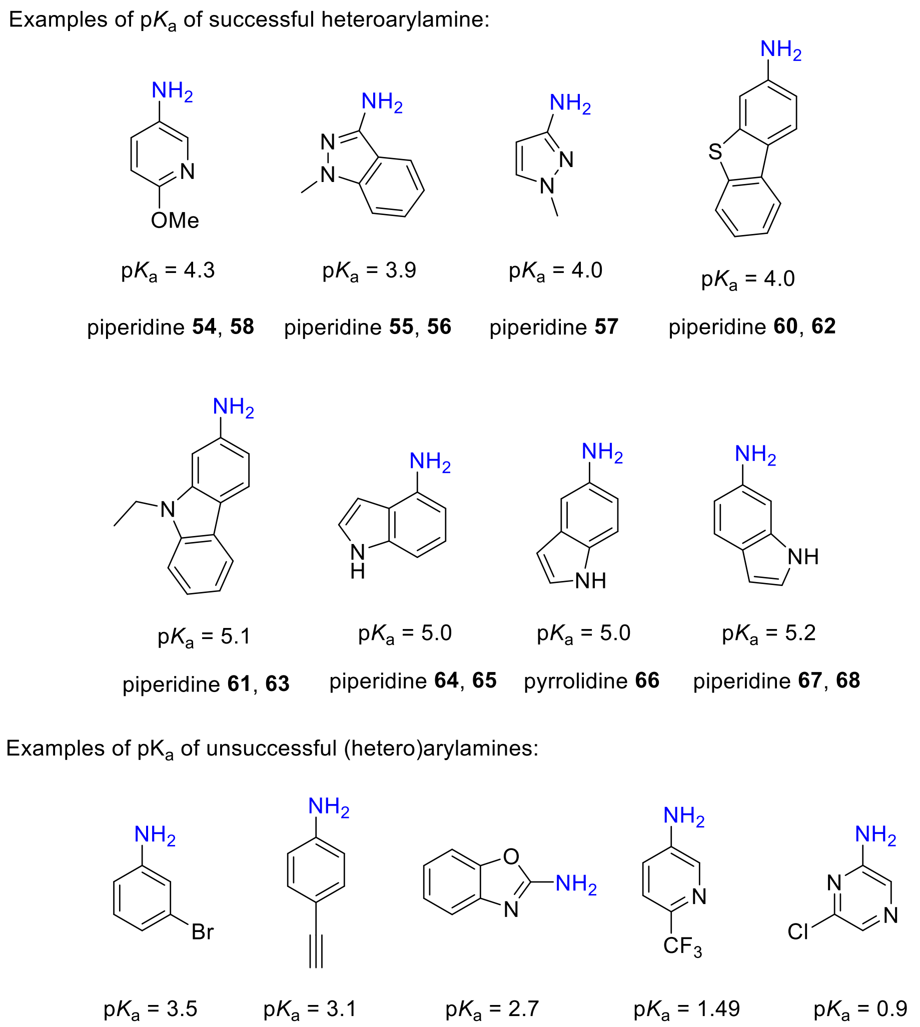

Figure S2. Examples of selected (hetero)arylamine and their pK_a values (predicted with SciFinder^n). The pK_a values refer to those of the protonated amines in blue. Where the reaction worked, the products are indicated.

6. Analytic data of isolated products

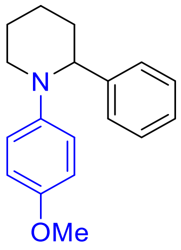

1-(4-Methoxyphenyl)-2-phenylpiperidine (1).[5] Purification by flash chromatography (n-hexane/ethyl acetate = 15:1), colourless oil (112.2 mg, 84%);

^{1}H NMR (400 MHz, $CDCl_3$) δ (ppm): 7.30 (d, *J* = 8.0 Hz, 2H), 7.26 – 7.17 (m, 2H), 7.16 – 7.04 (m, 1H), 6.95 (d, *J* = 8.5 Hz, 2H), 6.71 (d, *J* = 8.5 Hz, 2H), 4.08 (dd, *J* = 9.5, 3.2 Hz, 1H), 3.71 (s, 3H), 3.48 – 3.36 (m, 1H), 2.97 – 2.87 (m, 1H), 2.04 – 1.93 (m, 1H), 1.93 – 1.73 (m, 4H), 1.64 – 1.47 (m, 1H);

^{13}C{^{1}H} NMR (101 MHz, $CDCl_3$) δ (ppm): 154.7, 146.4, 144.8, 128.1, 127.6, 126.3, 123.9, 113.9, 64.6, 56.5, 55.4, 36.3, 26.6, 24.3;

HRMS for $C_{18}H_{22}NO$ [M+H]$^+$: m/z calcd 268.1696, found 268.1700.

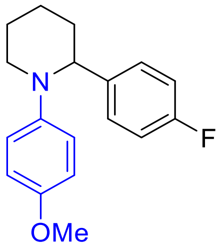

2-(4-Fluorophenyl)-1-(4-methoxyphenyl)piperidine (2). Purification by flash chromatography (n-hexane/ethyl acetate = 9:1), pale yellow oil (108.3 mg, 76%);

^{1}H NMR (400 MHz, $CDCl_3$) δ (ppm): 7.20 (dd, *J* = 8.3, 5.6 Hz, 2H), 6.96 – 6.77 (m, 4H), 6.67 (d, *J* = 8.5 Hz, 2H), 3.98 (dd, *J* = 9.9, 3.0 Hz, 1H), 3.69 (s, 3H), 3.42 – 3.28 (m, 1H), 2.91 – 2.77 (m, 1H), 1.97 – 1.61 (m, 5H), 1.59 – 1.40 (m, 1H);

^{13}C{^{1}H} NMR (101 MHz, $CDCl_3$) δ (ppm): 161.3 (d, *J* = 243.8 Hz), 155.0, 146.2, 140.5 (d, *J* = 3.1 Hz), 128.9 (d, *J* = 7.8 Hz), 124.4, 114.9 (d, *J* = 21.0 Hz), 113.9, 64.2, 56.9, 55.4, 36.6, 26.6, 24.4;

^{19}F{^{1}H} NMR (376 MHz, $CDCl_3$) δ (ppm): -116.92;

HRMS for $C_{18}H_{21}FNO$ [M+H]$^+$: m/z calcd 286.1602, found 286.1596.

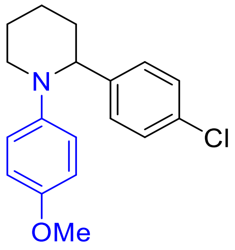

2-(4-Chlorophenyl)-1-(4-methoxyphenyl)piperidine (3). Purification by flash chromatography (n-hexane/ethyl acetate = 10:1), pale yellow oil (121.9 mg, 81%);

^{1}H NMR (400 MHz, $CDCl_3$) δ (ppm): 7.18 (d, *J* = 8.3 Hz, 2H), 7.12 (d, *J* = 8.4 Hz, 2H), 6.88 (d, *J* = 8.7 Hz, 2H), 6.67 (d, *J* = 8.7 Hz, 2H), 3.97 (dd, *J* = 9.9, 3.0 Hz, 1H), 3.69 (s, 3H), 3.40 – 3.28 (m, 1H), 2.89 – 2.76 (m, 1H), 1.95 – 1.61 (m, 5H), 1.59 – 1.40 (m, 1H);

^{13}C{^{1}H} NMR (101 MHz, $CDCl_3$) δ (ppm): 155.1, 146.1, 143.5, 131.7, 128.9, 128.3, 124.3, 113.9, 64.2, 57.0, 55.4, 36.5, 26.6, 24.4;

HRMS for $C_{18}H_{21}ClNO$ [M+H]$^+$: m/z calcd 302.1307, found 302.1298.

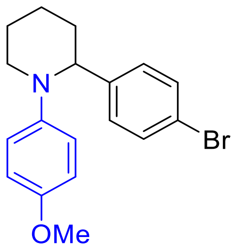

2-(4-Bromophenyl)-1-(4-methoxyphenyl)piperidine (4). Purification by flash chromatography (n-hexane/ethyl acetate = 10:1), pale yellow oil (129.8 mg, 75%);

^{1}H NMR (400 MHz, CDCl$_3$) δ (ppm): 7.32 – 7.27 (m, 2H), 7.17 – 7.11 (m, 2H), 6.94 – 6.86 (m, 2H), 6.72 – 6.63 (m, 2H), 3.98 (dd, *J* = 9.8, 3.2 Hz, 1H), 3.71 (s, 3H), 3.42 – 3.28 (m, 1H), 2.90 – 2.77 (m, 1H), 1.97 – 1.59 (m, 5H), 1.58 – 1.43 (m, 1H);

^{13}C{^{1}H} NMR (101 MHz, CDCl$_3$) δ (ppm): 155.1, 146.1, 144.1, 131.3, 129.3, 124.3, 119.9, 113.9, 64.2, 57.0, 55.4, 36.5, 26.6, 24.4;

HRMS for $C_{18}H_{21}BrNO$ [M+H]$^+$: m/z calcd 346.0802, 348.0781, found 346.0805, 348.0784.

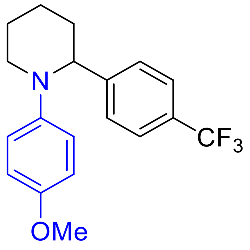

1-(4-Methoxyphenyl)-2-(4-(trifluoromethyl)phenyl)piperidine (5). Purification by flash chromatography (n-hexane/ethyl acetate = 10:1), yellow oil (125.7 mg, 75%);

^{1}H NMR (400 MHz, CDCl$_3$) δ (ppm): 7.49 – 7.30 (m, 4H), 6.89 (d, *J* = 8.5 Hz, 2H), 6.67 (d, *J* = 8.8 Hz, 2H), 4.07 (dd, *J* = 9.9, 3.1 Hz, 1H), 3.69 (s, 3H), 3.44 – 3.30 (m, 1H), 2.94 – 2.75 (m, 1H), 2.00 – 1.42 (m, 6H);

^{13}C{^{1}H} NMR (101 MHz, CDCl$_3$) δ (ppm): 155.2, 149.2, 146.0, 128.5 (q, *J* = 32.0 Hz), 127.8, 125.2 (q, *J* = 3.8 Hz), 124.4 (q, *J* = 273.7 Hz), 124.2, 114.1, 64.4, 57.1, 55.4, 36.6, 26.6, 24.4;

^{19}F{^{1}H} NMR (376 MHz, CDCl$_3$) δ (ppm): -62.30;

HRMS for $C_{19}H_{21}F_3NO$ [M+H]$^+$: m/z calcd 336.1570, found 336.1574.

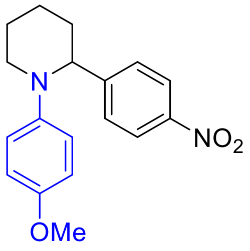

1-(4-Methoxyphenyl)-2-(4-nitrophenyl)piperidine (6). Purification by flash chromatography (n-hexane/ethyl acetate = 8:1), yellow oil (121.7 mg, 78%);

^{1}H NMR (400 MHz, $CDCl_3$) δ (ppm): 8.01 (d, J = 8.8 Hz, 2H), 7.42 (d, J = 8.7 Hz, 2H), 6.88 (d, J = 8.9 Hz, 2H), 6.65 (d, J = 9.0 Hz, 2H), 4.09 (dd, J = 10.2, 2.9 Hz, 1H), 3.67 (s, 3H), 3.42 – 3.30 (m, 1H), 2.86 – 2.75 (m, 1H), 1.97 – 1.73 (m, 4H), 1.71 – 1.57 (m, 1H), 1.57 – 1.43 (m, 1H);

^{13}C{^{1}H} NMR (101 MHz, $CDCl_3$) δ (ppm): 155.5, 153.0, 146.5, 145.7, 128.3, 124.6, 123.6, 114.1, 64.6, 57.5, 55.4, 36.6, 26.5, 24.4;

HRMS for $C_{18}H_{21}N_2O_3$ [M+H]$^+$: m/z calcd 313.1547, found 313.1542.

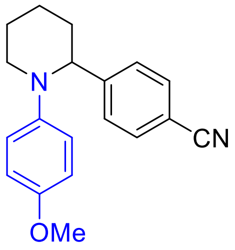

4-(1-(4-Methoxyphenyl)piperidin-2-yl)benzonitrile (7). Purification by flash chromatography (n-hexane/ethyl acetate = 10:1), yellow oil (84.7 mg, 58%);

^{1}H NMR (400 MHz, $CDCl_3$) δ (ppm): 7.44 (d, J = 8.3 Hz, 2H), 7.35 (d, J = 8.3 Hz, 2H), 6.86 (d, J = 8.9 Hz, 2H), 6.65 (d, J = 8.9 Hz, 2H), 4.03 (dd, J = 10.1, 3.0 Hz, 1H), 3.68 (s, 3H), 3.40 – 3.29 (m, 1H), 2.86 – 2.75 (m, 1H), 1.93 – 1.72 (m, 4H), 1.70 – 1.56 (m, 1H), 1.56 – 1.43 (m, 1H);

^{13}C{^{1}H} NMR (101 MHz, $CDCl_3$) δ (ppm): 155.3, 150.8, 145.8, 132.1, 128.3, 124.5, 119.2, 114.1, 110.1, 64.7, 57.3, 55.4, 36.5, 26.5, 24.4;

HRMS for $C_{19}H_{21}N_2O$ [M+H]$^+$: m/z calcd 293.1649, found 293.1640.

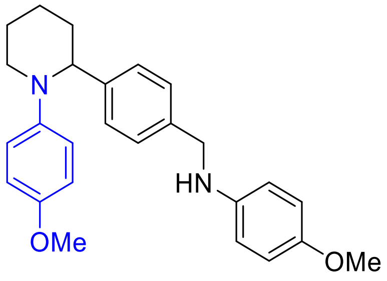

4-Methoxy-*N*-(4-(1-(4-methoxyphenyl)piperidin-2-yl)benzyl)aniline (by-product of 7). Purification by flash chromatography (n-hexane/ethyl acetate = 10:1), pale yellow oil (64.4 mg, 32%);

^{1}H NMR (400 MHz, $CDCl_3$) δ (ppm): 7.22 (d, J = 8.0 Hz, 2H), 7.16 (d, J = 8.0 Hz, 2H), 6.90 (d, J = 9.0 Hz, 2H), 6.77 (d, J = 8.9 Hz, 2H), 6.67 (d, J = 8.9 Hz, 2H), 6.57 (d, J = 8.8 Hz, 2H), 4.14 (s, 2H), 4.04 (dd, J = 9.5, 3.3 Hz, 1H), 3.74 (s, 3H), 3.69 (s, 3H), 3.40 – 3.33 (m, 1H), 2.93 – 2.82 (m, 1H), 1.97 – 1.88 (m, 1H), 1.87 – 1.67 (m, 4H), 1.58 – 1.43 (m, 1H);

^{13}C{^{1}H} NMR (101 MHz, $CDCl_3$) δ (ppm): 154.7, 152.2, 146.4, 143.9, 142.8, 137.4, 127.8, 127.6, 123.9, 114.9, 114.2, 113.9, 64.3, 56.6, 55.9, 55.4, 49.2, 36.3, 26.6, 24.3;

HRMS for $C_{26}H_{31}N_2O_2$ [M+H]$^+$: m/z calcd 403.2381, found 403.2374.

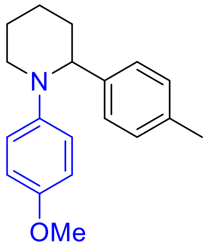

1-(4-Methoxyphenyl)-2-(*p*-tolyl)piperidine (8). Purification by flash chromatography (n-hexane/ethyl acetate = 10:1), yellow oil (106.9 mg, 76%);

^{1}H NMR (400 MHz, CDCl$_3$) δ (ppm): 7.14 (d, *J* = 7.8 Hz, 2H), 6.99 (d, *J* = 7.8 Hz, 2H), 6.91 (d, *J* = 8.8 Hz, 2H), 6.68 (d, *J* = 8.9 Hz, 2H), 4.02 (dd, *J* = 9.4, 3.3 Hz, 1H), 3.69 (s, 3H), 3.44 – 3.32 (m, 1H), 2.93 – 2.83 (m, 1H), 2.25 (s, 3H), 1.97 – 1.87 (m, 1H), 1.87 – 1.68 (m, 4H), 1.61 – 1.42 (m, 1H);

^{13}C{^{1}H} NMR (101 MHz, CDCl$_3$) δ (ppm): 154.7, 146.5, 141.8, 135.7, 128.9, 127.4, 123.8, 113.9, 64.2, 56.5, 55.4, 36.3, 26.6, 24.3, 21.2;

HRMS for $C_{19}H_{24}NO$ [M+H]$^+$: m/z calcd 282.1853, found 282.1850.

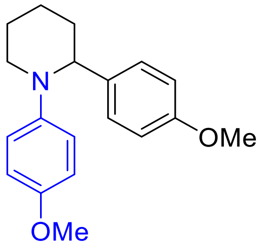

1,2-Bis(4-methoxyphenyl)piperidine (9). Purification by flash chromatography (n-hexane/ethyl acetate = 10:1), yellow oil (120.4 mg, 81%);

^{1}H NMR (400 MHz, CDCl$_3$) δ (ppm): 7.16 (d, *J* = 8.2 Hz, 2H), 6.91 (d, *J* = 8.4 Hz, 2H), 6.76 – 6.61 (m, 4H), 3.99 (dd, *J* = 9.6, 3.2 Hz, 1H), 3.72 (s, 3H), 3.69 (s, 3H), 3.42 – 3.28 (m, 1H), 2.96 – 2.79 (m, 1H), 2.00 – 1.65 (m, 5H), 1.61 – 1.41 (m, 1H);

^{13}C{^{1}H} NMR (101 MHz, CDCl$_3$) δ (ppm): 157.9, 154.8, 146.5, 136.9, 128.5, 124.0, 113.9, 113.5, 64.0, 56.6, 55.4, 55.2, 36.3, 26.6, 24.3;

HRMS for $C_{19}H_{24}NO_2$ [M+H]$^+$: m/z calcd 298.1802, found 298.1799.

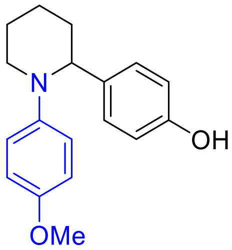

4-(1-(4-Methoxyphenyl)piperidin-2-yl)phenol (10). Purification by flash chromatography (n-hexane/ethyl acetate = 10:1), colourless oil (106.2 mg, 75%);

^{1}H NMR (400 MHz, $CDCl_3$) δ (ppm): 7.13 – 7.06 (m, 2H), 6.97 – 6.88 (m, 2H), 6.73 – 6.66 (m, 2H), 6.65 – 6.57 (m, 2H), 3.95 (dd, *J* = 9.6, 3.2 Hz, 1H), 3.71 (s, 3H), 3.41 – 3.30 (m, 1H), 2.96 – 2.84 (m, 1H), 1.98 – 1.70 (m, 5H), 1.61 – 1.43 (m, 1H);

^{13}C{^{1}H} NMR (101 MHz, $CDCl_3$) δ (ppm): 154.8, 154.0, 146.3, 136.6, 128.8, 124.3, 115.2, 113.9, 64.6, 56.7, 55.5, 36.1, 26.5, 24.4;

HRMS for $C_{18}H_{22}NO_2$ [M+H]$^+$: m/z calcd 284.1646, found 284.1642.

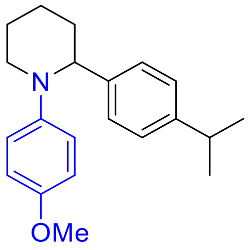

2-(4-Isopropylphenyl)-1-(4-methoxyphenyl)piperidine (11). Purification by flash chromatography (n-hexane/ethyl acetate = 10:1), pale yellow oil (116.0 mg, 75%);

^{1}H NMR (400 MHz, $CDCl_3$) δ (ppm): 7.16 (d, *J* = 8.1 Hz, 2H), 7.07 – 7.00 (m, 2H), 6.94 – 6.87 (m, 2H), 6.73 – 6.64 (m, 2H), 4.08 (dd, *J* = 8.9, 3.5 Hz, 1H), 3.69 (s, 3H), 3.45 – 3.30 (m, 1H), 2.98 – 2.88 (m, 1H), 2.82 (hept, *J* = 7.0 Hz, 1H), 2.01 – 1.88 (m, 1H), 1.88 – 1.67 (m, 4H), 1.60 – 1.43 (m, 1H), 1.20 (d, *J* = 7.1 Hz, 6H);

^{13}C{^{1}H} NMR (101 MHz, $CDCl_3$) δ (ppm): 154.4, 146.6, 146.5, 141.8, 127.4, 126.2, 123.3, 113.9, 63.9, 55.7, 55.4, 35.9, 33.7, 26.5, 24.1, 24.0;

HRMS for $C_{21}H_{28}NO$ [M+H]$^+$: m/z calcd 310.2166, found 310.2172.

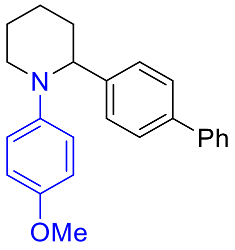

2-([1,1'-Biphenyl]-4-yl)-1-(4-methoxyphenyl)piperidine (12). Purification by flash chromatography (n-hexane/ethyl acetate = 10:1), pale yellow oil (133.9 mg, 78%);

^{1}H NMR (400 MHz, $CDCl_3$) δ (ppm): 7.58 – 7.49 (m, 2H), 7.45 – 7.34 (m, 4H), 7.34 – 7.27 (m, 2H), 6.96 – 6.89 (m, 2H), 6.71 – 6.64 (m, 2H), 4.09 (dd, *J* = 9.4, 3.3 Hz, 1H), 3.68 (s, 3H), 3.43 – 3.33 (m, 1H), 2.96 – 2.83 (m, 1H), 2.03 – 1.69 (m, 5H), 1.62 – 1.46 (m, 1H);

^{13}C{^{1}H} NMR (101 MHz, $CDCl_3$) δ (ppm): 154.6, 146.3, 143.8, 140.9, 138.9, 128.6, 127.8, 126.9, 126.9, 126.7, 123.7, 113.9, 64.1, 56.3, 55.3, 36.1, 26.5, 24.1;

HRMS for $C_{24}H_{26}NO$ [M+H]$^+$: m/z calcd 344.2009, found 344.2006.

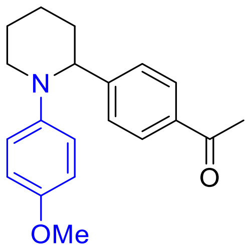

1-(4-(1-(4-Methoxyphenyl)piperidin-2-yl)phenyl)ethan-1-one (13). Purification by flash chromatography (n-hexane/ethyl acetate = 15:1), yellow oil (58.7 mg, 38%);

^{1}H NMR (400 MHz, $CDCl_3$) δ (ppm): 7.76 (d, *J* = 8.3 Hz, 2H), 7.34 (d, *J* = 8.2 Hz, 2H), 6.89 (d, *J* = 8.9 Hz, 2H), 6.65 (d, *J* = 8.9 Hz, 2H), 4.06 (dd, *J* = 9.9, 3.2 Hz, 1H), 3.67 (s, 3H), 3.43 – 3.29 (m, 1H), 2.88 – 2.77 (m, 1H), 2.50 (s, 3H), 1.95 – 1.63 (m, 5H), 1.59 – 1.39 (m, 1H);

^{13}C{^{1}H} NMR (101 MHz, $CDCl_3$) δ (ppm): 198.0, 155.1, 150.8, 146.1, 135.5, 128.5, 127.7, 124.3, 114.0, 64.6, 57.2, 55.4, 36.4, 26.6, 26.6, 24.4;

HRMS for $C_{20}H_{24}NO_2$ [M+H]$^+$: m/z calcd 310.1802, found 310.1796.

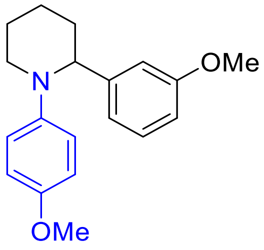

2-(3-Methoxyphenyl)-1-(4-methoxyphenyl)piperidine (14). Purification by flash chromatography (n-hexane/ethyl acetate = 10:1), yellow oil (121.8 mg, 82%);

^{1}H NMR (400 MHz, $CDCl_3$) δ (ppm): 7.09 – 7.05 (m, 1H), 6.94 – 6.87 (m, 2H), 6.86 – 6.79 (m, 2H), 6.70 – 6.63 (m, 2H), 6.63 – 6.56 (m, 1H), 4.01 (dd, *J* = 9.4, 3.3 Hz, 1H), 3.71 (s, 3H), 3.68 (s, 3H), 3.40 – 3.30 (m, 1H), 2.91 – 2.80 (m, 1H), 1.97 – 1.65 (m, 5H), 1.55 – 1.41 (m, 1H);

^{13}C{^{1}H} NMR (101 MHz, $CDCl_3$) δ (ppm): 159.5, 154.8, 146.6, 146.5, 129.1, 123.8, 120.1, 113.9, 113.2, 111.7, 64.5, 56.5, 55.4, 55.2, 36.2, 26.5, 24.2;

HRMS for $C_{19}H_{24}NO_2$ [M+H]$^+$: m/z calcd 298.1802, found 298.1795.

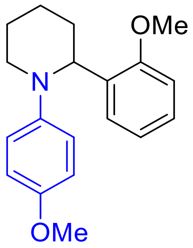

2-(2-Methoxyphenyl)-1-(4-methoxyphenyl)piperidine (15). Purification by flash chromatography (n-hexane/ethyl acetate = 10:1), yellow oil (68.4 mg, 46%);

^{1}H NMR (400 MHz, CDCl$_3$) δ (ppm): 7.31 (d, *J* = 7.6 Hz, 1H), 7.08 – 6.99 (m, 1H), 6.92 – 6.84 (m, 2H), 6.80 – 6.70 (m, 2H), 6.67 – 6.61 (m, 2H), 4.46 (dd, *J* = 9.7, 3.2 Hz, 1H), 3.85 (s, 3H), 3.67 (s, 3H), 3.49 – 3.41 (m, 1H), 2.86 – 2.75 (m, 1H), 1.99 – 1.70 (m, 4H), 1.67 – 1.43 (m, 2H);

^{13}C{^{1}H} NMR (101 MHz, CDCl$_3$) δ (ppm): 156.2, 154.7, 146.8, 133.2, 128.3, 126.9, 123.7, 120.7, 113.8, 110.2, 58.1, 56.2, 55.6, 55.4, 34.9, 26.9, 24.6;

HRMS for $C_{19}H_{24}NO_2$ [M+H]$^+$: m/z calcd 298.1802, found 298.1805.

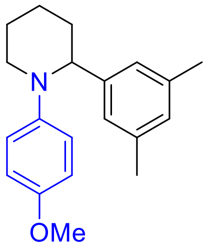

2-(3,5-Dimethylphenyl)-1-(4-methoxyphenyl)piperidine (16). Purification by flash chromatography (n-hexane/ethyl acetate = 10:1), yellow oil (112.1 mg, 76%);

^{1}H NMR (400 MHz, CDCl$_3$) δ (ppm): 6.95 – 6.88 (m, 2H), 6.86 (s, 2H), 6.72 (s, 1H), 6.71 – 6.63 (m, 2H), 3.98 (dd, *J* = 9.4, 3.4 Hz, 1H), 3.69 (s, 3H), 3.43 – 3.30 (m, 1H), 2.92 – 2.82 (m, 1H), 2.21 (s, 6H), 1.98 – 1.86 (m, 1H), 1.86 – 1.62 (m, 4H), 1.57 – 1.40 (m, 1H);

^{13}C{^{1}H} NMR (101 MHz, CDCl$_3$) δ (ppm): 154.6, 146.6, 144.7, 137.4, 127.9, 125.3, 123.5, 113.9, 64.4, 56.3, 55.4, 36.1, 26.5, 24.2, 21.5;

HRMS for $C_{20}H_{26}NO$ [M+H]$^+$: m/z calcd 296.2009, found 296.2022.

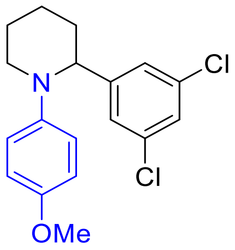

2-(3,5-Dichlorophenyl)-1-(4-methoxyphenyl)piperidine (17). Purification by flash chromatography (n-hexane/ethyl acetate = 10:1), yellow oil (114.3 mg, 68%);

^{1}H NMR (400 MHz, CDCl$_3$) δ (ppm): 7.15 (d, *J* = 2.0 Hz, 2H), 7.06 – 7.04 (m, 1H), 6.94 – 6.84 (m, 2H), 6.73 – 6.66 (m, 2H), 3.95 (dd, *J* = 9.9, 3.2 Hz, 1H), 3.70 (s, 3H), 3.38 – 3.27 (m, 1H), 2.86 – 2.74 (m, 1H), 1.96 – 1.72 (m, 4H), 1.71 – 1.56 (m, 1H), 1.55 – 1.39 (m, 1H);

^{13}C{^{1}H} NMR (101 MHz, CDCl$_3$) δ (ppm): 155.4, 148.7, 145.7, 134.6, 126.6, 126.1, 124.3, 114.2, 64.1, 56.9, 55.4, 36.4, 26.4, 24.2;

HRMS for $C_{18}H_{20}Cl_2NO$ [M+H]$^+$: m/z calcd 336.0917, found 336.0916.

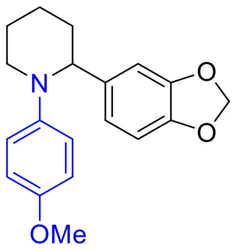

2-(Benzo[*d*][1,3]dioxol-5-yl)-1-(4-methoxyphenyl)piperidine (18). Purification by flash chromatography (n-hexane/ethyl acetate = 15:1), yellow solid (136.9 mg, 88%);

^{1}H NMR (400 MHz, CDCl$_3$) δ (ppm): 6.90 (d, *J* = 8.4 Hz, 2H), 6.78 (s, 1H), 6.71 – 6.65 (m, 3H), 6.60 (d, *J* = 8.0 Hz, 1H).5.84 (s, 2H), 3.91 (dd, *J* = 9.9, 3.1 Hz, 1H), 3.70 (s, 3H), 3.40 – 3.25 (m, 1H), 2.88 – 2.72 (m, 1H), 1.96 – 1.55 (m, 5H), 1.55 – 1.36 (m, 1H);

^{13}C{^{1}H} NMR (101 MHz, CDCl$_3$) δ (ppm): 154.9, 147.5, 146.4, 145.8, 139.1, 124.3, 120.6, 113.9, 107.9, 107.9, 100.8, 64.5, 56.9, 55.4, 36.6, 26.6, 24.4;

HRMS for $C_{19}H_{22}NO_3$ [M+H]$^+$: m/z calcd 312.1595, found 312.1594.

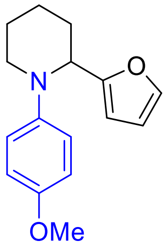

2-(Furan-2-yl)-1-(4-methoxyphenyl)piperidine (19). Purification by flash chromatography (n-hexane/ethyl acetate = 15:1), yellow oil (91.3 mg, 71%);

^{1}H NMR (400 MHz, CDCl$_3$) δ (ppm): 7.32 – 7.20 (m, 1H), 6.95 – 6.86 (m, 2H), 6.82 – 6.72 (m, 2H), 6.24 – 6.15 (m, 1H), 5.91 (d, *J* = 3.2 Hz, 1H), 4.62 – 4.54 (m, 1H), 3.75 (s, 3H), 3.24 – 3.12 (m, 2H), 2.22 – 2.09 (m, 1H), 2.06 – 1.93 (m, 1H), 1.88 – 1.51 (m, 4H);

^{13}C{^{1}H} NMR (101 MHz, CDCl$_3$) δ (ppm): 155.4, 153.9, 145.7, 140.9, 120.2, 114.2, 109.9, 107.3, 56.9, 55.6, 49.1, 30.6, 26.0, 21.7;

HRMS for $C_{16}H_{20}NO_2$ [M+H]$^+$: m/z calcd 258.1489, found 258.1495.

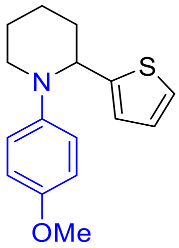

1-(4-Methoxyphenyl)-2-(thiophen-2-yl)piperidine (20). Purification by flash chromatography (n-hexane/ethyl acetate = 15:1), yellow oil (95.5 mg, 70%);

^{1}H NMR (400 MHz, $CDCl_3$) δ (ppm): 7.11 – 7.04 (m, 1H), 6.98 – 6.90 (m, 2H), 6.84 – 6.78 (m, 1H), 6.79 – 6.71 (m, 3H), 4.71 (dd, *J* = 6.5, 4.2 Hz, 1H), 3.73 (s, 3H), 3.30 – 3.20 (m, 1H), 3.15 – 3.05 (m, 1H), 2.17 – 1.91 (m, 2H), 1.89 – 1.68 (m, 3H), 1.68 – 1.50 (m, 1H);

^{13}C{^{1}H} NMR (101 MHz, $CDCl_3$) δ (ppm): 154.3, 147.4, 145.7, 126.1, 124.8, 123.8, 121.5, 114.2, 59.1, 55.5, 51.1, 34.4, 25.9, 22.3;

HRMS for $C_{16}H_{20}NOS$ [M+H]$^+$: m/z calcd 274.1261, found 274.1269.

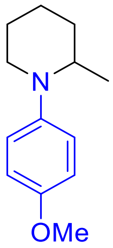

1-(4-Methoxyphenyl)-2-methylpiperidine (21). Purification by flash chromatography (n-hexane/ethyl acetate = 10:1), pale yellow oil (80.1 mg, 78%);

^{1}H NMR (400 MHz, $CDCl_3$) δ (ppm): 7.01 – 6.94 (m, 2H), 6.86 – 6.77 (m, 2H), 3.77 (s, 3H), 3.44 – 3.31 (m, 1H), 3.04 – 2.95 (m, 1H), 2.93 – 2.83 (m, 1H), 1.90 – 1.80 (m, 1H), 1.77 – 1.62 (m, 3H), 1.57 – 1.45 (m, 2H), 0.90 (d, J = 6.4 Hz, 3H);

^{13}C{^{1}H} NMR (101 MHz, $CDCl_3$) δ (ppm): 154.7, 146.1, 122.3, 114.3, 55.6, 54.3, 50.5, 33.4, 26.6, 21.9, 16.5;

HRMS for $C_{13}H_{20}NO$ [M+H]$^+$: m/z calcd 206.1540, found 206.1539.

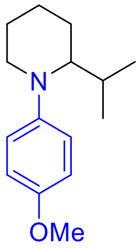

2-Isopropyl-1-(4-methoxyphenyl)piperidine (22). Purification by flash chromatography (n-hexane/ethyl acetate = 10:1), pale yellow oil (97.9 mg, 84%);

^{1}H NMR (400 MHz, $CDCl_3$) δ (ppm): 6.96 (d, *J* = 8.4 Hz, 2H), 6.83 (d, *J* = 8.4 Hz, 2H), 3.78 (s, 3H), 3.14 – 3.00 (m, 2H), 3.01 – 2.87 (m, 1H), 2.09 – 1.89 (m, 1H), 1.86 – 1.72 (m, 1H), 1.71 – 1.39 (m, 5H), 0.84 (t, *J* = 5.9 Hz, 6H);

^{13}C{^{1}H} NMR (101 MHz, $CDCl_3$) δ (ppm): 153.8, 146.4, 121.4, 114.4, 64.3, 55.6, 50.9, 27.3, 25.5, 24.7, 22.4, 20.4, 17.9;

HRMS for $C_{15}H_{24}NO$ [M+H]$^+$: m/z calcd 234.1853, found 234.1849.

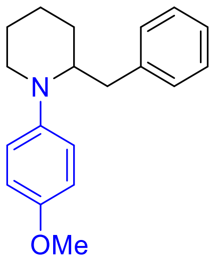

2-Benzyl-1-(4-methoxyphenyl)piperidine (23). Purification by flash chromatography (n-hexane/ethyl acetate = 10:1), pale yellow oil (71.7 mg, 51%);

^{1}H NMR (400 MHz, CDCl$_3$) δ (ppm): 7.36 – 7.24 (m, 4H), 7.25 – 7.14 (m, 1H), 6.67 (d, *J* = 8.6 Hz, 2H), 6.38 (d, *J* = 8.6 Hz, 2H), 3.75 – 3.71 (m, 1H), 3.72 (s, 3H), 3.45 – 3.32 (m, 1H), 3.05 – 2.95 (m, 1H), 2.17 – 2.04 (m, 1H), 2.01 – 1.80 (m, 3H), 1.70 – 1.38 (m, 4H);

^{13}C{^{1}H} NMR (101 MHz, CDCl$_3$) δ (ppm): 151.9, 144.2, 142.4, 128.4, 127.8, 126.3, 115.1, 114.8, 55.9, 54.9, 46.5, 30.4, 26.1, 25.9, 20.5;

HRMS for $C_{19}H_{24}NO$ [M+H]$^+$: m/z calcd 282.1853, found 282.1851.

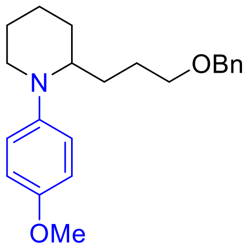

2-(3-(Benzyloxy)propyl)-1-(4-methoxyphenyl)piperidine (24). Purification by flash chromatography (n-hexane/ethyl acetate = 10:1), pale yellow oil (84.8 mg, 50%);

^{1}H NMR (400 MHz, CDCl$_3$) δ (ppm): 7.43 – 7.29 (m, 5H), 7.00 (d, *J* = 8.5 Hz, 2H), 6.84 (d, *J* = 8.5 Hz, 2H), 4.56 (s, 2H), 4.18 – 4.04 (m, 1H), 3.79 (s, 3H), 3.77 – 3.66 (m, 2H), 3.43 – 3.32 (m, 2H), 3.31 – 3.12 (m, 2H), 2.04 – 1.93 (m, 1H), 1.86 – 1.48 (m, 7H);

^{13}C{^{1}H} NMR (101 MHz, CDCl$_3$) δ (ppm): 153.8, 145.7, 138.6, 128.5, 127.8, 127.7, 120.1, 114.6, 73.3, 68.3, 66.1, 63.7, 55.7, 46.5, 33.5, 23.3, 23.3, 20.9;

HRMS for $C_{22}H_{30}NO_2$ [M+H]$^+$: m/z calcd 340.2272, found 340.2271.

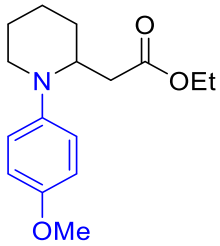

Ethyl 2-(1-(4-methoxyphenyl)piperidin-2-yl)acetate (25). Purification by flash chromatography (n-hexane/ethyl acetate = 10:1), pale yellow oil (90.1 mg, 65%);

^{1}H NMR (400 MHz, CDCl$_3$) δ (ppm): 6.79 – 6.72 (m, 2H), 6.63 – 6.56 (m, 2H), 4.17 – 4.01 (m, 2H), 3.73 (s, 3H), 3.70 – 3.64 (m, 1H), 2.85 – 2.78 (m, 1H), 2.03 – 1.94 (m, 1H), 1.91 – 1.80 (m, 1H), 1.71 – 1.62 (m, 3H), 1.57 – 1.30 (m, 3H), 1.25 – 1.13 (m, 1H), 1.21 (t, *J* = 7.1 Hz, 3H);

^{13}C{^{1}H} NMR (101 MHz, CDCl$_3$) δ (ppm): 174.2, 152.3, 141.4, 115.6, 115.0, 60.4, 55.9, 53.0, 44.9, 29.1, 26.0, 23.5, 22.9, 14.4;

HRMS for $C_{16}H_{24}NO_3$ [M+H]$^+$: m/z calcd 278.1751, found 278.1755.

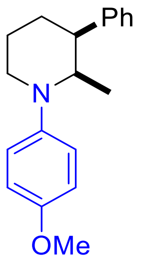

1-(4-Methoxyphenyl)-2-methyl-3-phenylpiperidine (26). Purification by flash chromatography (n-hexane/ethyl acetate = 15:1), the product was isolated in a mixture of diastereomers (d.r. 5:1) as a yellow oil (43.6 mg, 31%);

^{1}H NMR (400 MHz, CDCl$_3$) δ (ppm): 7.40 – 7.20 (m, 5H), 6.97 – 6.90 (m, 2H), 6.90 – 6.80 (m, 2H), 4.16 – 4.04 (m, 1H), 3.79 (s, 3H), 3.37 – 3.20 (m, 2H), 3.07 – 2.95 (m, 1H), 2.14 – 1.93 (m, 2H), 1.91 – 1.76 (m, 2H), 0.68 (d, *J* = 6.7 Hz, 3H);

^{13}C{^{1}H} NMR (101 MHz, CDCl$_3$) δ (ppm): 153.0, 145.3, 143.9, 128.4, 127.9, 126.3, 118.5, 114.6, 58.6, 55.7, 45.9, 42.2, 26.0, 22.6, 6.5;

HRMS for $C_{19}H_{24}NO$ [M+H]$^+$: m/z calcd 282.1853, found 282.1855.

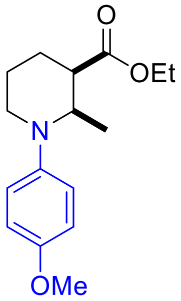

Ethyl 1-(4-methoxyphenyl)-2-methylpiperidine-3-carboxylate (27). Purification by flash chromatography (n-hexane/ethyl acetate = 15:1), the product was isolated in a mixture of diastereomers (d.r. 3:1) as a yellow oil (91.4 mg, 66%);

^{1}H NMR (400 MHz, CDCl$_3$) δ (ppm): 6.90 (d, *J* = 9.1 Hz, 2H), 6.83 (d, *J* = 9.1 Hz, 2H), 4.33 – 4.23 (m, 1H), 4.16 (q, *J* = 7.1 Hz, 2H), 3.77 (s, 3H), 3.18 – 3.06 (m, 1H), 2.98 – 2.84 (m, 2H), 1.92 – 1.54 (m, 4H), 1.27 (t, *J* = 7.1 Hz, 3H), 0.85 (d, *J* = 6.7 Hz, 3H);

^{13}C{^{1}H} NMR (101 MHz, CDCl$_3$) δ (ppm): 173.7, 153.3, 144.9, 118.9, 114.6, 60.5, 55.7, 54.3, 45.9, 42.0, 24.9, 20.3, 14.4, 7.9;

HRMS for $C_{16}H_{24}NO_3$ [M+H]$^+$: m/z calcd 278.1751, found 278.1755.

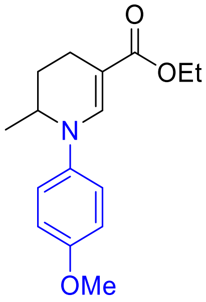

Ethyl 1-(4-methoxyphenyl)-6-methyl-1,4,5,6-tetrahydropyridine-3-carboxylate (28). Purification by flash chromatography (n-hexane/ethyl acetate = 8:1), yellow oil (103.1 mg, 75%);

^{1}H NMR (400 MHz, CDCl$_3$) δ (ppm): 7.69 (s, 1H), 7.03 (d, *J* = 8.9 Hz, 2H), 6.86 (d, *J* = 8.9 Hz, 2H), 4.17 (q, *J* = 7.1 Hz, 2H), 4.04 – 3.97 (m, 1H), 3.78 (s, 3H), 2.53 – 2.43 (m, 1H), 2.36 – 2.23 (m, 1H), 1.86 – 1.78 (m, 2H), 1.27 (t, *J* = 7.1 Hz, 3H), 1.17 (d, *J* = 6.6 Hz, 3H);

^{13}C{^{1}H} NMR (101 MHz, CDCl$_3$) δ (ppm): 168.7, 156.1, 140.5, 139.2, 120.9, 114.7, 98.8, 59.2, 55.6, 51.5, 27.1, 17.9, 16.8, 14.8;

HRMS for $C_{16}H_{22}NO_3$ [M+H]$^+$: m/z calcd 276.1595, found 276.1603.

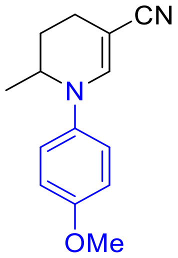

1-(4-Methoxyphenyl)-6-methyl-1,4,5,6-tetrahydropyridine-3-carbonitrile (29). Purification by flash chromatography (n-hexane/ethyl acetate = 8:1), yellow oil (77.5 mg, 68%);

^{1}H NMR (400 MHz, CDCl$_3$) δ (ppm): 7.09 (s, 1H), 6.97 (d, *J* = 9.0 Hz, 2H), 6.86 (d, *J* = 9.0 Hz, 2H), 4.09 – 3.92 (m, 1H), 3.79 (s, 3H), 2.45 – 2.31 (m, 1H), 2.30 – 2.18 (m, 1H), 1.97 – 1.73 (m, 2H), 1.17 (d, *J* = 6.6 Hz, 3H);

^{13}C{^{1}H} NMR (101 MHz, CDCl$_3$) δ (ppm): 156.6, 142.7, 138.5, 123.1, 121.3, 114.9, 77.4, 55.7, 51.4, 26.8, 18.8, 17.9;

HRMS for $C_{14}H_{17}N_2O$ [M+H]$^+$: m/z calcd 229.1336, found 229.1332.

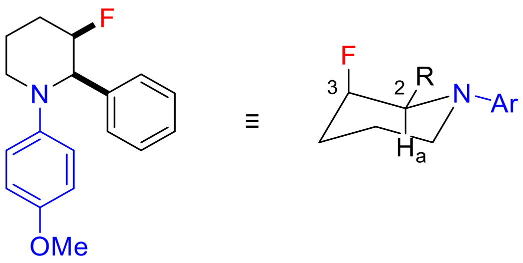

3-Fluoro-1-(4-methoxyphenyl)-2-phenylpiperidine (30). The product was isolated in a mixture of diastereomers (d.r. 5:1 cis/trans) as a yellow oil (102.6 mg, 72%) after column chromatography (n-hexane/EtOAc 20:1).

The orientation of the fluorine atom in the major isomer is assigned as axial due to the large value of ^3J(F, H_a).[6] **[^3J(3-F, 2-H_a) = 26.9 Hz]**.

^{1}H NMR (400 MHz, $CDCl_3$) δ (ppm): 7.35 – 7.26 (m, 2H), 7.23 – 7.06 (m, 3H), 6.92 (d, J = 8.8 Hz, 2H), 6.66 (d, J = 8.8 Hz, 2H), 4.77 (d, J = 47.4 Hz, 1H), 4.16 (d, J = 26.9 Hz, 1H), 3.68 (s, 3H), 3.48 – 3.37 (m, 1H), 2.91 – 2.77 (m, 1H), 2.32 – 2.13 (m, 2H), 1.98 – 1.60 (m, 2H);

^{13}C{^{1}H} NMR (101 MHz, $CDCl_3$) δ (ppm): 155.3, 145.5, 139.8, 128.9, 127.9, 127.0, 124.4, 114.0, 90.7 (d, J = 180.7 Hz), 67.2 (d, J = 17.4 Hz), 56.9, 55.4, 29.3 (d, J = 21.7 Hz), 21.2;

^{19}F{^{1}H} NMR (376 MHz, $CDCl_3$) δ (ppm): -193.74;

HRMS for $C_{18}H_{21}FNO$ [M+H]$^+$: m/z calcd 286.1602, found 286.1602.

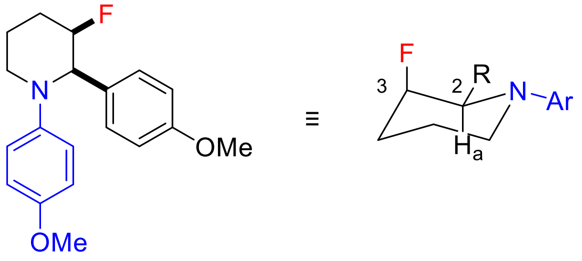

3-Fluoro-1,2-bis(4-methoxyphenyl)piperidine (31). The product was isolated in a mixture of diastereomers (d.r. 6:1 cis/trans) as a yellow oil (102.4 mg, 65%) after column chromatography (n-hexane/EtOAc 20:1);

The orientation of the fluorine atom in the major isomer is assigned as axial due to the large value of ^3J(F, H_a). **[^3J(3-F, 2-H_a) = 26.7 Hz]**.

^{1}H NMR (400 MHz, $CDCl_3$) δ (ppm): 7.22(d, J = 8.4 Hz, 2H), 6.92 (d, J = 8.4 Hz, 2H), 6.72 (d, J = 8.4 Hz, 2H), 6.67 (d, J = 8.4 Hz, 2H), 4.74 (d, J = 48.7 Hz, 1H), 4.11 (d, J = 26.7 Hz, 1H), 3.72 (s, 3H), 3.68 (s, 3H), 3.47 – 3.36 (m, 1H), 2.91 – 2.77 (m, 1H), 2.30 – 2.12 (m, 2H), 1.96 – 1.58 (m, 2H);

^{13}C{^{1}H} NMR (101 MHz, $CDCl_3$) δ (ppm): 158.5, 155.2, 145.6, 131.9, 129.9, 124.5, 113.9, 113.4, 90.9 (d, J = 180.3 Hz), 66.5 (d, J = 17.5 Hz), 56.8, 55.4, 55.2, 29.4 (d, J = 21.7 Hz), 21.2;

^{19}F{^{1}H} NMR (376 MHz, $CDCl_3$) δ (ppm): -193.69;

HRMS for $C_{19}H_{23}FNO_2$ [M+H]$^+$: m/z calcd 316.1707, found 316.1704.

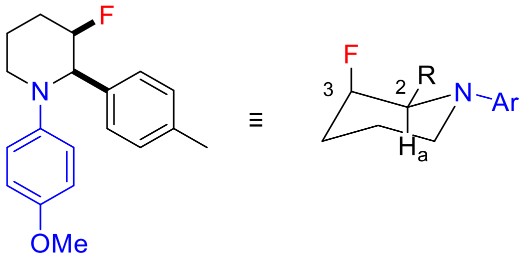

3-Fluoro-1-(4-methoxyphenyl)-2-(p-tolyl)piperidine (32). The product was isolated in a mixture of diastereomers (d.r. 6:1 cis/trans) as a yellow oil (110.6 mg, 74%) after column chromatography (n-hexane/EtOAc 20:1);

The orientation of the fluorine atom in the major isomer is assigned as axial due to the large value of ^3J(F, H_a). **[^3J(3-F, 2-H_a) = 26.8 Hz]**.

^{1}H NMR (400 MHz, $CDCl_3$) δ (ppm): 7.21 (d, J = 7.7 Hz, 2H), 7.01 (d, J = 7.7 Hz, 2H), 6.94 (d, J = 8.8 Hz, 2H), 6.69 (d, J = 8.9 Hz, 2H). 4.75 (d, J = 47.2 Hz, 1H), 4.14 (d, J = 26.8 Hz, 1H), 3.68 (s, 3H), 3.49 – 3.37 (m, 1H), 2.89 – 2.78 (m, 1H), 2.24 (s, 3H), 2.28 – 2.18 (m, 2H), 1.97 – 1.62 (m, 2H);

^{13}C{^{1}H} NMR (101 MHz, $CDCl_3$) δ (ppm): 155.2, 145.6, 136.7, 136.5, 128.6 (d, J = 1.6 Hz), 128.7, 124.4, 113.9, 90.8 (d, J = 180.6 Hz), 66.8 (d, J = 17.6 Hz), 56.8, 55.4, 29.4 (d, J = 21.7 Hz), 21.2 (d, J = 2.1 Hz), 21.1;

^{19}F{^{1}H} NMR (376 MHz, $CDCl_3$) δ (ppm): -193.46;

HRMS for $C_{19}H_{23}FNO$ [M+H]$^+$: m/z calcd 300.1758, found 300.1752.

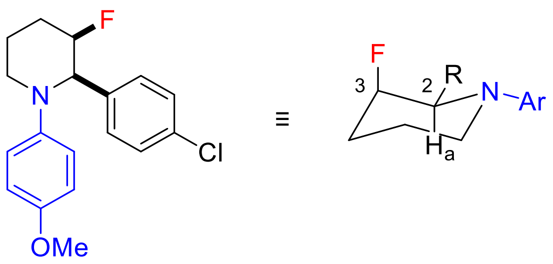

2-(4-Chlorophenyl)-3-fluoro-1-(4-methoxyphenyl)piperidine (33). The product was isolated in a mixture of diastereomers (d.r. 6:1 cis/trans) as a yellow oil (95.7 mg, 60%) after column chromatography (n-hexane/EtOAc 20:1);

The orientation of the fluorine atom in the major isomer is assigned as axial due to the large value of ^3J(F, H_a). **[^3J(3-F, 2-H_a) = 26.9 Hz]**.

^{1}H NMR (400 MHz, $CDCl_3$) δ (ppm): 7.27 (d, J = 8.5 Hz, 2H), 7.17 (d, J = 8.5 Hz, 2H), 6.92 (d, J = 8.9 Hz, 2H), 6.69 (d, J = 8.9 Hz, 2H), 4.74 (d, J = 47.3 Hz, 1H), 4.14 (d, J = 26.9 Hz, 1H), 3.71 (s, 3H), 3.48 – 3.36 (m, 1H), 2.91 – 2.77 (m, 1H), 2.32 – 2.16 (m, 2H), 1.90 – 1.63 (m, 2H);

^{13}C{^{1}H} NMR (101 MHz, $CDCl_3$) δ (ppm): 155.5, 145.2, 138.4, 132.6, 130.3, 128.2, 124.7, 114.1, 90.4 (d, J = 181.0 Hz), 66.7 (d, J = 17.4 Hz), 57.1, 55.4, 29.4 (d, J = 21.7 Hz), 21.1 (d, J = 2.0 Hz);

^{19}F{^{1}H} NMR (376 MHz, $CDCl_3$) δ (ppm): -193.31;

HRMS for $C_{18}H_{20}ClFNO$ [M+H]$^+$: m/z calcd 320.1212, found 320.1204.

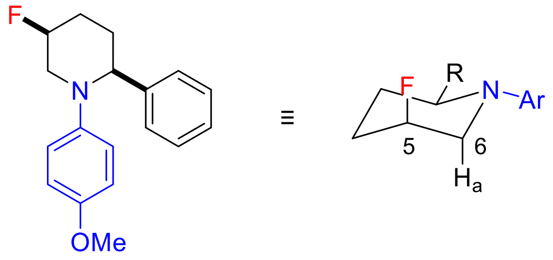

5-Fluoro-1-(4-methoxyphenyl)-2-phenylpiperidine (34). The product was isolated in a mixture of diastereomers (d.r. 4:1 cis/trans) as a yellow oil (106.9 mg, 75%) after column chromatography (n-hexane/EtOAc 15:1);

The orientation of the fluorine atom in the major isomer is assigned as axial due to the large value of ^3J(F, H_a). **[^3J(5-F, 6-H_a) = 30.5 Hz]**.

^{1}H NMR (400 MHz, $CDCl_3$) δ (ppm): 7.35 – 7.26 (m, 2H), 7.26 – 7.18 (m, 2H), 7.17 – 7.10 (m, 1H), 6.93 (d, J = 7.1 Hz, 2H), 6.71 (d, J = 7.1 Hz, 2H), 4.87 (d, J = 48.1 Hz, 1H), 4.21 (d, J = 8.7 Hz, 1H), 3.71 (s, 3H), 3.63 – 3.52 (m, 1H), 3.22 (dd, J = 30.5, 13.4 Hz, 1H), 2.29 – 2.01 (m, 2H), 2.01 – 1.72 (m, 2H);

^{13}C{^{1}H} NMR (101 MHz, $CDCl_3$) δ (ppm): 154.8, 145.1, 143.3, 128.3, 127.5, 126.7, 123.2, 114.2, 87.2 (d, J = 174.3 Hz), 62.9, 57.6 (d, J = 21.7 Hz), 55.5, 30.3 (d, J = 2.9 Hz), 28.8 (d, J = 20.9 Hz);

^{19}F{^{1}H} NMR (376 MHz, $CDCl_3$) δ (ppm): -183.67;

HRMS for $C_{18}H_{21}FNO$ $[M+H]^+$: m/z calcd 286.1602, found 286.1606.

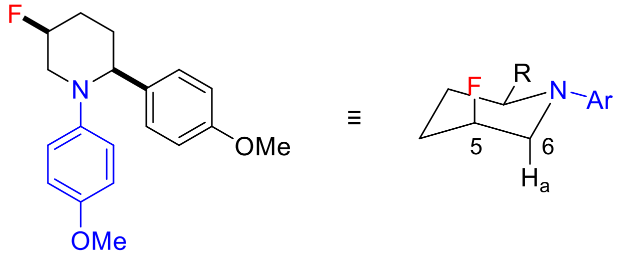

5-Fluoro-1,2-bis(4-methoxyphenyl)piperidine (35). The product was isolated in a mixture of diastereomers (d.r. 5:1 cis/trans) as a yellow oil (121.3 mg, 77%) after column chromatography (n-hexane/EtOAc 15:1);

The orientation of the fluorine atom in the major isomer is assigned as axial due to the large value of ^3J(F, H_a). **[^3J(5-F, 6-H_a) = 30.5 Hz]**.

^{1}H NMR (400 MHz, $CDCl_3$) δ (ppm): 7.18 (d, J = 6.8 Hz, 2H), 6.89 (d, J = 7.6 Hz, 2H), 6.73 (d, J = 6.8 Hz, 2H), 6.68 (d, J = 7.6 Hz, 2H).4.83 (d, J = 49.1 Hz, 1H), 4.12 (d, J = 8.8 Hz, 1H), 3.73 (s, 3H), 3.69 (s, 3H), 3.58 – 3.47 (m, 1H), 3.17 (dd, J = 30.5, 13.3 Hz, 1H), 2.22 – 1.98 (m, 2H), 1.95 – 1.72 (m, 2H);

^{13}C{^{1}H} NMR (101 MHz, $CDCl_3$) δ (ppm): 158.2, 154.8, 145.2, 135.3, 128.5, 123.3, 114.1, 113.7, 87.3 (d, J = 174.1 Hz), 62.4, 57.6 (d, J = 21.7 Hz), 55.4, 55.2, 30.3 (d, J = 2.9 Hz), 28.8 (d, J = 20.8 Hz);

^{19}F{^{1}H} NMR (376 MHz, $CDCl_3$) δ (ppm): -184.41;

HRMS for $C_{19}H_{23}FNO_2$ [M+H]$^+$: m/z calcd 316.1708, found 316.1707.

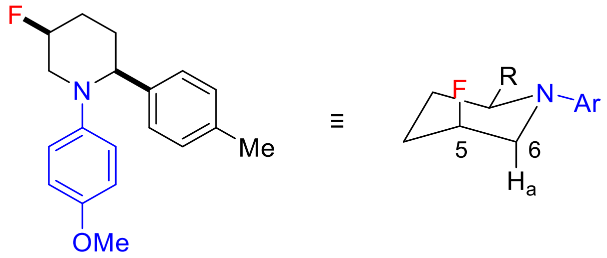

5-Fluoro-1-(4-methoxyphenyl)-2-(p-tolyl)piperidine (36). The product was isolated in a mixture of diastereomers (d.r. 4:1 cis/trans) as a yellow oil (104.7 mg, 70%) after column chromatography (n-hexane/EtOAc 15:1);

The orientation of the fluorine atom in the major isomer is assigned as axial due to the large value of ^{3}J(F, H_a). **[^{3}J(5-F, 6-H_a) = 29.6 Hz].**

^{1}H NMR (400 MHz, $CDCl_3$) δ (ppm): 7.17 (d, J = 7.6 Hz, 2H), 7.01 (d, J = 7.6 Hz, 2H), 6.91 (d, J = 7.1 Hz, 2H), 6.69 (d, J = 7.1 Hz, 2H), 4.83 (d, J = 48.1 Hz, 1H), 4.22 – 4.11 (m, 1H), 3.70 (s, 3H), 3.63 – 3.45 (m, 1H), 3.20 (dd, J = 29.6, 13.6 Hz, 1H), 2.25 (s, 3H), 2.22 – 1.98 (m, 2H), 1.97 – 1.72 (m, 2H);

^{13}C{^{1}H} NMR (101 MHz, $CDCl_3$) δ (ppm): 154.7, 145.2, 140.2, 136.1, 129.1, 127.4, 122.9, 114.2, 87.3 (d, J = 174.2 Hz), 62.5, 57.4 (d, J = 21.9 Hz), 55.5, 30.3 (d, J = 3.1 Hz), 28.7 (d, J = 20.8 Hz), 21.2;

^{19}F{^{1}H} NMR (376 MHz, $CDCl_3$) δ (ppm): -183.67;

HRMS for $C_{19}H_{23}FNO$ [M+H]$^+$: m/z calcd 300.1759, found 300.1758.

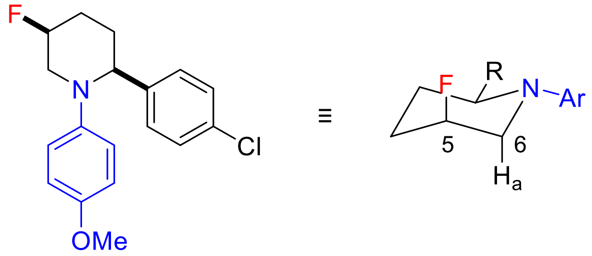

2-(4-Chlorophenyl)-5-fluoro-1-(4-methoxyphenyl)piperidine (37). The product was isolated in a mixture of diastereomers (d.r. 4:1 cis/trans) as a yellow oil (105.3 mg, 66%) after column chromatography (n-hexane/EtOAc 15:1);

The orientation of the fluorine atom in the major isomer is assigned as axial due to the large value of ^{3}J(F, H_a). **[^{3}J(5-F, 6-H_a) = 33.1 Hz].**

^{1}H NMR (400 MHz, $CDCl_3$) δ (ppm): 7.21 (d, J = 7.9 Hz, 2H), 7.15 (d, J = 7.9 Hz, 2H), 6.89 (d, J = 8.5 Hz, 2H), 6.68 (d, J = 8.5 Hz, 2H), 4.85 (d, J = 47.4 Hz, 1H), 4.10 (d, J = 8.7 Hz, 1H), 3.70 (s, 3H), 3.61 – 3.48 (m, 1H), 3.10 (dd, J = 33.1, 13.4 Hz, 1H), 2.17 – 2.02 (m, 2H), 1.94 – 1.71 (m, 2H);

^{13}C{^{1}H} NMR (101 MHz, $CDCl_3$) δ (ppm): 155.2, 144.9, 142.1, 132.2, 128.9, 128.5, 123.9, 114.2, 86.9 (d, J = 174.4 Hz), 62.9, 58.5 (d, J = 21.1 Hz), 55.4, 30.5 (d, J = 2.3 Hz), 28.8 (d, J = 21.2 Hz);

^{19}F{^{1}H} NMR (376 MHz, $CDCl_3$) δ (ppm): -184.39;

HRMS for $C_{18}H_{20}ClFNO$ [M+H]$^+$: m/z calcd 320.1212, found 320.1212.

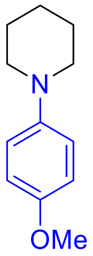

1-(4-Methoxyphenyl)piperidine (38). Purification by flash chromatography (n-hexane/ethyl acetate = 10:1), colourless oil (82.2 mg, 86%);

^{1}H NMR (400 MHz, $CDCl_3$) δ (ppm): 6.97 – 6.90 (m, 2H), 6.89 – 6.81 (m, 2H), 3.78 (s, 3H), 3.14 – 2.96 (m, 4H), 1.83 – 1.69 (m, 4H), 1.66 – 1.49 (m, 2H);

$^{13}C\{^1H\}$ NMR (101 MHz, $CDCl_3$) δ (ppm): 153.6, 147.0, 118.8, 114.4, 55.6, 52.3, 26.2, 24.3.

HRMS for $C_{12}H_{18}NO$ [M+H]$^+$: m/z calcd 192.1383, found 192.1388.

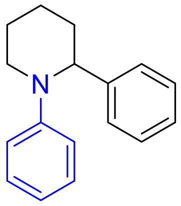

1,2-Diphenylpiperidine (39).[5] Purification by flash chromatography (n-hexane/ethyl acetate = 10:1), yellow oil (56.9 mg, 48%);

^{1}H NMR (400 MHz, $CDCl_3$) δ (ppm): 7.39 – 7.22 (m, 4H), 7.23 – 7.11 (m, 3H), 6.94 (d, J = 8.1 Hz, 2H), 6.82 – 6.78 (m, 1H), 4.55 (dd, J = 6.7, 4.6 Hz, 1H), 3.54 – 3.38 (m, 1H), 3.37 – 3.21 (m, 1H), 2.11 – 1.92 (m, 2H), 1.89 – 1.66 (m, 3H), 1.66 – 1.53 (m, 1H);

$^{13}C\{^1H\}$ NMR (101 MHz, $CDCl_3$) δ (ppm): 151.9, 143.7, 128.9, 128.4, 127.3, 126.3, 119.7, 118.9, 61.1, 50.6, 33.6, 25.8, 22.2;

HRMS for $C_{17}H_{20}N$ [M+H]$^+$: m/z calcd 238.1591, found 238.1593.

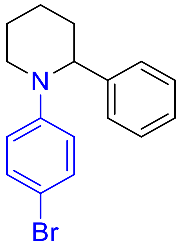

1-(4-Bromophenyl)-2-phenylpiperidine (40). Purification by flash chromatography (n-hexane/ethyl acetate = 8:1), yellow oil (92.9 mg, 59%);

^{1}H NMR (400 MHz, CDCl$_3$) δ (ppm): 7.25 – 7.18 (m, 6H), 7.18 – 7.10 (m, 1H), 6.75 (d, *J* = 8.9 Hz, 2H), 4.44 (dd, *J* = 6.8, 4.5 Hz, 1H), 3.46 – 3.30 (m, 1H), 3.28 – 3.14 (m, 1H), 2.10 – 1.85 (m, 2H), 1.83 – 1.61 (m, 3H), 1.61 – 1.43 (m, 1H);

^{13}C{^{1}H} NMR (101 MHz, CDCl$_3$) δ (ppm): 150.9, 143.3, 131.7, 128.5, 127.2, 126.5, 120.5, 111.9, 61.1, 50.7, 33.6, 25.7, 21.9;

HRMS for $C_{17}H_{19}BrN$ [M+H]$^+$: m/z calcd 316.0696, 318.0675, found 316.0699, 318.0678.

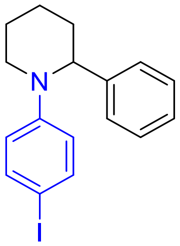

1-(4-Iodophenyl)-2-phenylpiperidine (41). Purification by flash chromatography (n-hexane/ethyl acetate = 10:1), yellow oil (85.7 mg, 47%);

^{1}H NMR (400 MHz, CDCl$_3$) δ (ppm): 7.42 (d, *J* = 8.9 Hz, 2H), 7.31 – 7.23 (m, 4H), 7.23 – 7.13 (m, 1H), 6.67 (d, *J* = 8.9 Hz, 2H), 4.54 (t, *J* = 5.5 Hz, 1H), 3.45 – 3.36 (m, 1H), 3.36 – 3.25 (m, 1H), 2.09 – 1.90 (m, 2H), 1.84 – 1.74 (m, 2H), 1.74 – 1.50 (m, 2H);

^{13}C{^{1}H} NMR (101 MHz, CDCl$_3$) δ (ppm): 151.4, 143.0, 137.7, 128.5, 127.2, 126.5, 120.3, 81.2, 60.5, 49.5, 33.1, 25.5, 21.5;

HRMS for $C_{17}H_{19}IN$ [M+H]$^+$: m/z calcd 364.0557, found 364.0559.

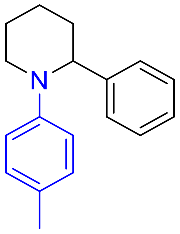

2-Phenyl-1-(*p*-tolyl)piperidine (42). Purification by flash chromatography (n-hexane/ethyl acetate = 10:1), yellow oil (71.5 mg, 57%);

^{1}H NMR (400 MHz, CDCl$_3$) δ (ppm): 7.32 (d, *J* = 7.7 Hz, 2H), 7.29 – 7.21 (m, 2H), 7.21 – 7.12 (m, 1H), 6.99 (d, *J* = 8.1 Hz, 2H), 6.88 (d, *J* = 8.1 Hz, 2H), 4.36 (dd, *J* = 8.1, 3.9 Hz, 1H), 3.52 – 3.39 (m, 1H), 3.20 – 3.07 (m, 1H), 2.24 (s, 3H), 2.09 – 1.97 (m, 1H), 1.97 – 1.71 (m, 4H), 1.67 – 1.49 (m, 1H);

^{13}C{^{1}H} NMR (101 MHz, CDCl$_3$) δ (ppm): 150.0, 144.4, 129.8, 129.4, 128.3, 127.4, 126.3, 120.4, 62.4, 53.2, 34.8, 26.1, 23.1, 20.7;

HRMS for $C_{18}H_{22}N$ [M+H]$^+$: m/z calcd 252.1747, found 252.1742.

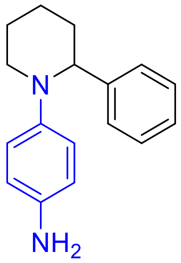

4-(2-Phenylpiperidin-1-yl)aniline (43). Purification by flash chromatography (n-hexane/ethyl acetate = 5:1), yellow oil (71.8 mg, 57%);

^{1}H NMR (400 MHz, $CDCl_3$) δ (ppm): 7.26 – 7.21 (m, 2H), 7.19 – 7.11 (m, 2H), 7.10 – 7.01 (m, 1H), 6.80 (d, *J* = 8.6 Hz, 2H), 6.49 – 6.40 (m, 2H), 3.95 (dd, *J* = 9.9, 3.2 Hz, 1H), 3.59 – 3.03 (m, 3H), 2.89 – 2.75 (m, 1H), 1.95 – 1.63 (m, 5H), 1.58 – 1.40 (m, 1H);

^{13}C{^{1}H} NMR (101 MHz, $CDCl_3$) δ (ppm): 145.2, 145.1, 141.4, 128.1, 127.7, 126.2, 124.5, 115.7, 65.1, 57.2, 36.6, 26.7, 24.6;

HRMS for $C_{17}H_{21}N_2$ [M+H]$^+$: m/z calcd 253.1700, found 253.1701.

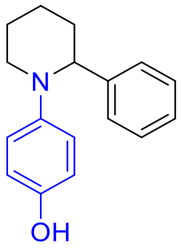

4-(2-Phenylpiperidin-1-yl)phenol (44). Purification by flash chromatography (n-hexane/ethyl acetate = 10:1), yellow oil (97.4 mg, 77%);

^{1}H NMR (400 MHz, $CDCl_3$) δ (ppm): 7.31 – 7.24 (m, 2H), 7.22 – 7.15 (m, 2H), 7.13 – 7.08 (m, 1H), 6.97 – 6.75 (m, 2H), 6.66 – 6.44 (s, 2H), 5.07 (brs, 1H), 4.12 – 3.83 (m, 1H), 3.50 – 3.19 (m, 1H), 3.09 – 2.74 (m, 1H), 2.04 – 1.72 (m, 5H), 1.64 – 1.42 (m, 1H);

^{13}C{^{1}H} NMR (101 MHz, $CDCl_3$) δ (ppm): 150.8, 146.4, 144.7, 128.1, 127.7, 126.4, 124.4, 115.5, 65.1, 56.7, 36.2, 26.5, 24.4;

HRMS for $C_{17}H_{20}NO$ [M+H]$^+$: m/z calcd 254.1540, found 254.1539.

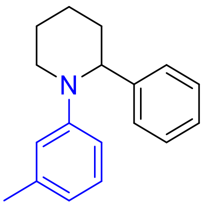

2-Phenyl-1-(m-tolyl)piperidine (45). Purification by flash chromatography (n-hexane/ethyl acetate = 10:1), yellow oil (71.5 mg, 57%);

^{1}H NMR (400 MHz, $CDCl_3$) δ (ppm): 7.39 – 7.24 (m, 4H), 7.24 – 7.13 (m, 1H), 7.12 – 7.01 (m, 1H), 6.79 (s, 1H), 6.74 (d, J = 8.4 Hz, 1H), 6.63 (d, J = 7.5 Hz, 1H), 4.64 – 4.51 (m, 1H), 3.51 – 3.38 (m, 1H), 3.38 – 3.25 (m, 1H), 2.29 (s, 3H), 2.11 – 1.94 (m, 2H), 1.91 – 1.52 (m, 4H);

^{13}C{^{1}H} NMR (101 MHz, $CDCl_3$) δ (ppm): 151.9, 143.7, 138.5, 128.7, 128.4, 127.3, 126.3, 120.4, 119.5, 115.6, 60.9, 50.2, 33.3, 25.7, 22.1, 21.8;

HRMS for $C_{18}H_{22}N$ $[M+H]^+$: m/z calcd 252.1747, found 252.1749.

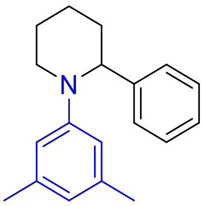

1-(3,5-Dimethylphenyl)-2-phenylpiperidine (46). Purification by flash chromatography (n-hexane/ethyl acetate = 10:1), yellow oil (67.6 mg, 51%);

^{1}H NMR (400 MHz, $CDCl_3$) δ (ppm): 7.36 – 7.27 (m, 4H), 7.22 – 7.12 (m, 1H), 6.56 (s, 2H), 6.45 (s, 1H), 4.60 (t, J = 5.5 Hz, 1H), 3.45 – 3.26 (m, 2H), 2.23 (s, 6H), 2.09 – 1.95 (m, 2H), 1.85 – 1.48 (m, 4H);

^{13}C{^{1}H} NMR (101 MHz, $CDCl_3$) δ (ppm): 151.9, 143.6, 138.4, 128.4, 127.3, 126.3, 121.3, 116.1, 60.7, 49.6, 32.8, 25.7, 21.9, 21.7;

HRMS for $C_{19}H_{24}N$ $[M+H]^+$: m/z calcd 266.1904, found 266.1905.

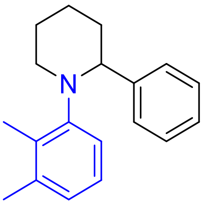

1-(2,3-Dimethylphenyl)-2-phenylpiperidine (47). Purification by flash chromatography (n-hexane/ethyl acetate = 10:1), yellow oil (72.9 mg, 55%);

^{1}H NMR (400 MHz, $CDCl_3$) δ (ppm): 7.22 – 7.14 (m, 2H), 7.14 – 7.06 (m, 2H), 7.04 – 6.95 (m, 1H), 6.84 – 6.76 (m, 2H), 6.76 – 6.64 (m, 1H), 4.03 (d, J = 10.6 Hz, 1H), 3.07 (d, J = 12.1 Hz, 1H), 2.58 – 2.44 (m, 1H), 2.33 (s, 3H), 2.19 (s, 3H), 2.00 – 1.47 (m, 6H);

^{13}C{^{1}H} NMR (101 MHz, $CDCl_3$) δ (ppm): 150.9, 145.3, 137.3, 133.1, 127.9, 127.1, 126.1, 125.0, 124.9, 120.0, 65.4, 56.9, 37.8, 26.7, 25.4, 20.6, 13.4;

HRMS for $C_{19}H_{24}N$ $[M+H]^+$: m/z calcd 266.1904, found 266.1902.

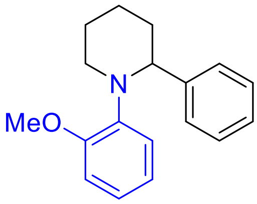

1-(2-Methoxyphenyl)-2-phenylpiperidine (48). Purification by flash chromatography (n-hexane/ethyl acetate = 8:1), yellow oil (108.7 mg, 82%);

^{1}H NMR (400 MHz, $CDCl_3$) δ (ppm): 7.27 (d, *J* = 7.6 Hz, 2H), 7.22 – 7.12 (m, 2H), 7.12 – 7.02 (m, 1H), 6.93 – 6.84 (m, 2H), 6.80 (d, *J* = 8.2 Hz, 1H), 6.73 – 6.60 (m, 1H), 4.17 (d, *J* = 10.5 Hz, 1H), 3.90 (s, 3H), 3.52 (d, *J* = 11.6 Hz, 1H), 2.68 (t, *J* = 11.9 Hz, 1H), 2.01 – 1.69 (m, 5H), 1.67 – 1.48 (m, 1H);

^{13}C{^{1}H} NMR (101 MHz, $CDCl_3$) δ (ppm): 154.6, 145.2, 141.4, 128.1, 127.3, 126.2, 124.4, 123.8, 120.4, 111.7, 64.6, 55.6, 54.9, 37.3, 26.6, 25.2;

HRMS for $C_{19}H_{24}N$ [M+H]$^+$: m/z calcd 266.1904, found 266.1902.

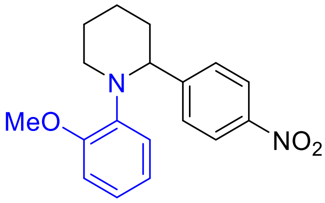

1-(2-Methoxyphenyl)-2-(4-nitrophenyl)piperidine (49). Purification by flash chromatography (n-hexane/ethyl acetate = 10:1), yellow oil (115.4 mg, 74%);

^{1}H NMR (400 MHz, $CDCl_3$) δ (ppm): 7.98 (d, *J* = 6.9 Hz, 2H), 7.41 (d, *J* = 6.9 Hz, 2H), 6.97 – 6.57 (m, 4H), 4.27 (d, *J* = 10.9 Hz, 1H), 3.87 (s, 3H), 3.43 (d, *J* = 11.6 Hz, 1H), 2.68 (t, *J* = 11.8 Hz, 1H), 1.96 – 1.46 (m, 6H);

^{13}C{^{1}H} NMR (101 MHz, $CDCl_3$) δ (ppm): 154.9, 153.2, 146.5, 140.6, 128.0, 124.7, 124.5, 123.6, 120.5, 111.7, 64.2, 55.6, 54.8, 37.1, 26.4, 24.9;

HRMS for $C_{18}H_{21}N_2O_3$ [M+H]$^+$: m/z calcd 313.1547, found 313.1549.

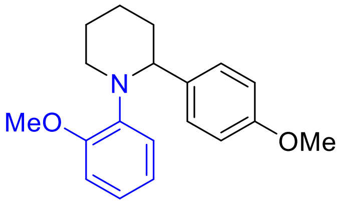

1-(2-Methoxyphenyl)-2-(4-methoxyphenyl)piperidine (50). Purification by flash chromatography (n-hexane/ethyl acetate = 10:1), yellow oil (96.5 mg, 65%);

^{1}H NMR (400 MHz, $CDCl_3$) δ (ppm): 7.14 (d, *J* = 6.9 Hz, 2H), 6.90 – 6.83 (m, 2H), 6.76 (d, *J* = 8.0 Hz, 2H), 6.69 – 6.62 (m, 2H), 4.07 (d, *J* = 10.5 Hz, 1H), 3.86 (s, 3H), 3.69 (s, 3H), 3.45 (d, *J* = 11.1 Hz, 1H), 2.68 – 2.56 (m, 1H), 1.92 – 1.66 (m, 4H), 1.62 – 1.47 (m, 2H);

^{13}C{^{1}H} NMR (101 MHz, $CDCl_3$) δ (ppm): 157.9, 154.7, 141.5, 137.4, 128.2, 124.6, 123.8, 120.4, 113.4, 111.7, 63.9, 55.7, 55.2, 55.1, 37.4, 26.7, 25.2;

HRMS for $C_{19}H_{24}NO_2$ [M+H]$^+$: m/z calcd 298.1802, found 298.1805.

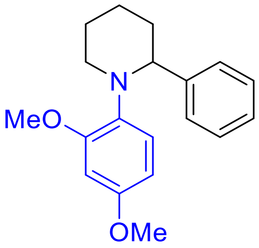

1-(2,4-Dimethoxyphenyl)-2-phenylpiperidine (51). Purification by flash chromatography (n-hexane/ethyl acetate = 10:1), yellow oil (89.1 mg, 60%);

^{1}H NMR (400 MHz, $CDCl_3$) δ (ppm): 7.23 (d, J = 7.6 Hz, 2H), 7.14– 7.10 (m, 2H), 7.06 – 7.02 (m, 1H), 6.79 (d, J = 8.7 Hz, 1H), 6.34 (d, J = 2.7 Hz, 1H), 6.17 (dd, J = 8.6, 2.8 Hz, 1H), 4.06 (d, J = 9.2 Hz, 1H), 3.83 (s, 3H), 3.66 (s, 3H), 3.35 (d, J = 11.4 Hz, 1H), 2.66 (t, J = 11.7 Hz, 1H), 1.94 – 1.65 (m, 5H), 1.59 – 1.43 (m, 1H);

^{13}C{^{1}H} NMR (101 MHz, $CDCl_3$) δ (ppm): 156.6, 155.9, 145.3, 134.9, 127.9, 127.4, 126.2, 125.2, 103.4, 99.7, 65.2, 55.7, 55.6, 55.3, 37.5, 26.7, 25.3;

HRMS for $C_{19}H_{24}NO_2$ [M+H]$^+$: m/z calcd 298.1802, found 298.1802.

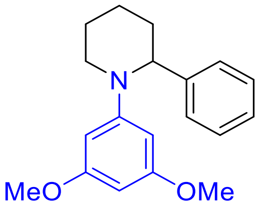

1-(3,5-Dimethoxyphenyl)-2-phenylpiperidine (52). Purification by flash chromatography (n-hexane/ethyl acetate = 10:1), yellow oil (74.3 mg, 50%);

^{1}H NMR (400 MHz, $CDCl_3$) δ (ppm): 7.29 – 7.21 (m, 4H), 7.18 – 7.11 (m, 1H), 6.06 (d, J = 2.2 Hz, 2H), 5.94 – 5.89 (m, 1H), 4.58 (t, J = 5.3 Hz, 1H), 3.68 (s, 6H), 3.38 – 3.31 (m, 2H), 2.04 – 1.95 (m, 2H), 1.77 – 1.69 (m, 2H), 1.68 – 1.49 (m, 2H);

^{13}C{^{1}H} NMR (101 MHz, $CDCl_3$) δ (ppm): 161.3, 153.6, 143.3, 128.5, 127.2, 126.4, 96.6, 91.3, 60.5, 55.3, 48.8, 32.5, 25.5, 21.4;

HRMS for $C_{19}H_{24}NO_2$ [M+H]$^+$: m/z calcd 298.1802, found 298.1804.

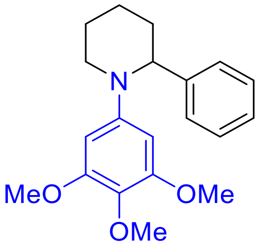

2-Phenyl-1-(3,4,5-trimethoxyphenyl)piperidine (53). Purification by flash chromatography (n-hexane/ethyl acetate = 10:1), yellow oil (104.7 mg, 64%);

^{1}H NMR (400 MHz, CDCl$_3$) δ (ppm): 7.32 – 7.18 (m, 5H), 7.18 – 7.06 (m, 1H), 6.16 (s, 2H), 4.20 (dd, *J* = 8.5, 3.7 Hz, 1H), 3.75 (s, 3H), 3.72 (s, 6H), 3.52 – 3.41 (m, 1H), 3.13 – 3.00 (m, 1H), 2.04 – 1.94 (m, 1H), 1.92 – 1.72 (m, 4H), 1.63 – 1.45 (m, 1H);

^{13}C{^{1}H} NMR (101 MHz, CDCl$_3$) δ (ppm): 153.1, 148.8, 144.3, 132.5, 128.3, 127.2, 126.4, 98.9, 63.5, 60.9, 56.0, 53.9, 34.9, 26.2, 23.3;

HRMS for $C_{20}H_{26}NO_3$ [M+H]$^+$: m/z calcd 328.1908, found 328.1917.

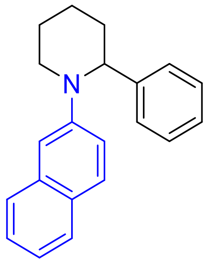

1-(Naphthalen-2-yl)-2-phenylpiperidine (54). Purification by flash chromatography (n-hexane/ethyl acetate = 10:1), yellow oil (110.5 mg, 77%);

^{1}H NMR (400 MHz, CDCl$_3$) δ (ppm): 7.66 – 7.57 (m, 2H), 7.54 (d, *J* = 8.2 Hz, 1H), 7.33 – 7.26 (m, 3H), 7.24 – 7.15 (m, 4H), 7.14 – 7.03 (m, 2H), 4.69 – 4.62 (m, 1H), 3.53 – 3.43 (m, 1H), 3.39 – 3.26 (m, 1H), 2.07 – 1.92 (m, 2H), 1.83 – 1.74 (m, 2H), 1.74 – 1.63 (m, 1H), 1.62 – 1.50 (m, 1H);

^{13}C{^{1}H} NMR (101 MHz, CDCl$_3$) δ (ppm): 149.6, 143.6, 134.6, 128.6, 128.5, 127.5, 127.3, 126.8, 126.4, 126.0, 123.2, 121.0, 113.6, 61.1, 50.5, 33.5, 25.8, 22.1;

HRMS for $C_{21}H_{22}N$ [M+H]$^+$: m/z calcd 288.1747, found 288.1749.

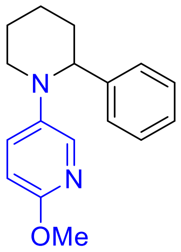

2-Methoxy-5-(2-phenylpiperidin-1-yl)pyridine (55). Purification by flash chromatography (n-hexane/ethyl acetate = 15:1), yellow oil (60.3 mg, 45%);

^{1}H NMR (400 MHz, CDCl$_3$) δ (ppm): 7.82 (d, *J* = 2.8 Hz, 1H), 7.31 – 7.23 (m, 3H), 7.21 – 7.14 (m, 2H), 7.13 – 7.05 (m, 1H), 6.52 (d, *J* = 8.8 Hz, 1H), 3.99 (dd, *J* = 9.9, 3.2 Hz, 1H), 3.82 (s, 3H), 3.42 – 3.28 (m, 1H), 2.94 – 2.79 (m, 1H), 2.00 – 1.67 (m, 5H), 1.62 – 1.43 (m, 1H);

^{13}C{^{1}H} NMR (101 MHz, CDCl$_3$) δ (ppm): 159.7, 144.1, 143.0, 142.0, 134.4, 128.4 127.6, 126.6, 110.3, 64.9, 57.2, 53.4, 36.5, 26.6, 24.4;

HRMS for $C_{17}H_{21}N_2O$ [M+H]$^+$: m/z calcd 269.1649, found 269.1655.

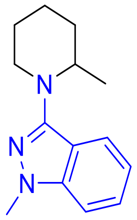

1-Methyl-3-(2-methylpiperidin-1-yl)-1H-indazole (56). Purification by flash chromatography (n-hexane/ethyl acetate = 20:1), yellow oil (44.6 mg, 39%);

^{1}H NMR (400 MHz, CDCl$_3$) δ (ppm): 7.72 (d, *J* = 8.2 Hz, 1H), 7.35 – 7.28 (m, 1H), 7.27 – 7.19 (m, 1H), 7.04 – 6.95 (m, 1H), 4.11 – 4.00 (m, 1H), 3.89 (s, 3H), 3.47 – 3.38 (m, 1H), 3.34 – 3.22 (m, 1H), 2.03 – 1.91 (m, 1H), 1.82 – 1.67 (m, 3H), 1.65 – 1.54 (m, 2H), 1.09 (d, *J* = 6.6 Hz, 3H);

^{13}C{^{1}H} NMR (101 MHz, CDCl$_3$) δ (ppm): 151.8, 141.8, 126.3, 121.6, 118.4, 116.6, 108.8, 52.7, 46.7, 35.1, 32.1, 26.1, 20.7, 15.7;

HRMS for $C_{14}H_{20}N_3$ [M+H]$^+$: m/z calcd 230.1652, found 230.1655.

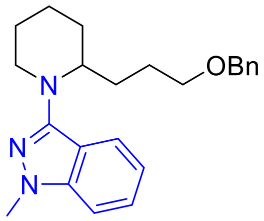

3-(2-(3-(Benzyloxy)propyl)piperidin-1-yl)-1-methyl-1*H*-indazole (57). Purification by flash chromatography (n-hexane/ethyl acetate = 20:1), yellow oil (59.9 mg, 33%);

^{1}H NMR (400 MHz, CDCl$_3$) δ (ppm): 7.74 (d, *J* = 8.2 Hz, 1H), 7.44 – 7.18 (m, 7H), 7.07 – 6.97 (m, 1H), 4.59 (s, 2H), 4.38 – 4.24 (m, 1H), 4.01 – 3.93 (m, 1H), 3.90 (s, 3H), 3.87 – 3.73 (m, 4H), 3.48 – 3.34 (m, 1H), 2.10 – 1.84 (m, 3H), 1.84 – 1.53 (m, 5H);

^{13}C{^{1}H} NMR (101 MHz, CDCl$_3$) δ (ppm): 151.6, 141.9, 138.7, 128.5, 127.8, 127.6, 126.6, 121.4, 118.5, 115.7, 108.9, 73.3, 67.9, 65.6, 62.4, 45.4, 35.1, 34.5, 23.9, 23.7, 20.7;

HRMS for $C_{23}H_{30}N_3O$ [M+H]$^+$: m/z calcd 364.2384, found 364.2388.

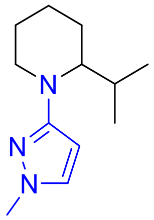

2-Isopropyl-1-(1-methyl-1*H*-pyrazol-3-yl)piperidine (58). Purification by flash chromatography (n-hexane/ethyl acetate = 20:1), yellow oil (61.1 mg, 59%);

^{1}H NMR (400 MHz, CDCl$_3$) δ (ppm): 7.08 (d, *J* = 2.3 Hz, 1H), 5.50 (d, *J* = 2.3 Hz, 1H), 3.70 (s, 3H), 3.65 – 3.54 (m, 1H), 3.28 – 3.17 (m, 1H), 3.09 – 2.97 (m, 1H), 2.29 – 2.14 (m, 1H), 1.76 – 1.39 (m, 6H), 0.92 (d, *J* = 6.6 Hz, 3H), 0.88 (d, *J* = 6.7 Hz, 3H);

$^{13}C\{^1H\}$ NMR (101 MHz, $CDCl_3$) δ (ppm): 160.1, 131.0, 90.6, 61.1, 42.7, 38.7, 26.5, 24.7, 24.2, 20.4, 20.4, 20.2;

HRMS for $C_{12}H_{22}N_3$ $[M+H]^+$: m/z calcd 208.1809, found 208.1813.

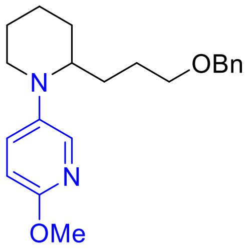

3-(2-(3-(Benzyloxy)propyl)piperidin-1-yl)-1-methyl-1*H*-indazole (59). Purification by flash chromatography (n-hexane/ethyl acetate = 20:1), yellow oil (64.6 mg, 38%);

^1H NMR (400 MHz, $CDCl_3$) δ (ppm): 7.90 (d, J = 3.0 Hz, 1H), 7.39 – 7.26 (m, 6H), 6.65 (d, J = 8.9 Hz, 1H), 4.52 (s, 2H), 4.13 – 4.01 (m, 1H), 3.88 (s, 3H), 3.77 – 3.59 (m, 2H), 3.33 – 3.11 (m, 4H), 1.99 – 1.88 (m, 1H), 1.85 – 1.61 (m, 4H), 1.60 – 1.46 (m, 3H);

$^{13}C\{^1H\}$ NMR (101 MHz, $CDCl_3$) δ (ppm): 158.8, 142.5, 138.4, 137.1, 131.4, 128.5, 127.8, 127.7, 110.9, 73.3, 68.4, 66.6, 63.5, 53.5, 47.1, 33.2, 23.4, 23.3, 20.9;

HRMS for $C_{21}H_{29}N_2O_2$ $[M+H]^+$: m/z calcd 341.2224, found 341.2224.

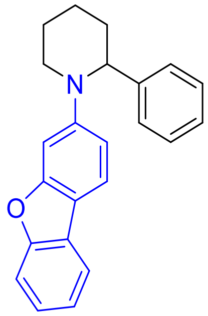

1-(Dibenzo[b,d]furan-3-yl)-2-phenylpiperidine (60). Purification by flash chromatography (n-hexane/ethyl acetate = 20:1), yellow oil (103.1mg, 63%);

^1H NMR (400 MHz, $CDCl_3$) δ (ppm): 7.67 (dd, J = 7.4, 1.5 Hz, 1H), 7.58 (d, J = 8.5 Hz, 1H), 7.35 (d, J = 8.0 Hz, 1H), 7.26 – 7.18 (m, 3H), 7.18 – 7.10 (m, 3H), 7.07 – 7.00 (m, 1H), 6.96 (d, J = 2.1 Hz, 1H), 6.85 (dd, J = 8.5, 2.1 Hz, 1H), 4.57 – 4.50 (m, 1H), 3.46 – 3.35 (m, 1H), 3.34 – 3.20 (m, 1H), 2.04 – 1.84 (m, 2H), 1.81 – 1.46 (m, 4H);

$^{13}C\{^1H\}$ NMR (101 MHz, $CDCl_3$) δ (ppm): 157.8, 156.2, 152.3, 143.3, 128.5, 127.3, 126.5, 125.4, 124.9, 122.6, 120.6, 119.6, 116.4, 114.7, 111.2, 101.2, 61.5, 50.7, 33.5, 25.7, 21.9;

HRMS for $C_{23}H_{22}NO$ $[M+H]^+$: m/z calcd 328.1696, found 328.1691.

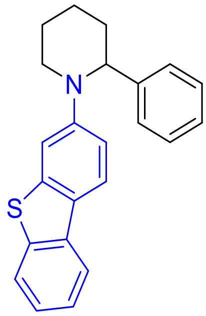

1-(Dibenzo[b,d]thiophen-3-yl)-2-phenylpiperidine (61). Purification by flash chromatography (n-hexane/ethyl acetate = 20:1), yellow oil (120.1 mg, 70%);

^{1}H NMR (400 MHz, $CDCl_3$) δ (ppm): 7.73 (dd, *J* = 7.5, 1.5 Hz, 1H), 7.64 (d, *J* = 8.5 Hz, 1H), 7.42 (d, *J* = 7.6 Hz, 1H), 7.31 – 7.24 (m, 3H), 7.24 – 7.18 (m, 3H), 7.14 – 7.07 (m, 1H), 7.03 (d, *J* = 2.1 Hz, 1H), 6.92 (dd, *J* = 8.5, 2.2 Hz, 1H), 4.64 – 4.57 (m, 1H), 3.54 – 3.41 (m, 1H), 3.40 – 3.28 (m, 1H), 2.09 – 1.92 (m, 2H), 1.87 – 1.73 (m, 2H), 1.73 – 1.62 (m, 1H), 1.62 – 1.49 (m, 1H);

^{13}C{^{1}H} NMR (101 MHz, $CDCl_3$) δ (ppm): 157.8, 156.2, 152.3, 143.3, 128.5, 127.3, 126.5, 125.4, 124.8, 122.6, 120.6, 119.6, 116.3, 114.7, 111.2, 101.2, 61.5, 50.7, 33.4, 25.7, 21.8;

HRMS for $C_{23}H_{22}NS$ $[M+H]^+$: m/z calcd 344.1468, found 344.1470.

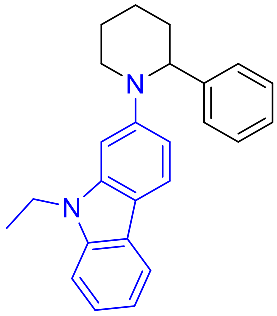

9-Ethyl-2-(2-phenylpiperidin-1-yl)-9H-carbazole (62). Purification by flash chromatography (n-hexane/ethyl acetate = 20:1), yellow oil (134.5mg, 76%);

^{1}H NMR (400 MHz, $CDCl_3$) δ (ppm): 7.86 (d, *J* = 7.7 Hz, 1H), 7.66 – 7.58 (m, 1H), 7.30 – 7.19 (m, 3H), 7.13 (d, *J* = 8.2 Hz, 1H), 7.10 – 6.95 (m, 5H), 6.91 – 6.83 (m, 1H), 4.08 – 3.98 (m, 3H), 3.43 – 3.35 (m, 1H), 2.90 – 2.79 (m, 1H), 1.91 – 1.63 (m, 5H), 1.53 – 1.38 (m, 1H), 1.18 (t, *J* = 7.2 Hz, 3H).

^{13}C{^{1}H} NMR (101 MHz, $CDCl_3$) δ (ppm): 145.6, 145.1, 140.3, 136.5, 128.1, 127.7, 126.2, 125.3, 122.9, 122.9, 122.8, 120.3, 118.3, 115.3, 108.4, 108.3, 65.7, 58.3, 37.5, 36.9, 26.9, 24.8, 13.9;

HRMS for $C_{25}H_{27}N_2$ $[M+H]^+$: m/z calcd 355.2169, found 355.2173.

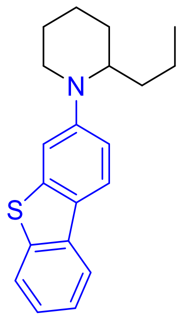

1-(Dibenzo[b,d]thiophen-3-yl)-2-propylpiperidine (63). Purification by flash chromatography (n-hexane/ethyl acetate = 20:1), yellow oil (108.2 mg, 70%);

^{1}H NMR (400 MHz, CDCl$_3$) δ (ppm): 7.91 – 7.86 (m, 2H), 7.68 (d, *J* = 7.9 Hz, 1H), 7.32 – 7.28 (m, 1H), 7.26 – 7.22 (m, 1H), 7.20 – 7.14 (m, 1H), 6.99 (d, *J* = 8.8 Hz, 1H), 3.93 – 3.77 (m, 1H), 3.37 (d, *J* = 12.3 Hz, 1H), 3.11 – 2.91 (m, 1H), 1.81 – 1.06 (m, 10H), 0.79 (t, *J* = 7.3 Hz, 3H).

^{13}C{^{1}H} NMR (101 MHz, CDCl$_3$) δ (ppm): 151.0, 141.5, 138.4, 136.2, 126.9, 124.9, 124.4, 122.7, 122.0, 120.4, 114.9, 108.6, 56.3, 43.9, 29.9, 27.9, 25.7, 20.4, 19.3, 14.3;

HRMS for $C_{20}H_{24}NS$ [M+H]$^+$: m/z calcd 310.1624, found 310.1627.

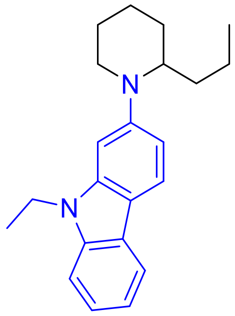

9-Ethyl-2-(2-propylpiperidin-1-yl)-9H-carbazole (64). Purification by flash chromatography (n-hexane/ethyl acetate = 20:1), yellow oil (113.6 mg, 71%);

^{1}H NMR (400 MHz, CDCl$_3$) δ (ppm): 7.96 (d, *J* = 7.7 Hz, 1H), 7.67 (s, 1H), 7.40 – 7.29 (m, 1H), 7.26 (d, *J* = 8.2 Hz, 1H), 7.23 – 7.14 (m, 2H), 7.14 – 7.05 (m, 1H), 4.21 (q, *J* = 7.2 Hz, 2H), 3.35 – 3.24 (m, 1H), 3.15 – 3.03 (m, 1H), 3.03 – 2.92 (m, 1H), 1.89 – 1.80 (m, 1H), 1.78 – 1.37 (m, 5H), 1.31 (t, *J* = 7.2 Hz, 3H), 1.29 – 1.12 (m, 3H), 1.11 – 0.95 (m, 1H), 0.67 (t, *J* = 7.1 Hz, 3H);

^{13}C{^{1}H} NMR (101 MHz, CDCl$_3$) δ (ppm): 140.5, 136.1, 125.5, 123.3, 123.0, 120.9, 120.4, 118.4, 112.6, 108.7, 108.5, 59.9, 51.4, 37.7, 31.9, 29.8, 29.8, 26.4, 21.8, 19.7, 14.4, 14.0;

HRMS for $C_{22}H_{29}N_2$ [M+H]$^+$: m/z calcd 321.2326, found 321.2325.

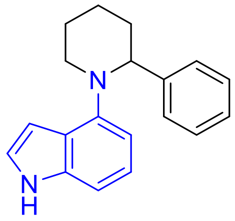

4-(2-Phenylpiperidin-1-yl)-1*H*-indole (65). Purification by flash chromatography (n-hexane/ethyl acetate = 20:1), yellow oil (80.1mg, 58%);

^{1}H NMR (400 MHz, CDCl$_3$) δ (ppm): 7.98 (s, 1H), 7.39 – 7.29 (m, 2H), 7.18 – 7.07 (m, 3H), 7.07 – 6.99 (m, 1H), 6.95 – 6.82 (m, 2H), 6.71 – 6.69 (m, 1H), 6.53 (dd, *J* = 7.1, 1.4 Hz, 1H), 4.35 (dd, *J* = 9.6, 3.3 Hz, 1H), 3.82 – 3.67 (m, 1H), 2.93 – 2.78 (m, 1H), 2.07 – 1.97 (m, 1H), 1.96 – 1.72 (m, 4H), 1.68 – 1.53 (m, 1H);

^{13}C{^{1}H} NMR (101 MHz, CDCl$_3$) δ (ppm): 145.9, 145.4, 136.9, 128.1, 127.0, 126.1, 124.0, 122.7, 122.2, 111.9, 105.9, 101.4, 63.9, 55.2, 36.8, 26.7, 24.8;

HRMS for $C_{19}H_{21}N_2$ [M+H]$^+$: m/z calcd 277.1700, found 277.1699.

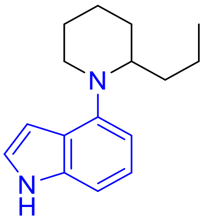

4-(2-Propylpiperidin-1-yl)-1*H*-indole (66). Purification by flash chromatography (n-hexane/ethyl acetate = 20:1), yellow oil (61.7 mg, 51%);

^{1}H NMR (400 MHz, CDCl$_3$) δ (ppm): 8.09 (s, 1H), 7.13 – 7.06 (m, 2H), 7.01 (d, *J* = 8.0 Hz, 1H), 6.66 – 6.53 (m, 2H), 3.85 – 3.71 (m, 1H), 3.39 – 3.25 (m, 1H), 3.19 – 3.02 (m, 1H), 2.02 – 1.93 (m, 1H), 1.75 – 1.51 (m, 6H), 1.45 – 1.34 (m, 1H), 1.17 – 1.03 (m, 1H), 0.94 – 0.81 (m, 1H), 0.75 (t, *J* = 7.3 Hz, 3H);

^{13}C{^{1}H} NMR (101 MHz, CDCl$_3$) δ (ppm): 146.2, 137.2, 122.6, 122.3, 109.5, 104.9, 101.9, 57.6, 47.9, 30.5, 29.9, 28.9, 26.5, 20.8, 20.1, 14.4;

HRMS for $C_{16}H_{23}N_2$ [M+H]$^+$: m/z calcd 243.1856, found 243.1859.

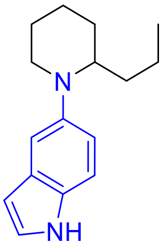

5-(2-Propylpiperidin-1-yl)-1*H*-indole (67). Purification by flash chromatography (n-hexane/ethyl acetate = 20:1), yellow oil (95.6 mg, 79%);

^{1}H NMR (400 MHz, CDCl$_3$) δ (ppm): 8.18 (s, 1H), 7.20 – 7.10 (m, 2H), 7.01 (d, *J* = 3.2 Hz, 1H), 6.92 (d, *J* = 8.8 Hz, 1H), 6.36 (d, *J* = 3.2 Hz, 1H), 3.31 – 3.20 (m, 1H), 3.09 – 2.99 (m, 1H), 2.97 – 2.85 (m, 1H), 1.88 – 1.76 (m, 1H), 1.71 – 1.57 (m, 3H), 1.55 – 1.47 (m, 1H), 1.46 – 1.37 (m, 1H), 1.31 – 1.16 (m, 3H), 1.08 – 0.97 (m, 1H), 0.67 (t, *J* = 7.3 Hz, 3H);

^{13}C{^{1}H} NMR (101 MHz, CDCl$_3$) δ (ppm): 145.9, 131.9, 128.4, 124.6, 118.0, 111.9, 111.4, 102.3, 59.5, 50.9, 31.5, 29.7, 26.5, 21.7, 19.8, 14.4;

HRMS for $C_{16}H_{23}N_2$ $[M+H]^+$: m/z calcd 243.1856, found 243.1854.

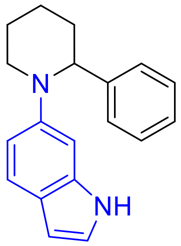

5-(2-Propylpiperidin-1-yl)-1*H*-indole (68). Purification by flash chromatography (n-hexane/ethyl acetate = 20:1), yellow oil (111.8 mg, 81%);

^{1}H NMR (400 MHz, $CDCl_3$) δ (ppm): 7.85 (s, 1H), 7.40 (d, J = 8.8 Hz, 1H), 7.32 (d, J = 7.6 Hz, 2H), 7.17 – 7.13 (m, 2H), 7.05 (d, J = 8.3 Hz, 1H), 7.00 – 6.98 (m, 1H), 6.96 – 6.86 (m, 2H), 6.38 – 6.36 (m, 1H), 4.24 (dd, J = 9.0, 3.6 Hz, 1H), 3.58 – 3.43 (m, 1H), 3.10 – 2.92 (m, 1H), 2.05 – 1.94 (m, 1H), 1.93 – 1.72 (m, 4H), 1.65 – 1.51 (m, 1H);

^{13}C{^{1}H} NMR (101 MHz, $CDCl_3$) δ (ppm): 144.8, 136.5, 128.2, 127.5, 126.2, 123.2, 120.6, 116.6, 104.1, 102.3, 64.1, 56.1, 35.9, 29.9, 26.6, 24.0;

HRMS for $C_{19}H_{21}N_2$ $[M+H]^+$: m/z calcd 277.1700, found 277.1703.

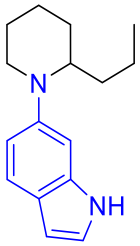

6-(2-Propylpiperidin-1-yl)-1*H*-indole (69). Purification by flash chromatography (n-hexane/ethyl acetate = 20:1), yellow oil (92.1 mg, 76%);

^{1}H NMR (400 MHz, $CDCl_3$) δ (ppm): 8.13 (s, 1H), 7.40 (d, J = 8.6 Hz, 1H), 6.95 – 6.88 (m, 1H), 6.82 (d, J = 8.6 Hz, 1H), 6.73 (s, 1H), 6.34 (s, 1H), 3.50 – 3.38 (m, 1H), 3.03 – 2.88 (m, 2H), 1.83 – 1.71 (m, 1H), 1.68 – 1.51 (m, 4H), 1.49 – 1.41 (m, 1H), 1.39 – 1.13 (m, 3H), 1.10 – 0.97 (m, 1H), 0.69 (t, J = 7.3 Hz, 3H).

^{13}C{^{1}H} NMR (101 MHz, $CDCl_3$) δ (ppm): 147.9, 137.0, 122.9, 122.2, 120.8, 114.3, 102.2, 101.2, 58.4, 47.9, 30.4, 28.9, 26.1, 20.6, 20.1, 14.3;

HRMS for $C_{16}H_{23}N_2$ $[M+H]^+$: m/z calcd 243.1856, found 243.1850.

7. NMR spectra of isolated products

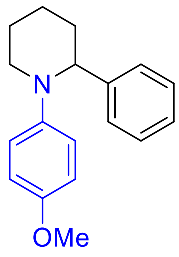

1

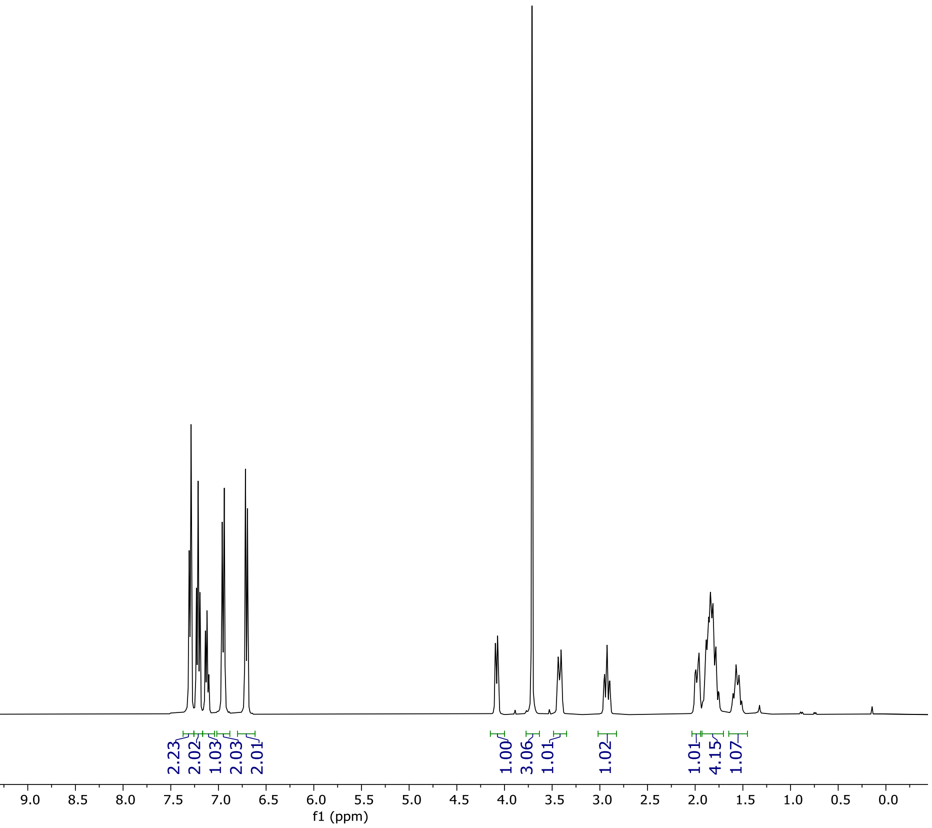

^{1}H NMR (400 MHz, $CDCl_3$) spectrum of compound **1**

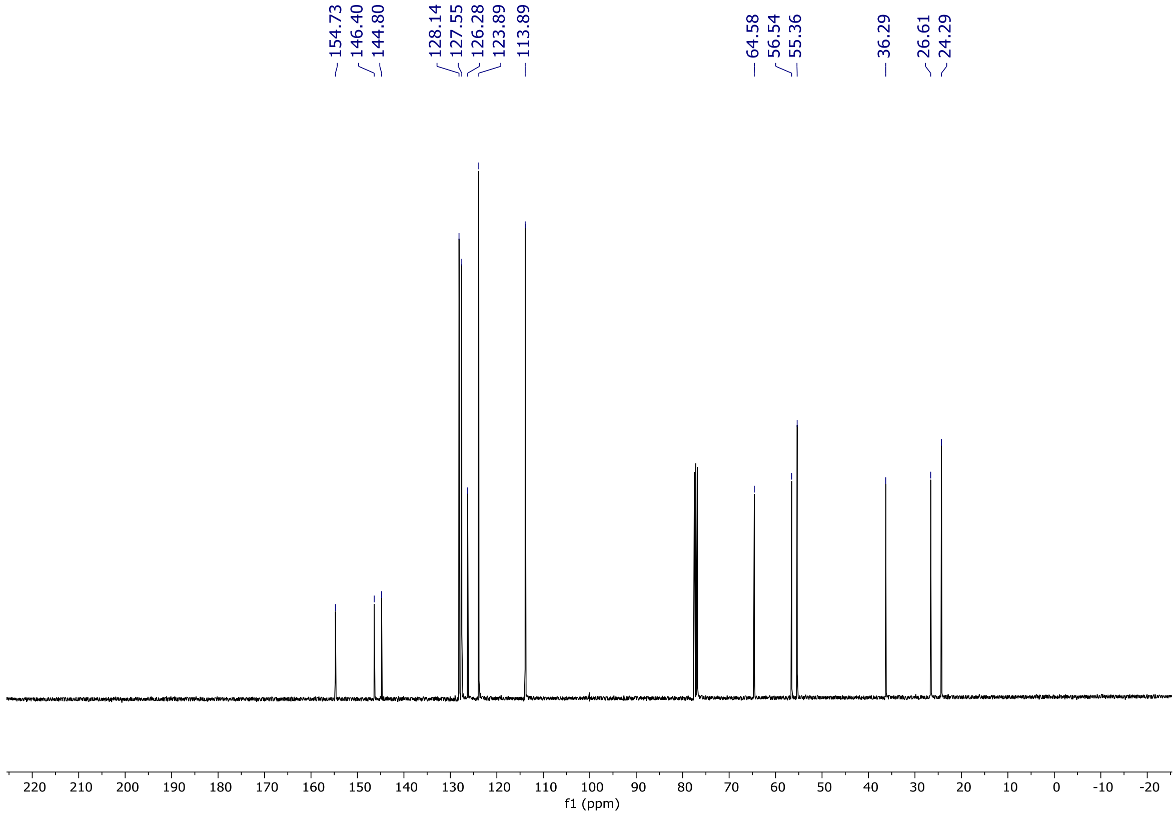

^{13}C{^{1}H} NMR (101 MHz, $CDCl_3$) spectrum of compound **1**

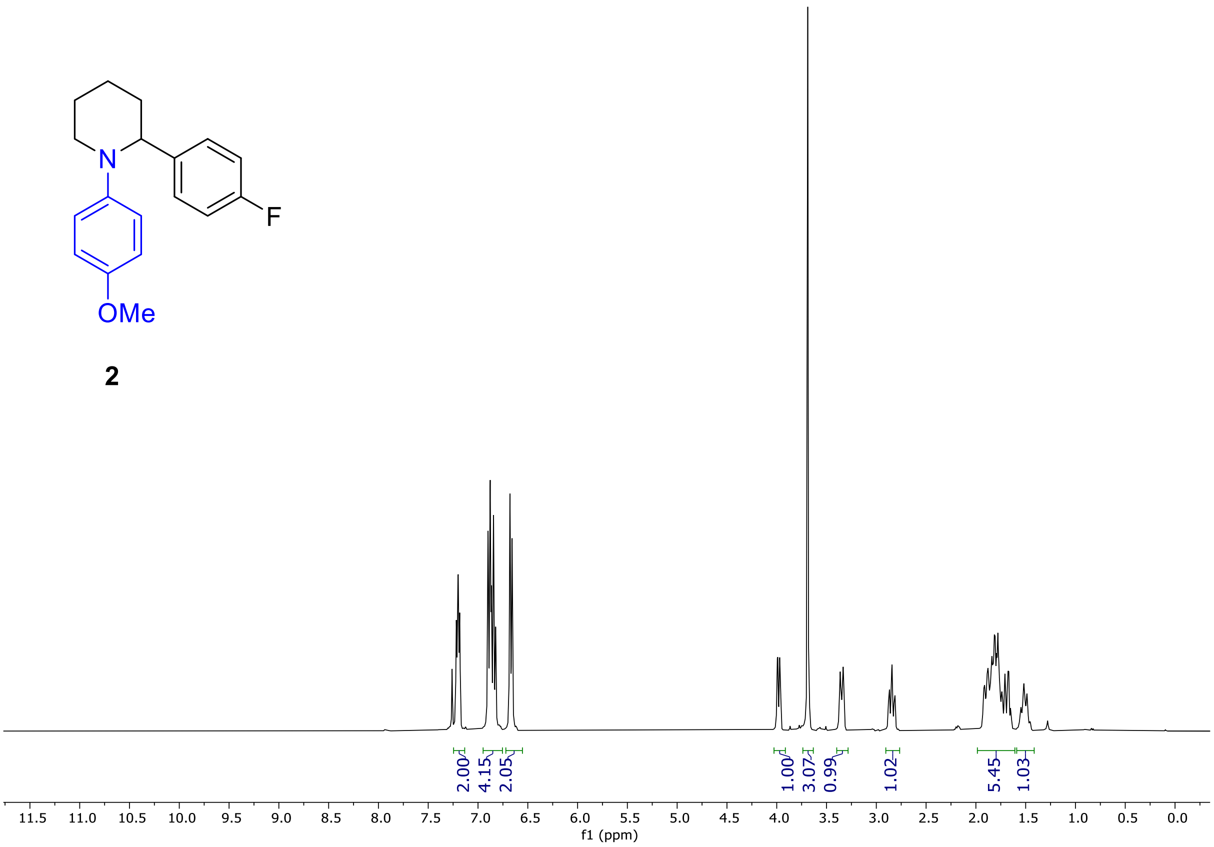

^{1}H NMR (400 MHz, $CDCl_3$) spectrum of compound **2**

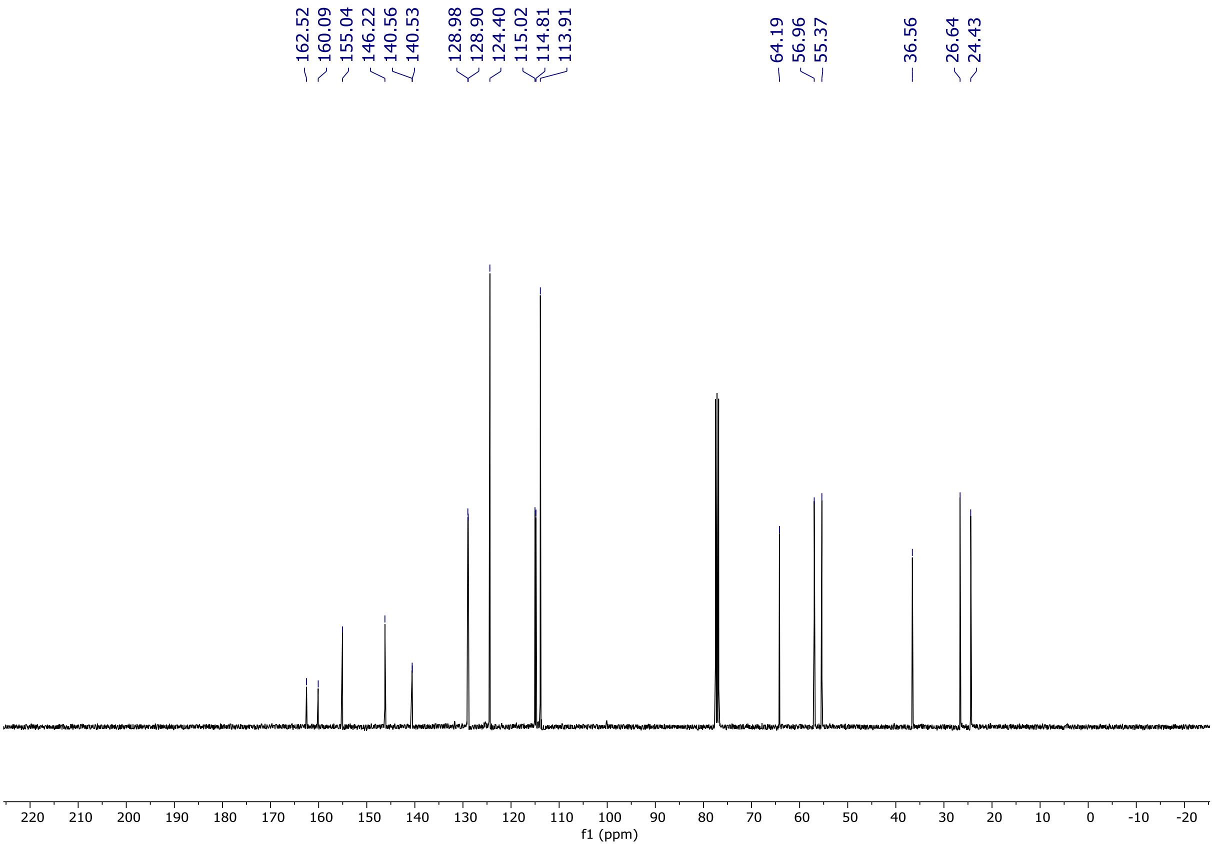

^{13}C{^{1}H} NMR (101 MHz, $CDCl_3$) spectrum of compound **2**

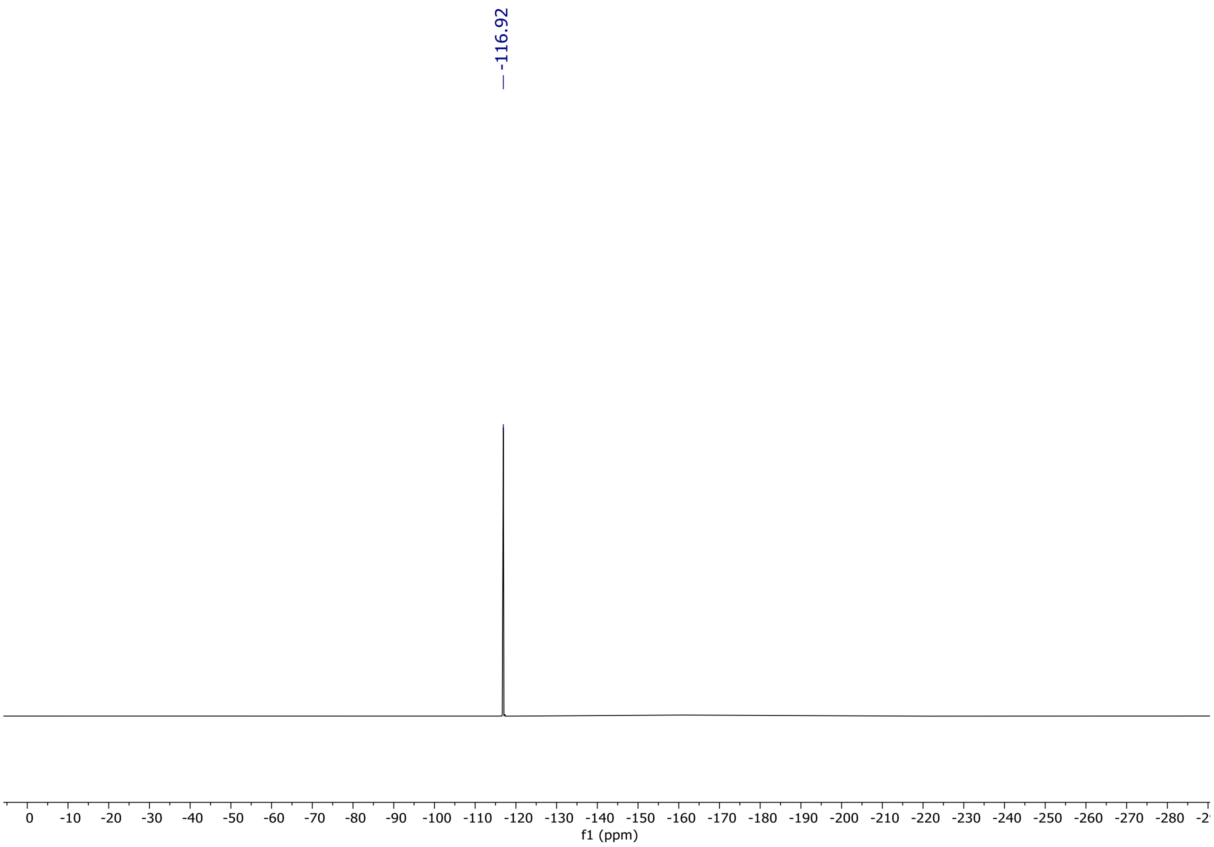

$^{19}F\{^1H\}$ NMR (376 MHz, $CDCl_3$) spectrum of compound **2**

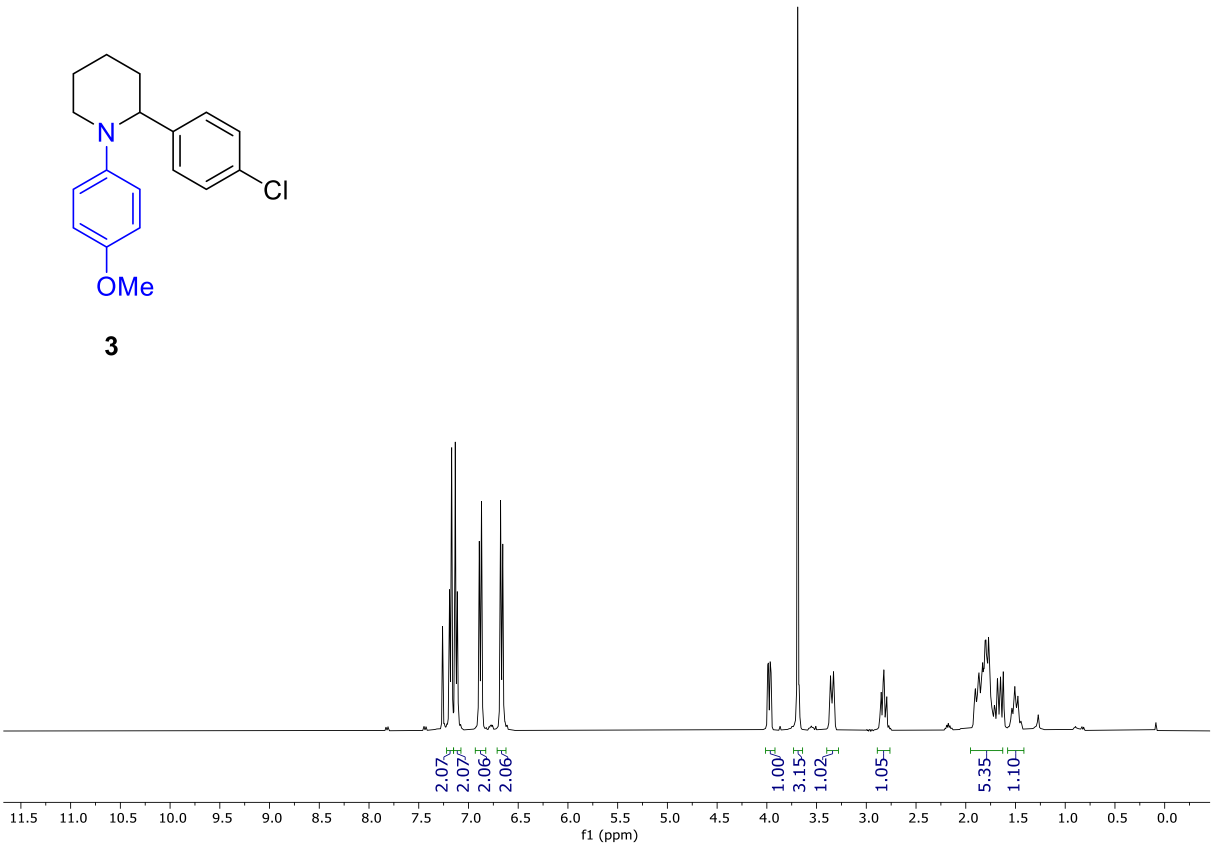

^{1}H NMR (400 MHz, CDCl$_3$) spectrum of compound **3**

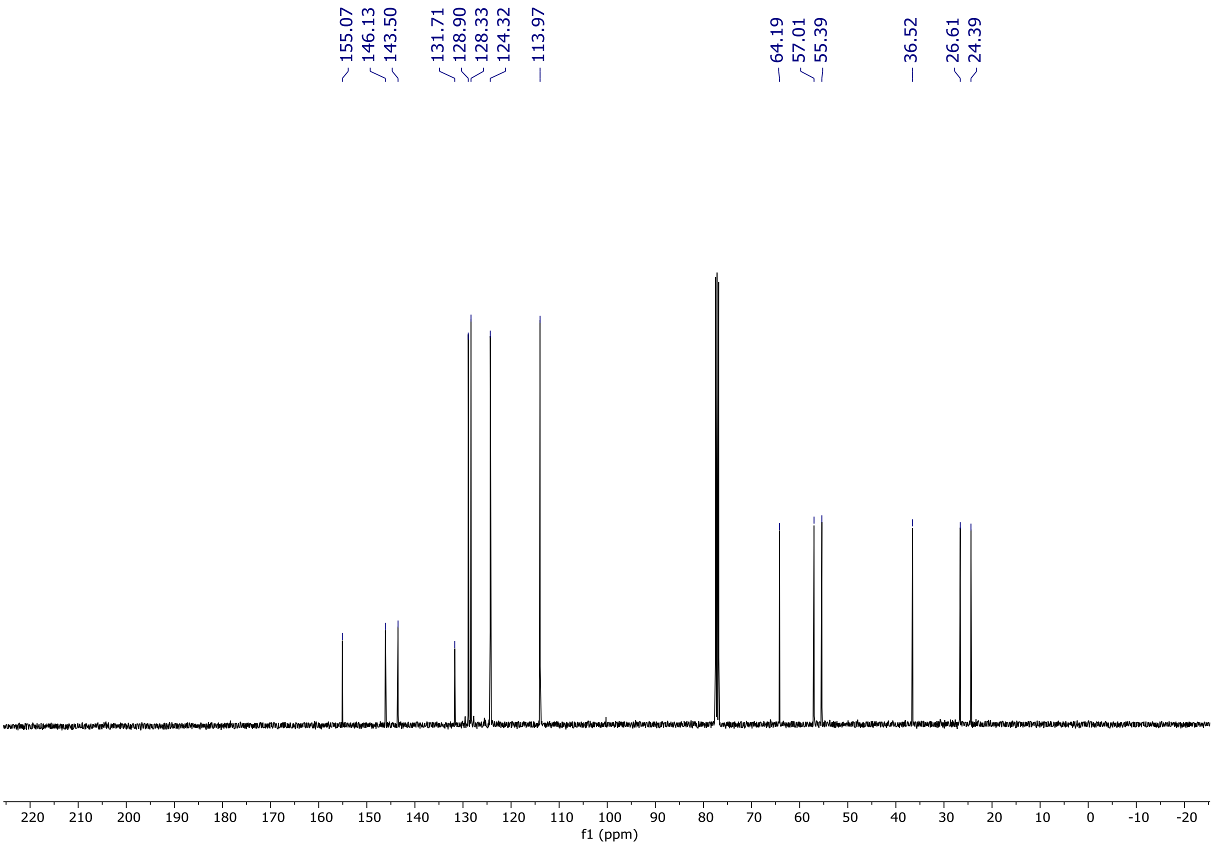

^{13}C{^{1}H} NMR (101 MHz, CDCl$_3$) spectrum of compound **3**

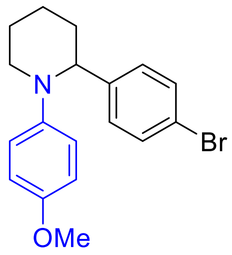

4

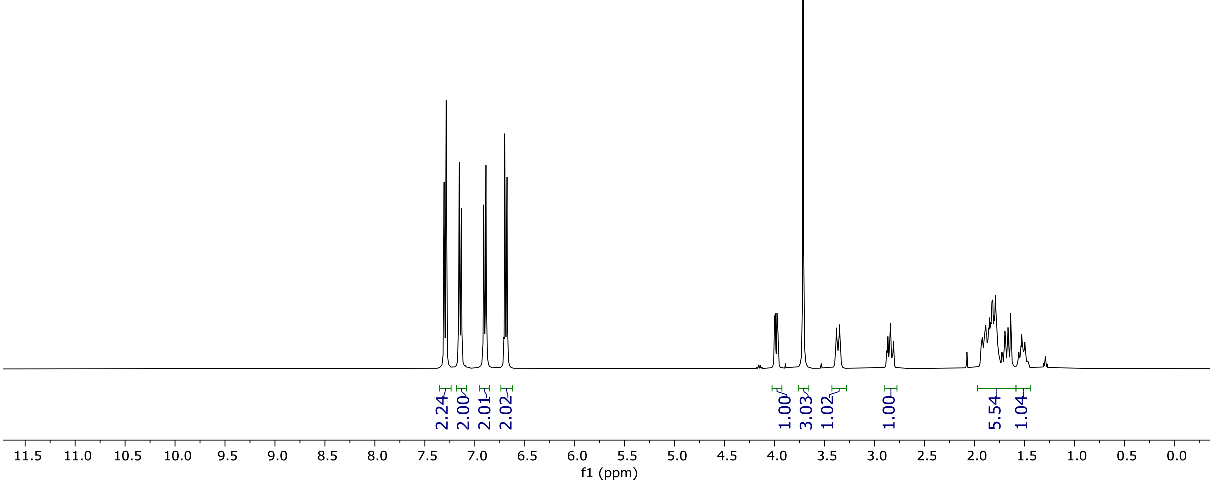

^{1}H NMR (400 MHz, $CDCl_3$) spectrum of compound **4**

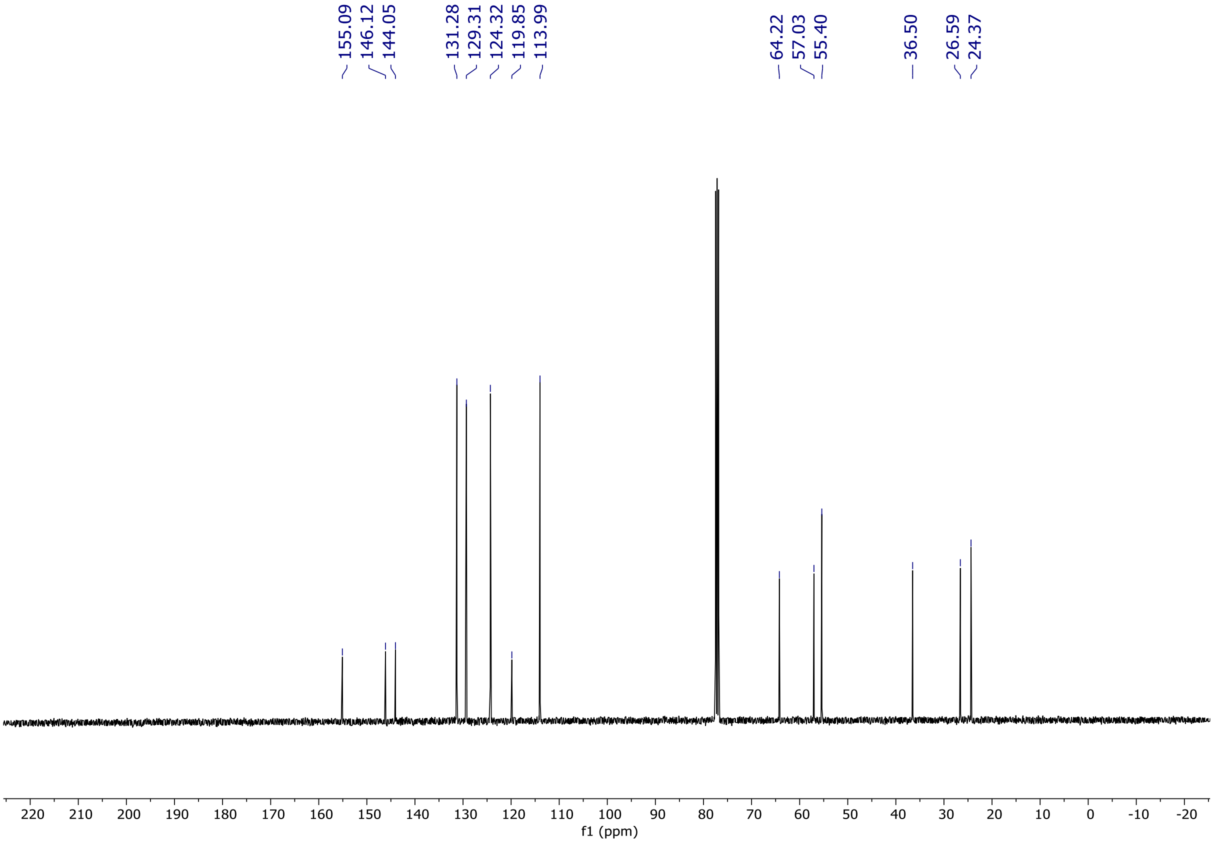

$^{13}C\{^{1}H\}$ NMR (101 MHz, $CDCl_3$) spectrum of compound **4**

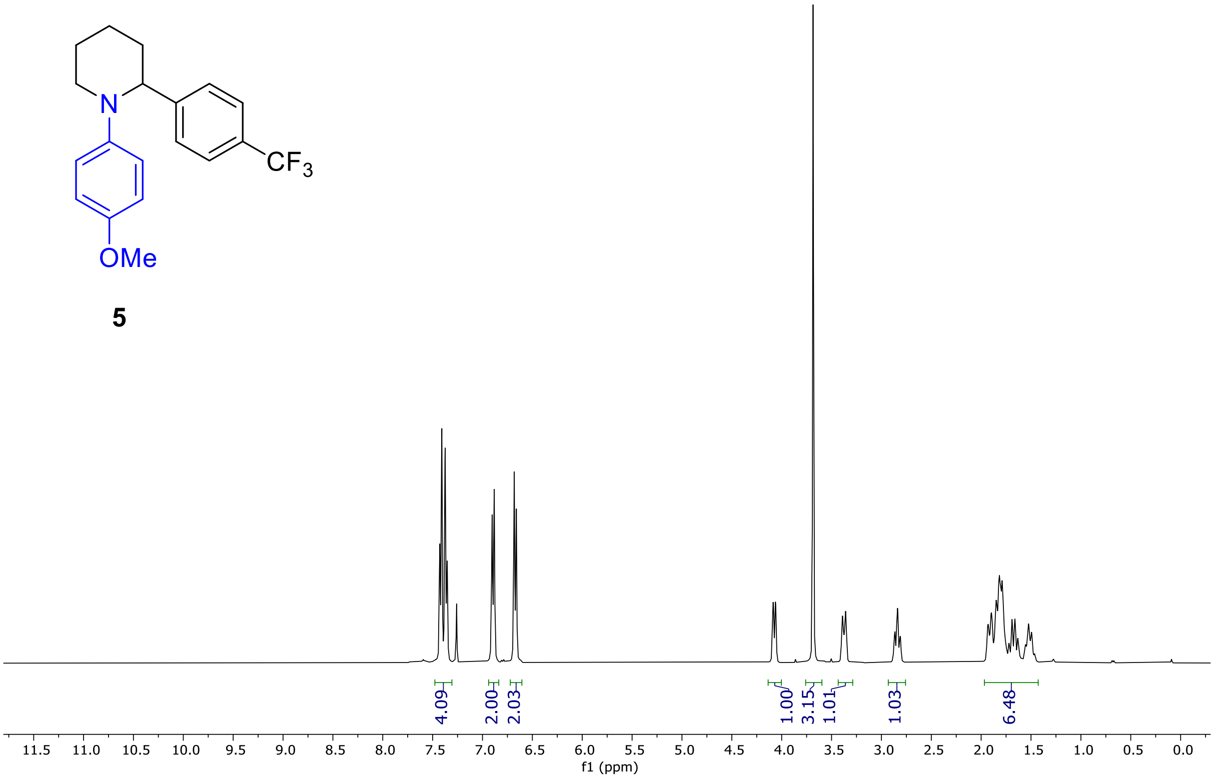

^{1}H NMR (400 MHz, $CDCl_3$) spectrum of compound **5**

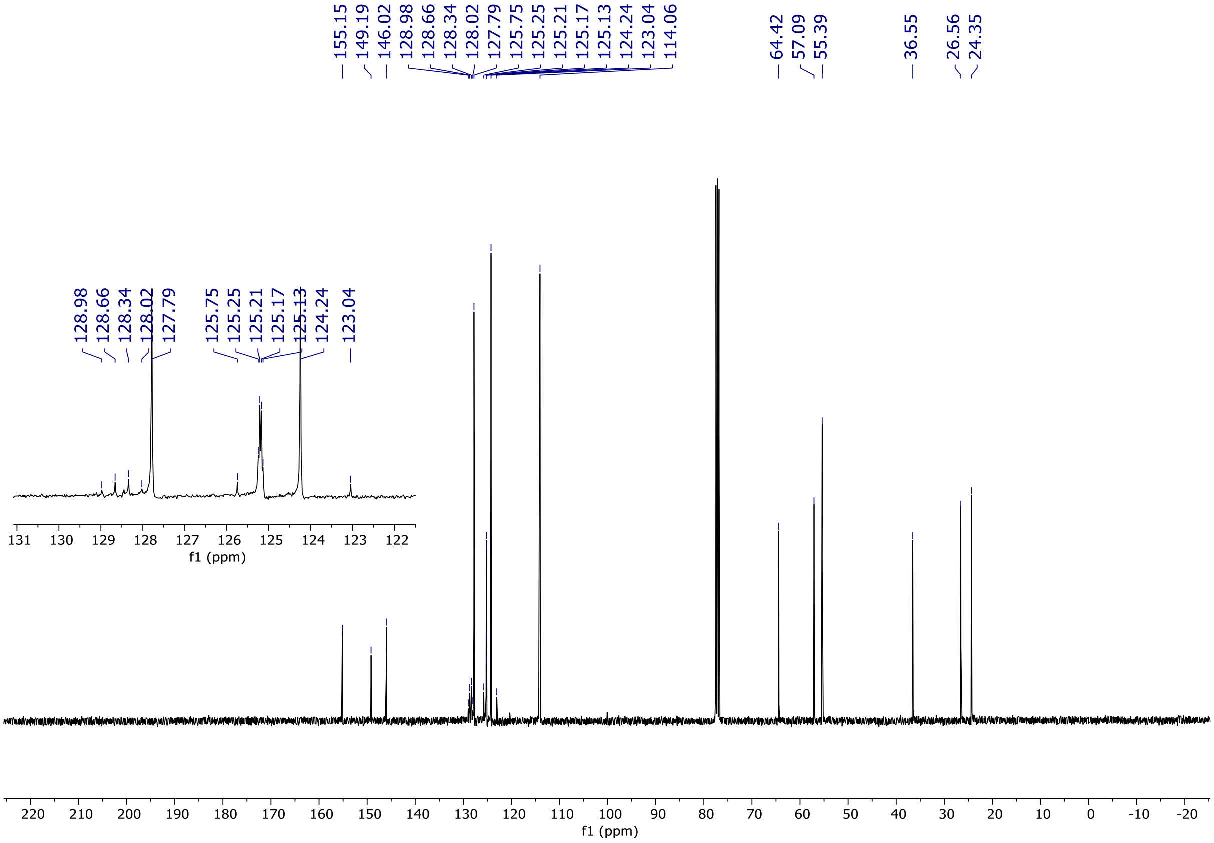

$^{13}C\{^{1}H\}$ NMR (101 MHz, $CDCl_3$) spectrum of compound **5**

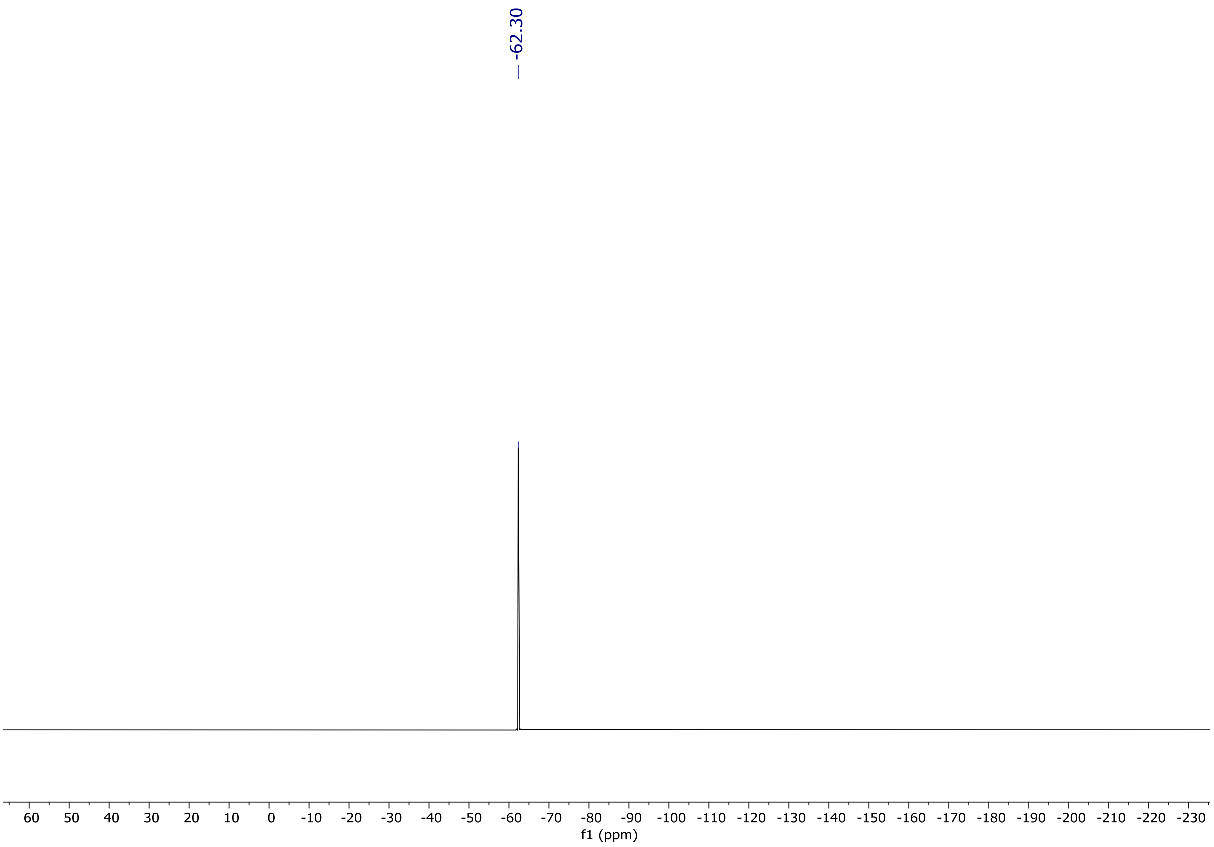

$^{19}F\{^{1}H\}$ NMR (376 MHz, $CDCl_3$) spectrum of compound **5**

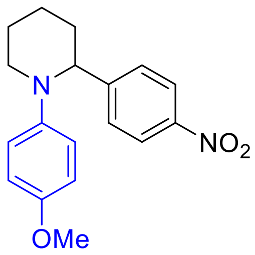

6

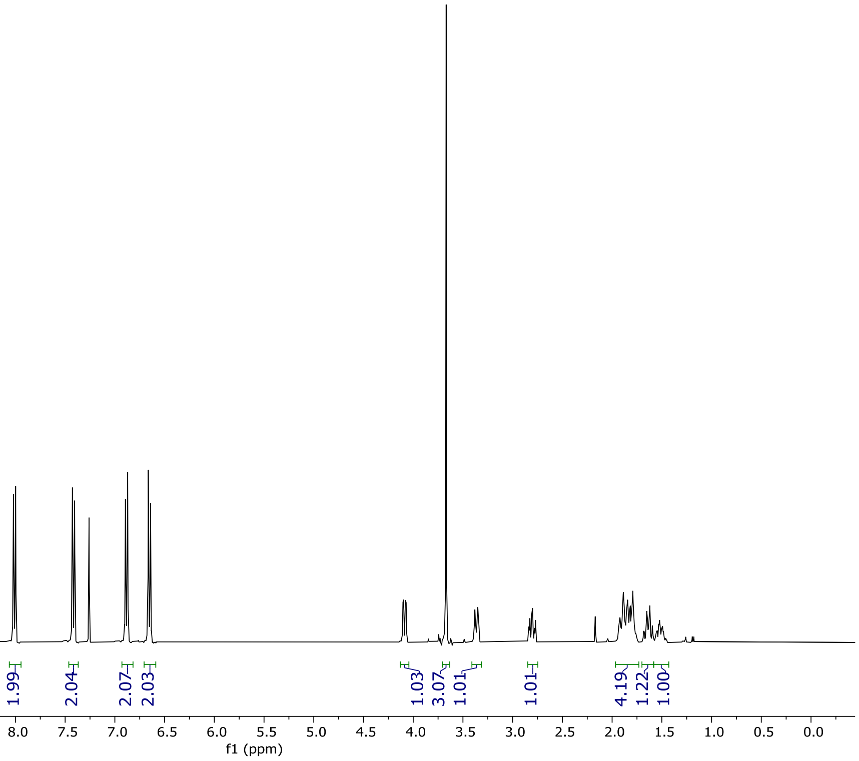

^{1}H NMR (400 MHz, $CDCl_3$) spectrum of compound **6**

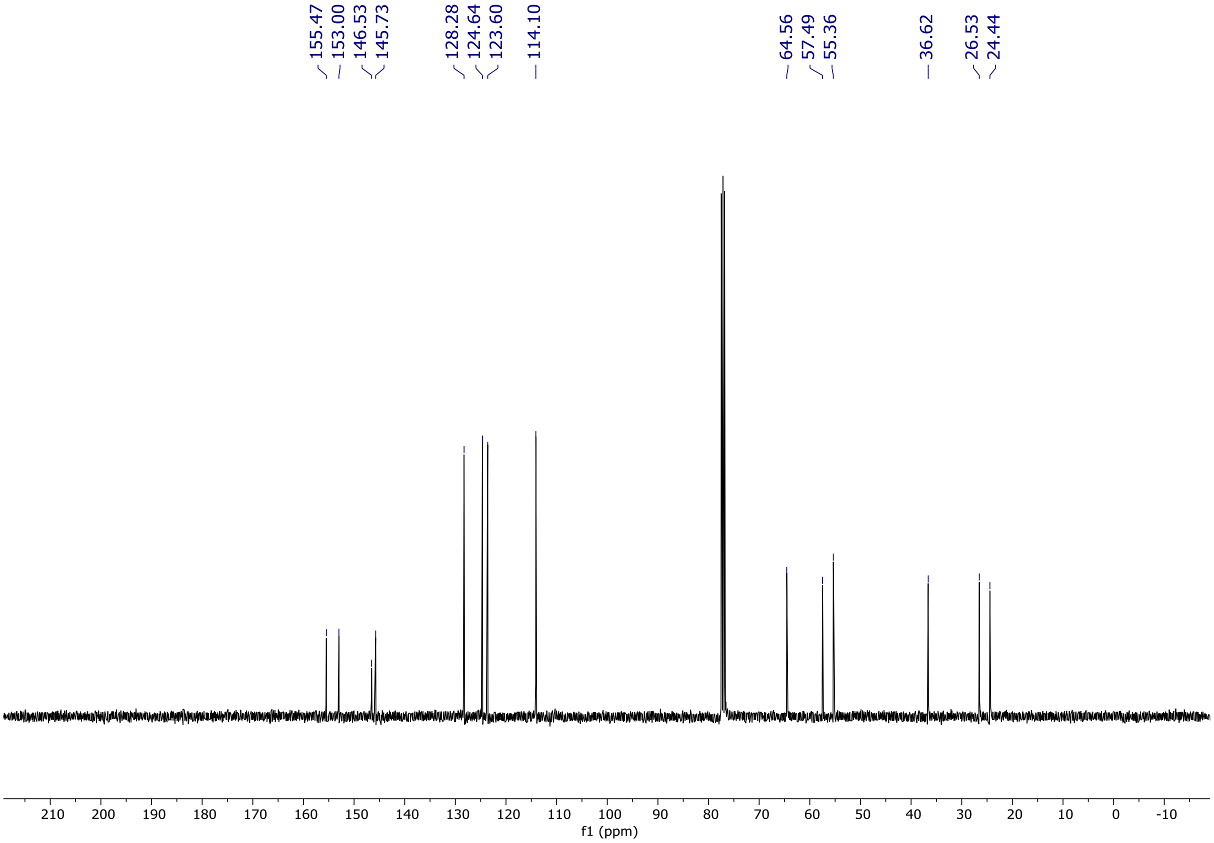

^{13}C{^{1}H} NMR (101 MHz, $CDCl_3$) spectrum of compound **6**

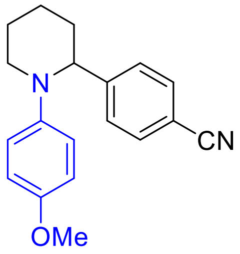

7

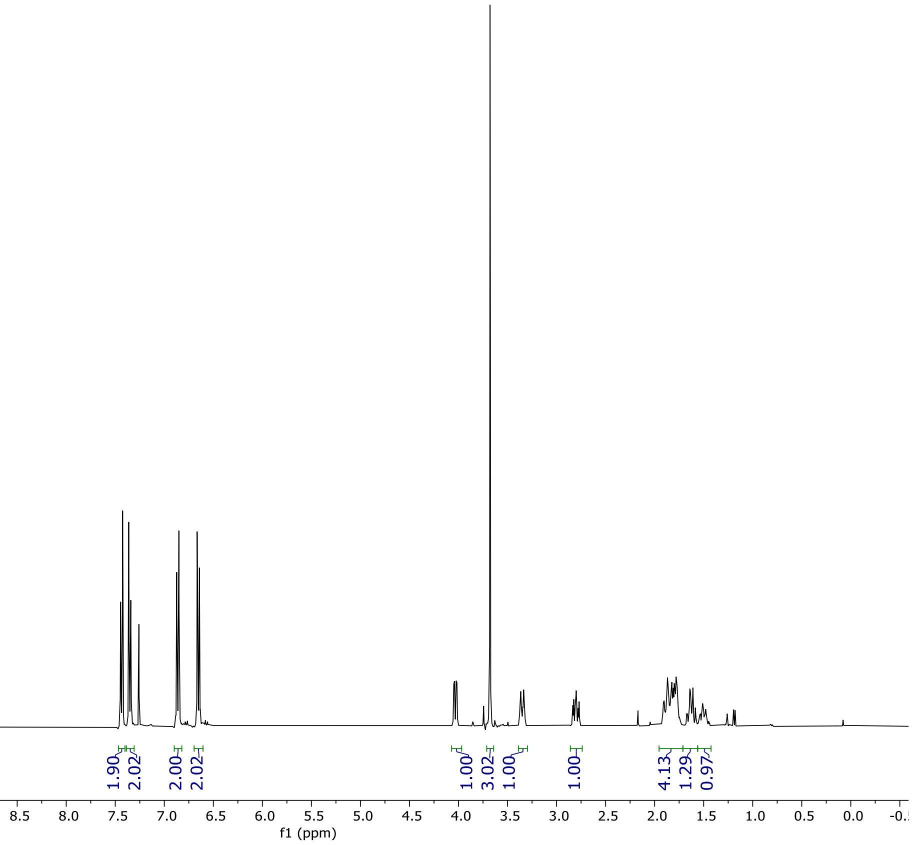

^{1}H NMR (400 MHz, $CDCl_3$) spectrum of compound **7**

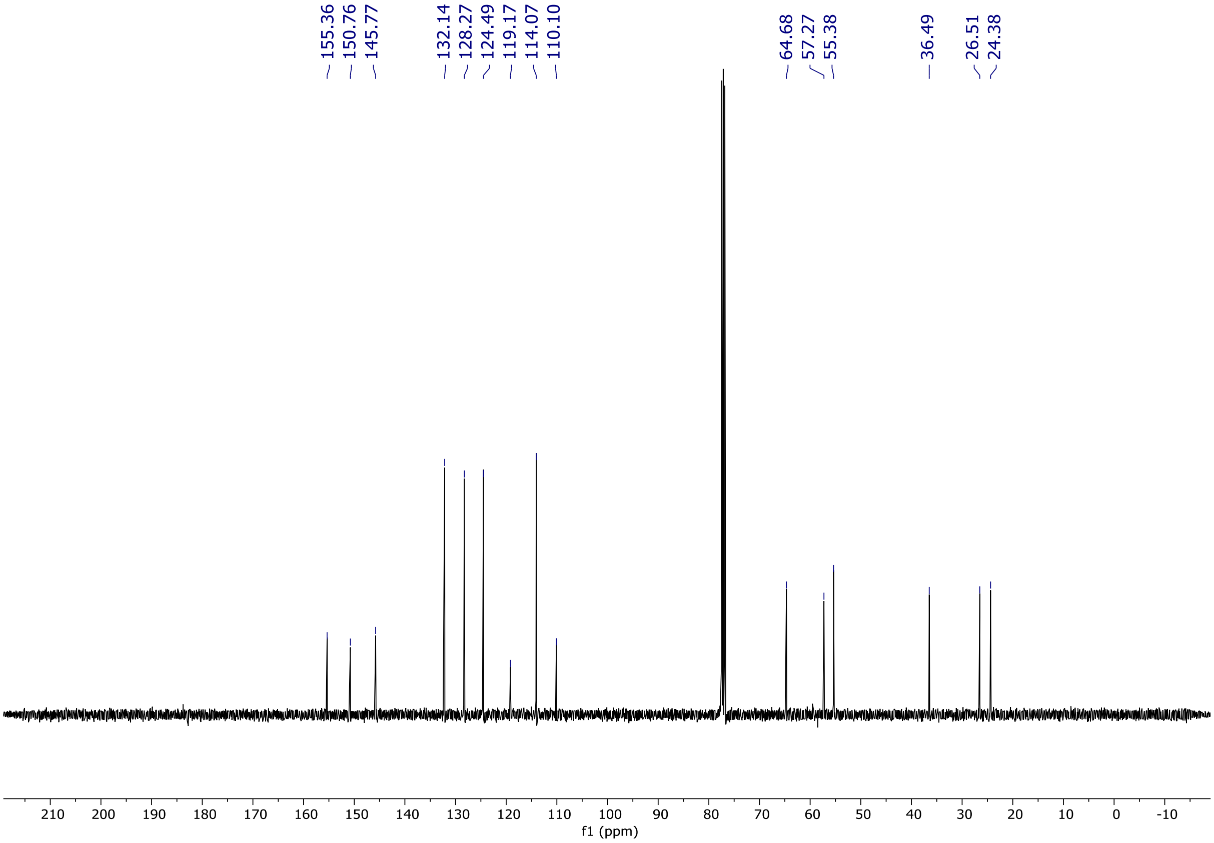

^{13}C{^{1}H} NMR (101 MHz, $CDCl_3$) spectrum of compound **7**

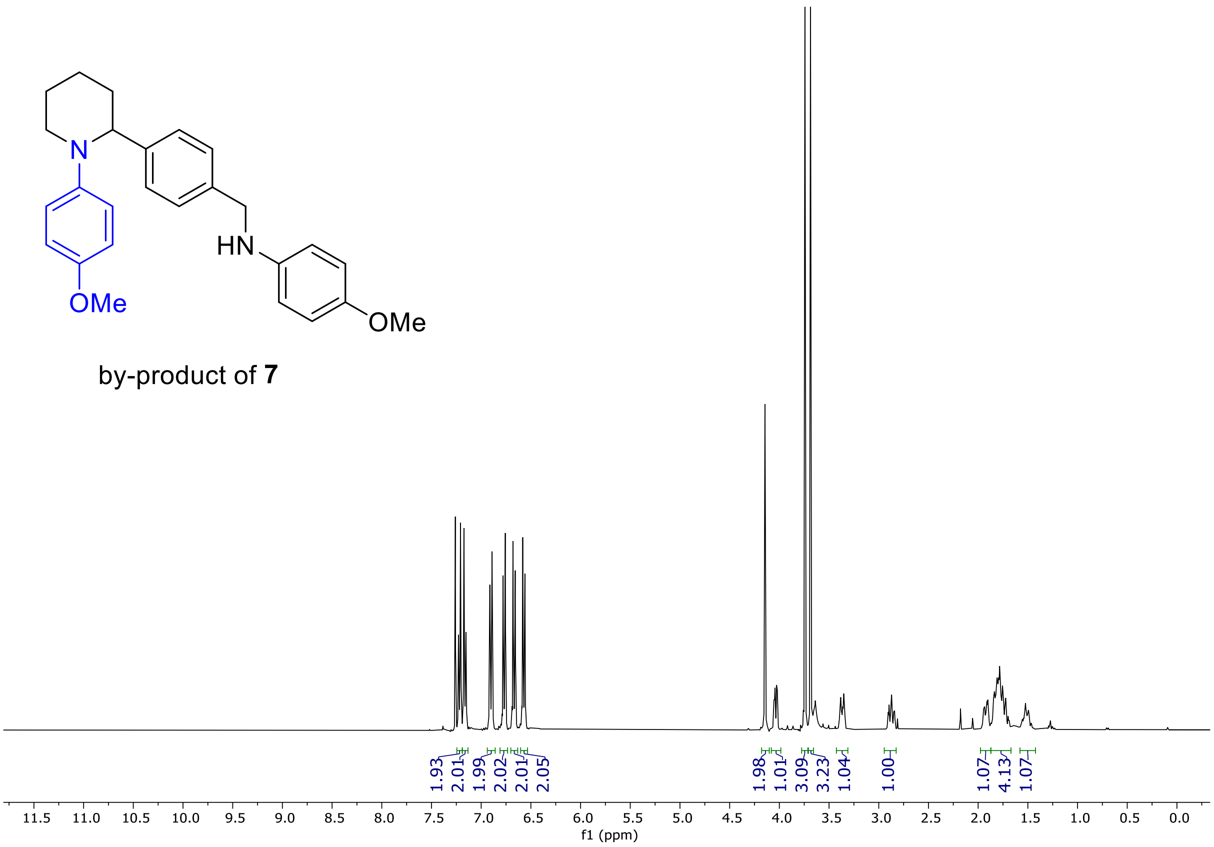

by-product of **7**

^{1}H NMR (400 MHz, $CDCl_3$) spectrum of the by-product of **7**

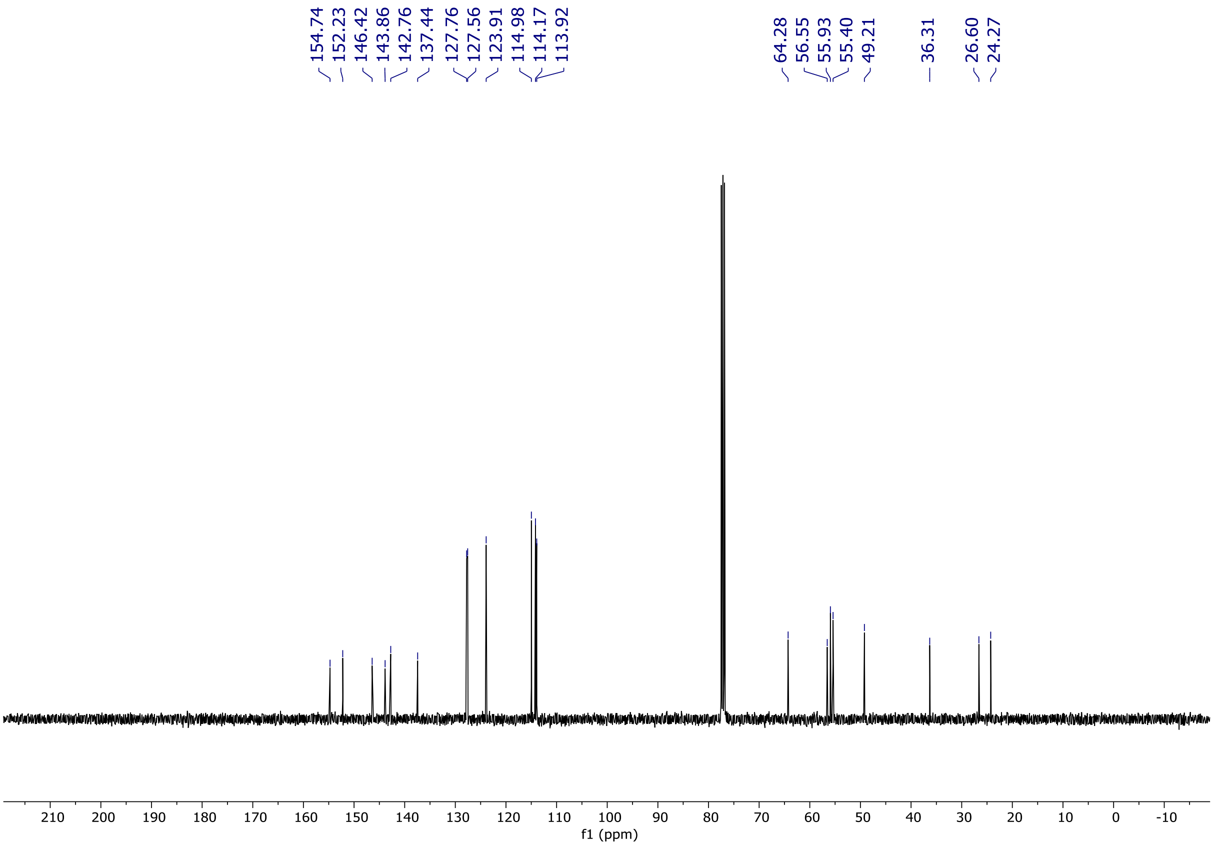

$^{13}C\{^{1}H\}$ NMR (101 MHz, $CDCl_3$) spectrum of the by-product of **7**

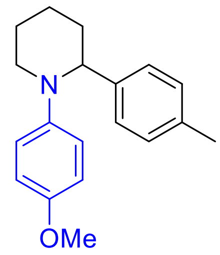

8

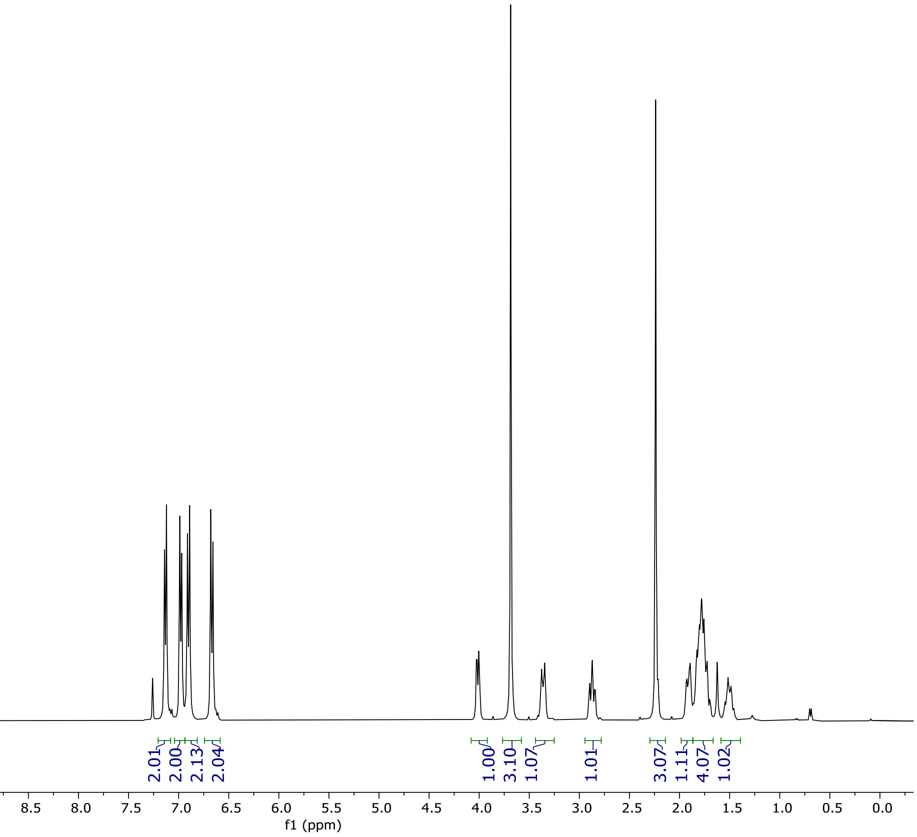

^{1}H NMR (400 MHz, $CDCl_3$) spectrum of compound **8**

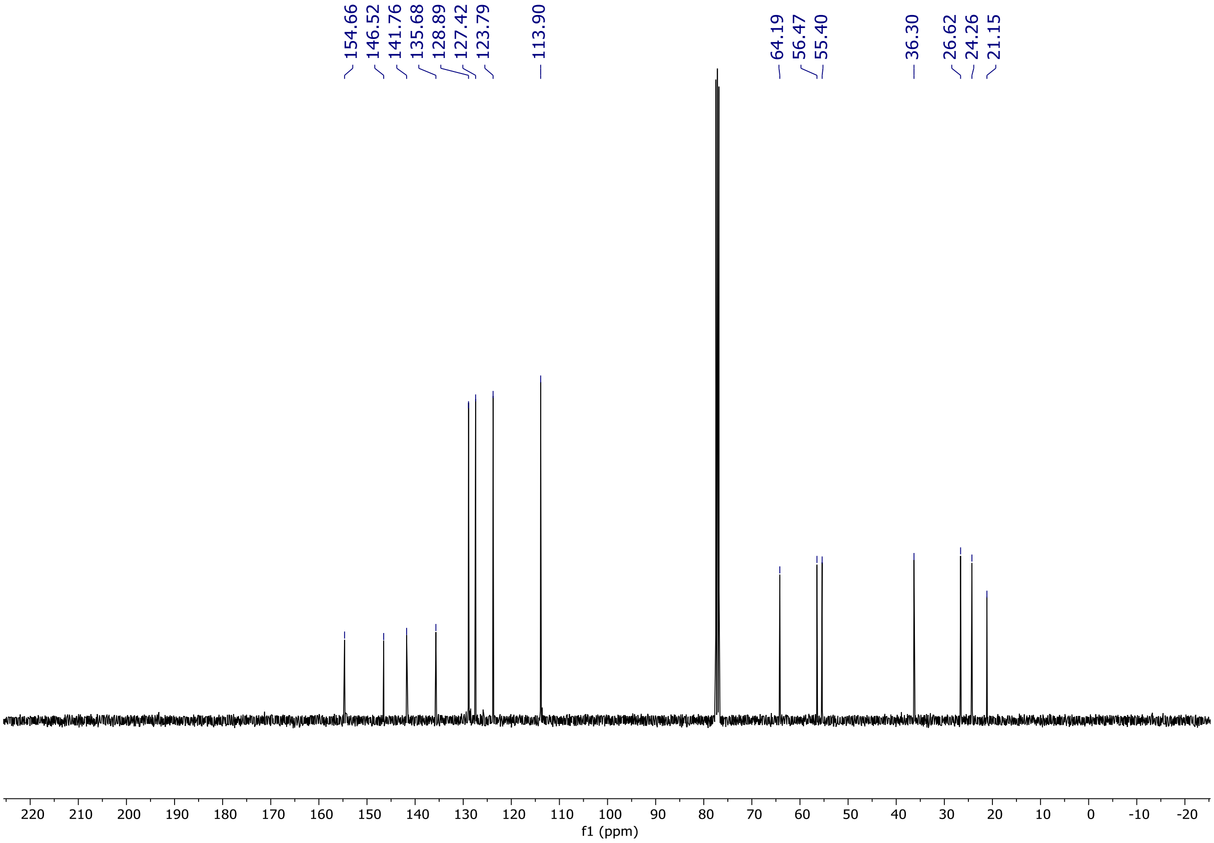

^{13}C{^{1}H} NMR (101 MHz, $CDCl_3$) spectrum of compound **8**

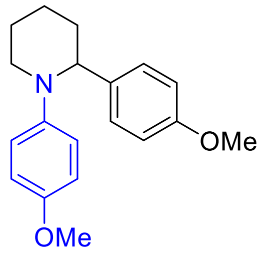

9

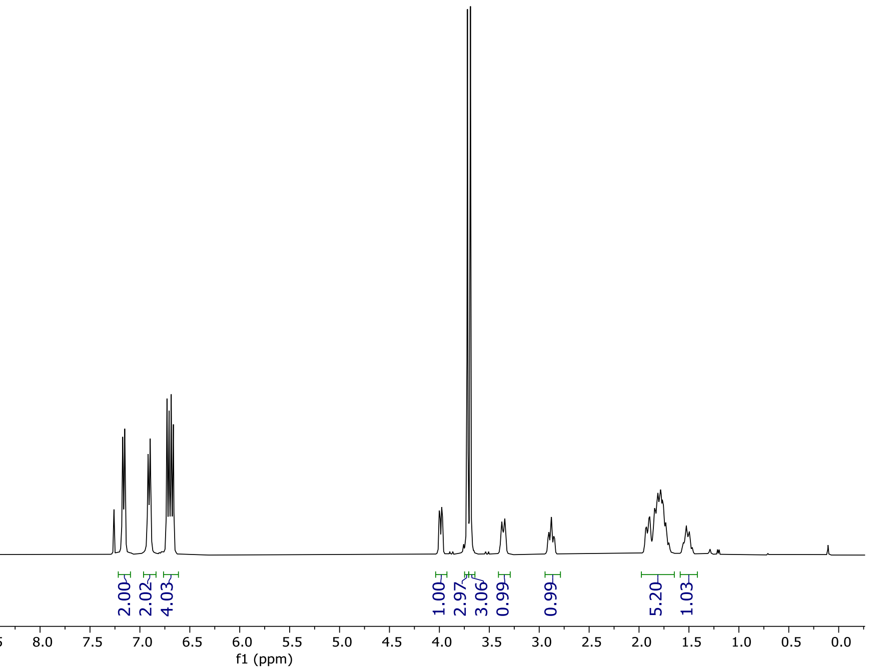

^{1}H NMR (400 MHz, $CDCl_3$) spectrum of compound **9**

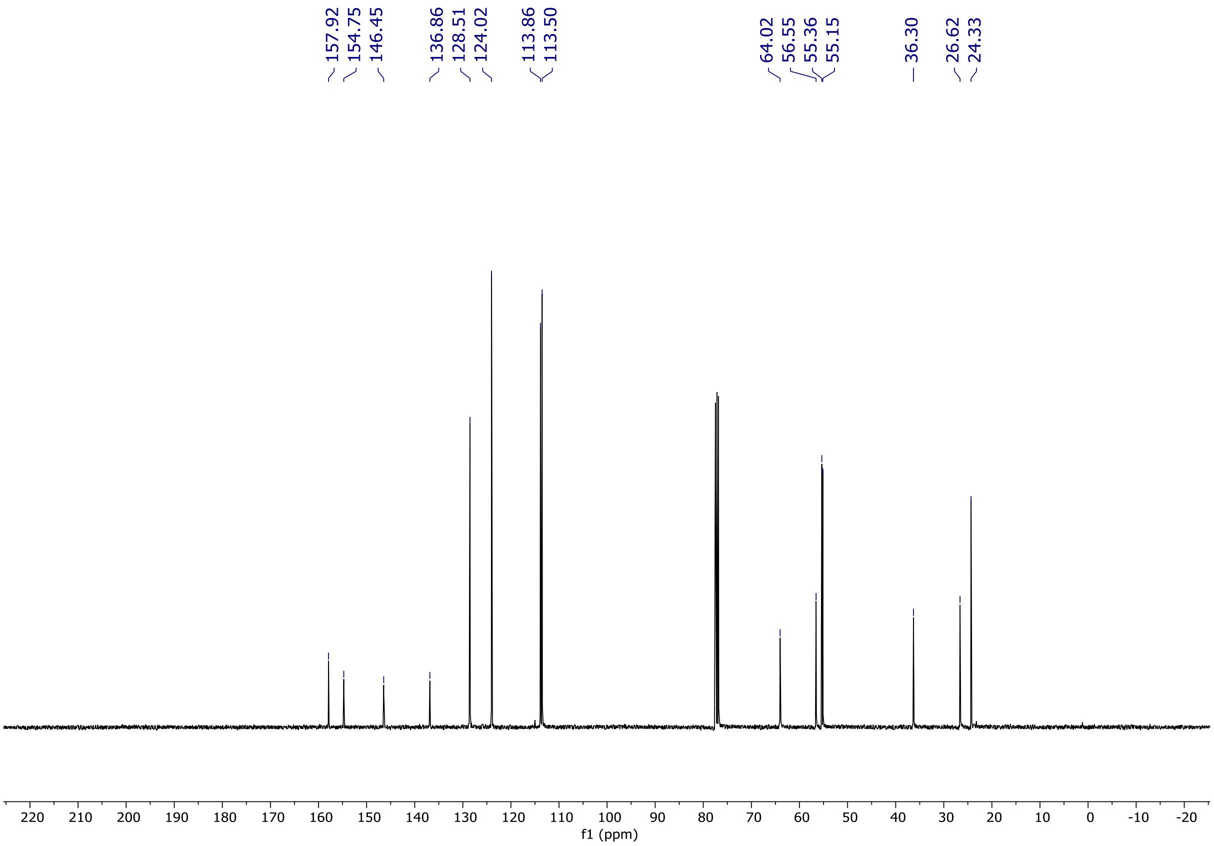

$^{13}C\{^{1}H\}$ NMR (101 MHz, $CDCl_3$) spectrum of compound **9**

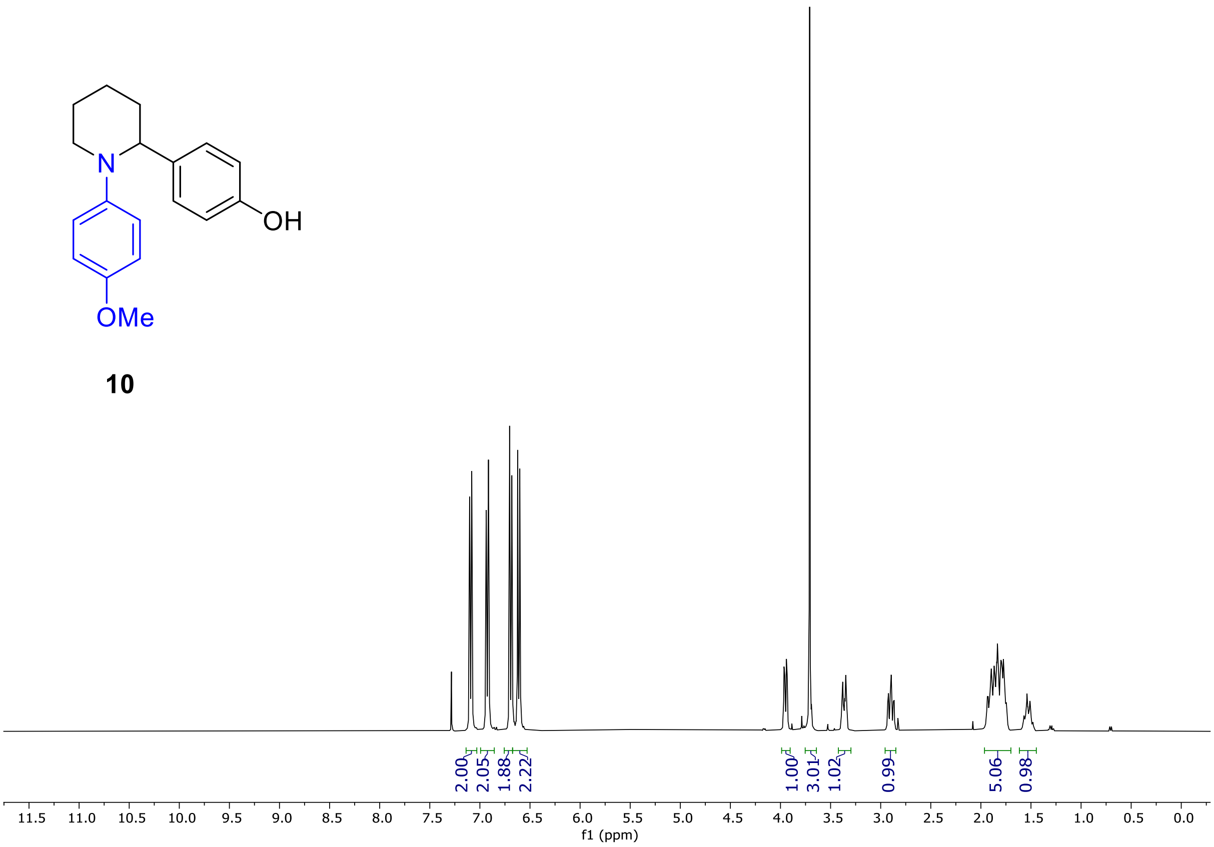

^{1}H NMR (400 MHz, $CDCl_3$) spectrum of compound **10**

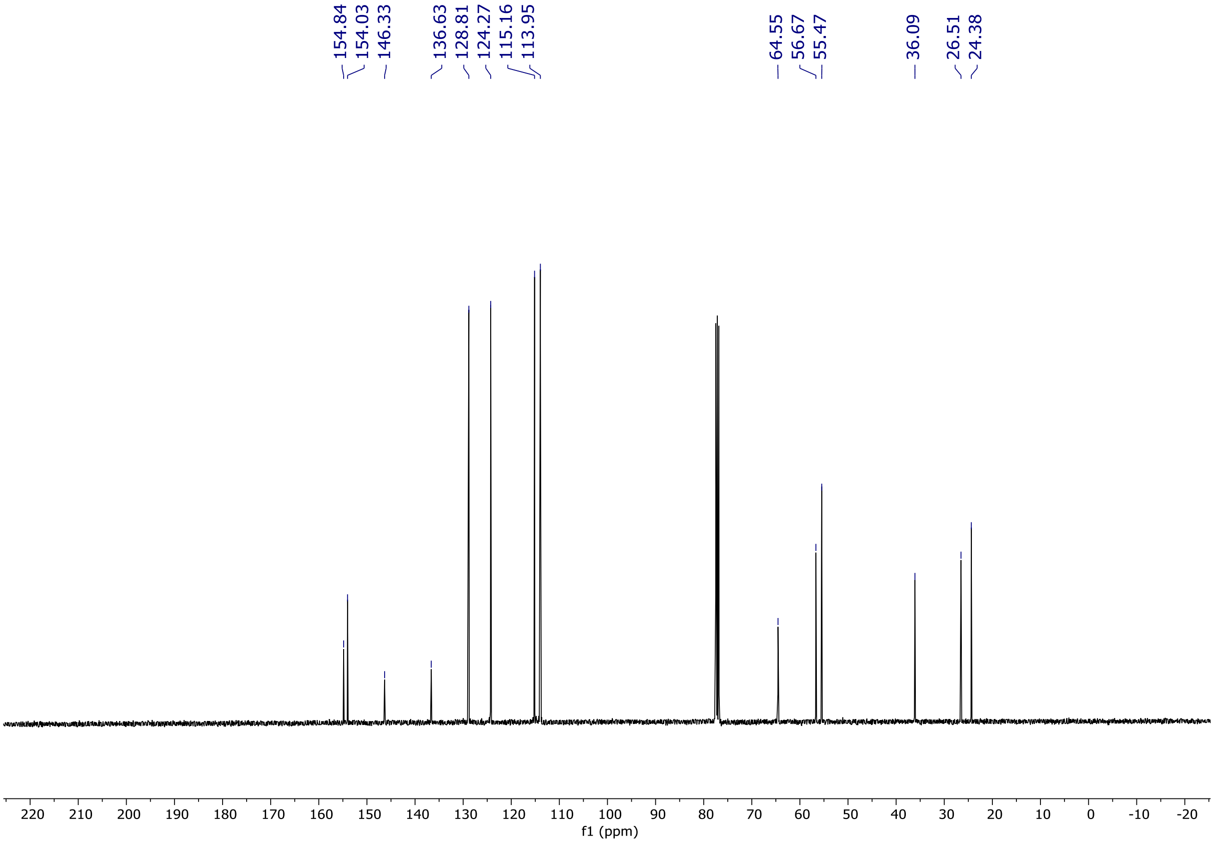

^{13}C{^{1}H} NMR (101 MHz, $CDCl_3$) spectrum of compound **10**

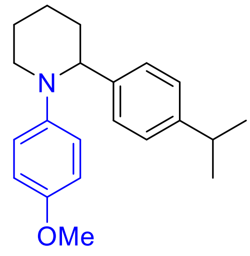

11

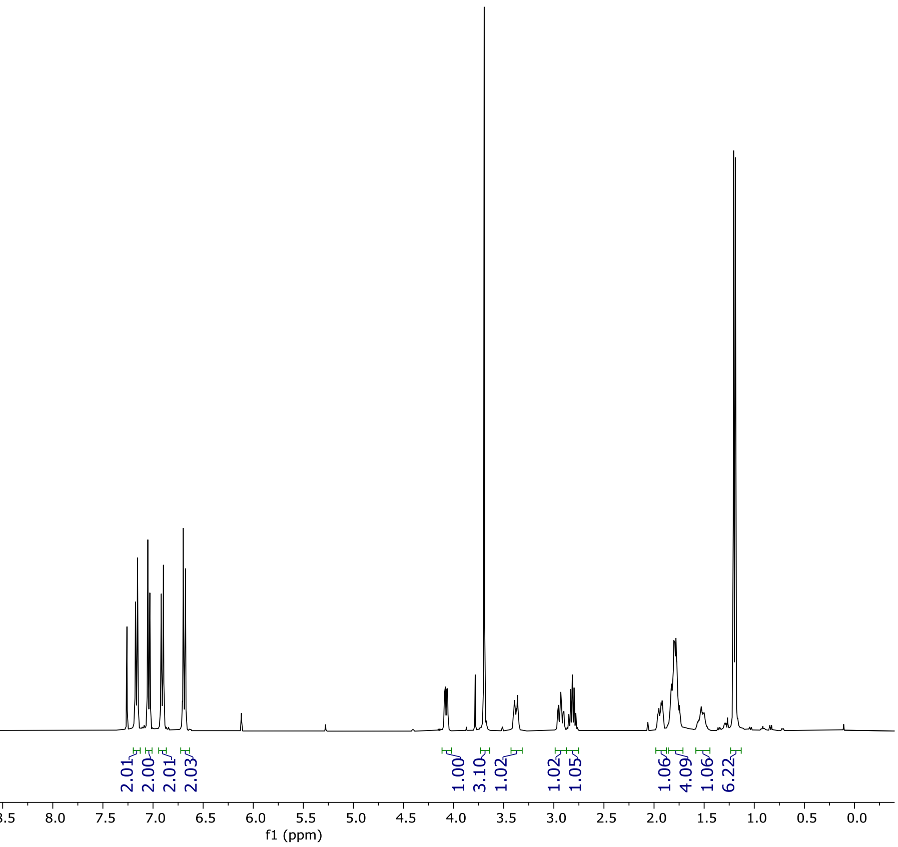

^{1}H NMR (400 MHz, $CDCl_3$) spectrum of compound **11**

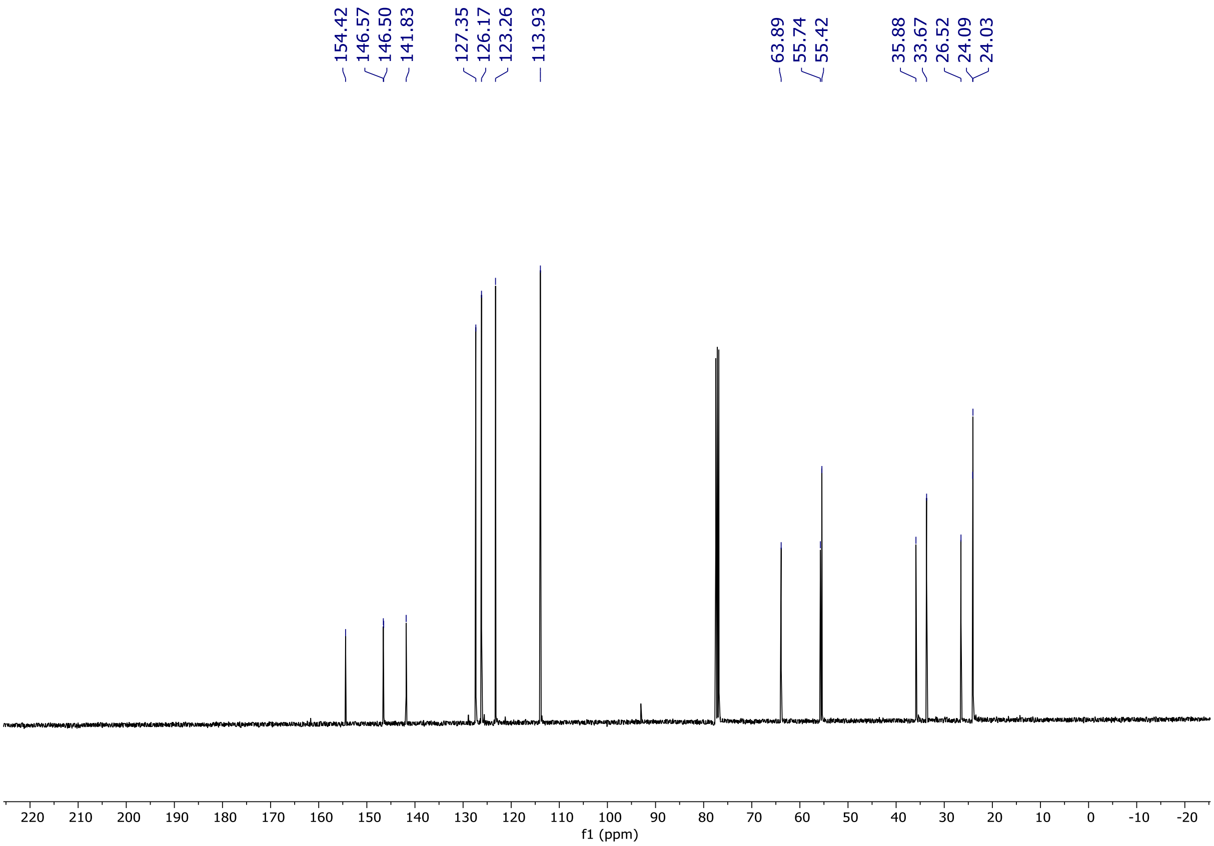

^{13}C{^{1}H} NMR (101 MHz, $CDCl_3$) spectrum of compound **11**

^{1}H NMR (400 MHz, $CDCl_3$) spectrum of compound **2**

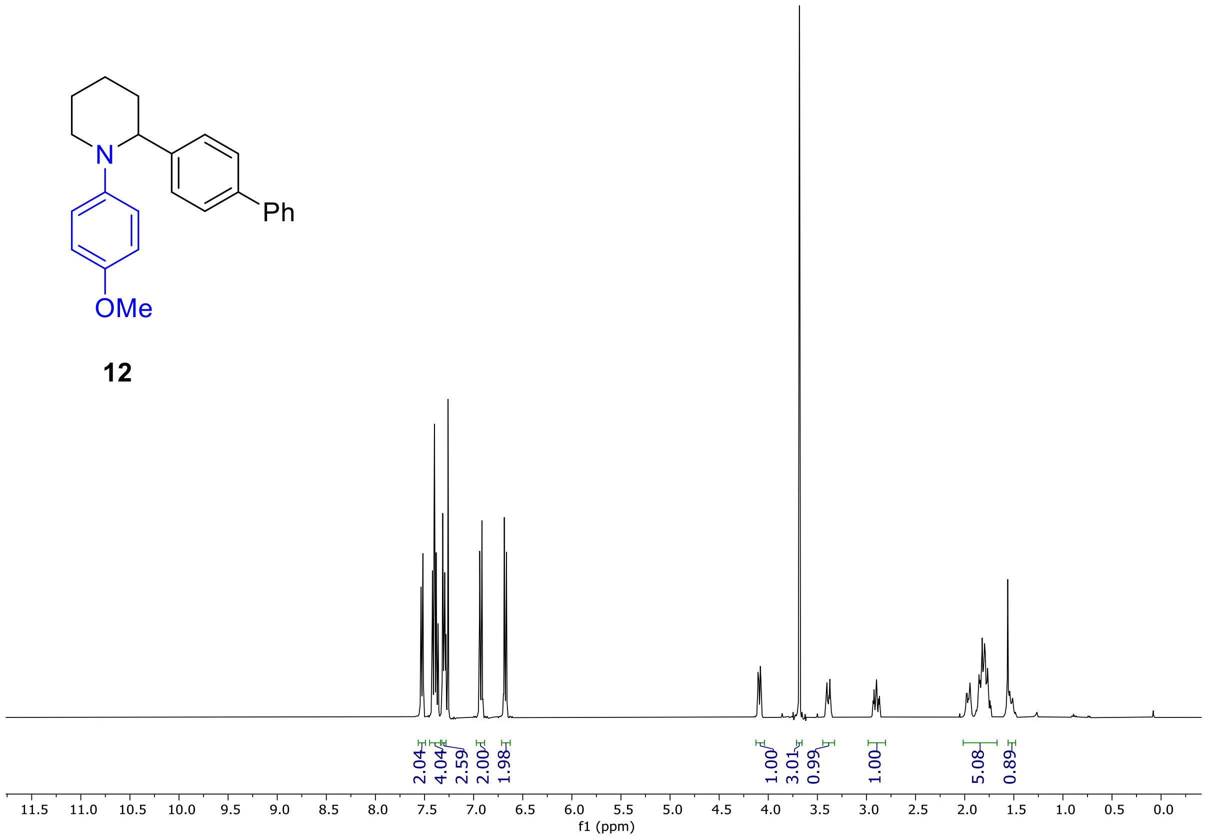

^{1}H NMR (400 MHz, $CDCl_3$) spectrum of compound **12**

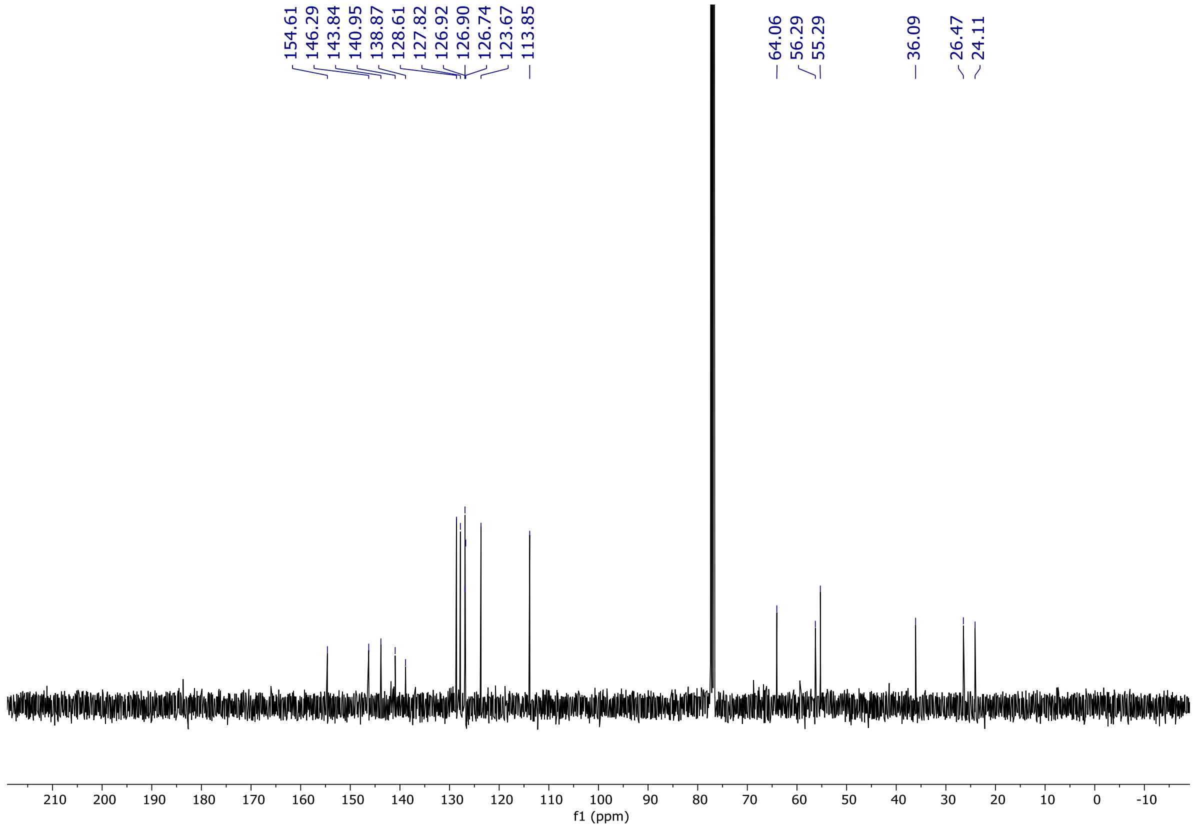

$^{13}C\{^{1}H\}$ NMR (101 MHz, $CDCl_3$) spectrum of compound **12**

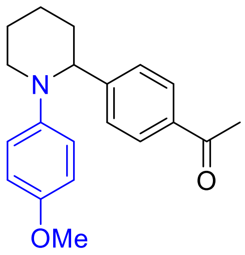

13

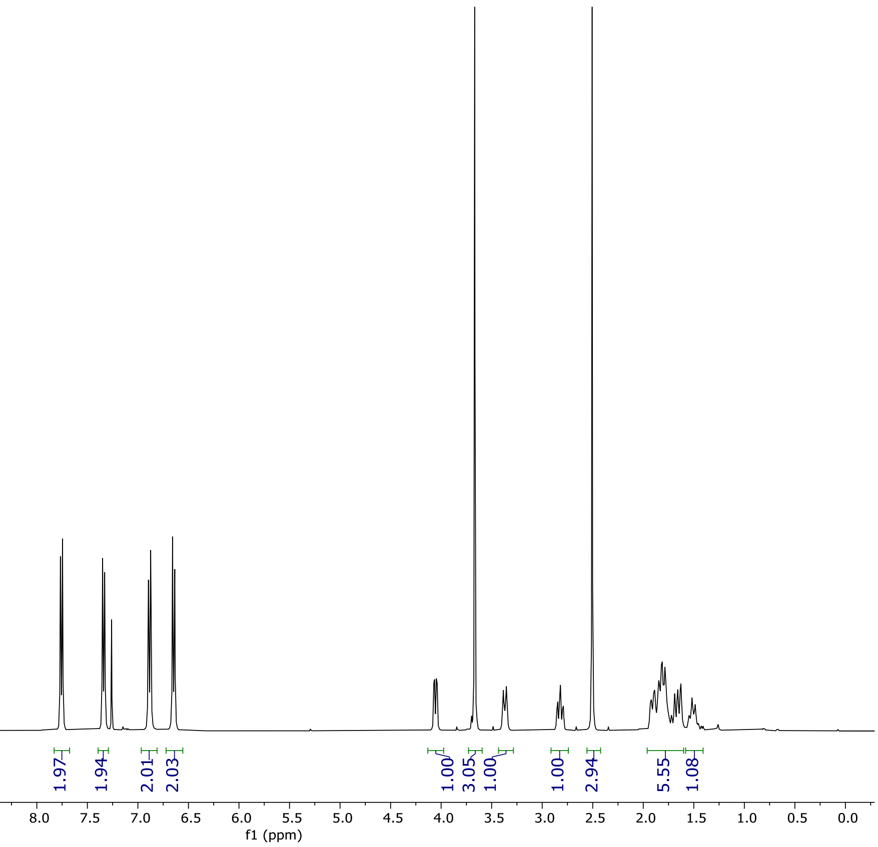

^{1}H NMR (400 MHz, $CDCl_3$) spectrum of compound **13**

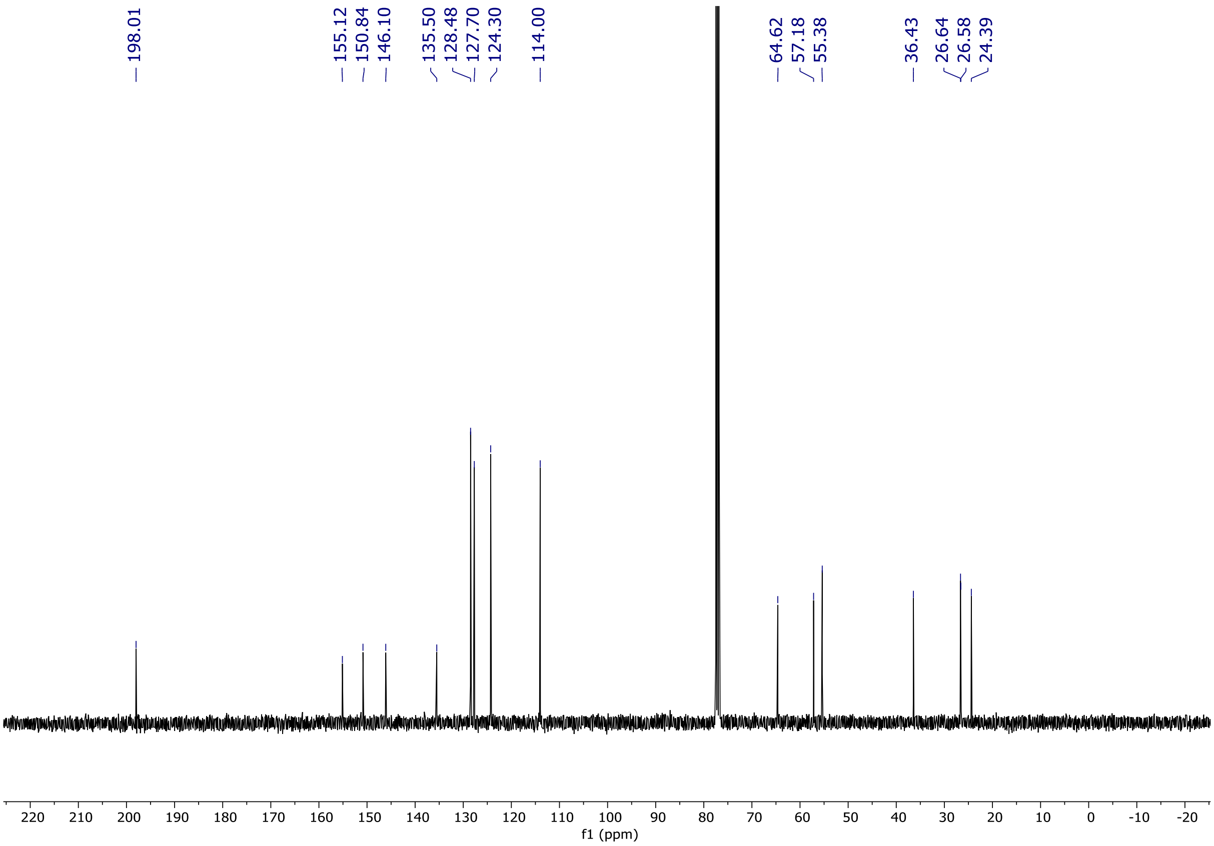

$^{13}C\{^{1}H\}$ NMR (101 MHz, $CDCl_3$) spectrum of compound **13**

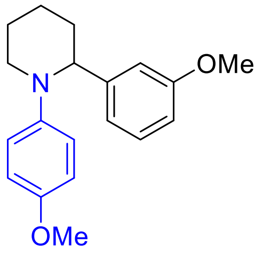

14

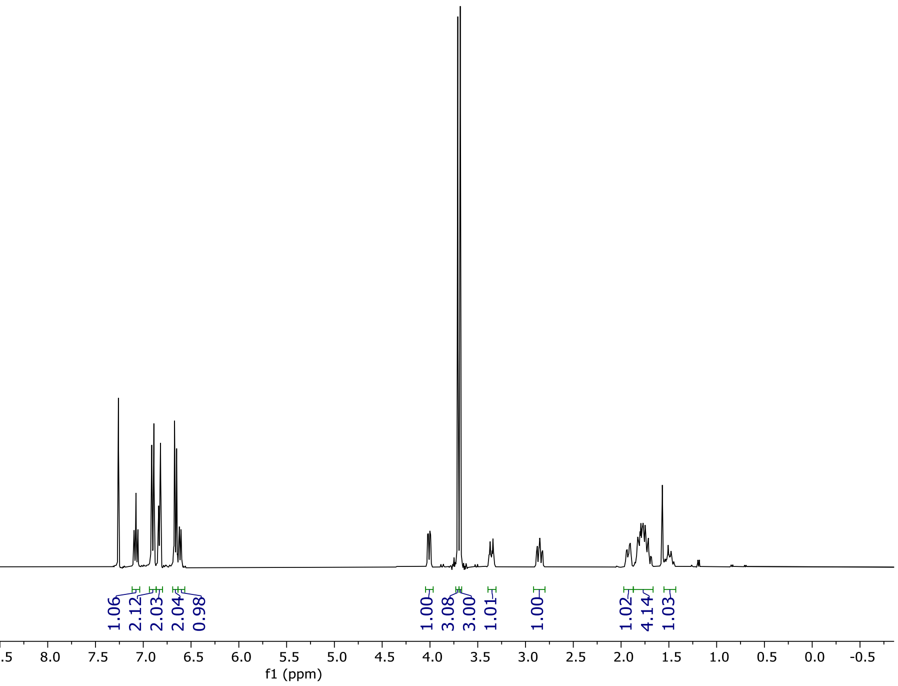

^{1}H NMR (400 MHz, $CDCl_3$) spectrum of compound **14**

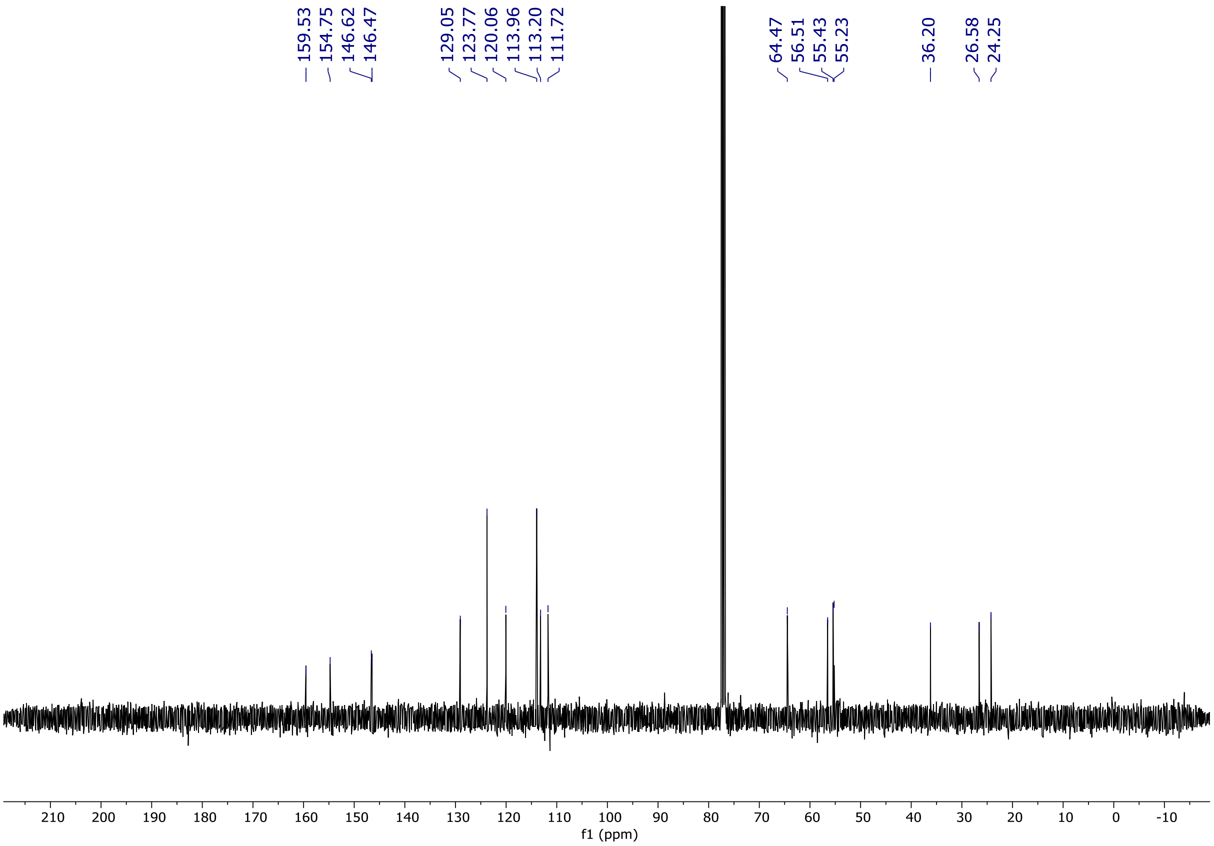

$^{13}C\{^{1}H\}$ NMR (101 MHz, $CDCl_3$) spectrum of compound **14**

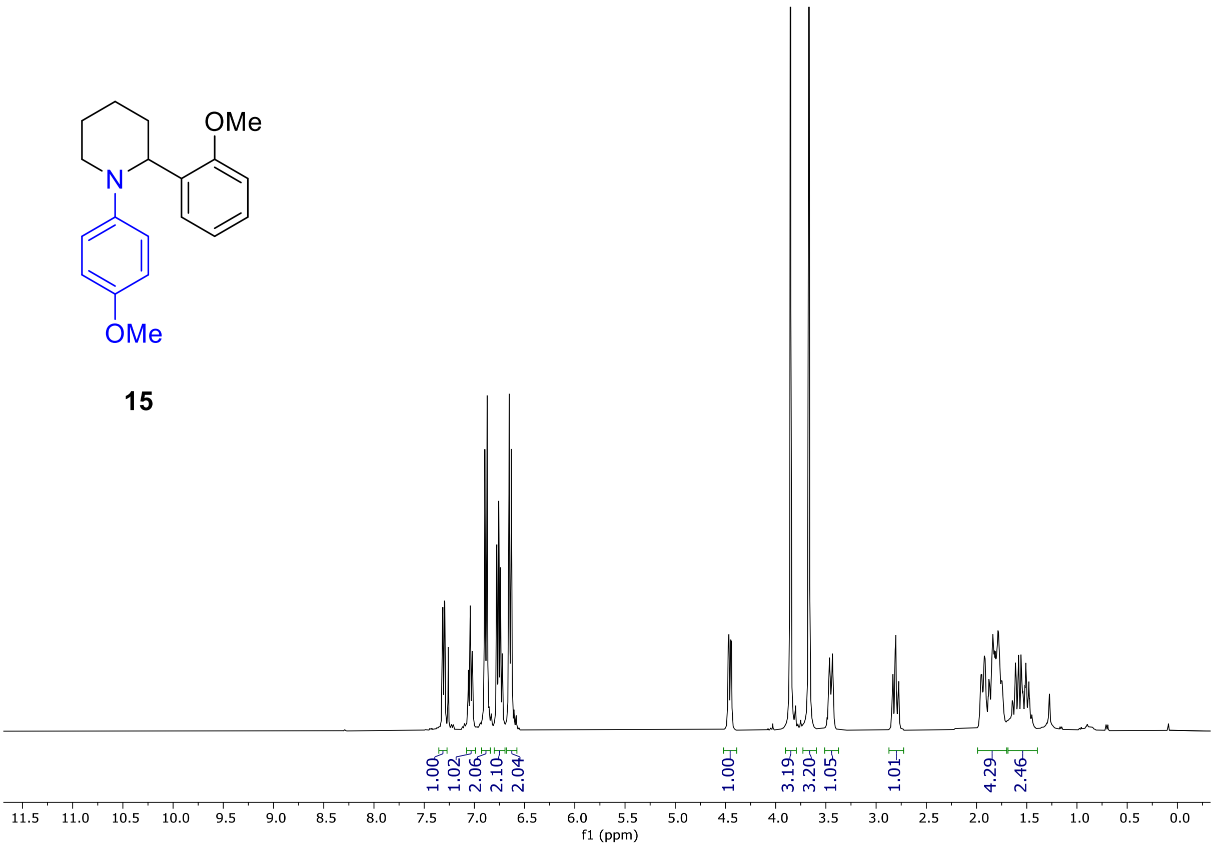

^{1}H NMR (400 MHz, $CDCl_3$) spectrum of compound **15**

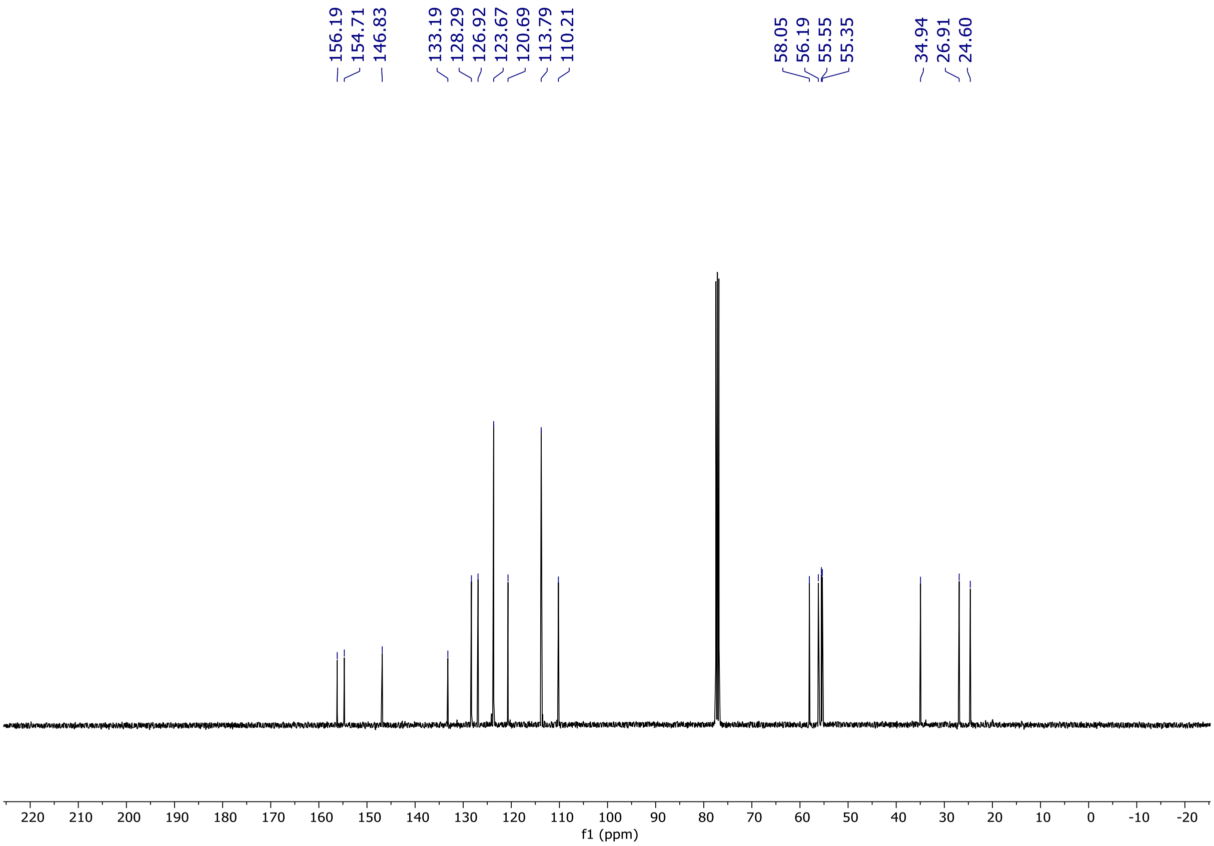

$^{13}C\{^{1}H\}$ NMR (101 MHz, $CDCl_3$) spectrum of compound **15**

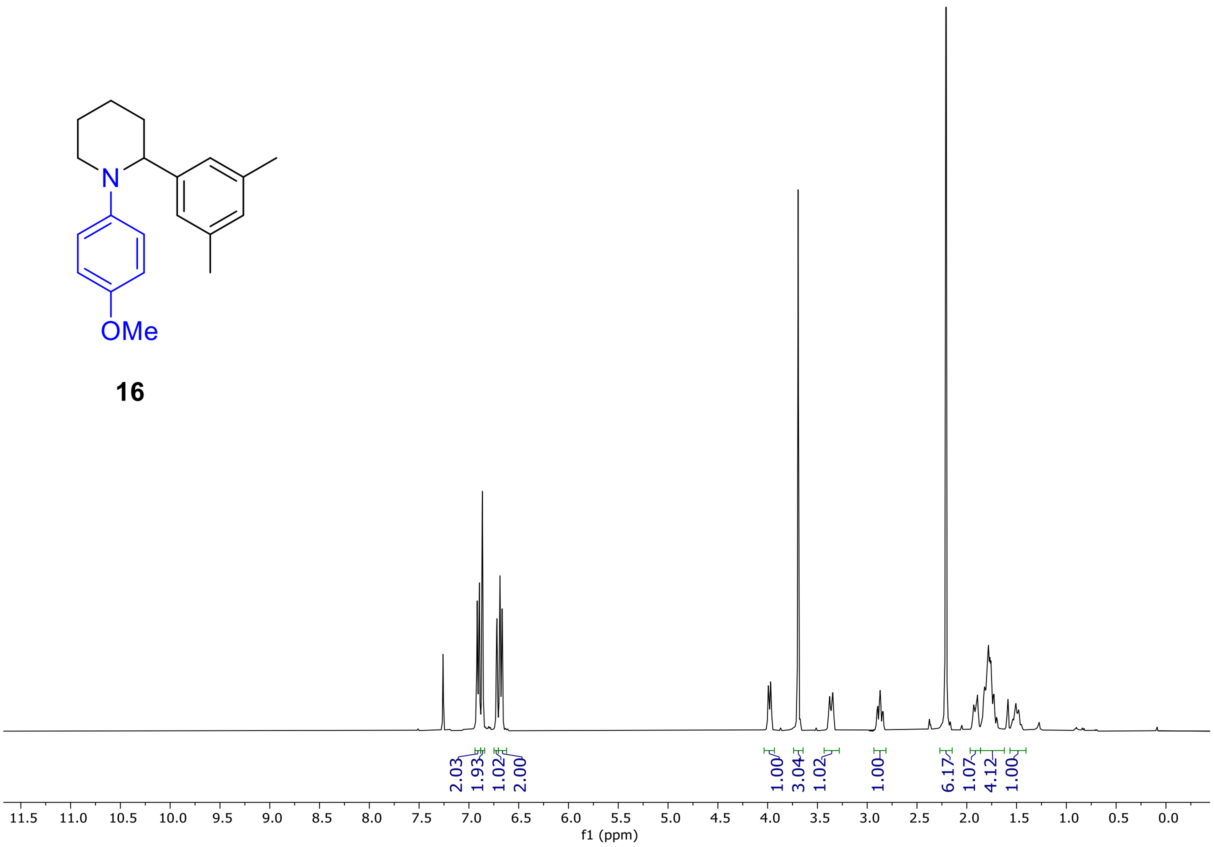

^{1}H NMR (400 MHz, $CDCl_3$) spectrum of compound **16**

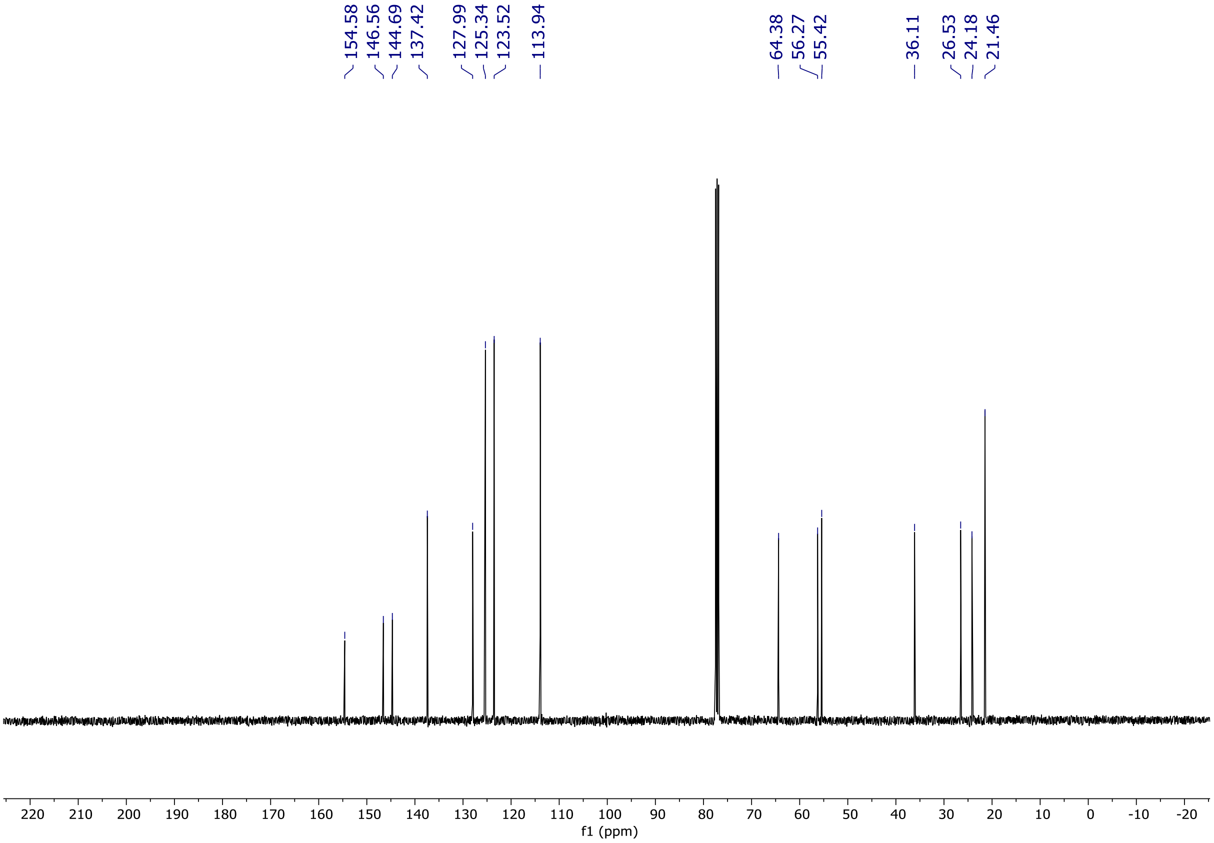

^{13}C{^{1}H} NMR (101 MHz, $CDCl_3$) spectrum of compound **16**

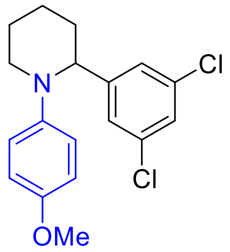

17

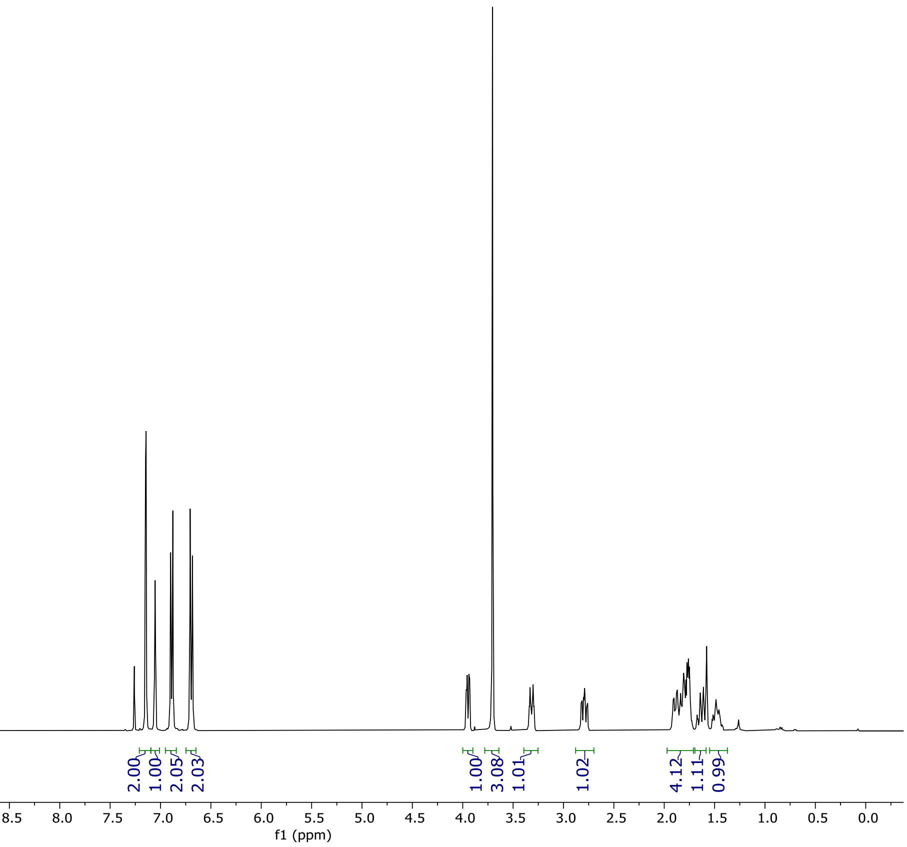

^{1}H NMR (400 MHz, $CDCl_3$) spectrum of compound **17**

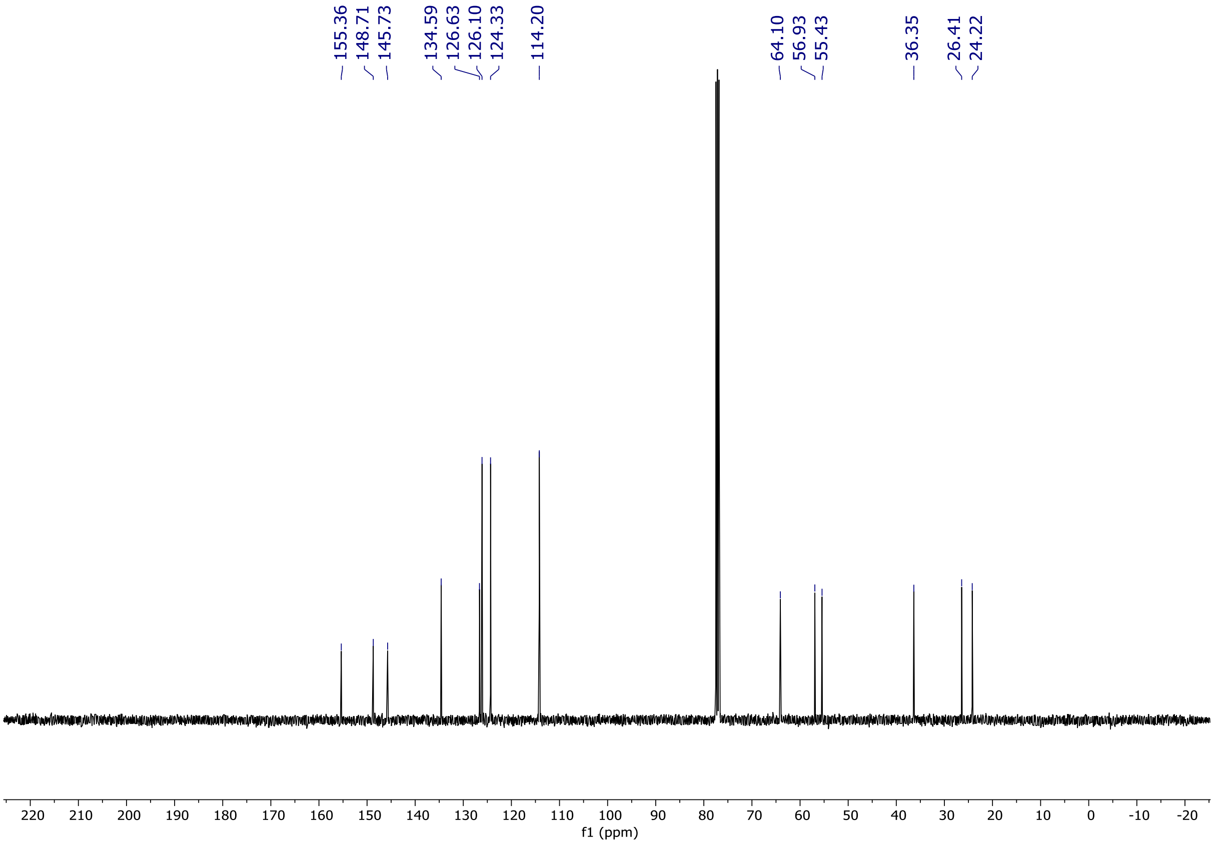

^{13}C{^{1}H} NMR (101 MHz, $CDCl_3$) spectrum of compound **17**

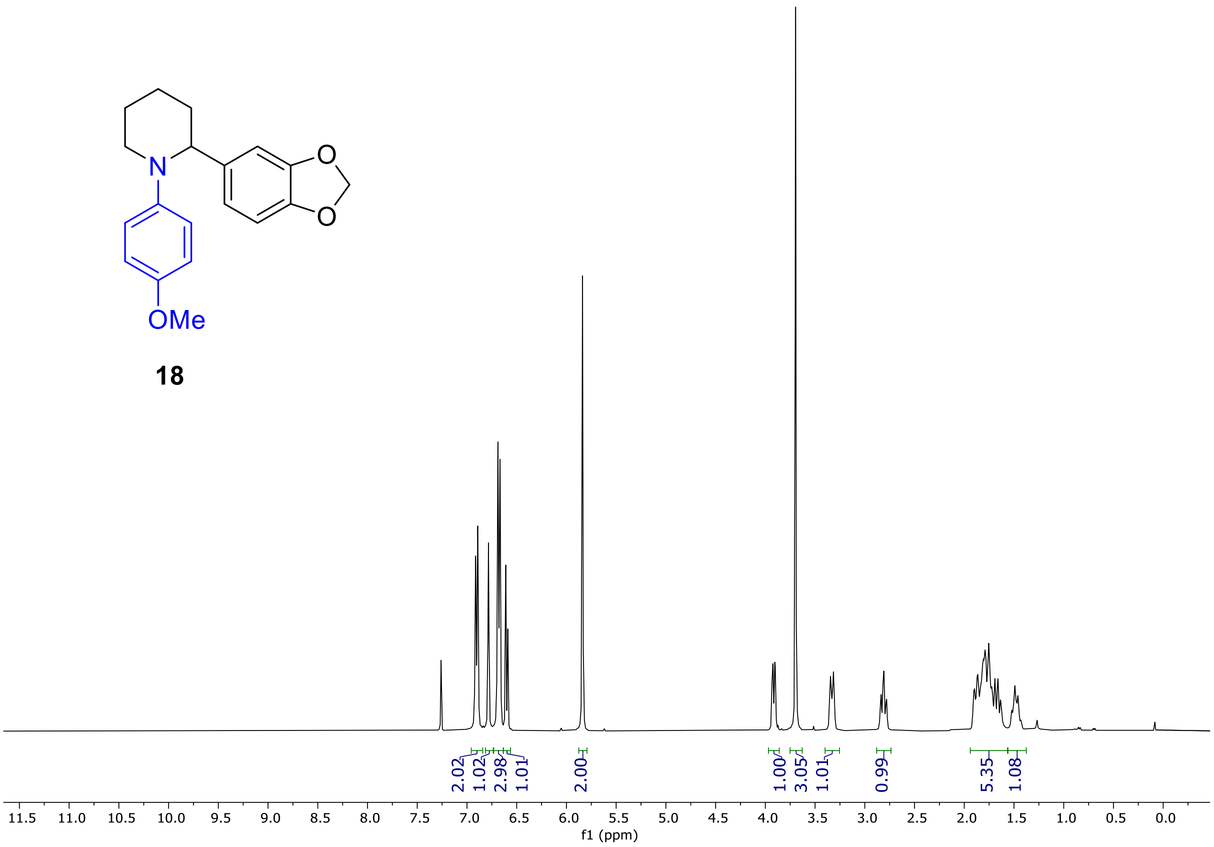

18

^{1}H NMR (400 MHz, $CDCl_3$) spectrum of compound **18**

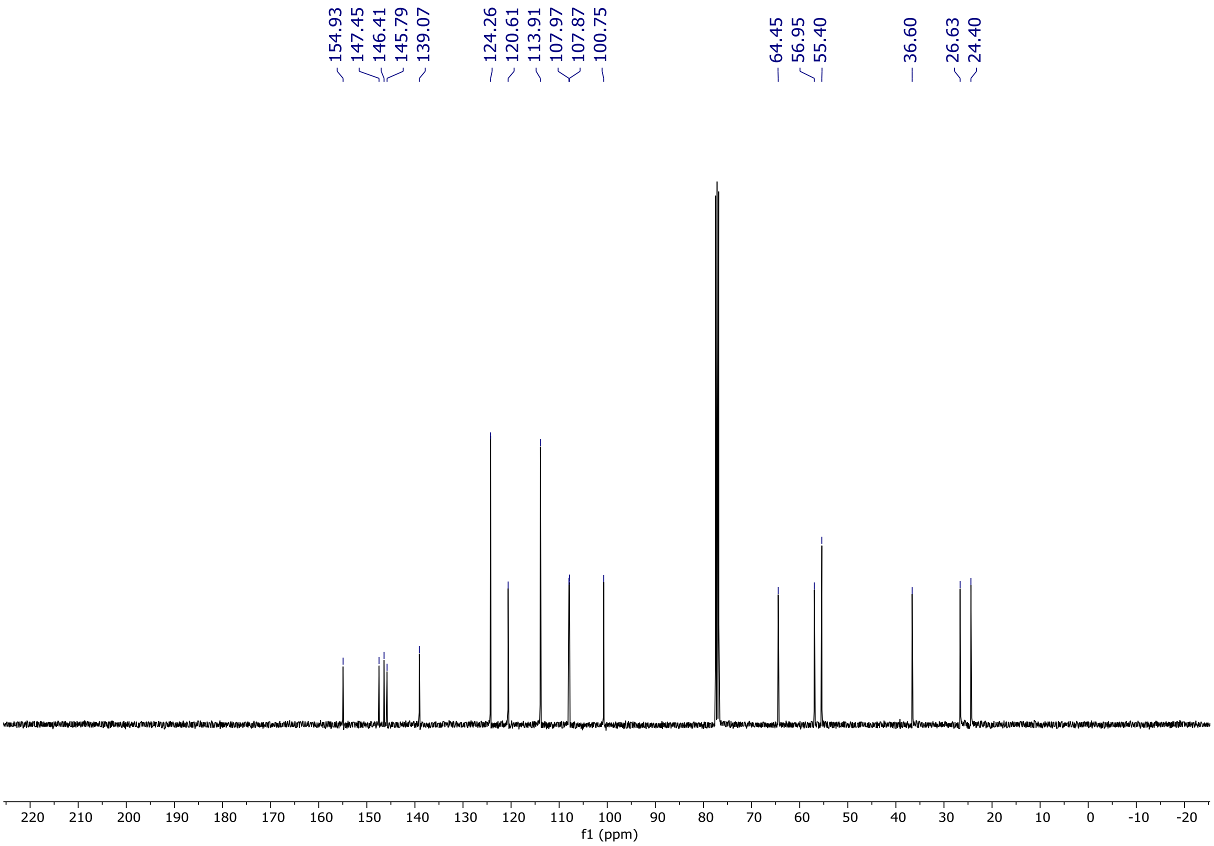

^{13}C{^{1}H} NMR (101 MHz, $CDCl_3$) spectrum of compound **18**

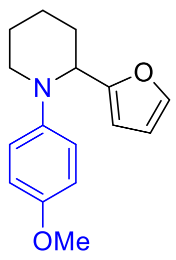

19

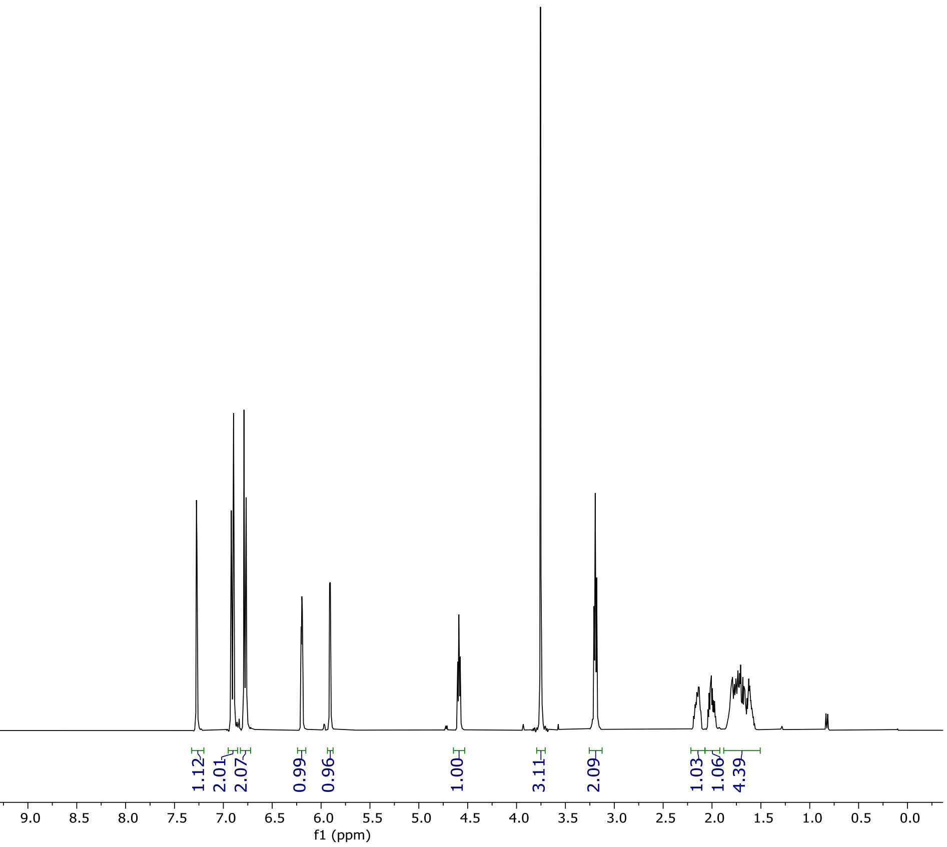

^{1}H NMR (400 MHz, $CDCl_3$) spectrum of compound **19**

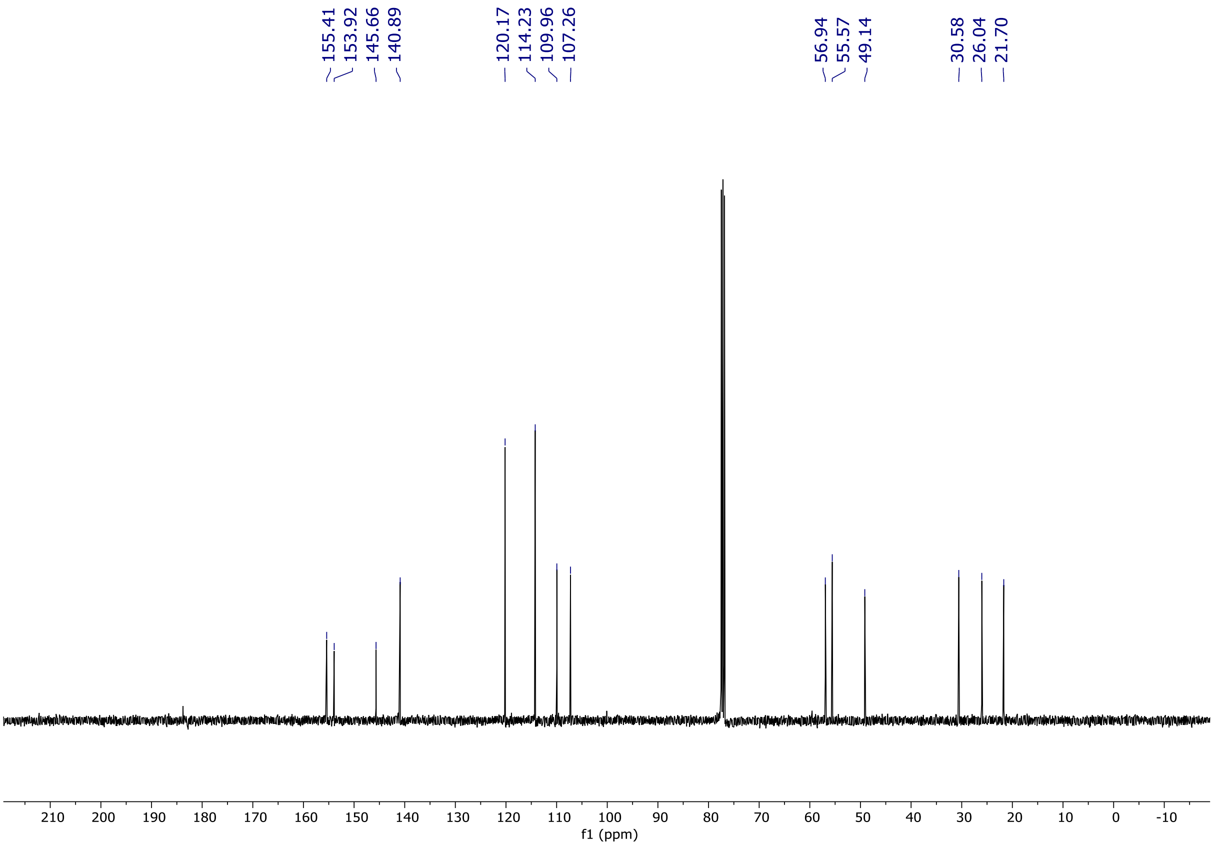

$^{13}C\{^{1}H\}$ NMR (101 MHz, $CDCl_3$) spectrum of compound **19**

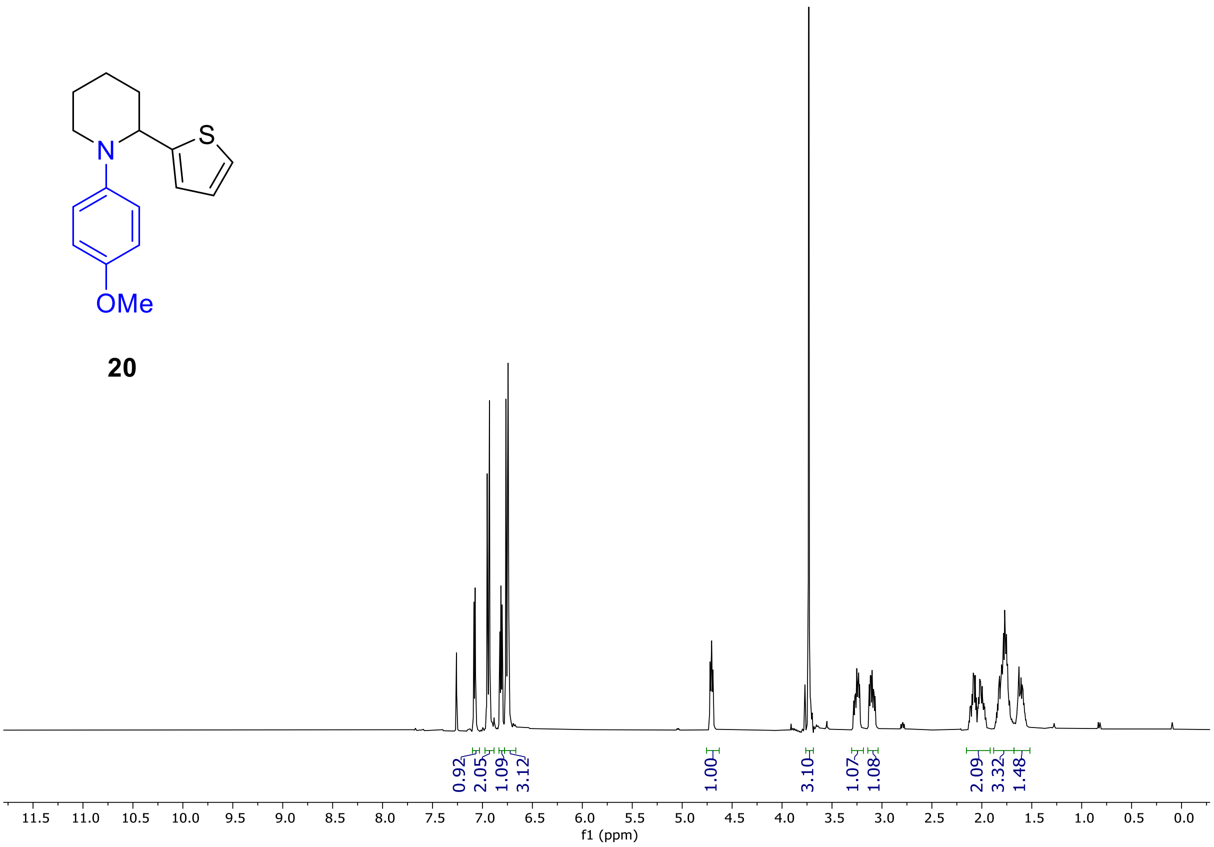

^{1}H NMR (400 MHz, $CDCl_3$) spectrum of compound **20**

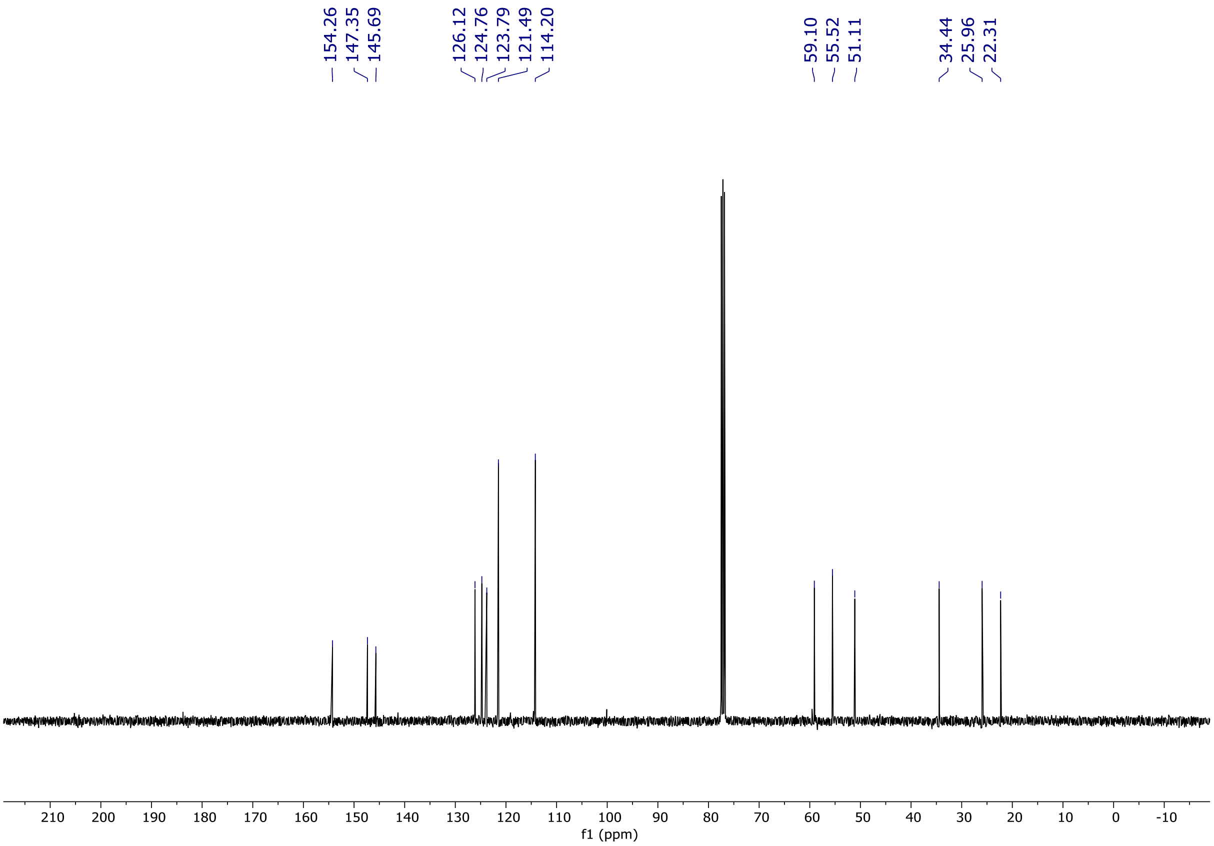

$^{13}C\{^{1}H\}$ NMR (101 MHz, $CDCl_3$) spectrum of compound **20**

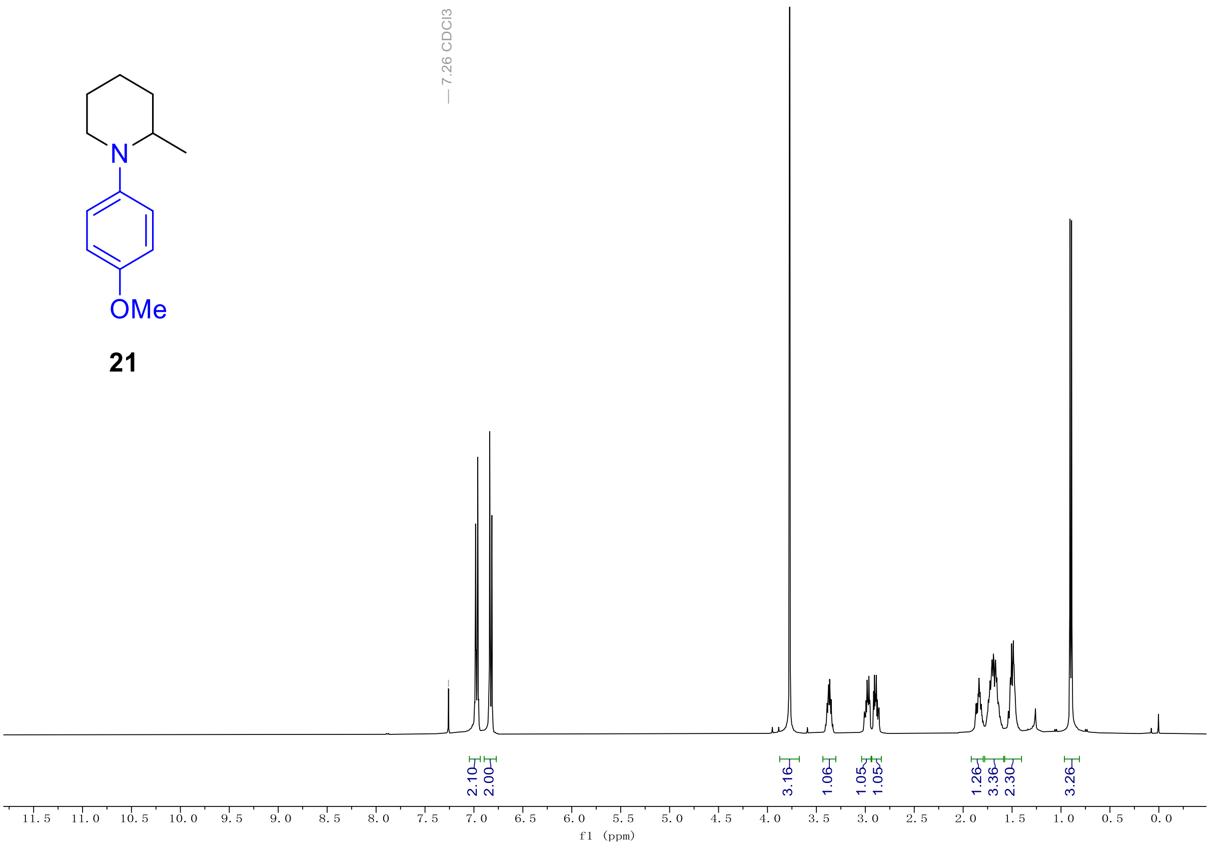

^{1}H NMR (400 MHz, $CDCl_3$) spectrum of compound **21**

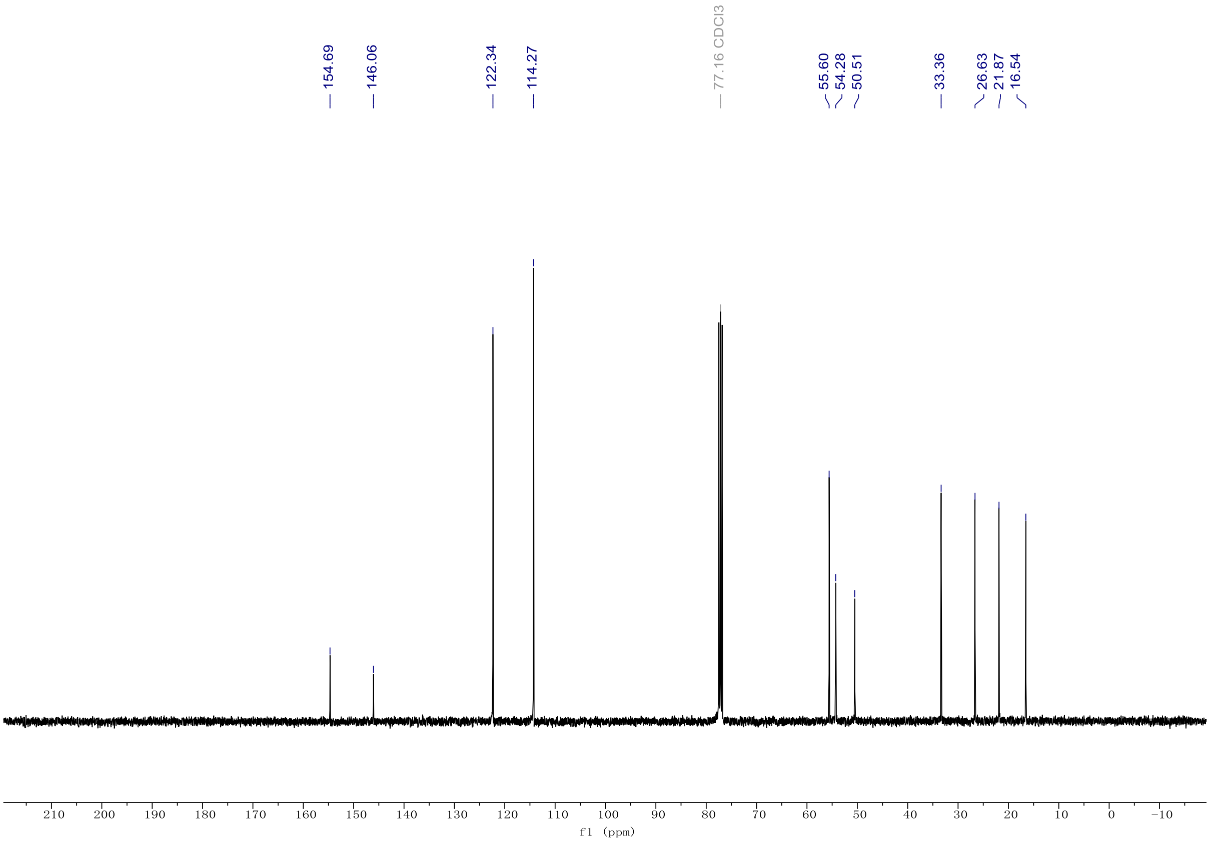

^{13}C{^{1}H} NMR (101 MHz, $CDCl_3$) spectrum of compound **21**

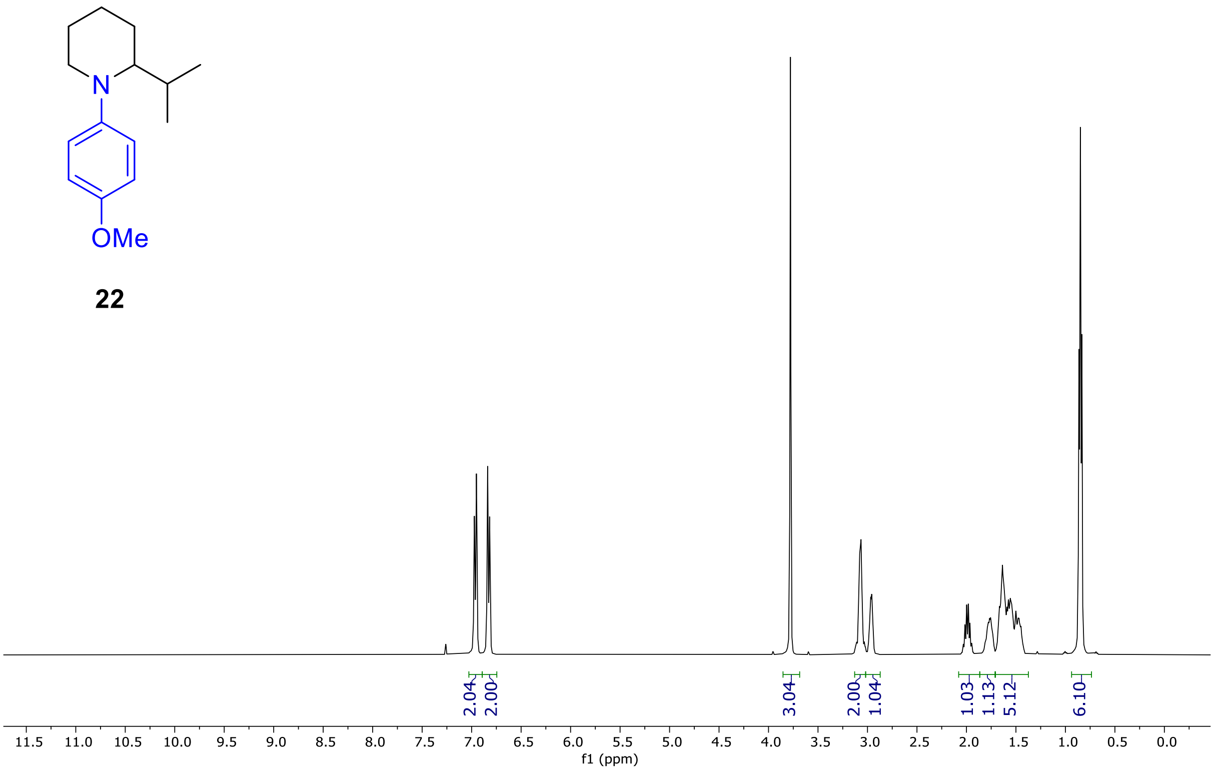

^{1}H NMR (400 MHz, $CDCl_3$) spectrum of compound **22**

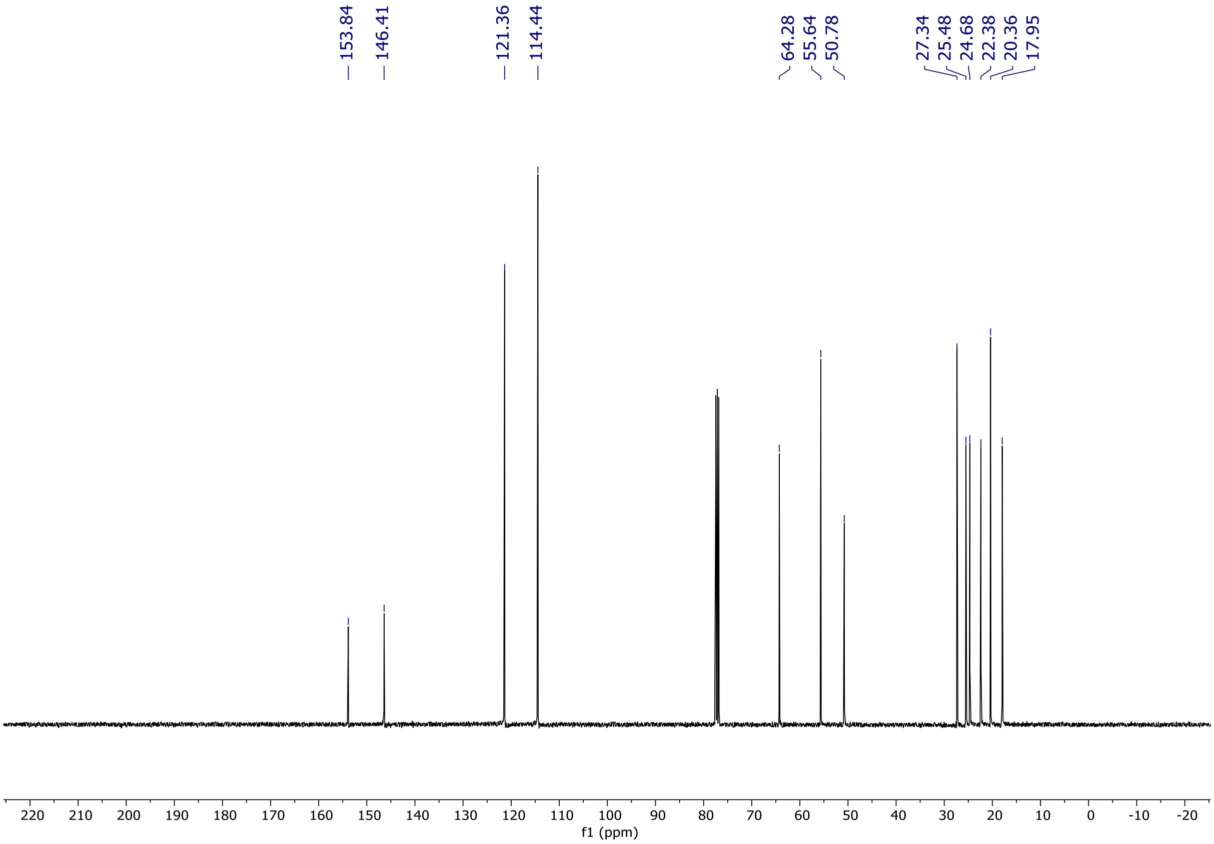

$^{13}C\{^{1}H\}$ NMR (101 MHz, $CDCl_3$) spectrum of compound **22**

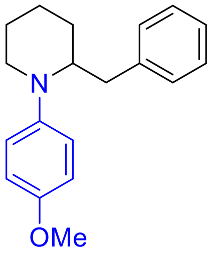

23

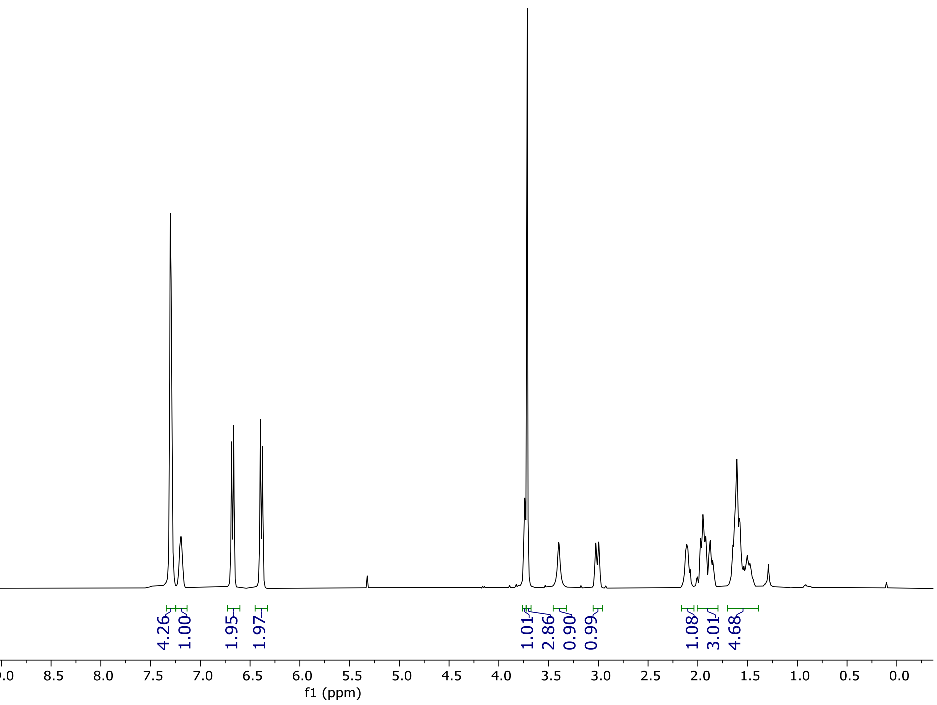

^{1}H NMR (400 MHz, $CDCl_3$) spectrum of compound **23**

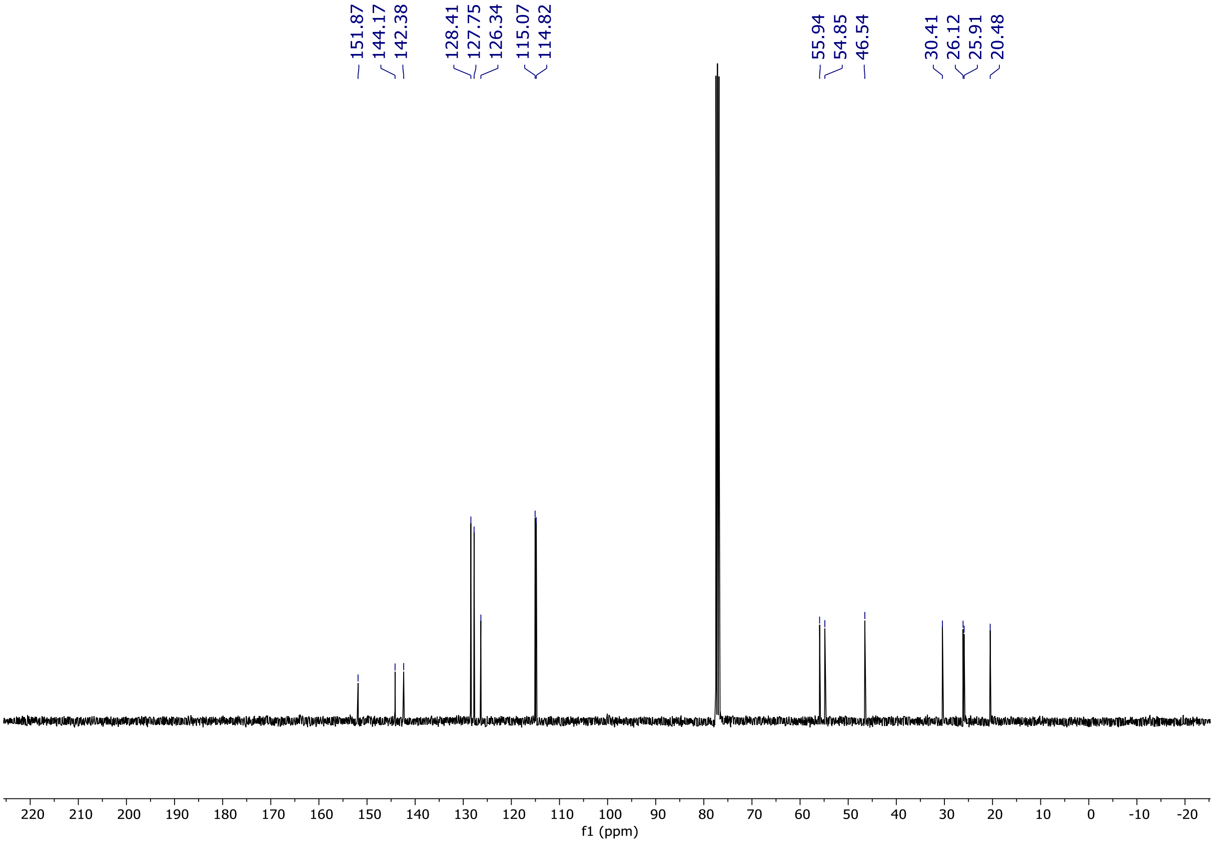

$^{13}C\{^{1}H\}$ NMR (101 MHz, $CDCl_3$) spectrum of compound **23**

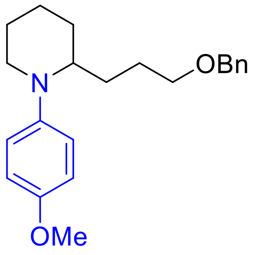

24

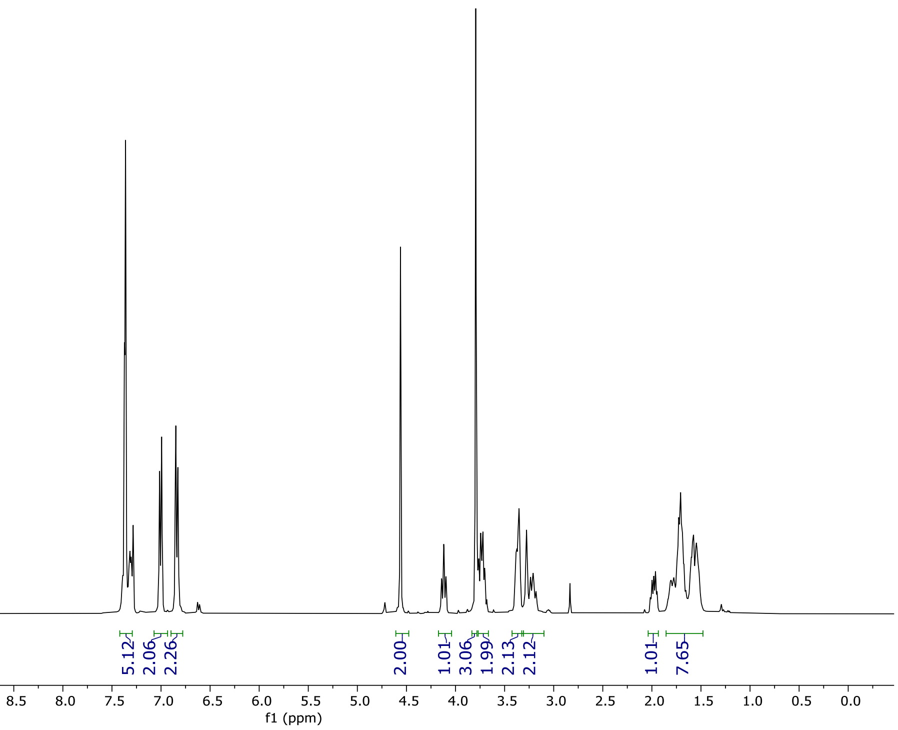

^{1}H NMR (400 MHz, $CDCl_3$) spectrum of compound **24**

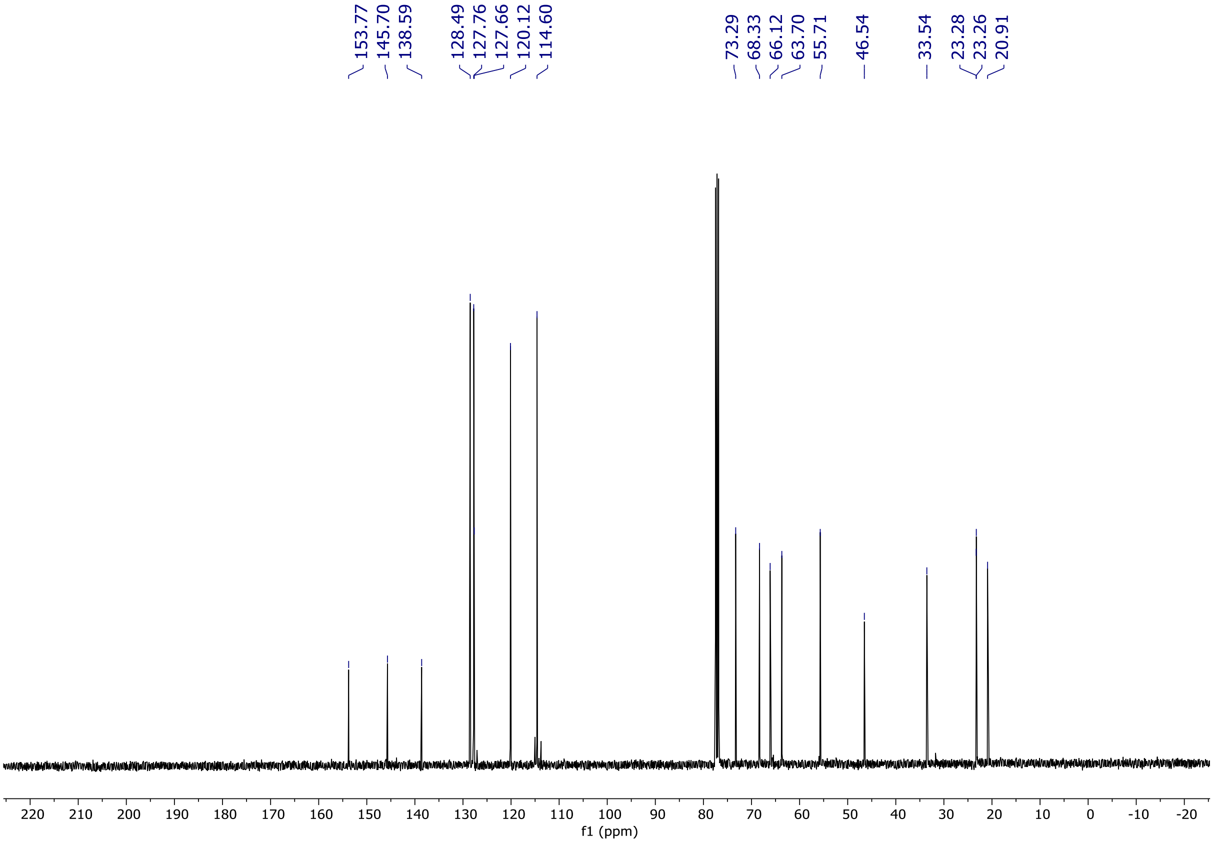

^{13}C{^{1}H} NMR (101 MHz, $CDCl_3$) spectrum of compound **24**

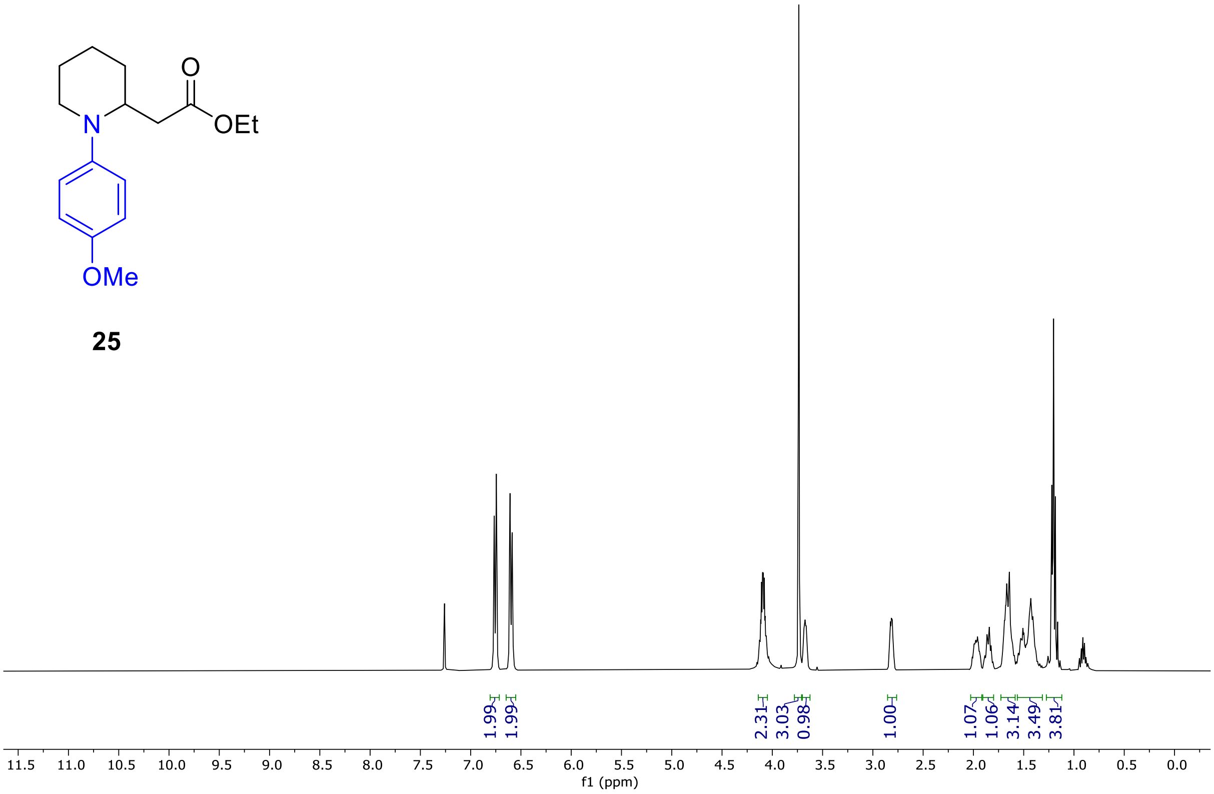

^{1}H NMR (400 MHz, $CDCl_3$) spectrum of compound **25**

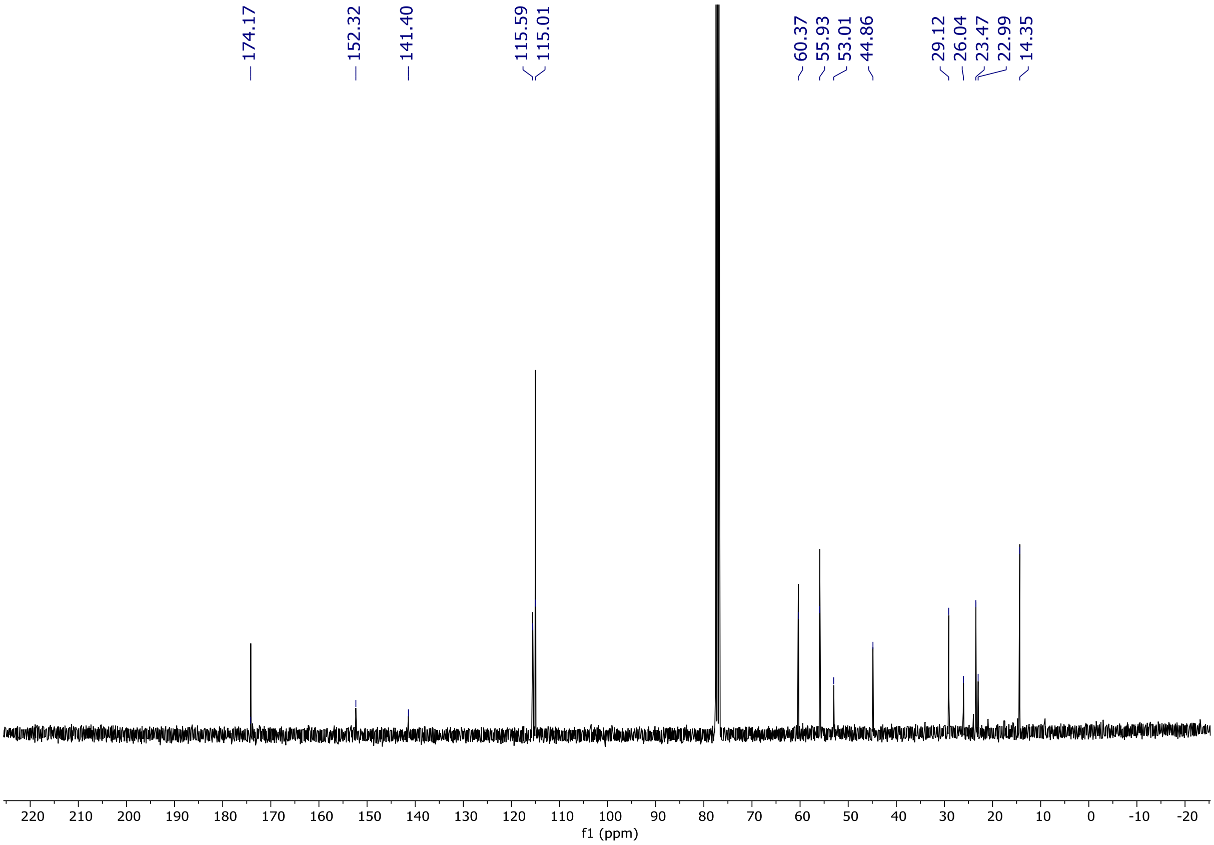

^{13}C{^{1}H} NMR (101 MHz, $CDCl_3$) spectrum of compound **25**

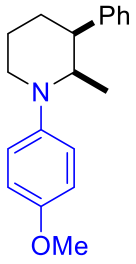

26

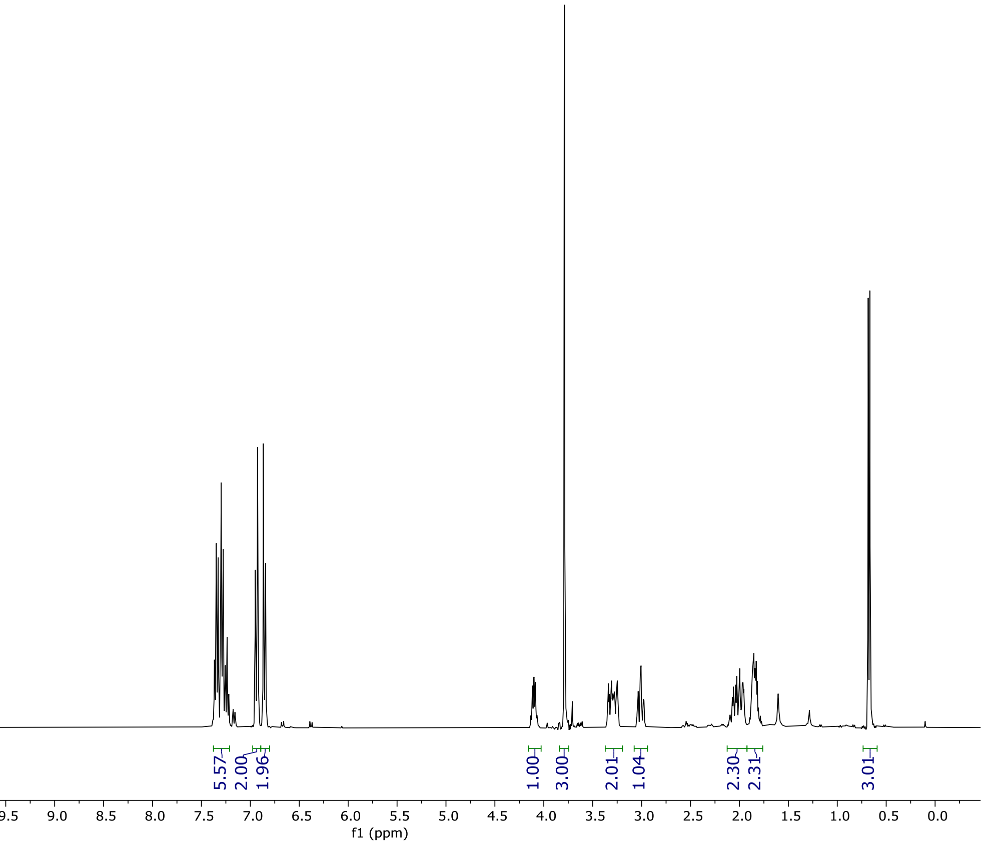

^{1}H NMR (400 MHz, $CDCl_3$) spectrum of compound **26**

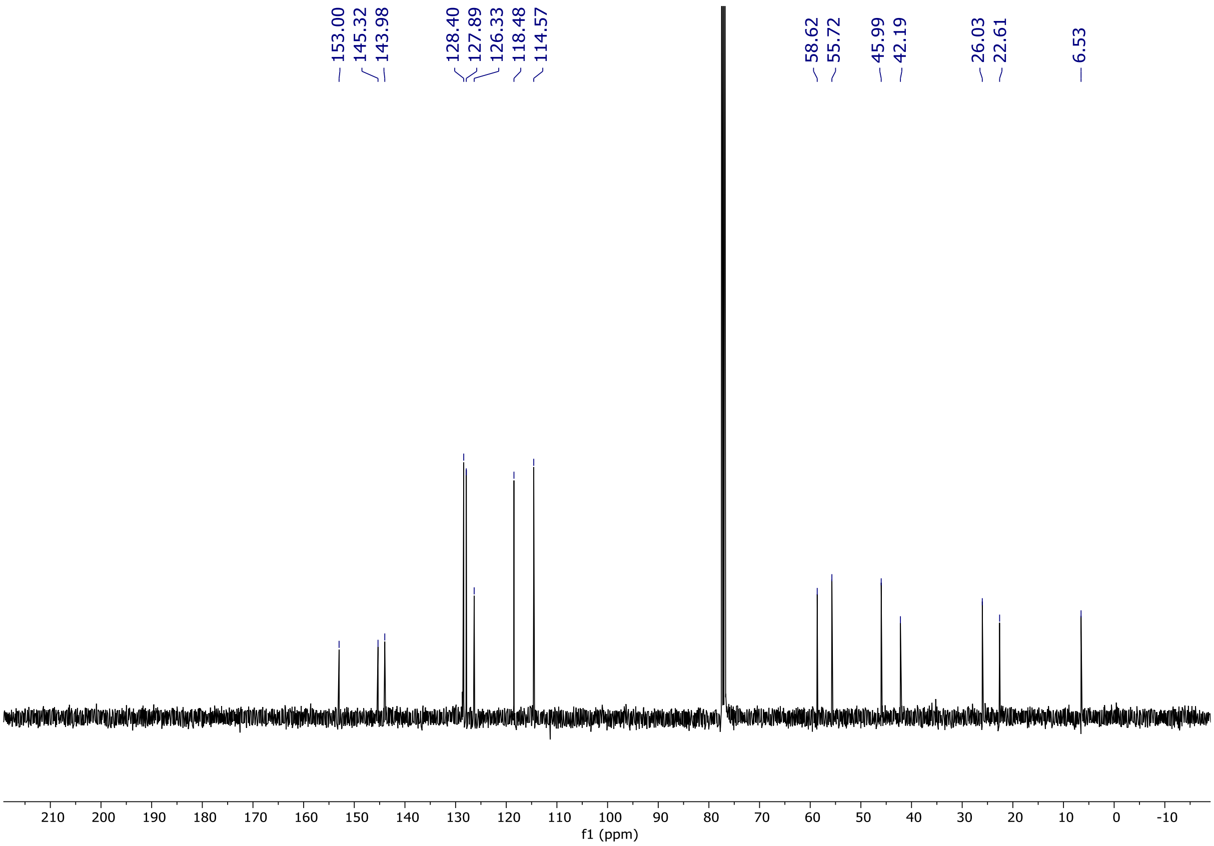

^{13}C{^{1}H} NMR (101 MHz, $CDCl_3$) spectrum of compound **26**

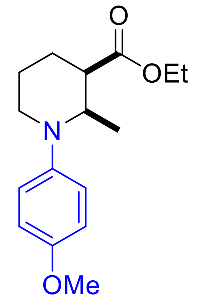

27

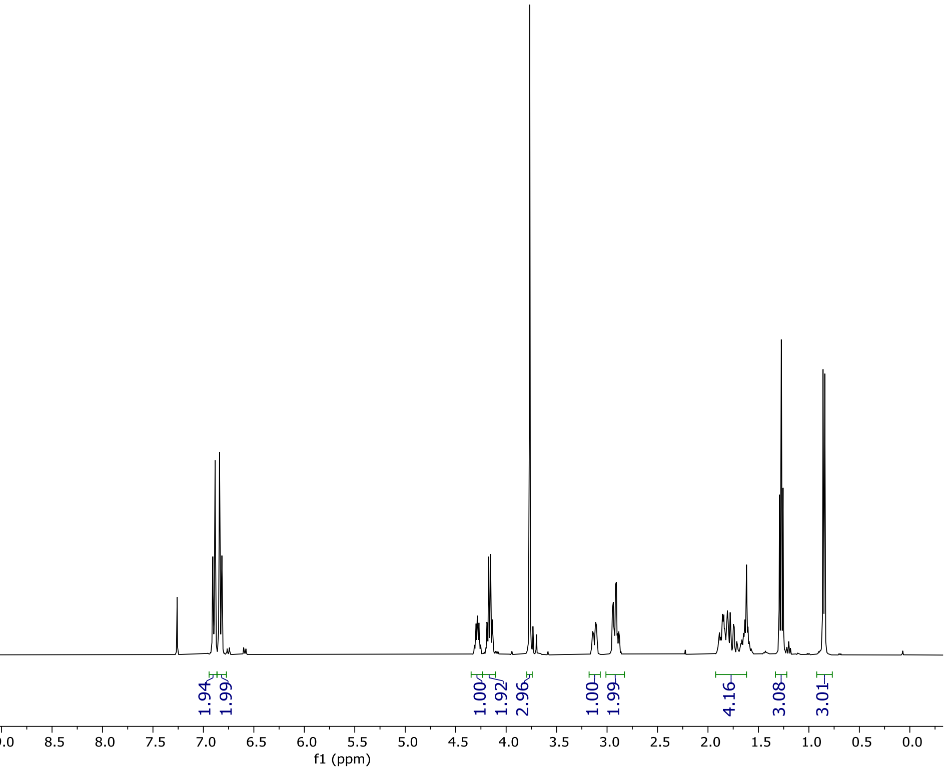

^{1}H NMR (400 MHz, $CDCl_3$) spectrum of compound **27**

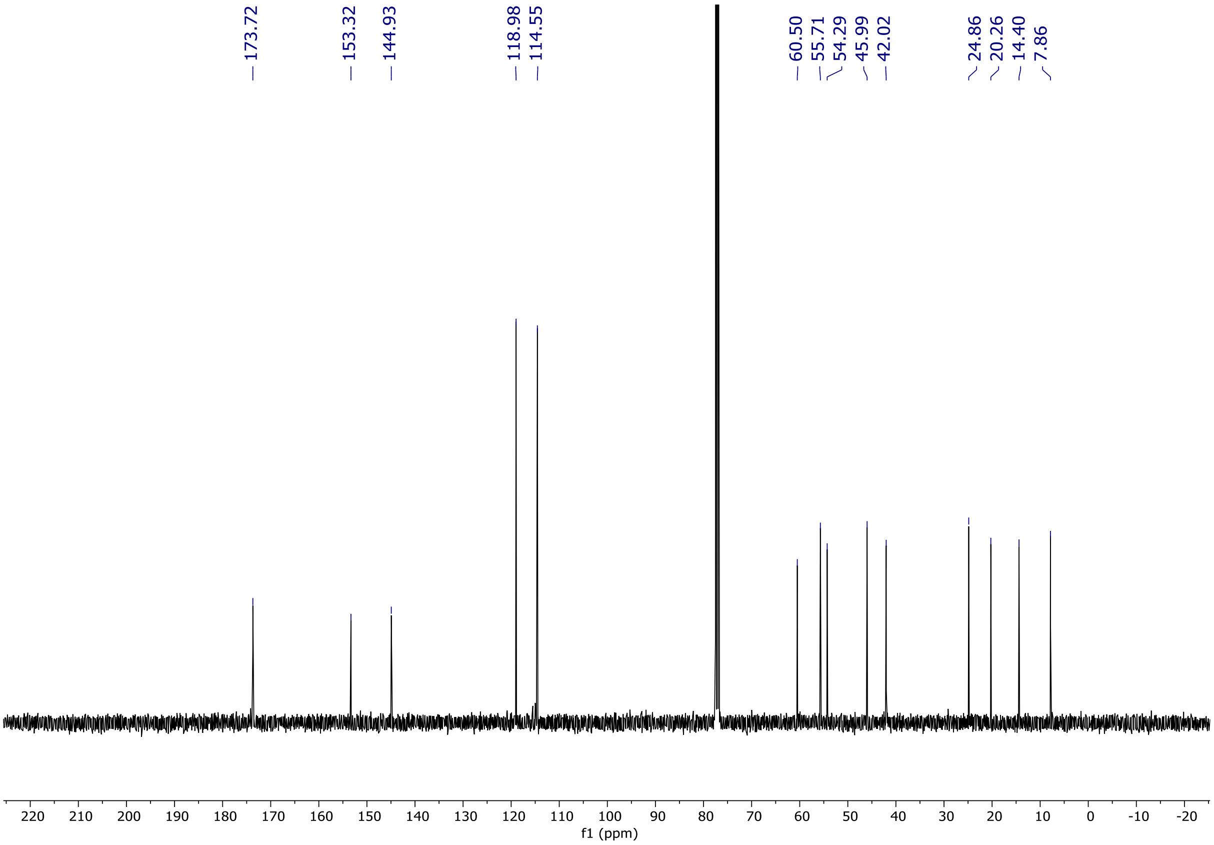

$^{13}C\{^{1}H\}$ NMR (101 MHz, $CDCl_3$) spectrum of compound **27**

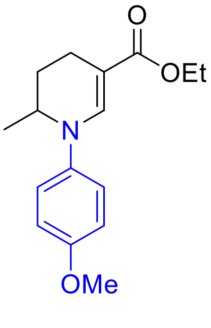

28

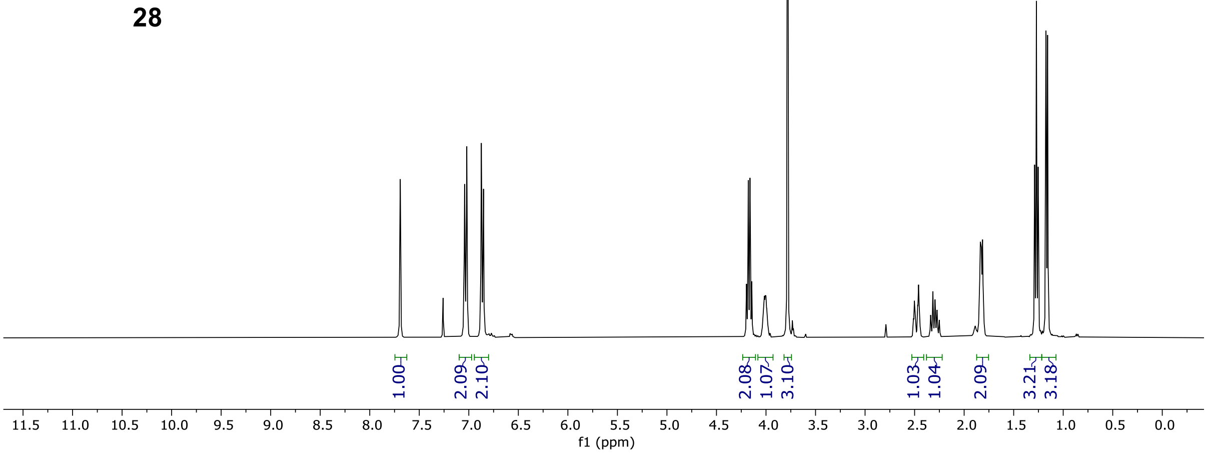

^{1}H NMR (400 MHz, $CDCl_3$) spectrum of compound **28**

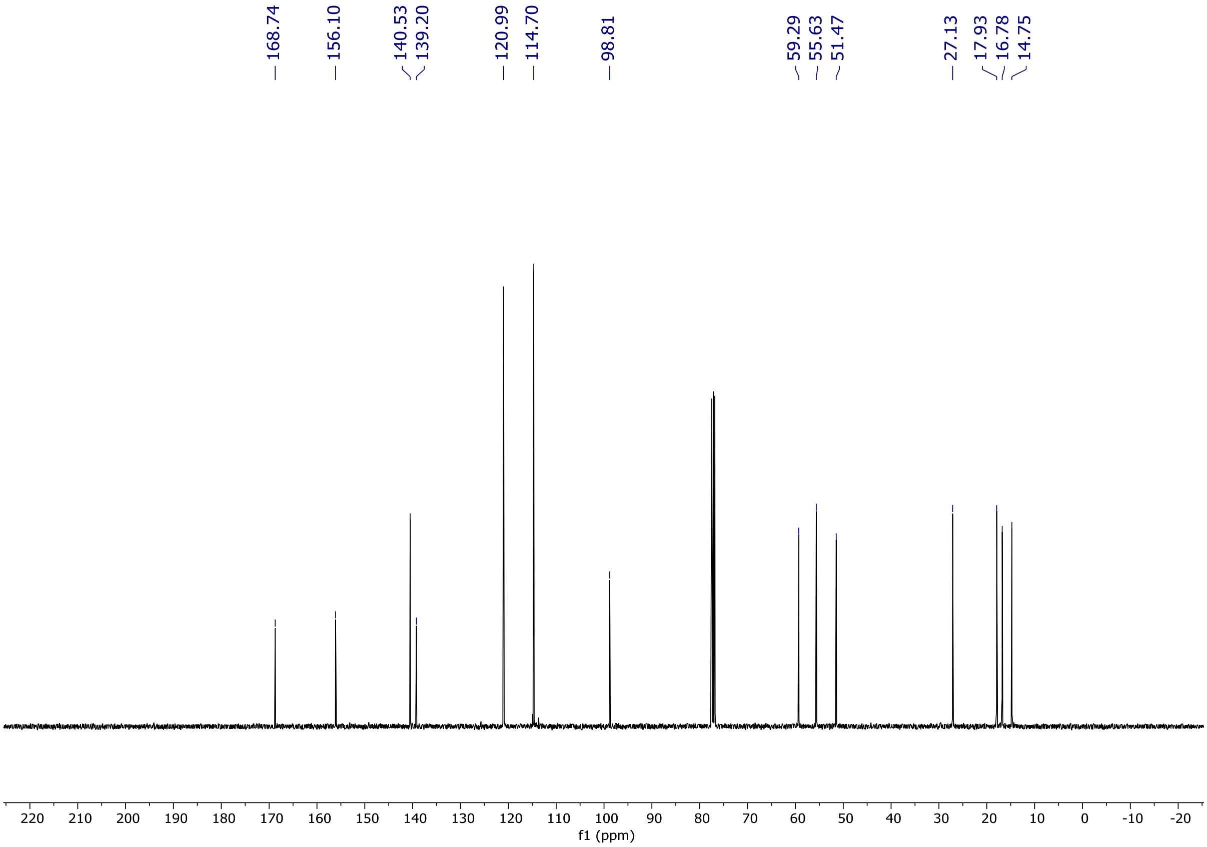

^{13}C{^{1}H} NMR (101 MHz, $CDCl_3$) spectrum of compound **28**

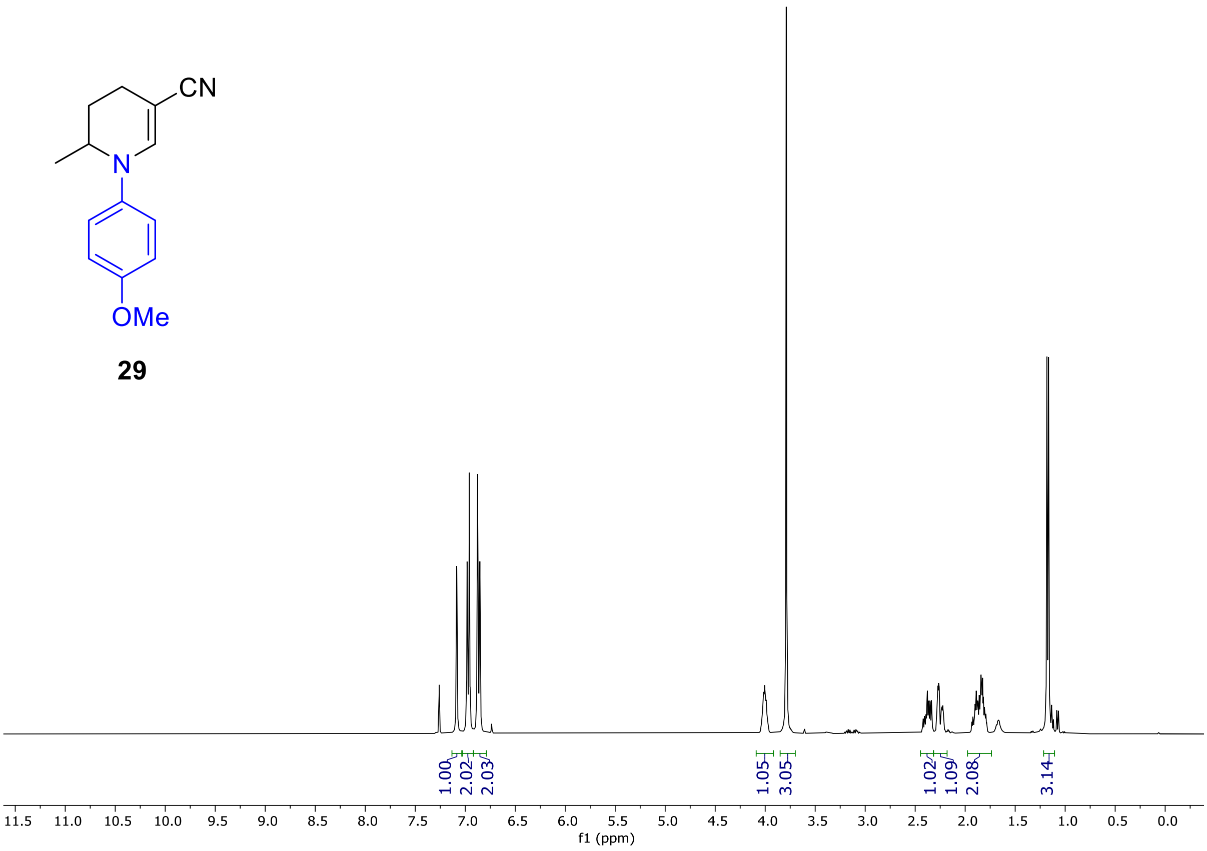

29

^{1}H NMR (400 MHz, $CDCl_3$) spectrum of compound **29**

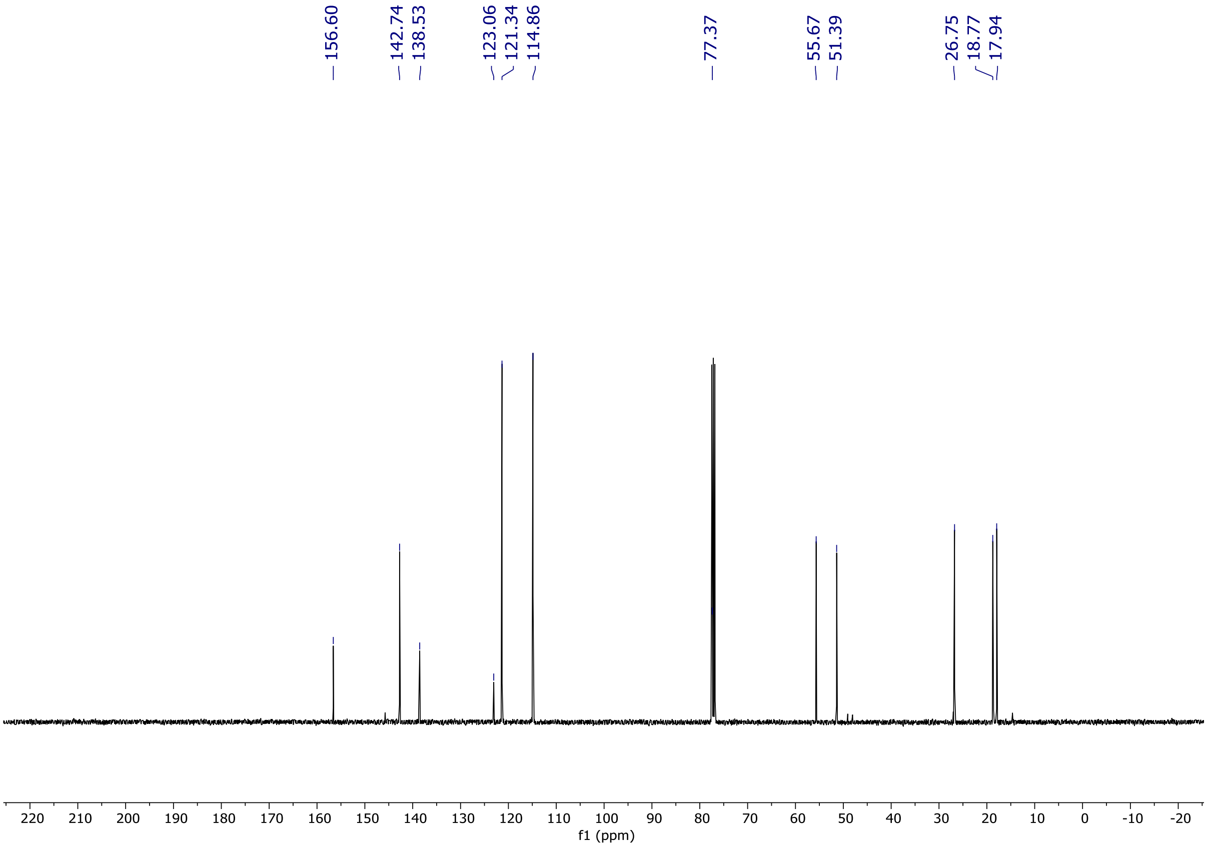

^{13}C{^{1}H} NMR (101 MHz, $CDCl_3$) spectrum of compound **29**

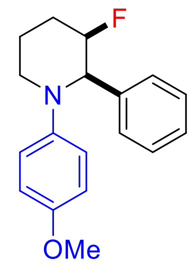

30

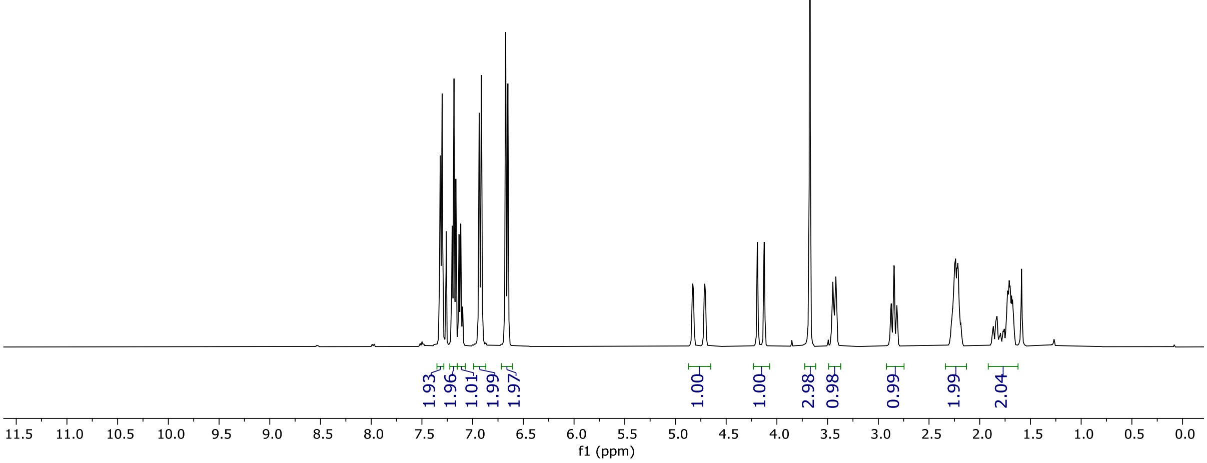

^{1}H NMR (400 MHz, $CDCl_3$) spectrum of compound **30**

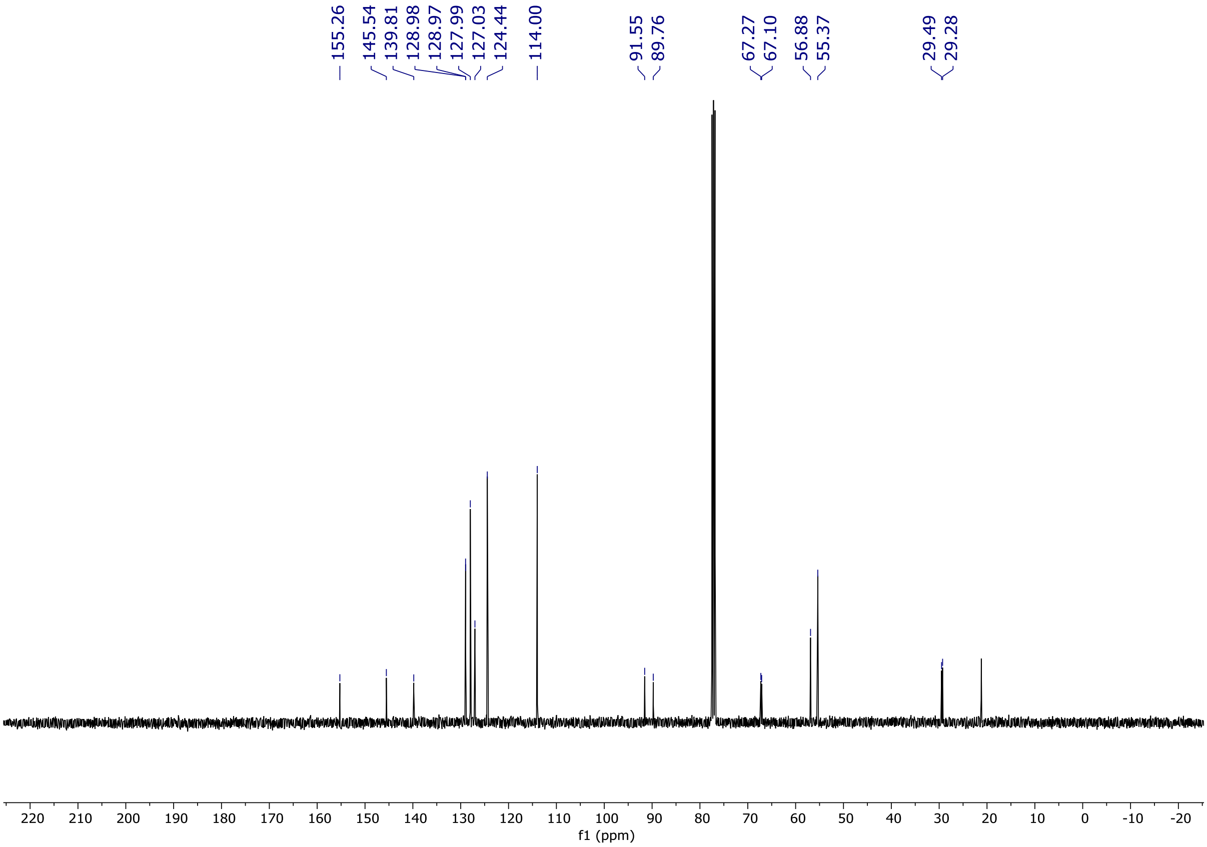

$^{13}C\{^{1}H\}$ NMR (101 MHz, $CDCl_3$) spectrum of compound **30**

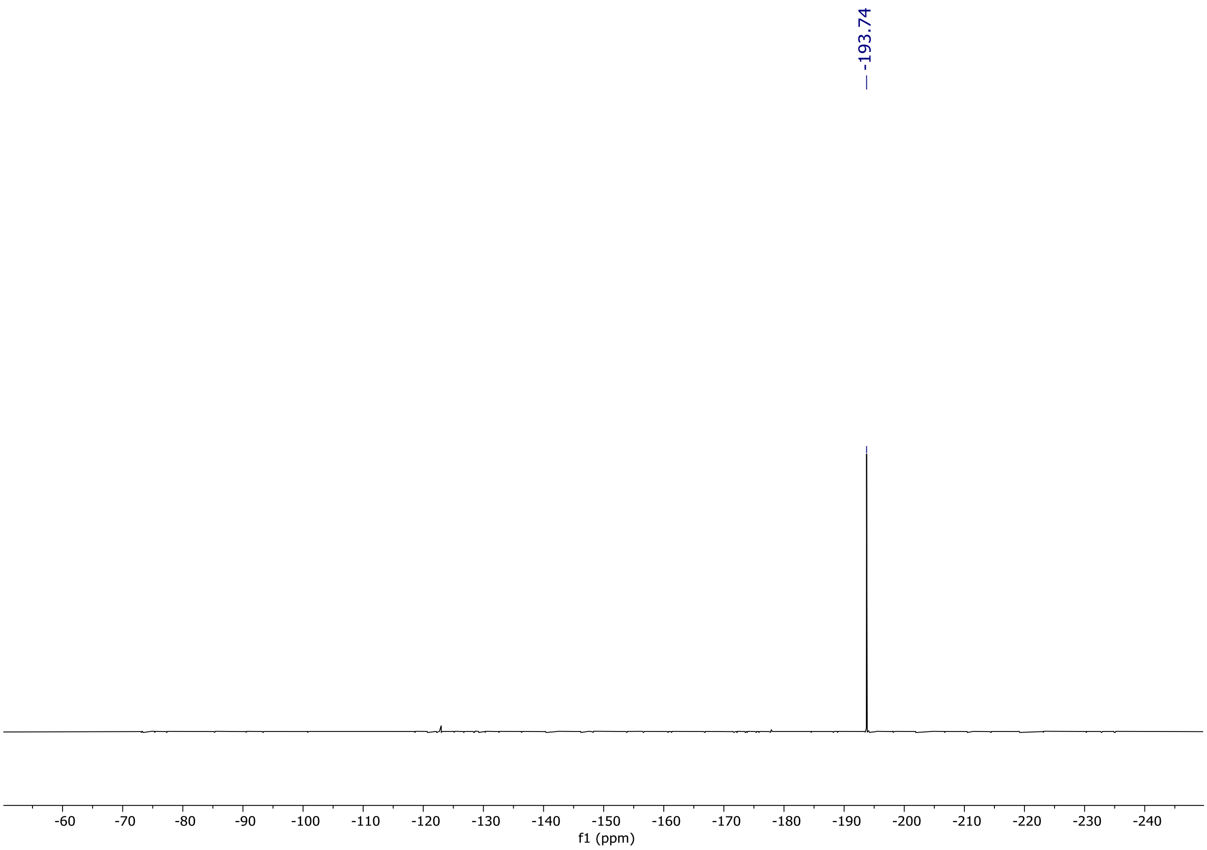

$^{19}F\{^{1}H\}$ NMR (376 MHz, $CDCl_3$) spectrum of compound **30**

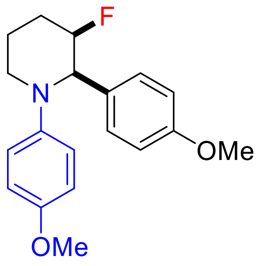

31

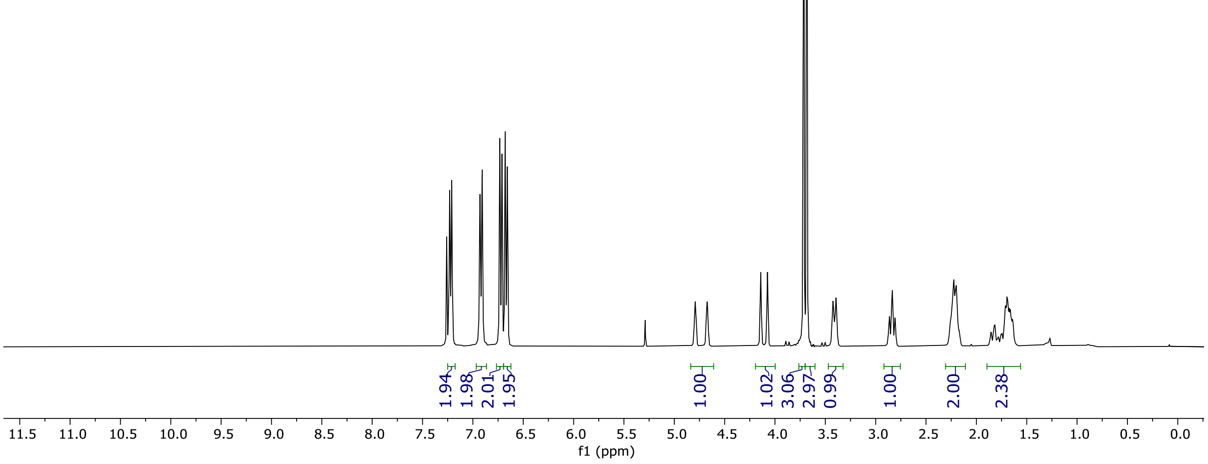

^{1}H NMR (400 MHz, $CDCl_3$) spectrum of compound **31**

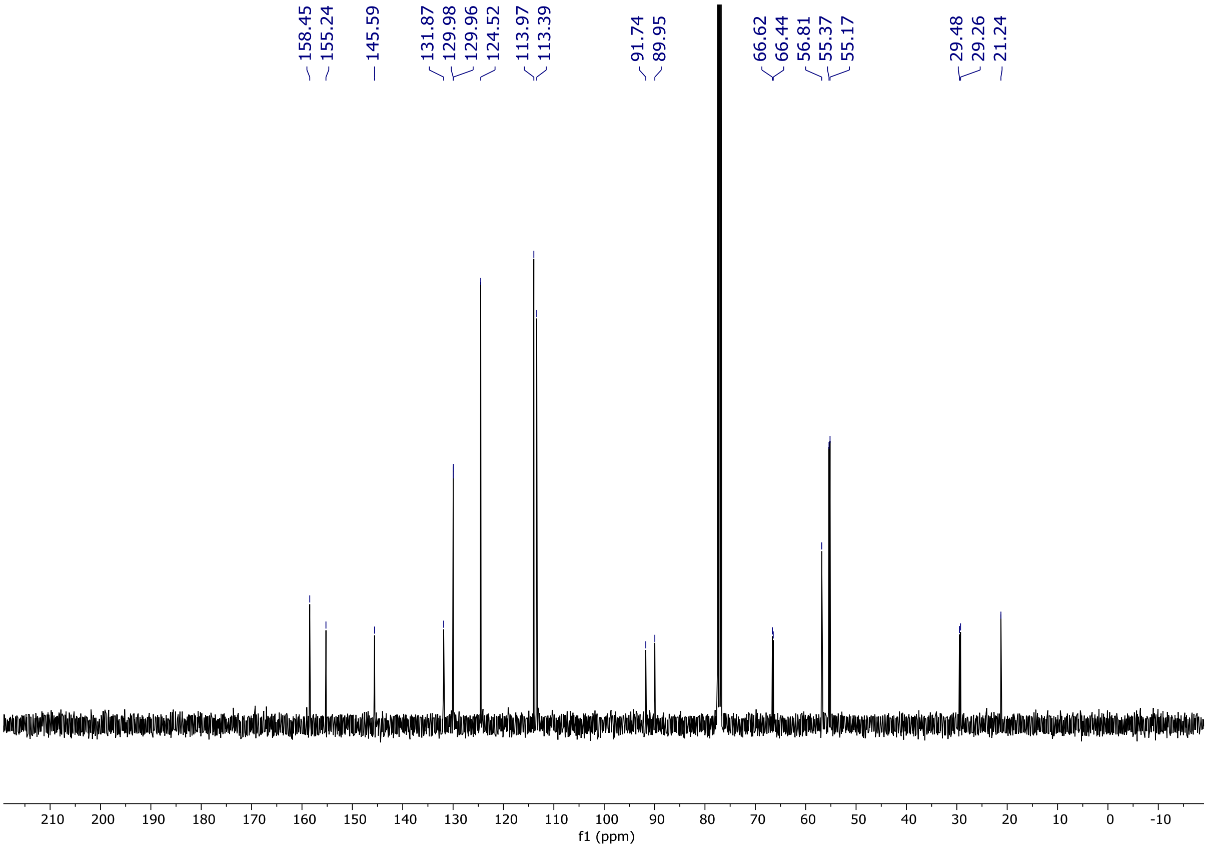

^{13}C{^{1}H} NMR (101 MHz, $CDCl_3$) spectrum of compound **31**

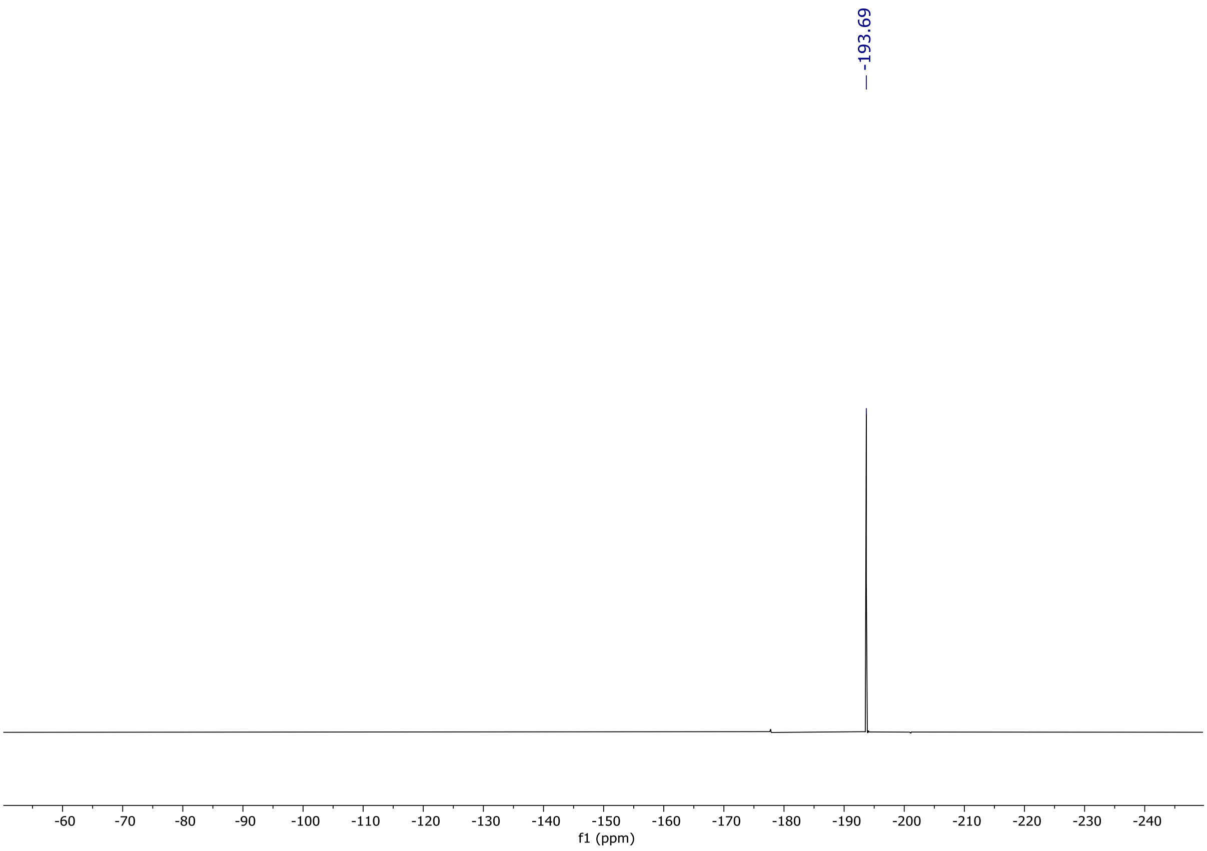

$^{19}F\{^{1}H\}$ NMR (376 MHz, $CDCl_3$) spectrum of compound **31**

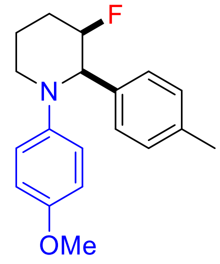

32

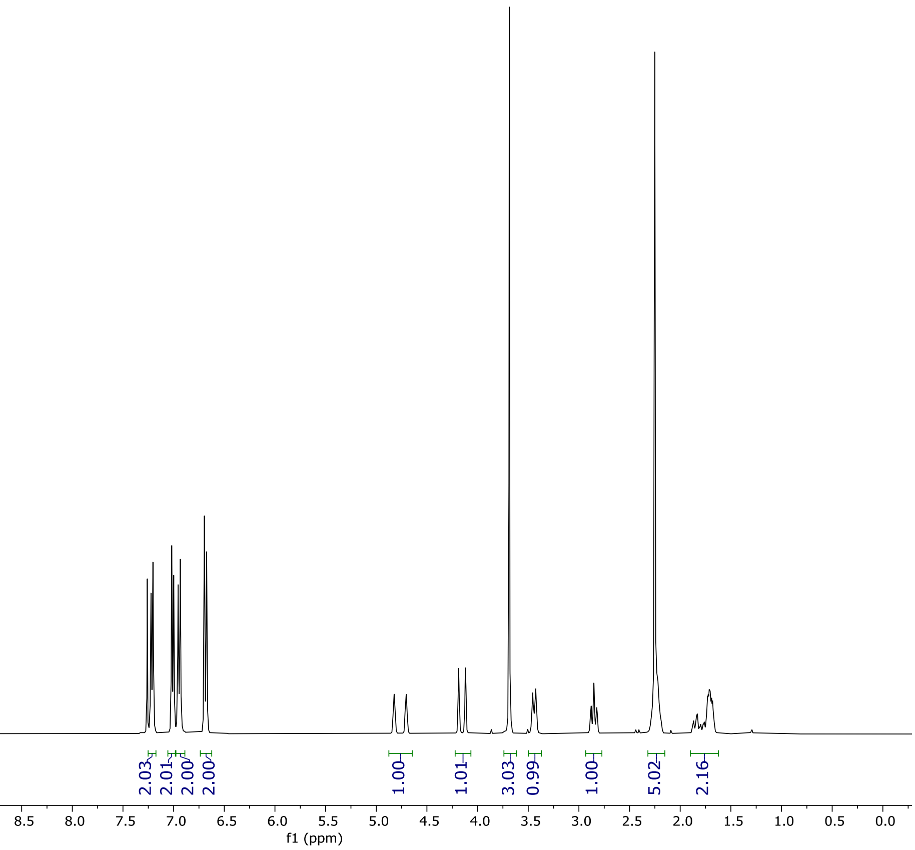

^{1}H NMR (400 MHz, $CDCl_3$) spectrum of compound **32**

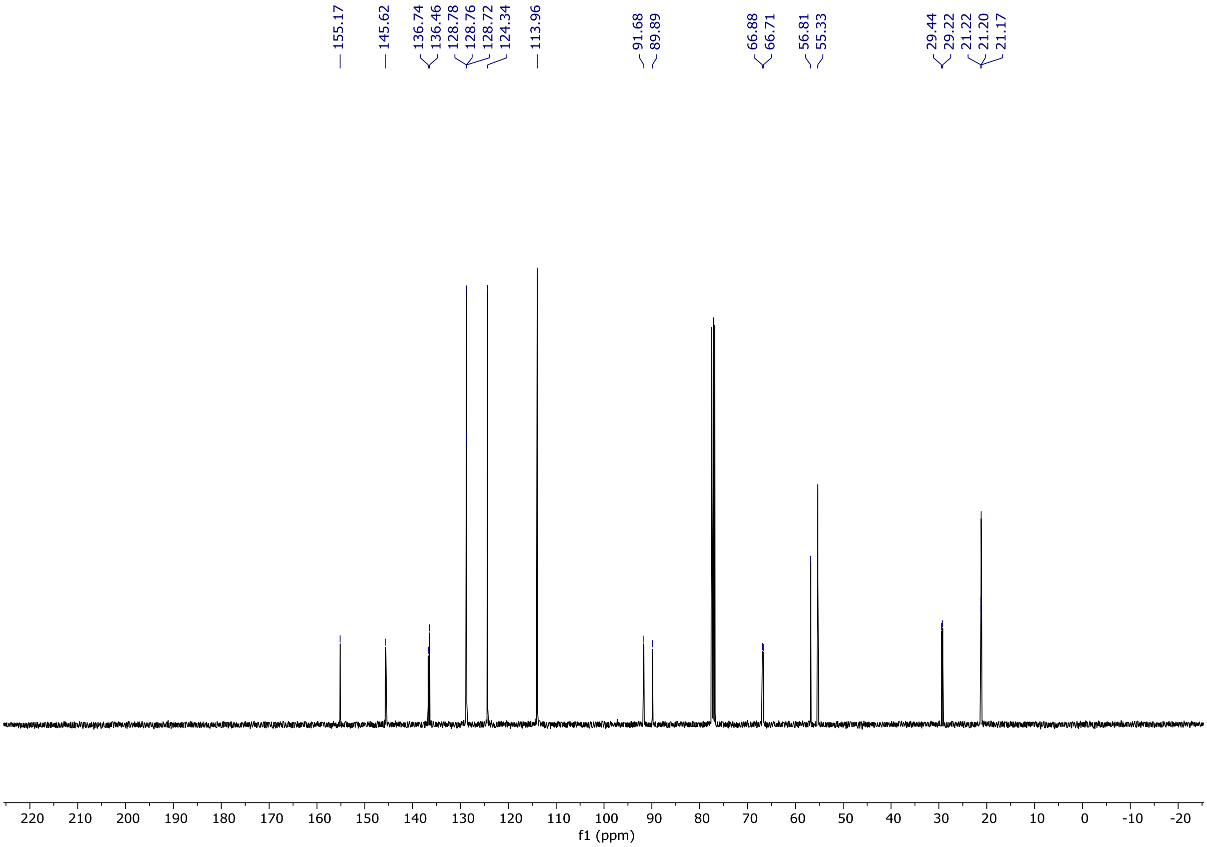

^{13}C{^{1}H} NMR (101 MHz, $CDCl_3$) spectrum of compound **32**

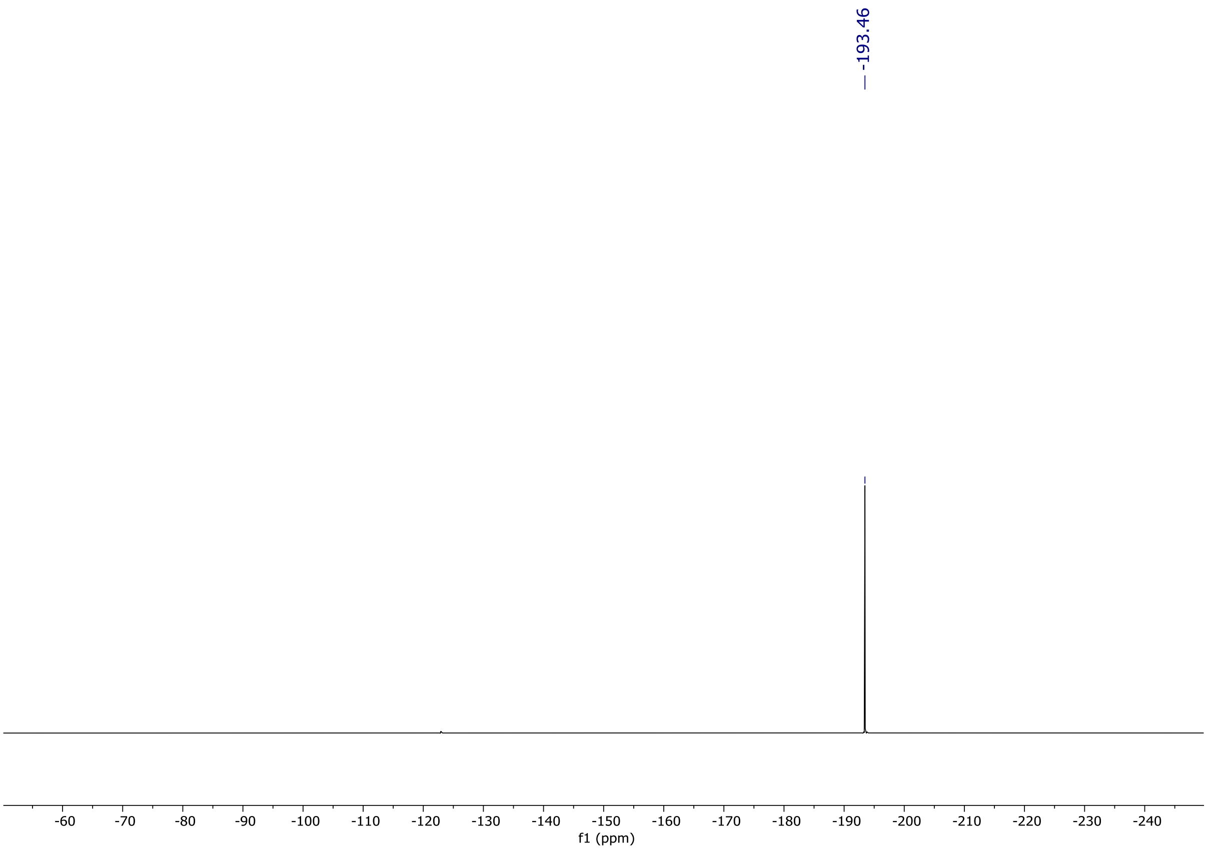

$^{19}F\{^{1}H\}$ NMR (376 MHz, $CDCl_3$) spectrum of compound **32**

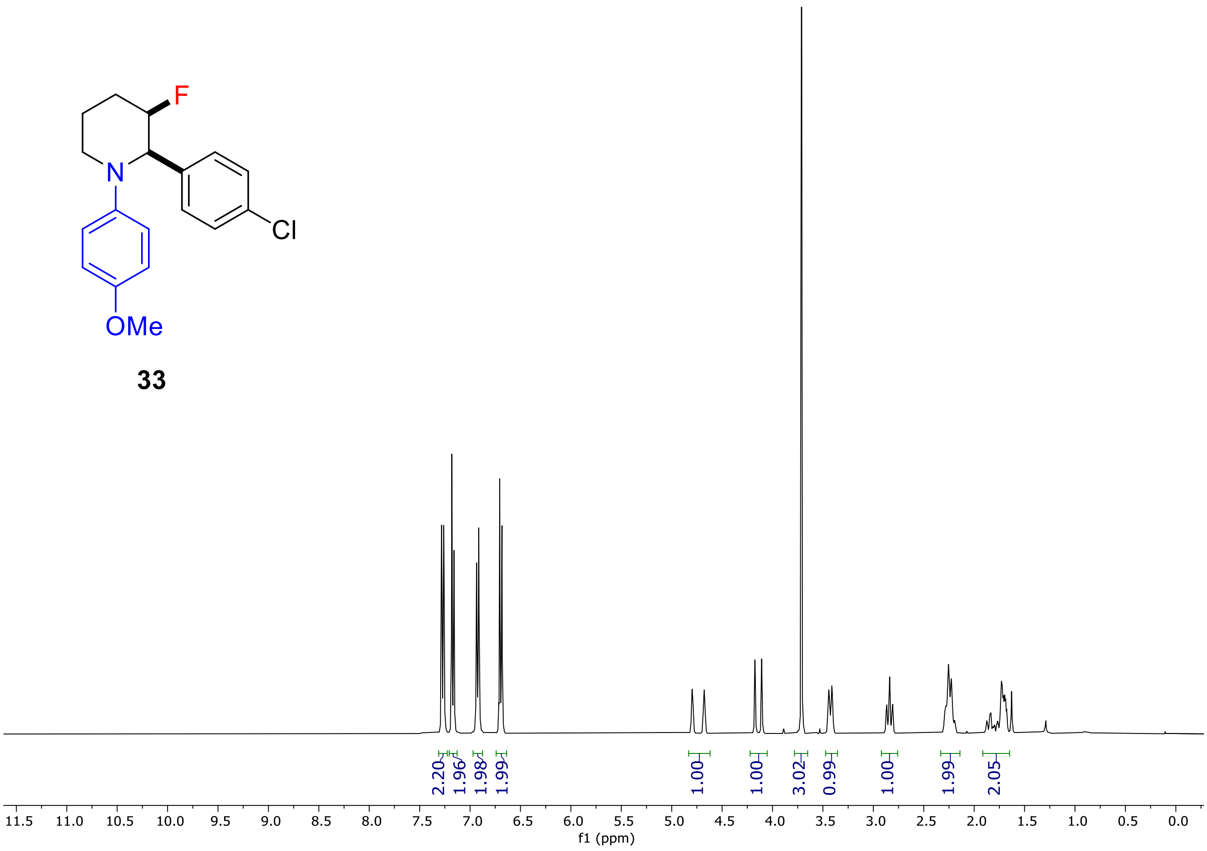

^{1}H NMR (400 MHz, $CDCl_3$) spectrum of compound **33**

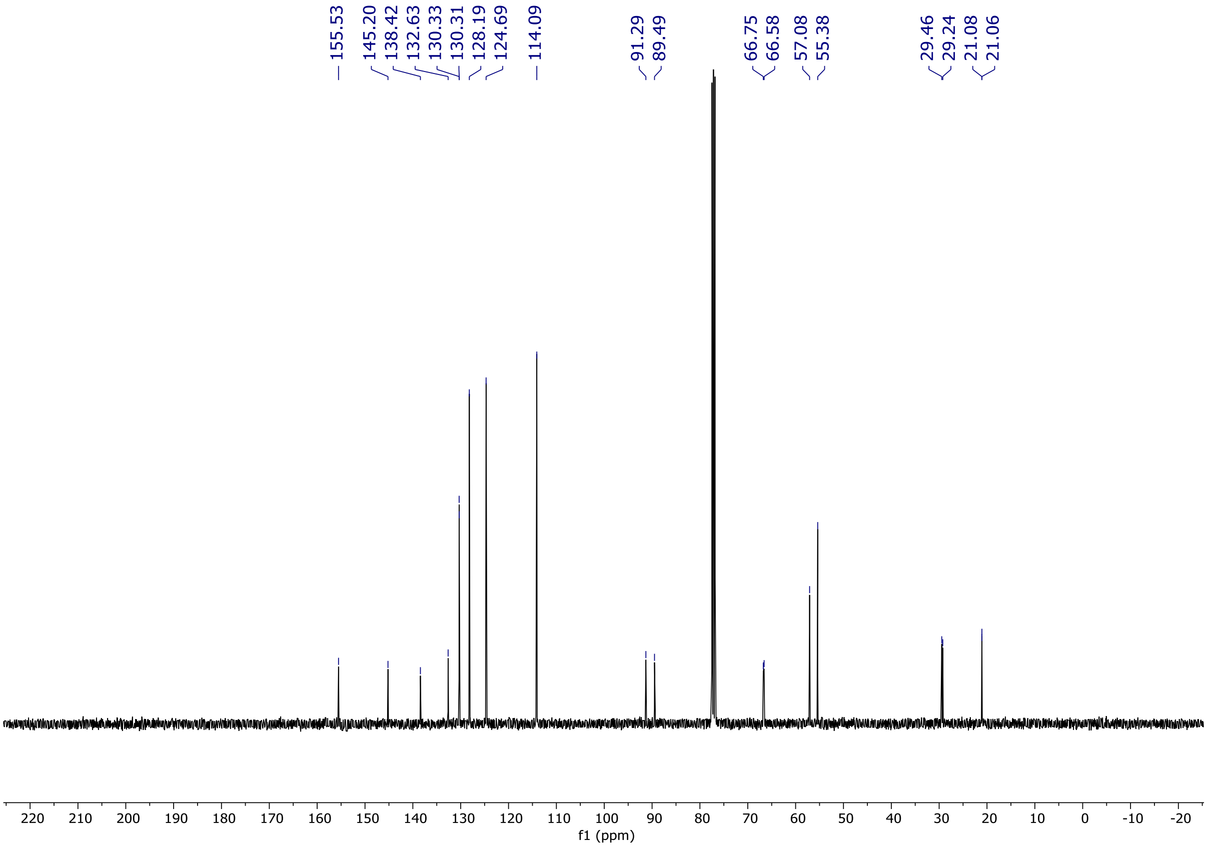

^{13}C{^{1}H} NMR (101 MHz, $CDCl_3$) spectrum of compound **33**

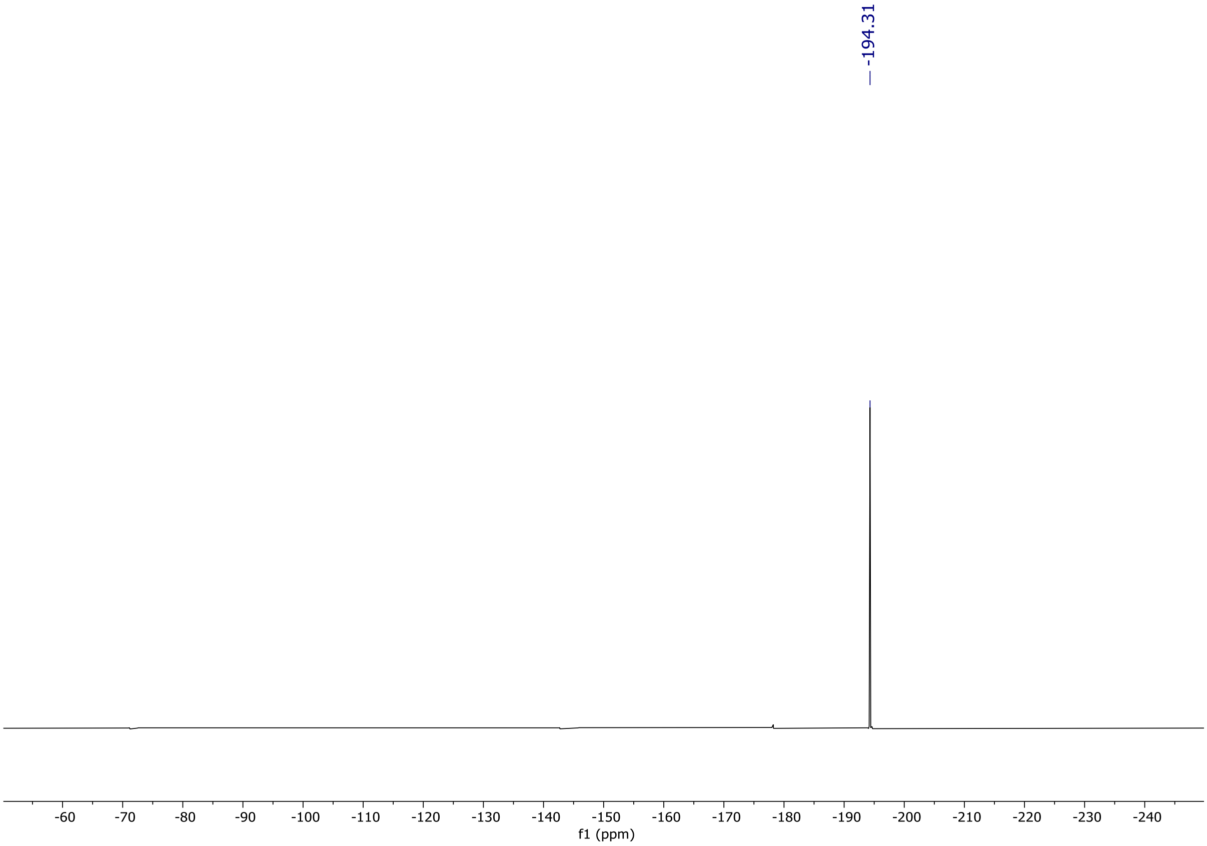

$^{19}F\{^{1}H\}$ NMR (376 MHz, $CDCl_3$) spectrum of compound **33**

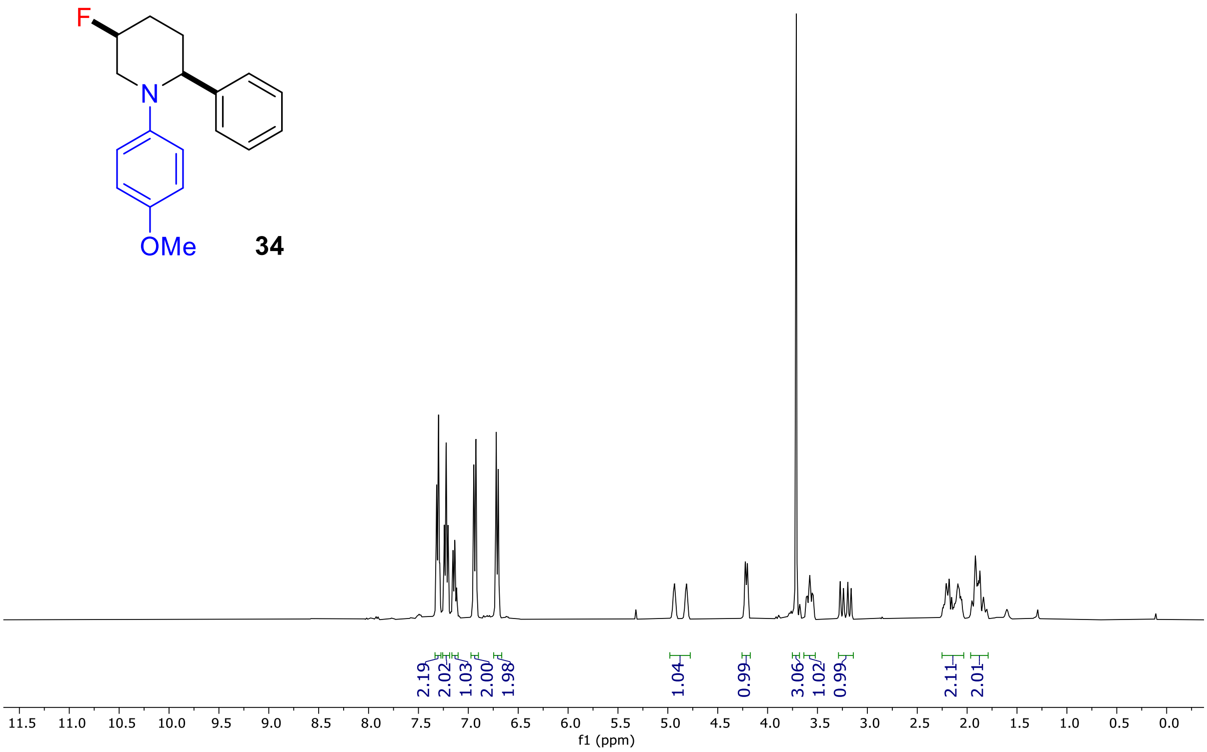

^{1}H NMR (400 MHz, $CDCl_3$) spectrum of compound **34**

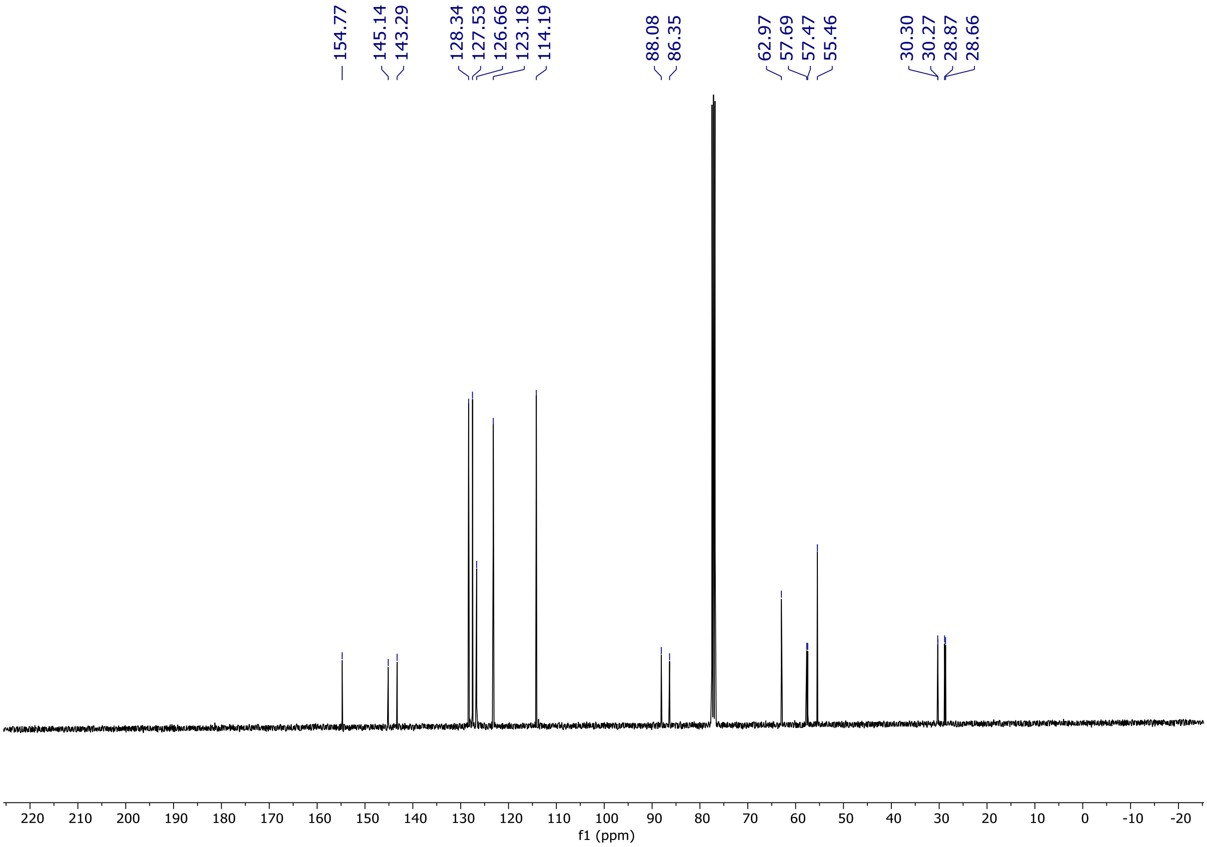

$^{13}C\{^{1}H\}$ NMR (101 MHz, $CDCl_3$) spectrum of compound **34**

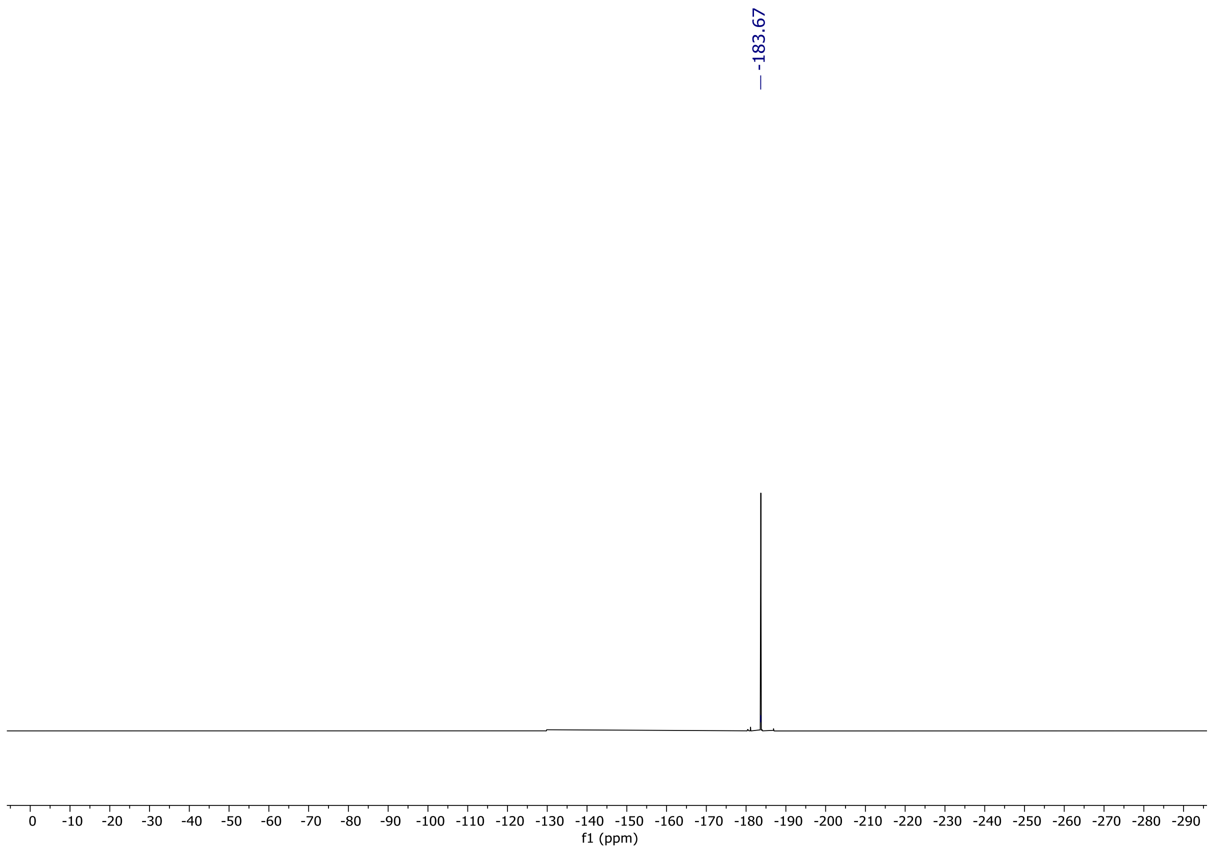

$^{19}F\{^{1}H\}$ NMR (376 MHz, $CDCl_3$) spectrum of compound **34**

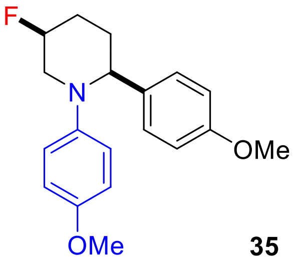

35

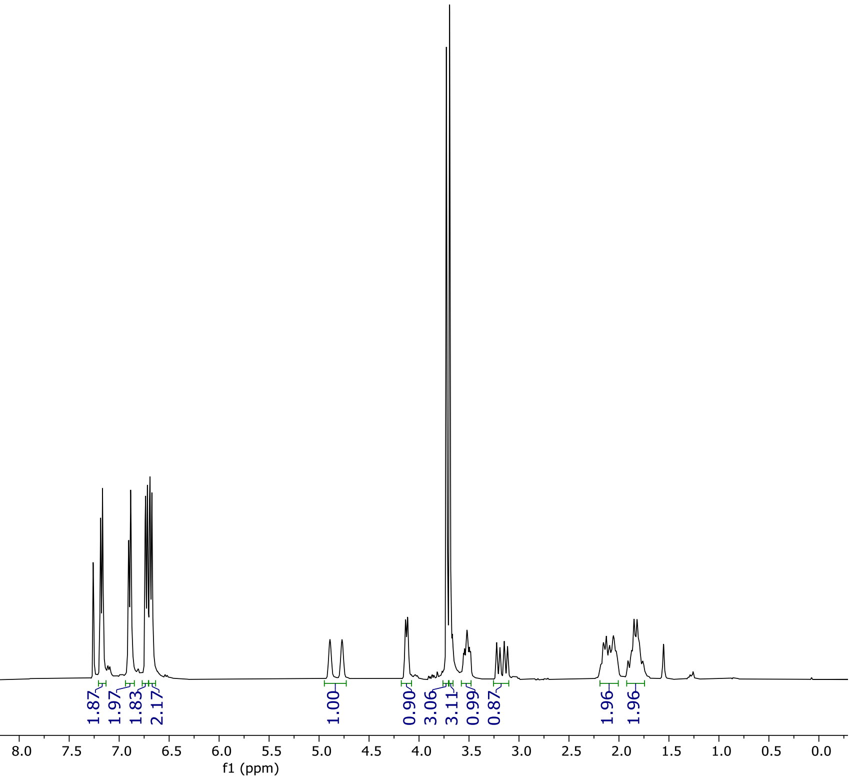

^{1}H NMR (400 MHz, $CDCl_3$) spectrum of compound **35**

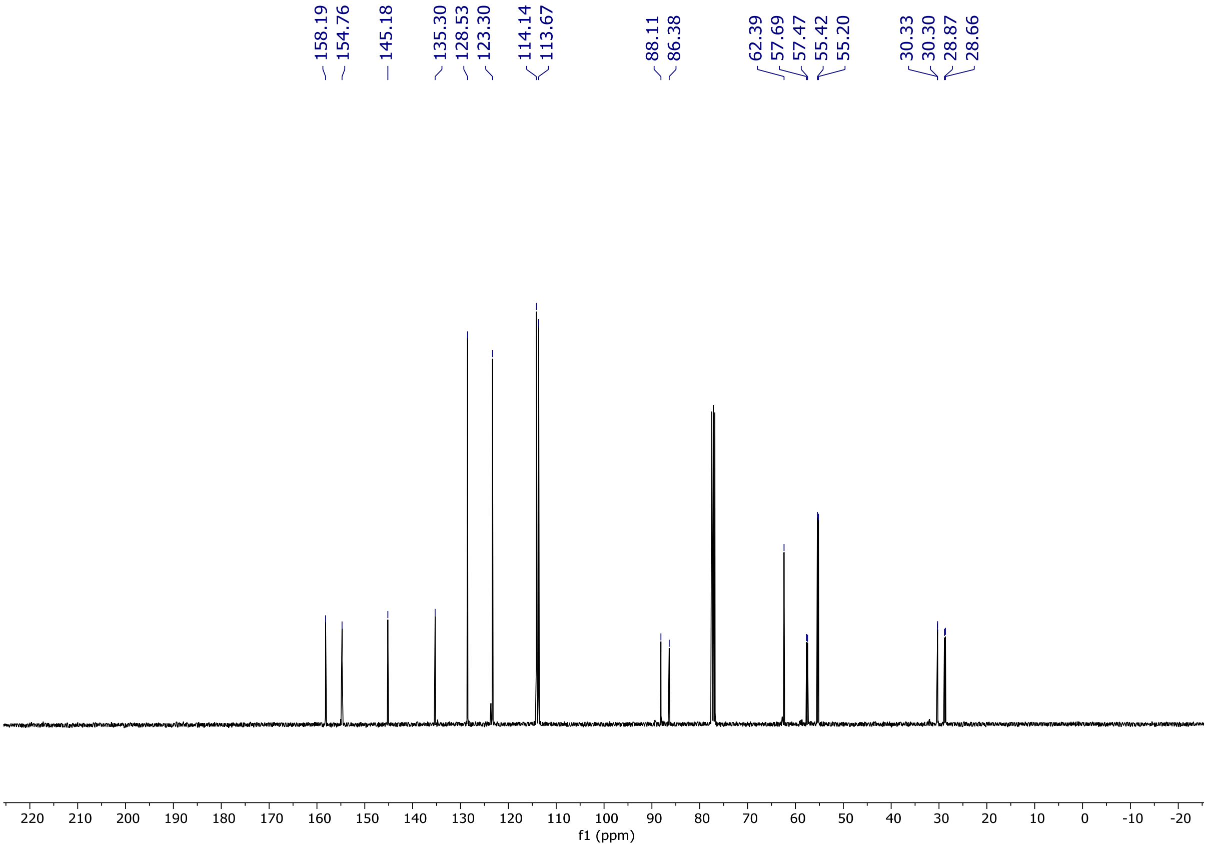

^{13}C{^{1}H} NMR (101 MHz, $CDCl_3$) spectrum of compound **35**

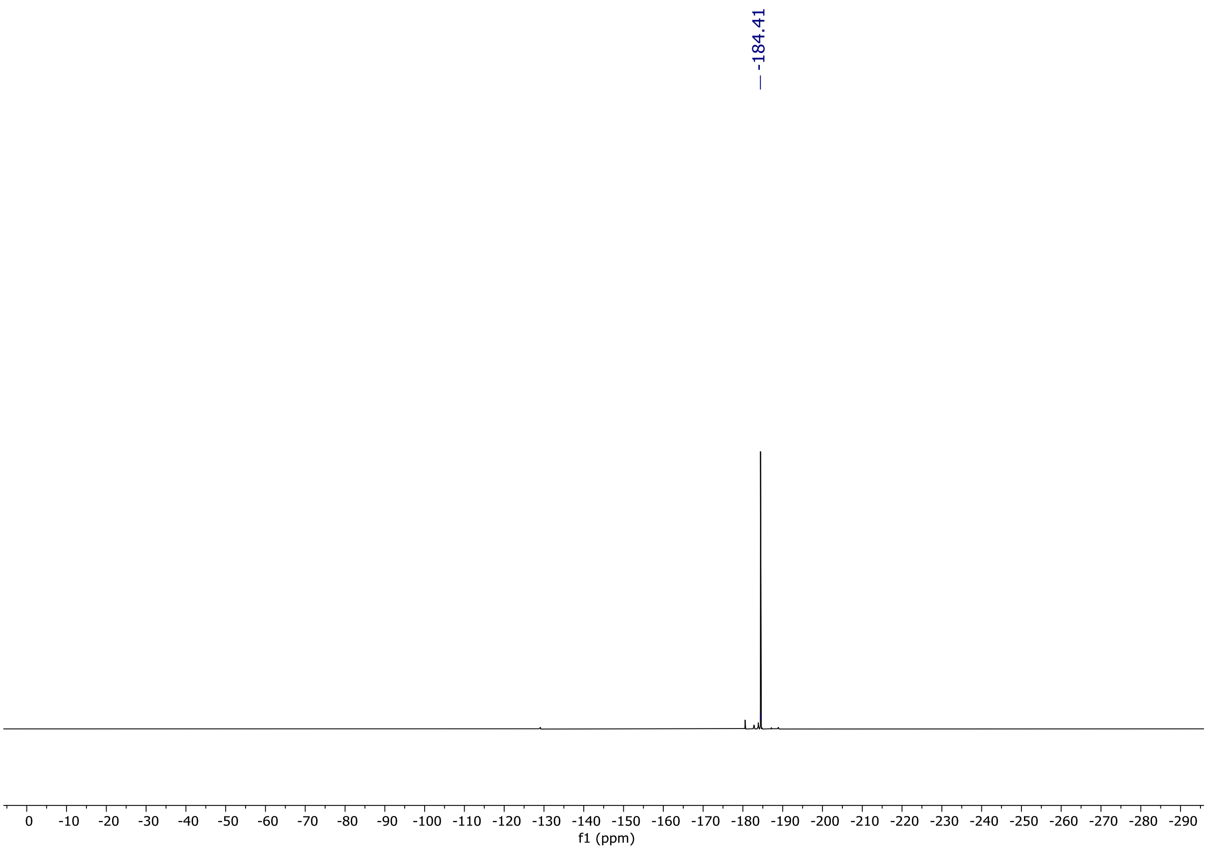

$^{19}F\{^{1}H\}$ NMR (376 MHz, $CDCl_3$) spectrum of compound **35**

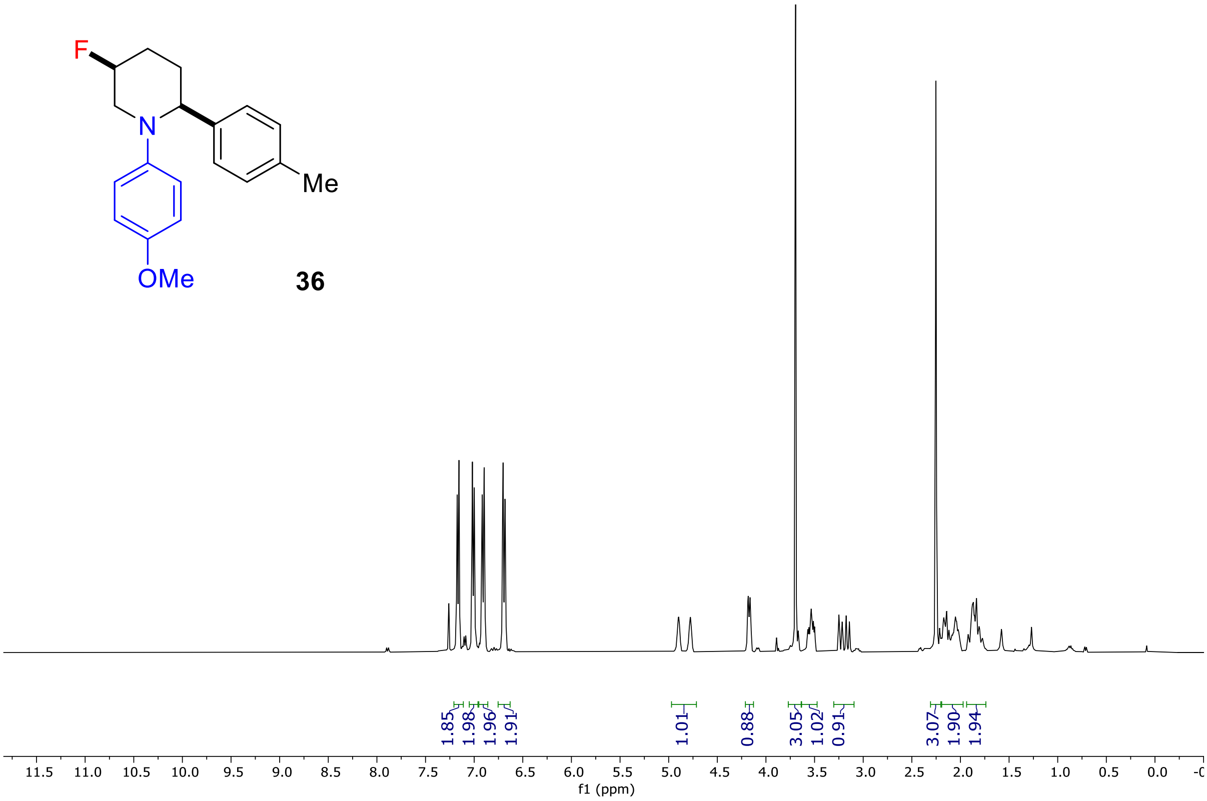

^{1}H NMR (400 MHz, $CDCl_3$) spectrum of compound **36**

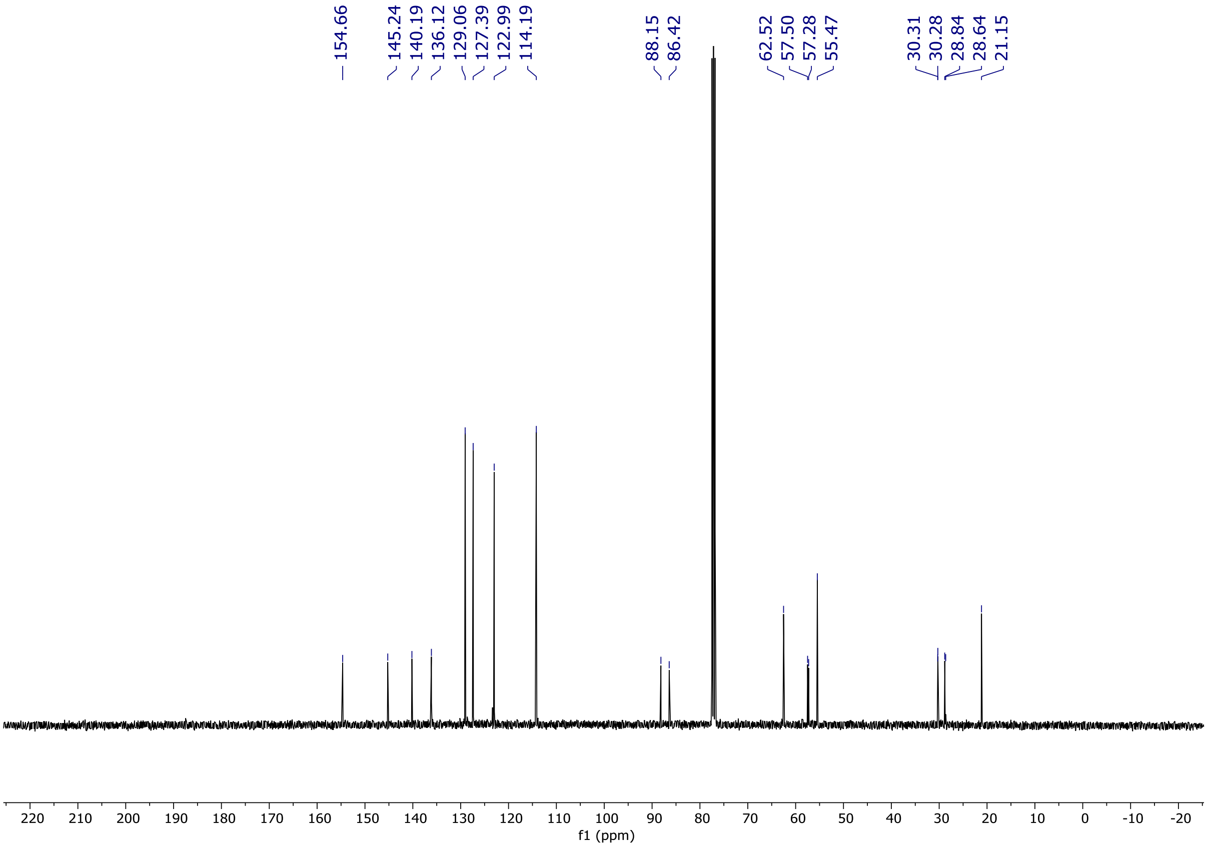

$^{13}C\{^{1}H\}$ NMR (101 MHz, $CDCl_3$) spectrum of compound **36**

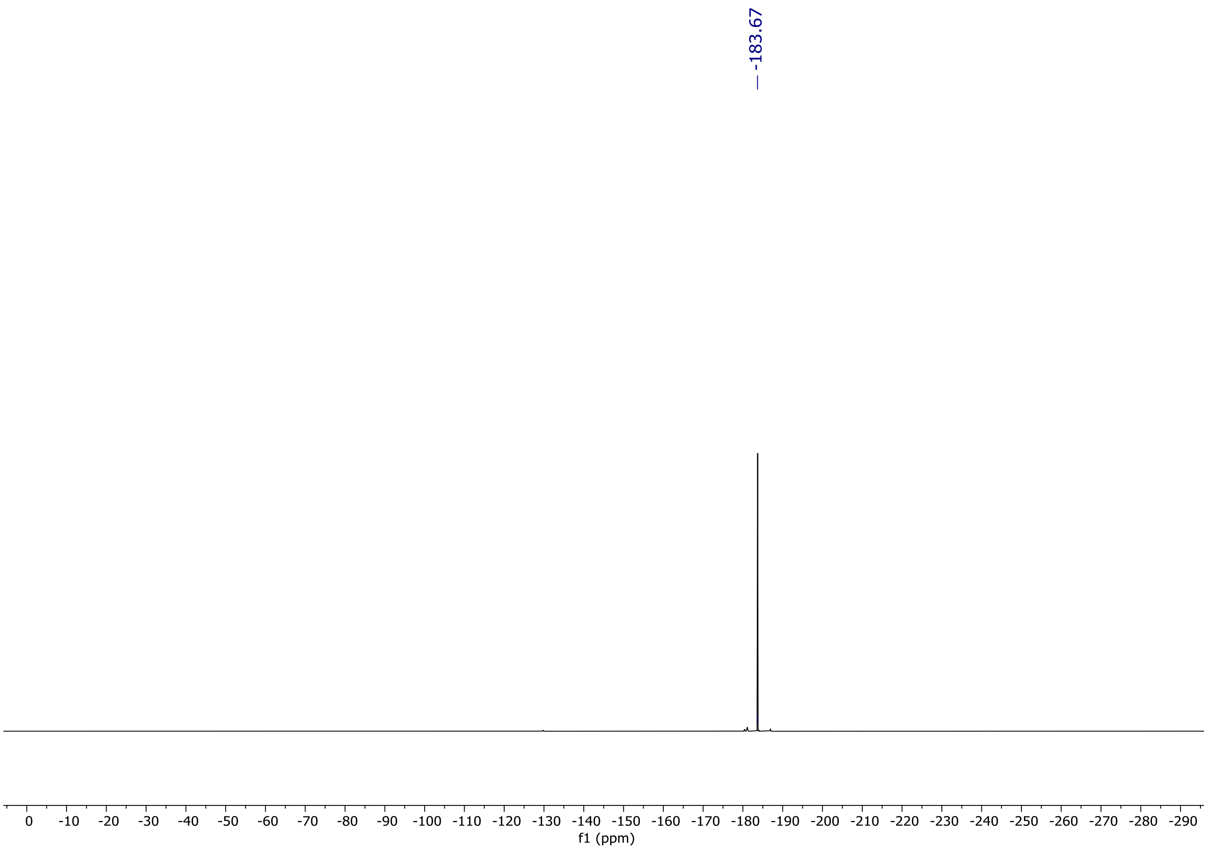

$^{19}F\{^{1}H\}$ NMR (376 MHz, $CDCl_3$) spectrum of compound **36**

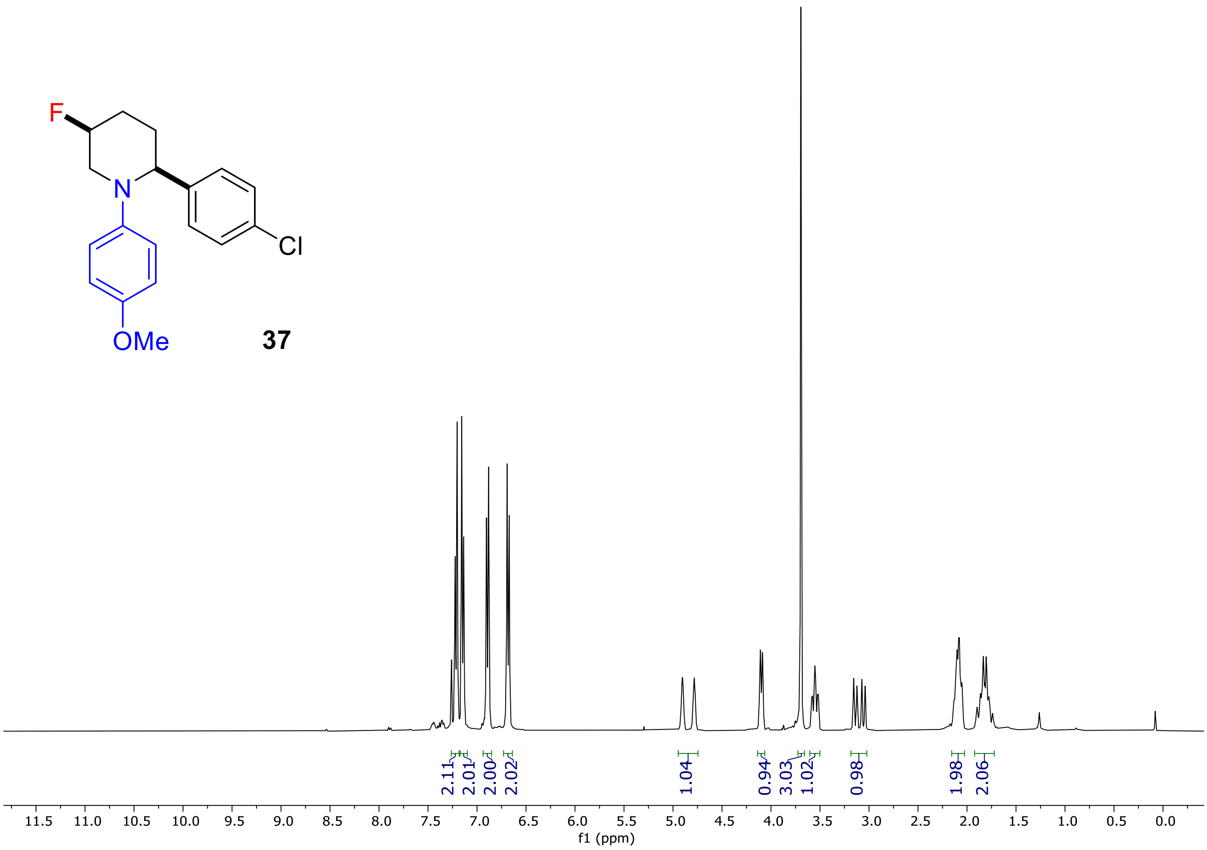

^{1}H NMR (400 MHz, $CDCl_3$) spectrum of compound **37**

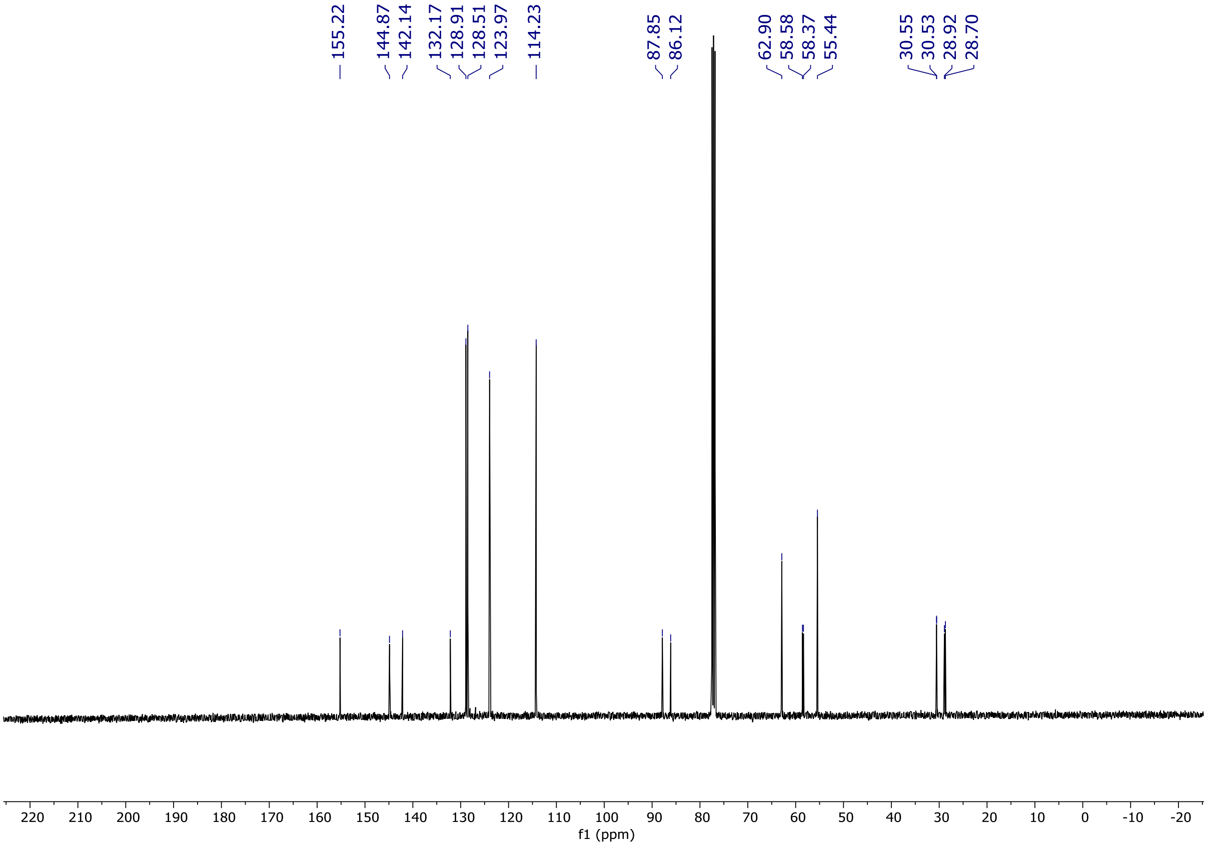

^{13}C{^{1}H} NMR (101 MHz, $CDCl_3$) spectrum of compound **37**

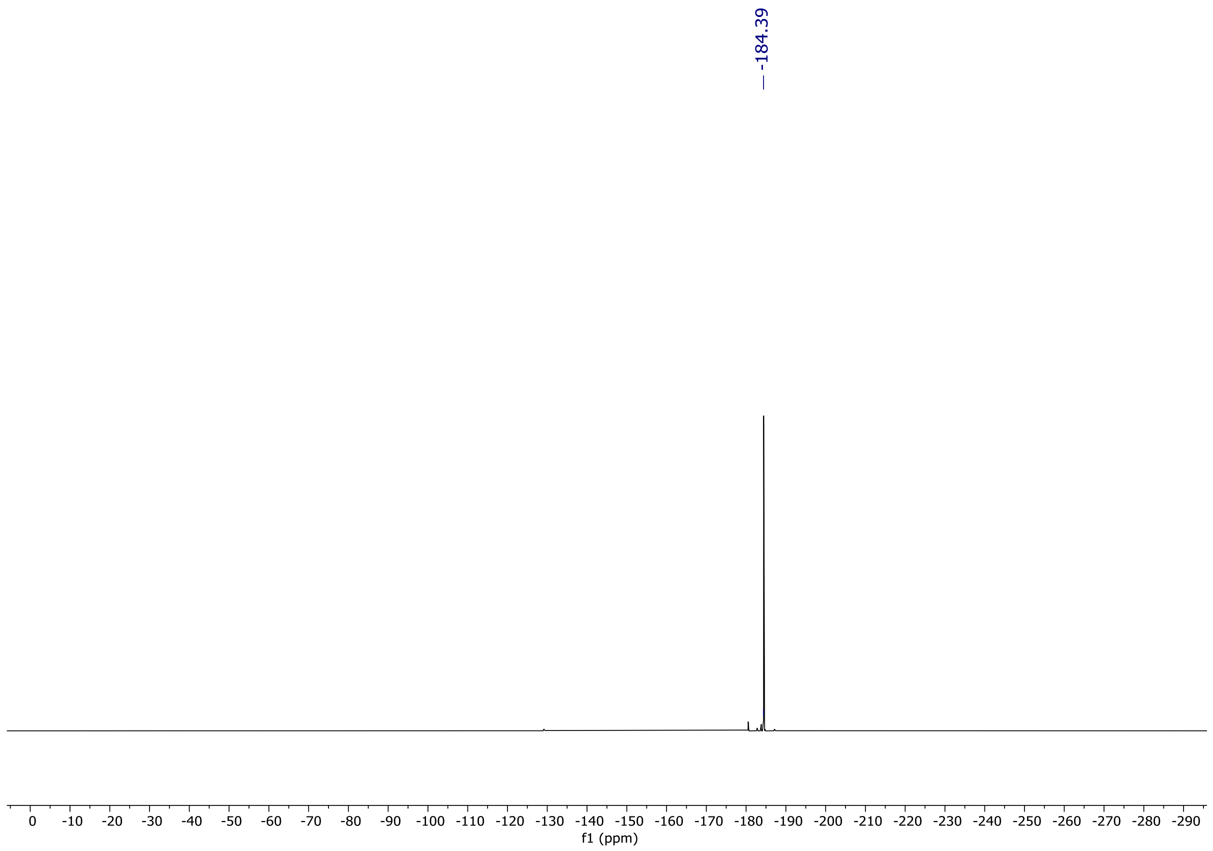

$^{19}F\{^{1}H\}$ NMR (376 MHz, $CDCl_3$) spectrum of compound **37**

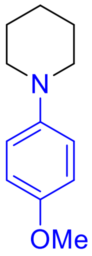

38

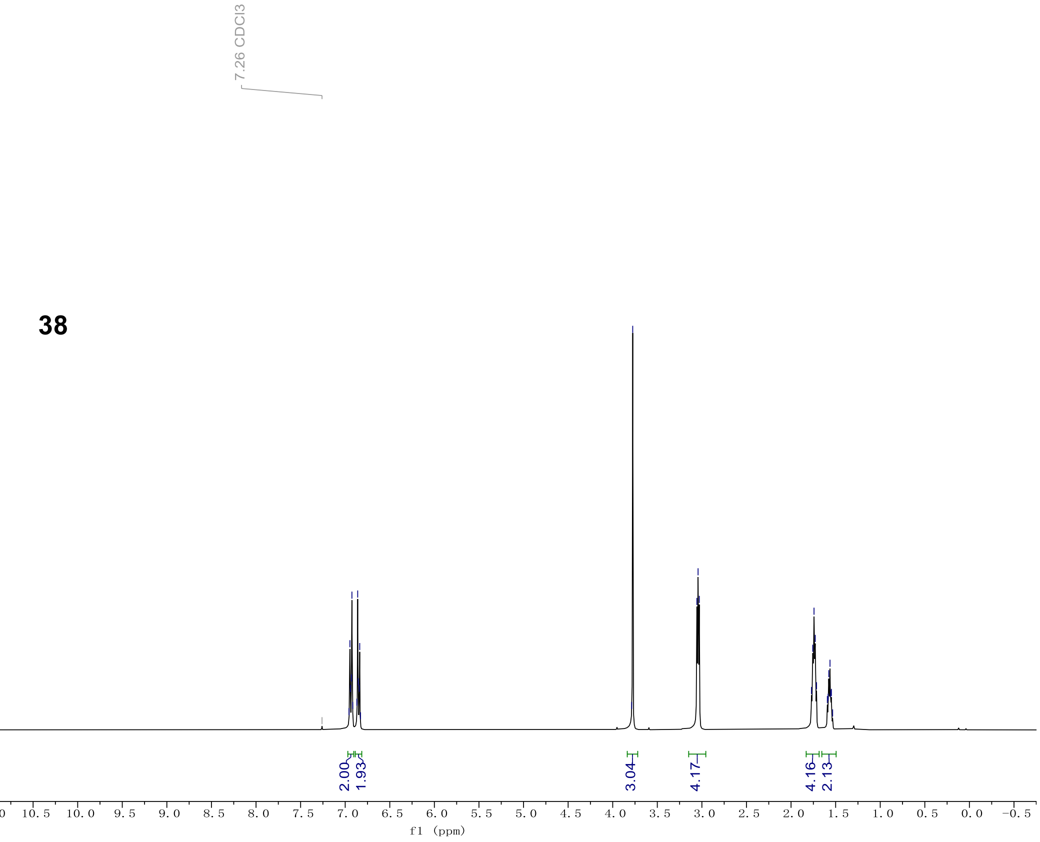

^{1}H NMR (400 MHz, $CDCl_3$) spectrum of compound **38**

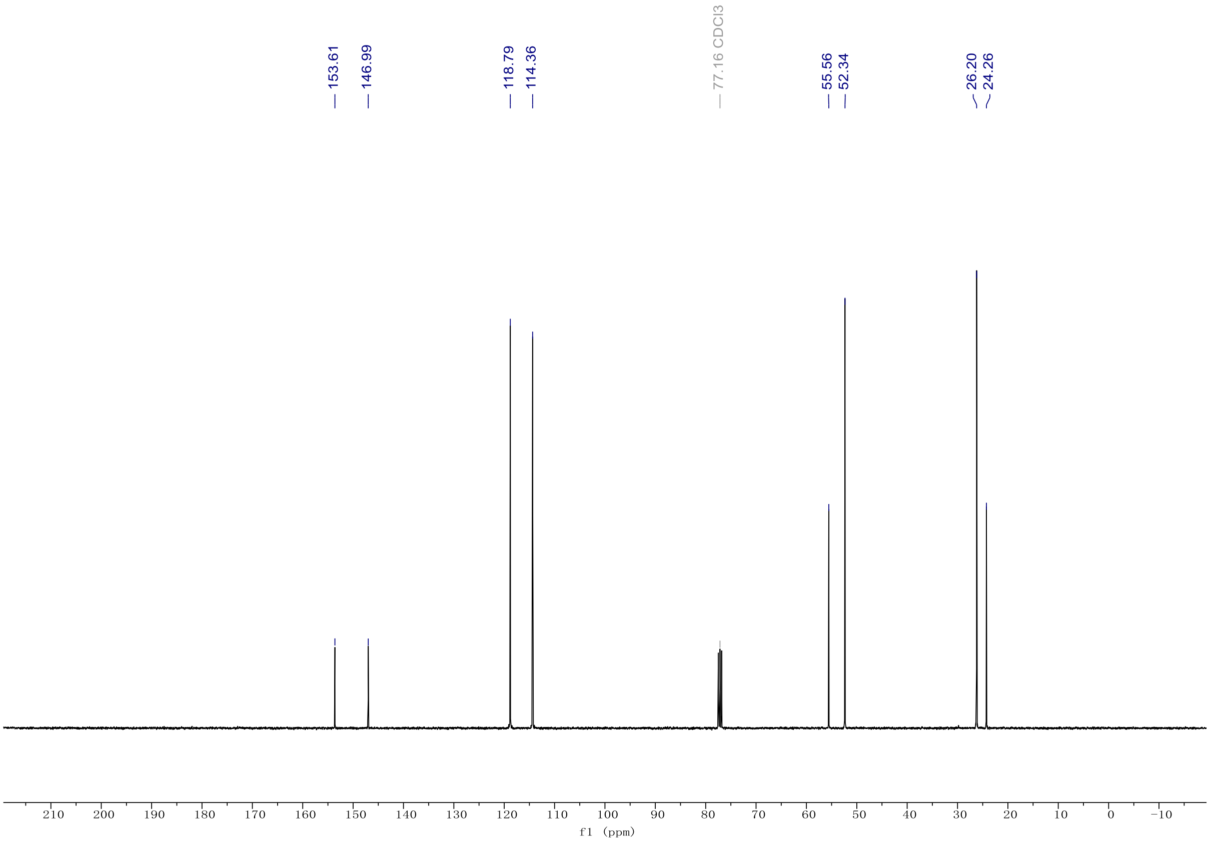

^{13}C{^{1}H} NMR (101 MHz, $CDCl_3$) spectrum of compound **38**

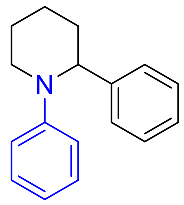

39

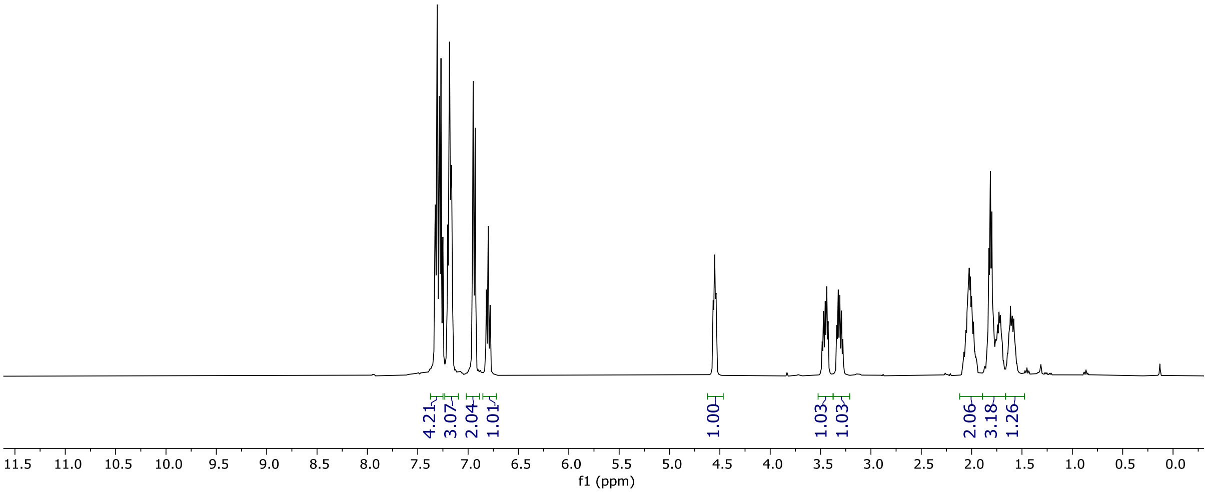

^{1}H NMR (400 MHz, CDCl$_3$) spectrum of compound **39**

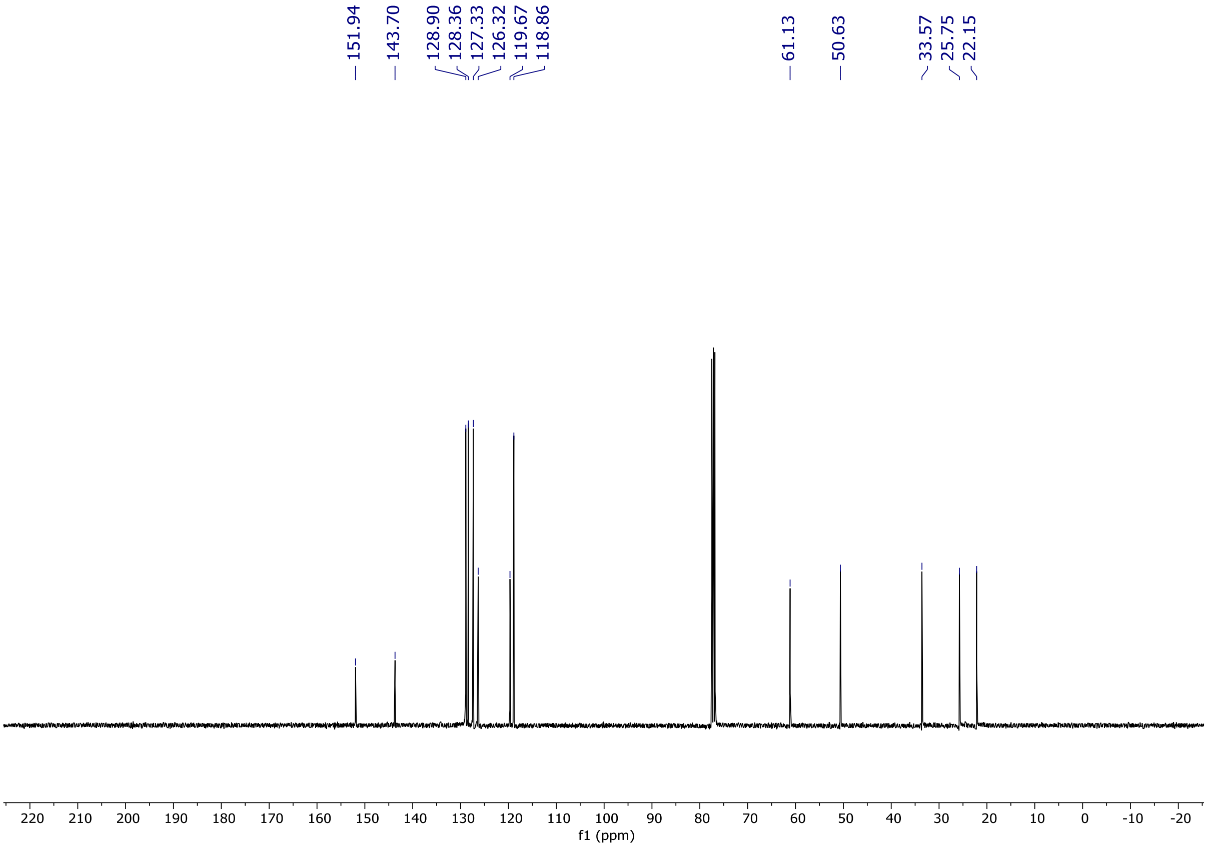

^{13}C{^{1}H} NMR (101 MHz, CDCl$_3$) spectrum of compound **39**

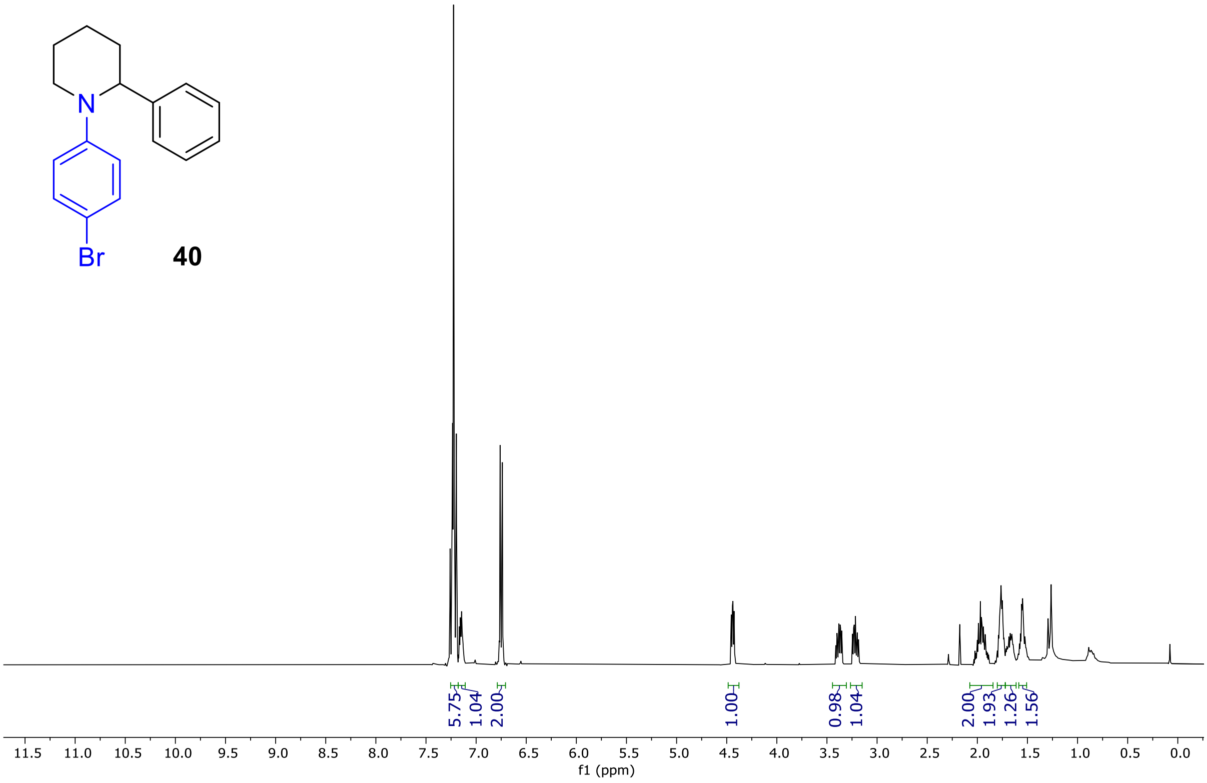

40

^{1}H NMR (400 MHz, CDCl$_3$) spectrum of compound **40**

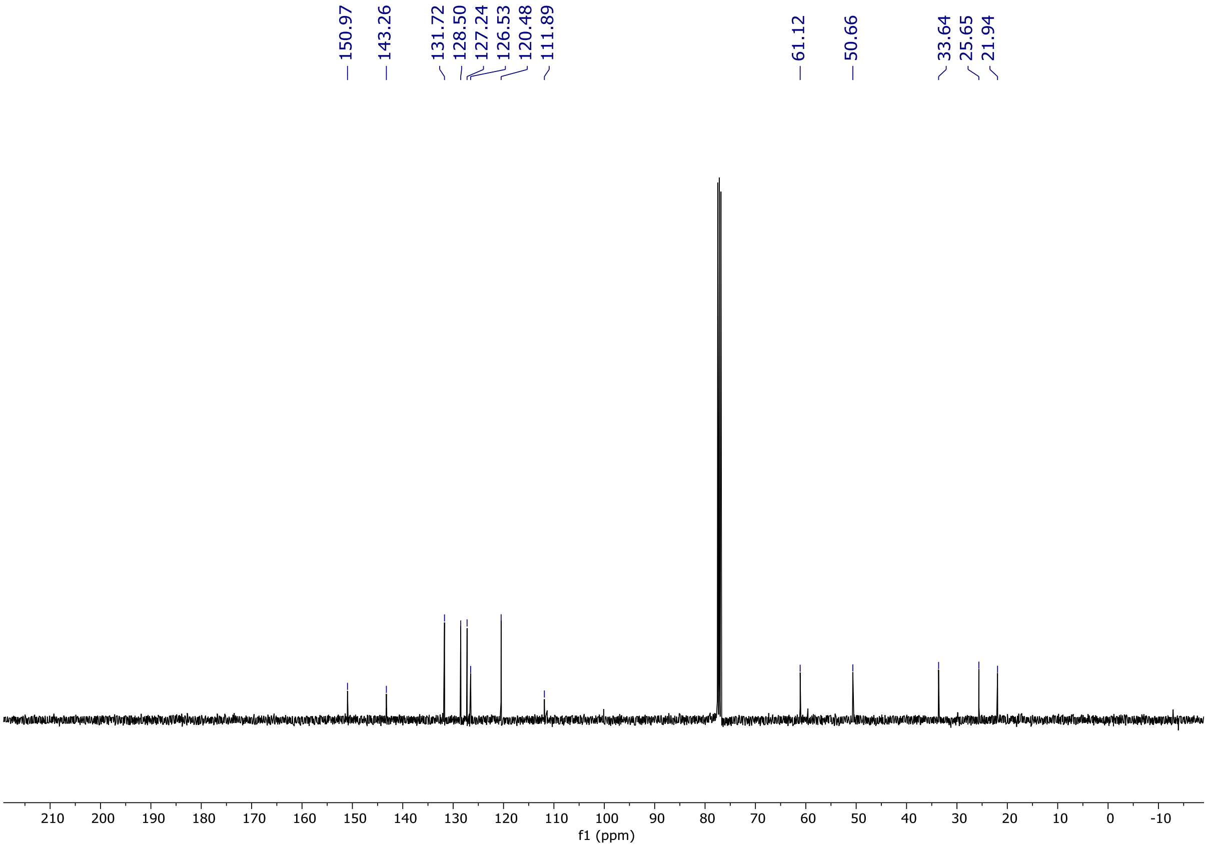

^{13}C{^{1}H} NMR (101 MHz, CDCl$_3$) spectrum of compound **40**

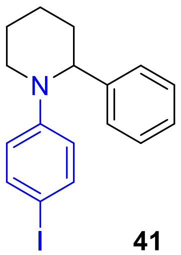

41

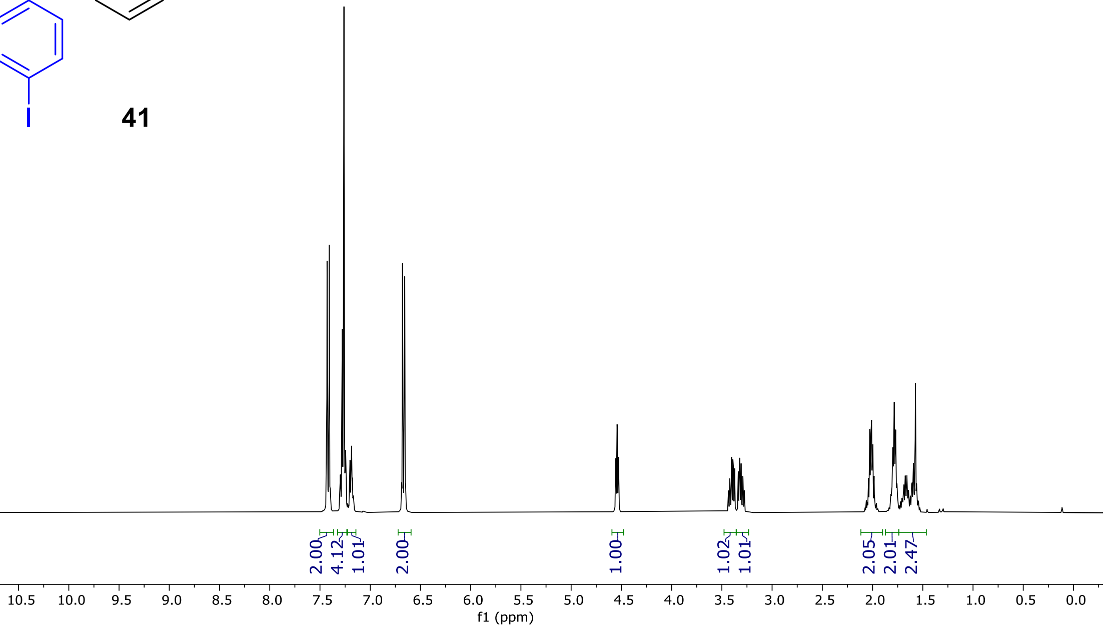

^{1}H NMR (400 MHz, $CDCl_3$) spectrum of compound **41**

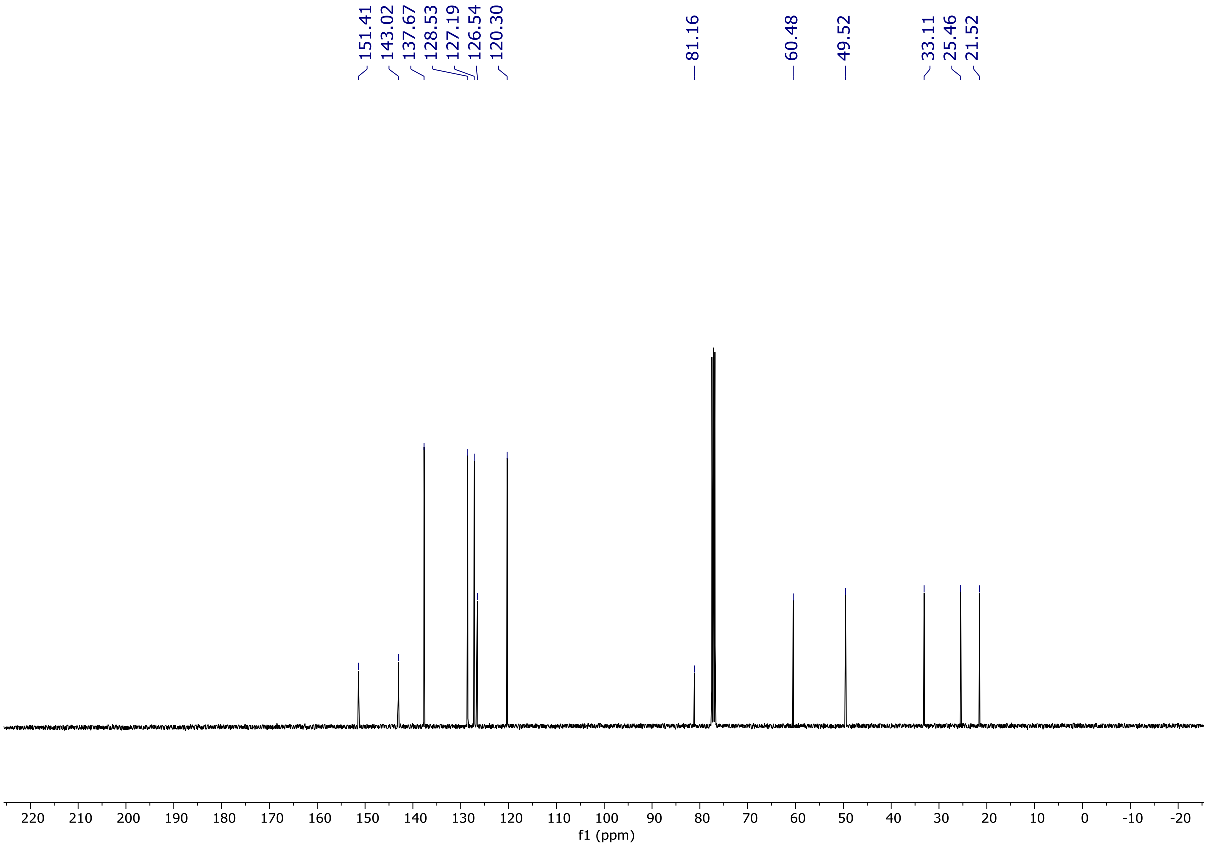

^{13}C{^{1}H} NMR (101 MHz, $CDCl_3$) spectrum of compound **41**

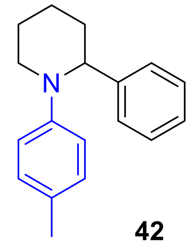

42

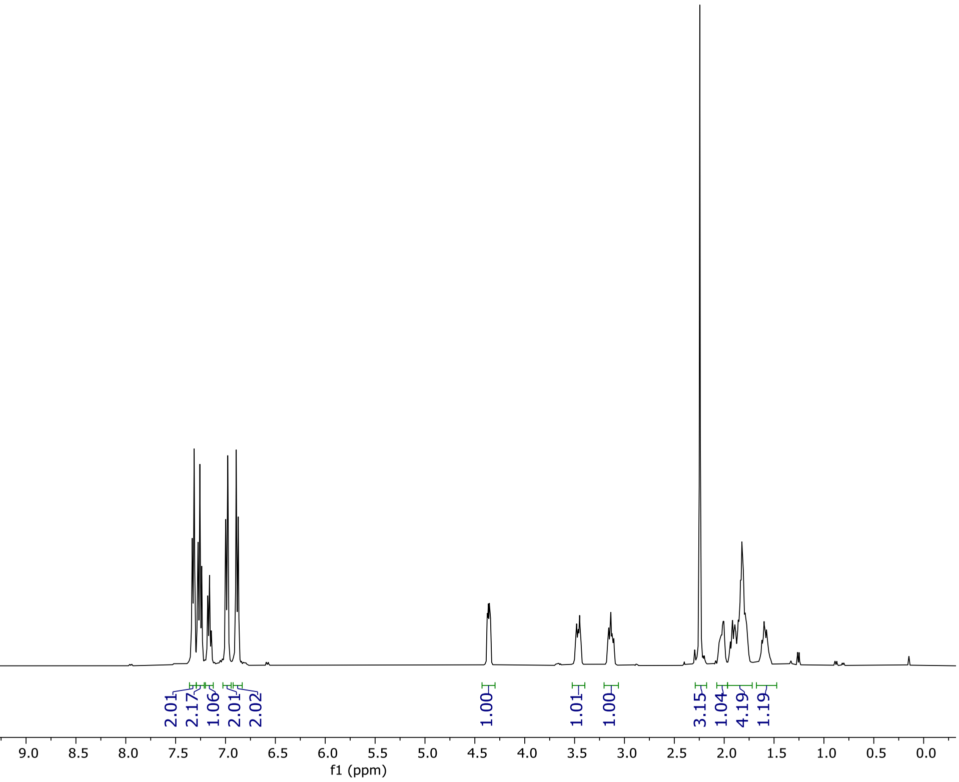

^{1}H NMR (400 MHz, $CDCl_3$) spectrum of compound **42**

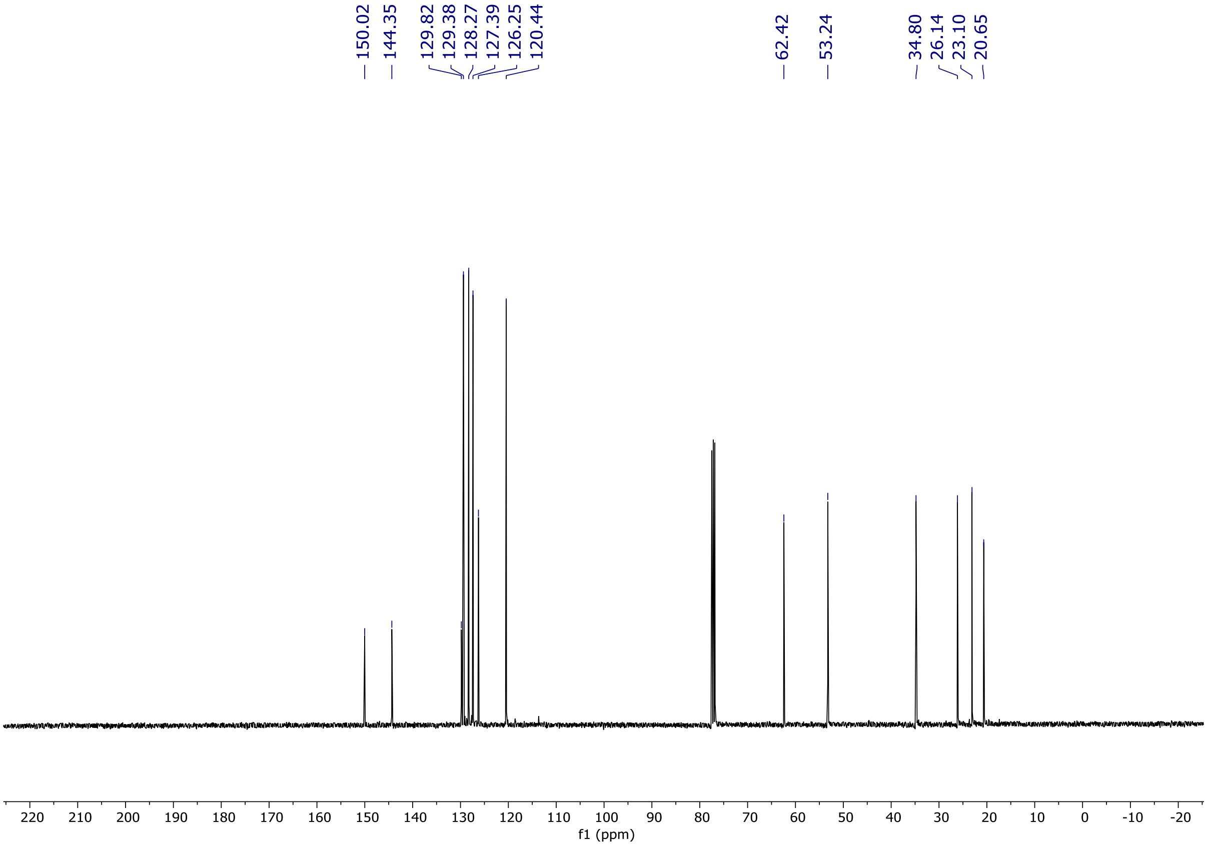

$^{13}C\{^{1}H\}$ NMR (101 MHz, $CDCl_3$) spectrum of compound **42**

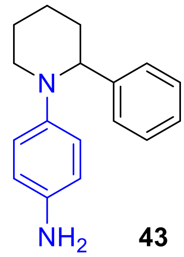

43

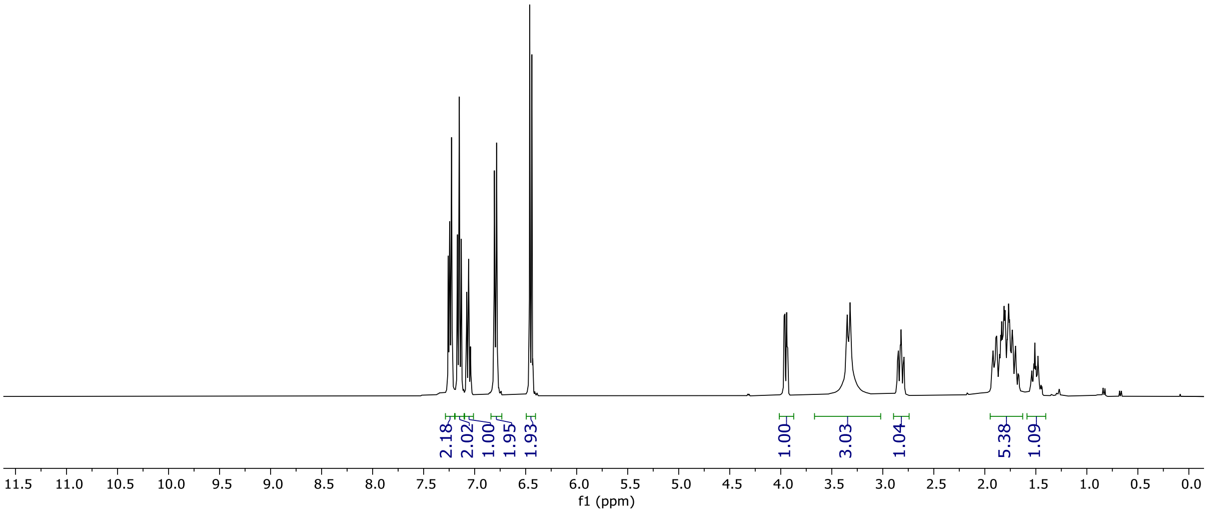

^{1}H NMR (400 MHz, $CDCl_3$) spectrum of compound **43**

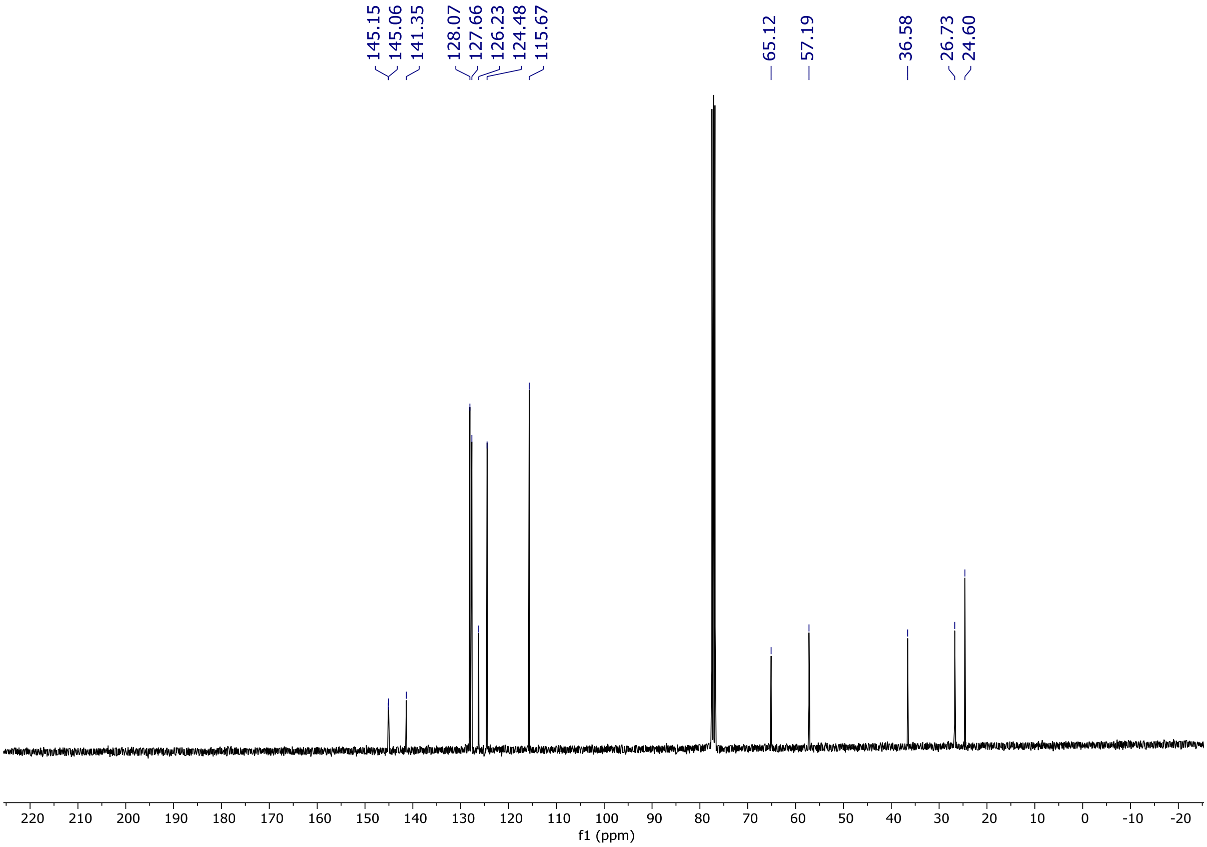

^{13}C{^{1}H} NMR (101 MHz, $CDCl_3$) spectrum of compound **43**

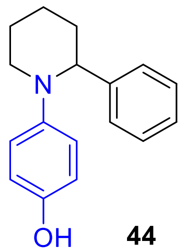

44

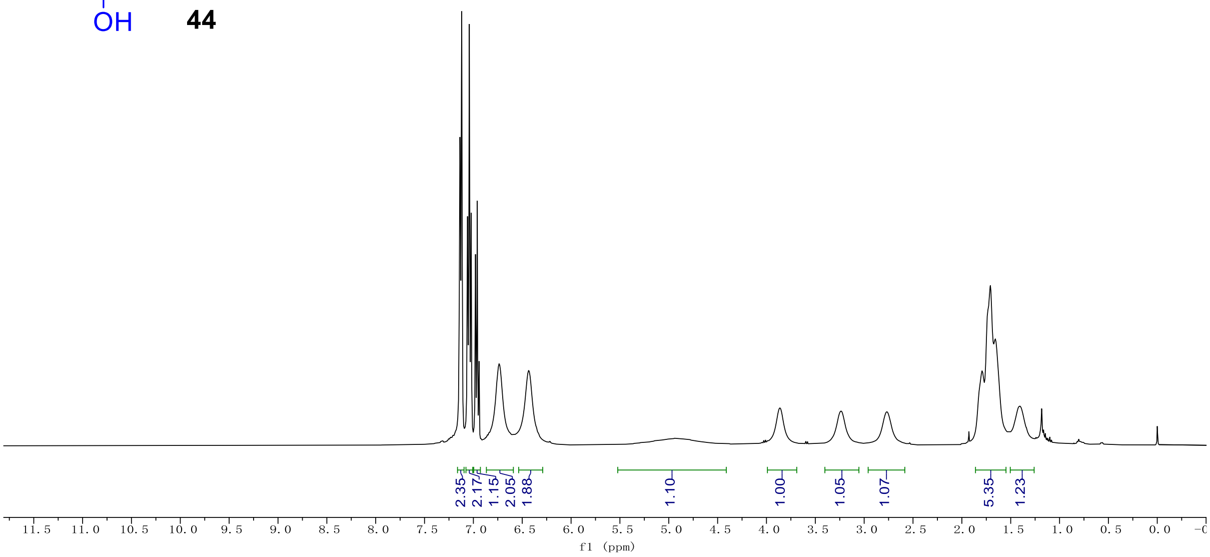

^{1}H NMR (400 MHz, $CDCl_3$) spectrum of compound **44**

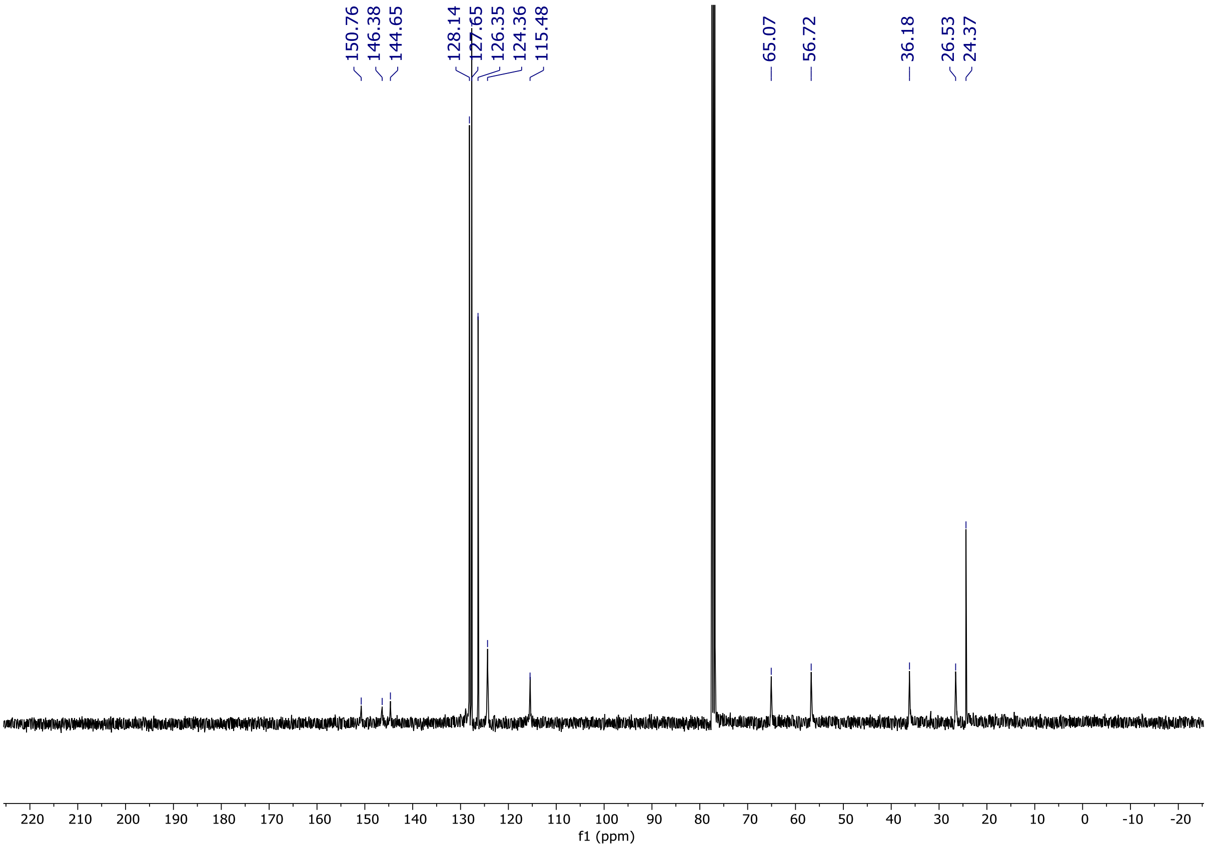

^{13}C{^{1}H} NMR (101 MHz, $CDCl_3$) spectrum of compound **44**

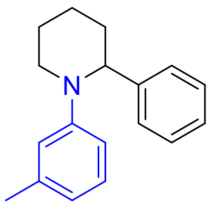

45

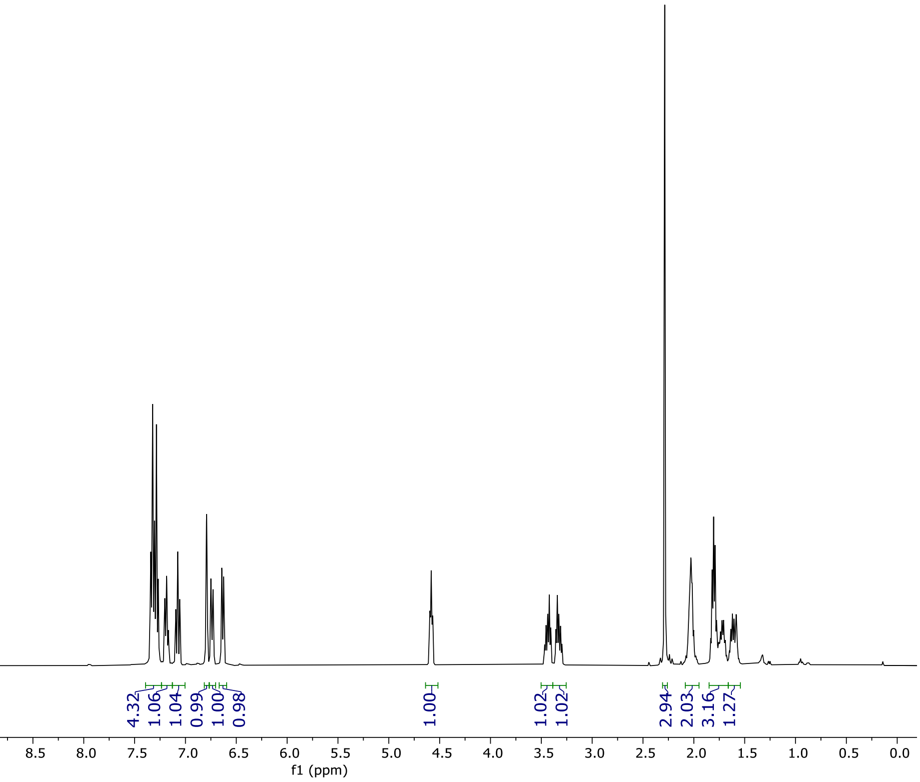

^{1}H NMR (400 MHz, $CDCl_3$) spectrum of compound **45**

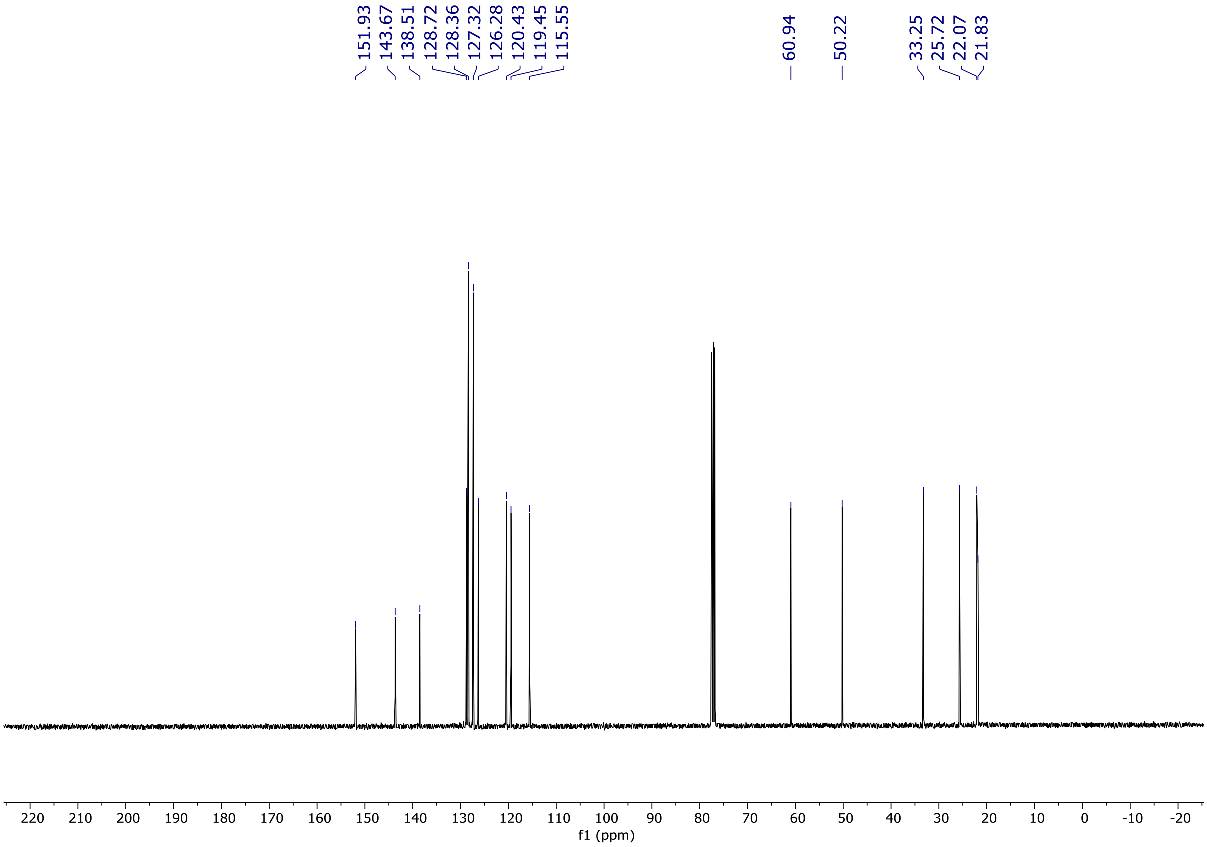

^{13}C{^{1}H} NMR (101 MHz, $CDCl_3$) spectrum of compound **45**

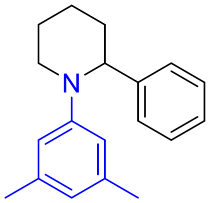

46

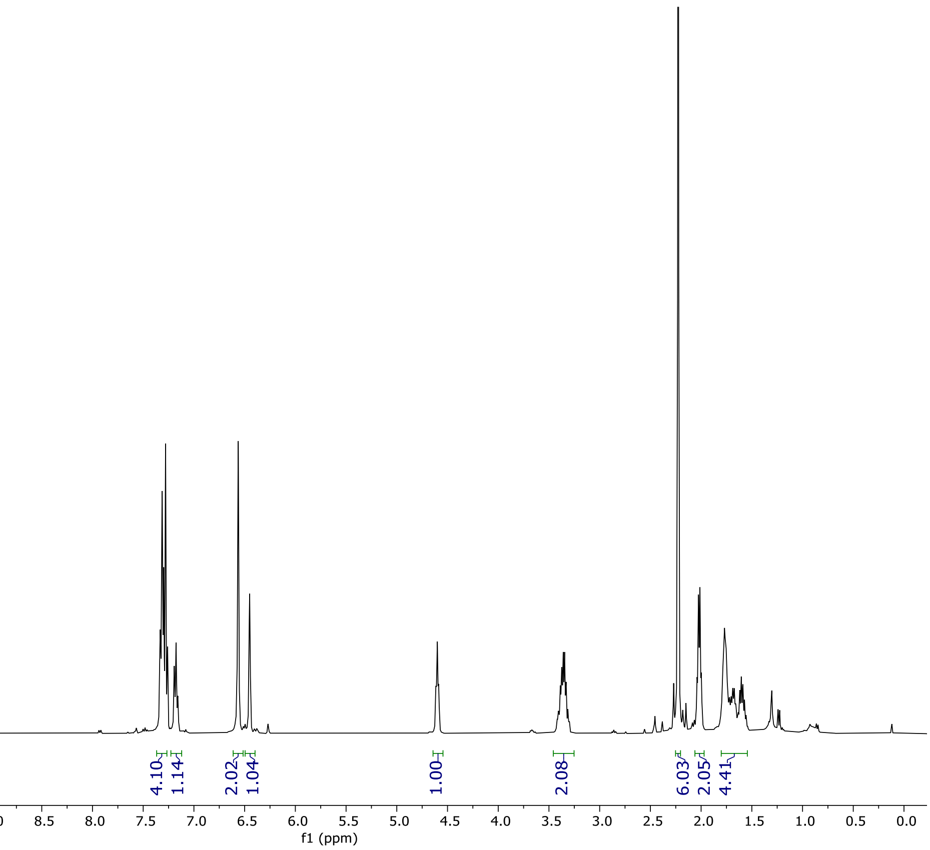

^{1}H NMR (400 MHz, $CDCl_3$) spectrum of compound **46**

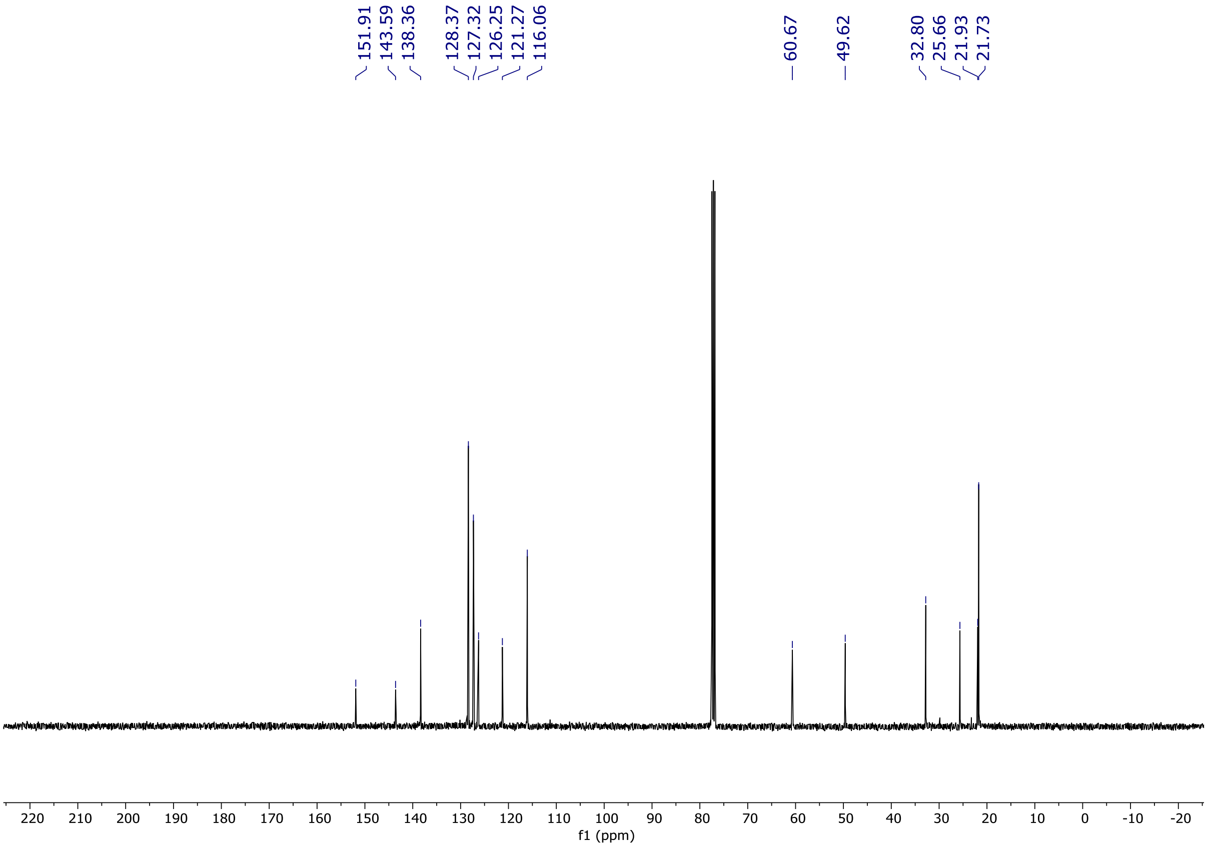

^{13}C{^{1}H} NMR (101 MHz, $CDCl_3$) spectrum of compound **46**

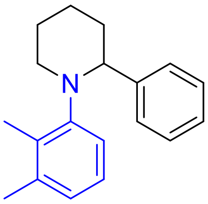

47

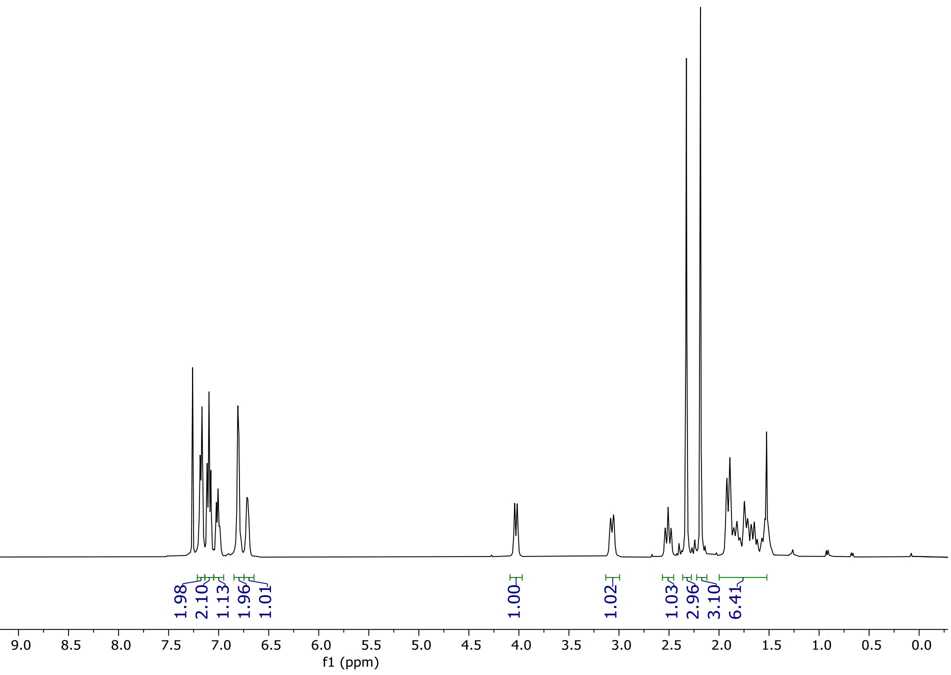

^{1}H NMR (400 MHz, $CDCl_3$) spectrum of compound **47**

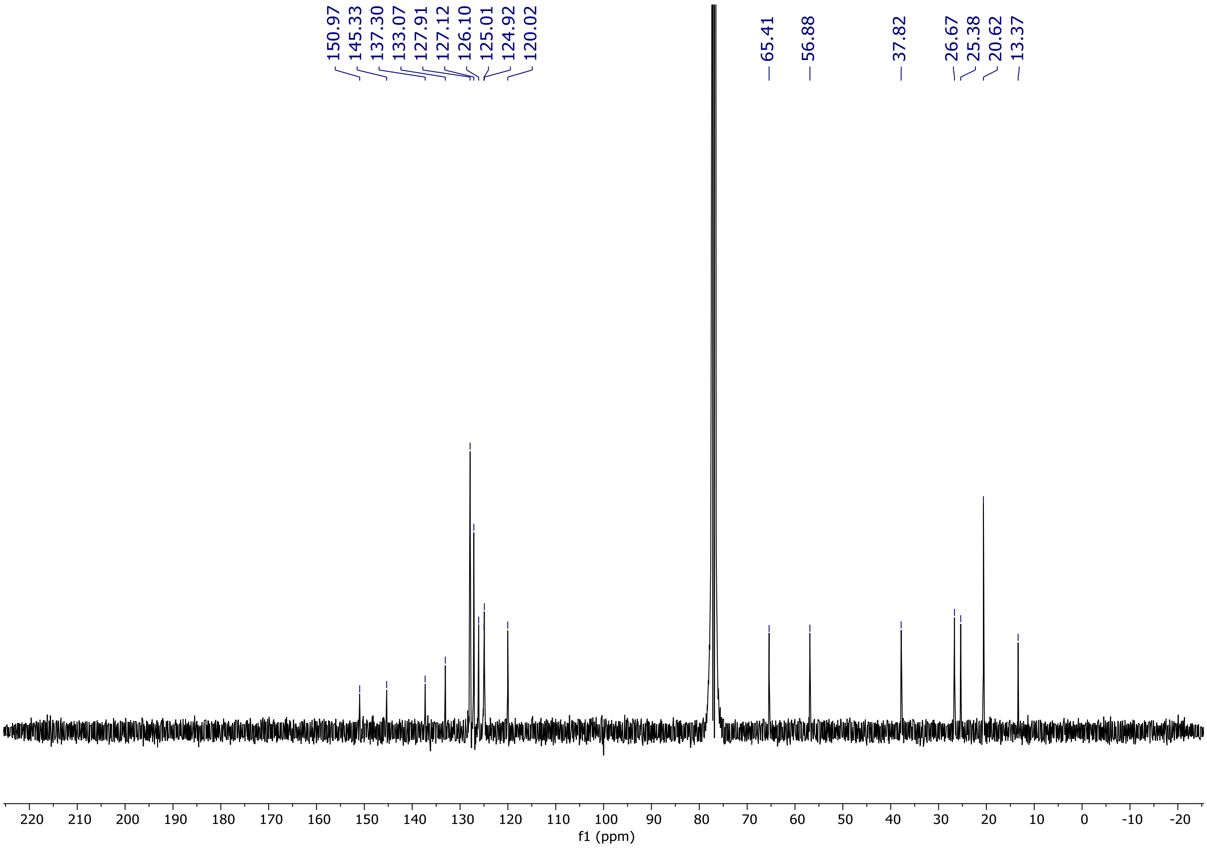

^{13}C{^{1}H} NMR (101 MHz, $CDCl_3$) spectrum of compound **47**

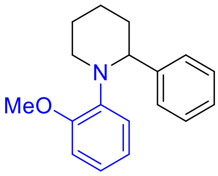

48

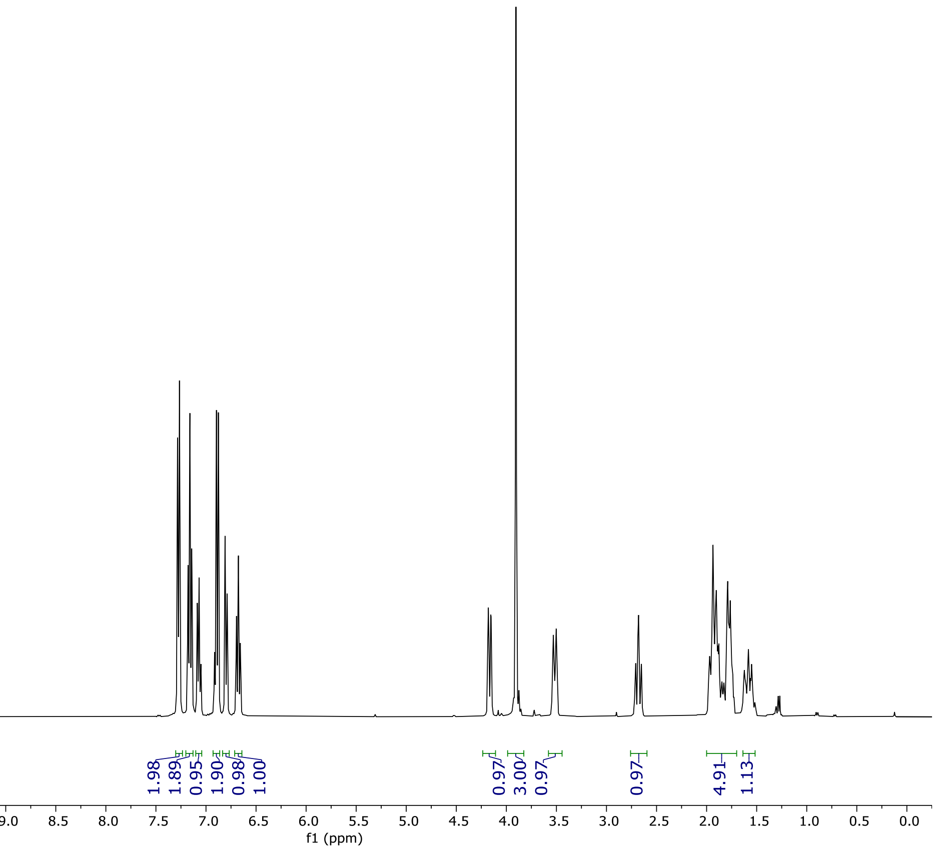

^{1}H NMR (400 MHz, $CDCl_3$) spectrum of compound **48**

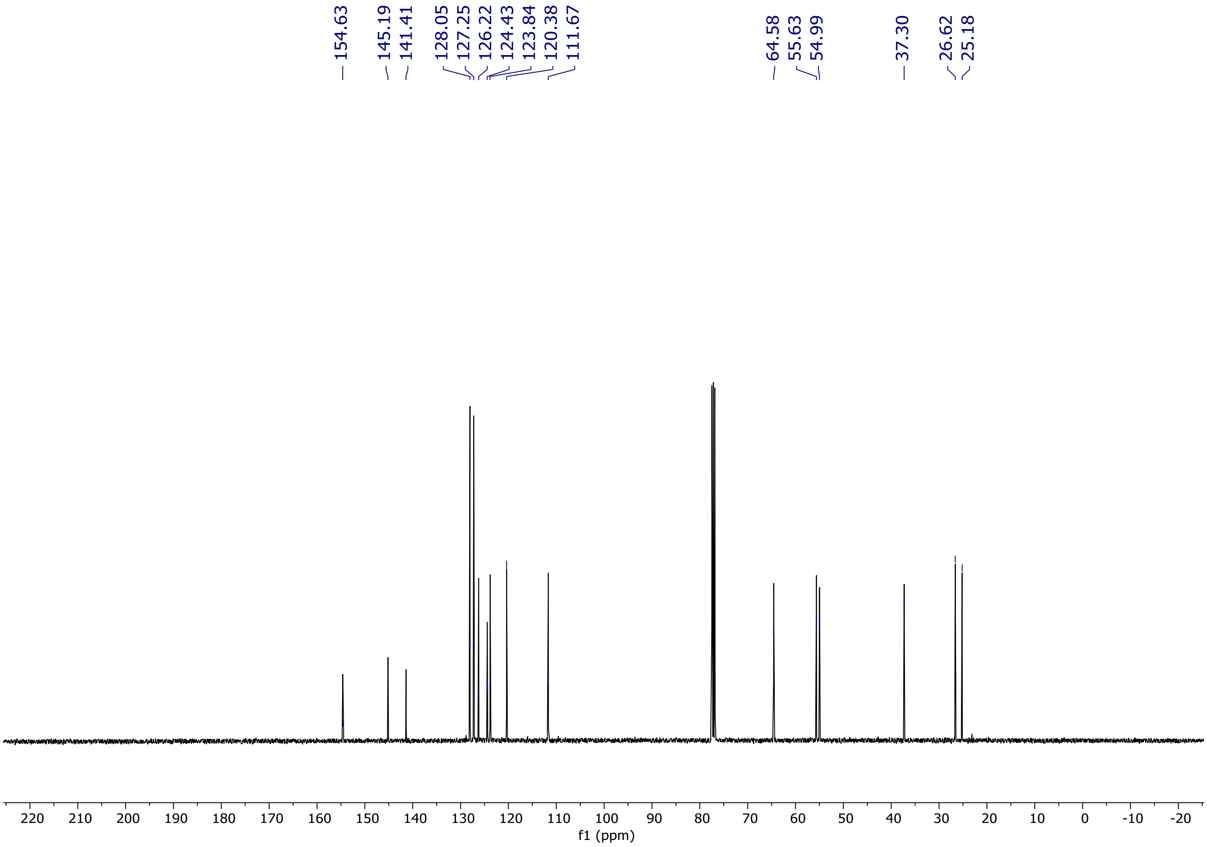

^{13}C{^{1}H} NMR (101 MHz, $CDCl_3$) spectrum of compound **48**

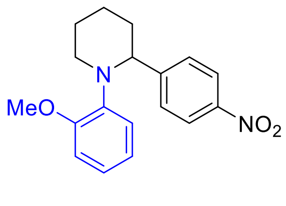

49

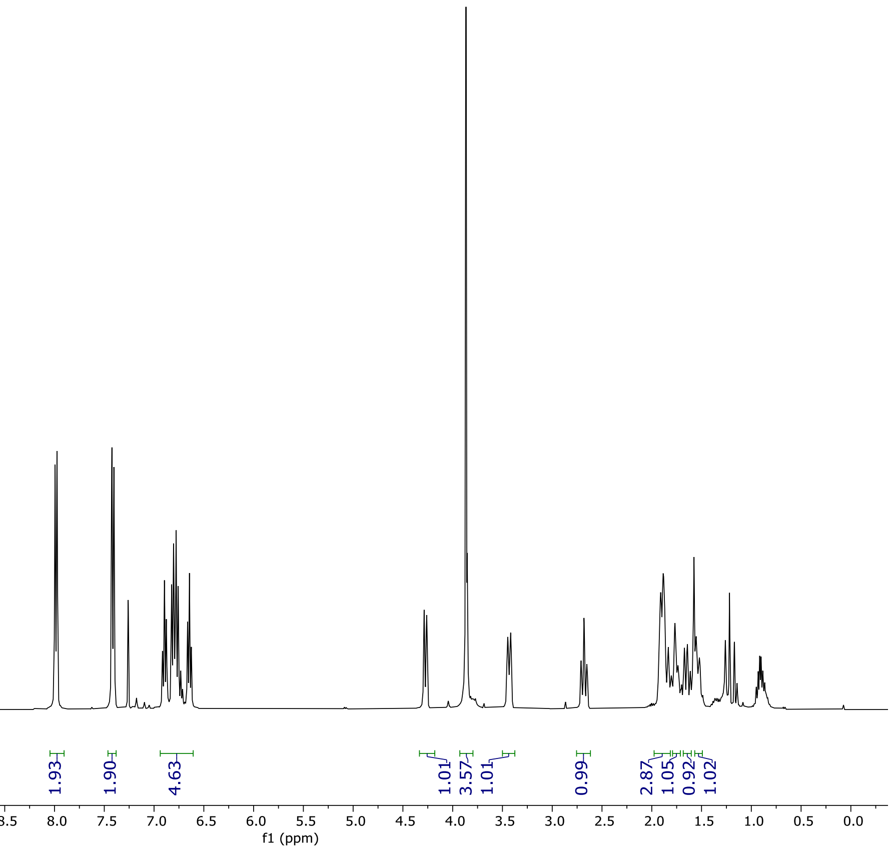

^{1}H NMR (400 MHz, $CDCl_3$) spectrum of compound **49**

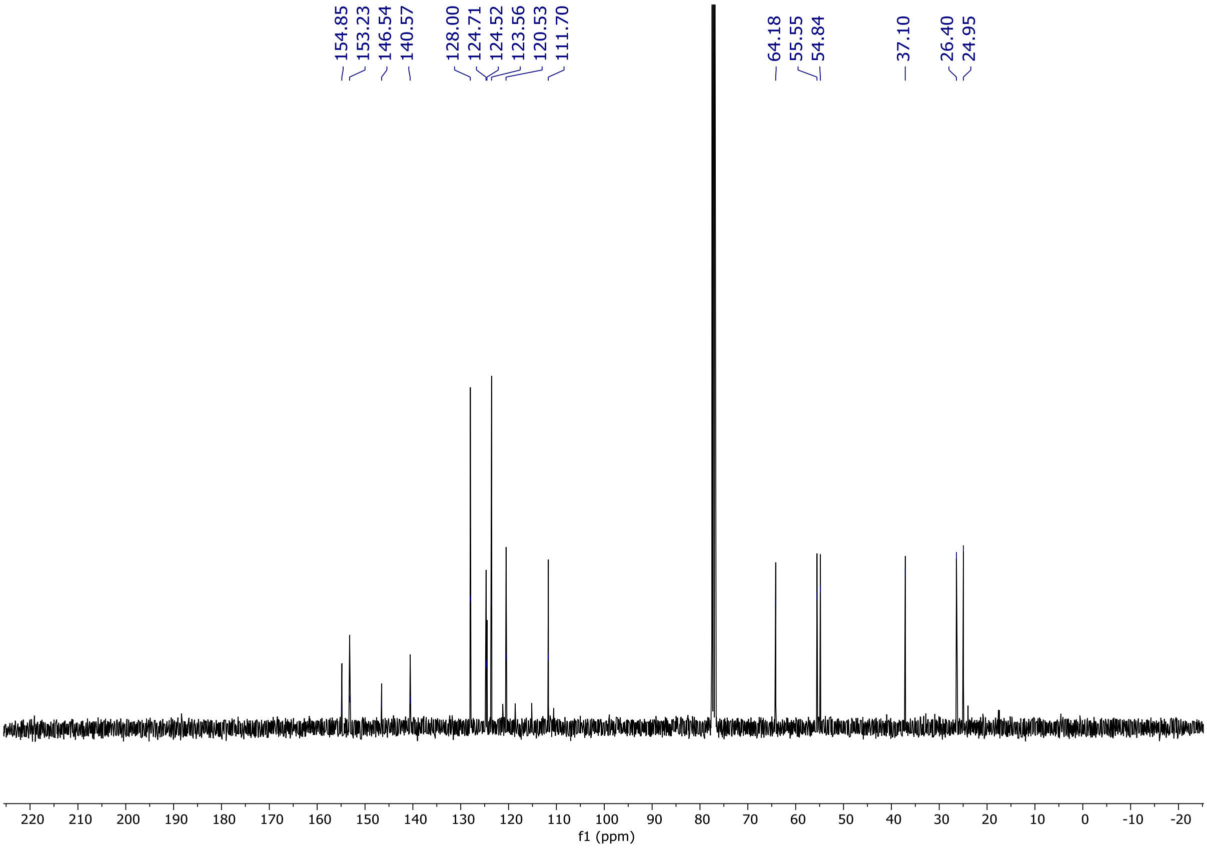

^{13}C{^{1}H} NMR (101 MHz, $CDCl_3$) spectrum of compound **49**

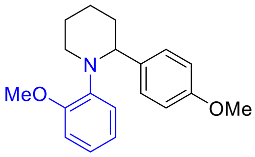

50

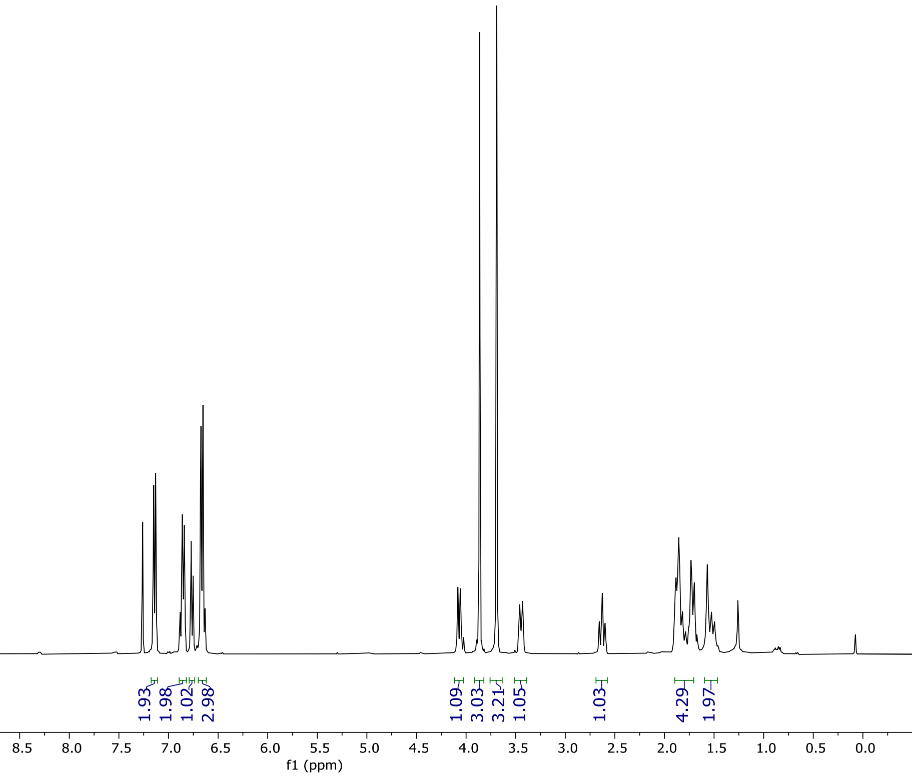

^{1}H NMR (400 MHz, $CDCl_3$) spectrum of compound **50**

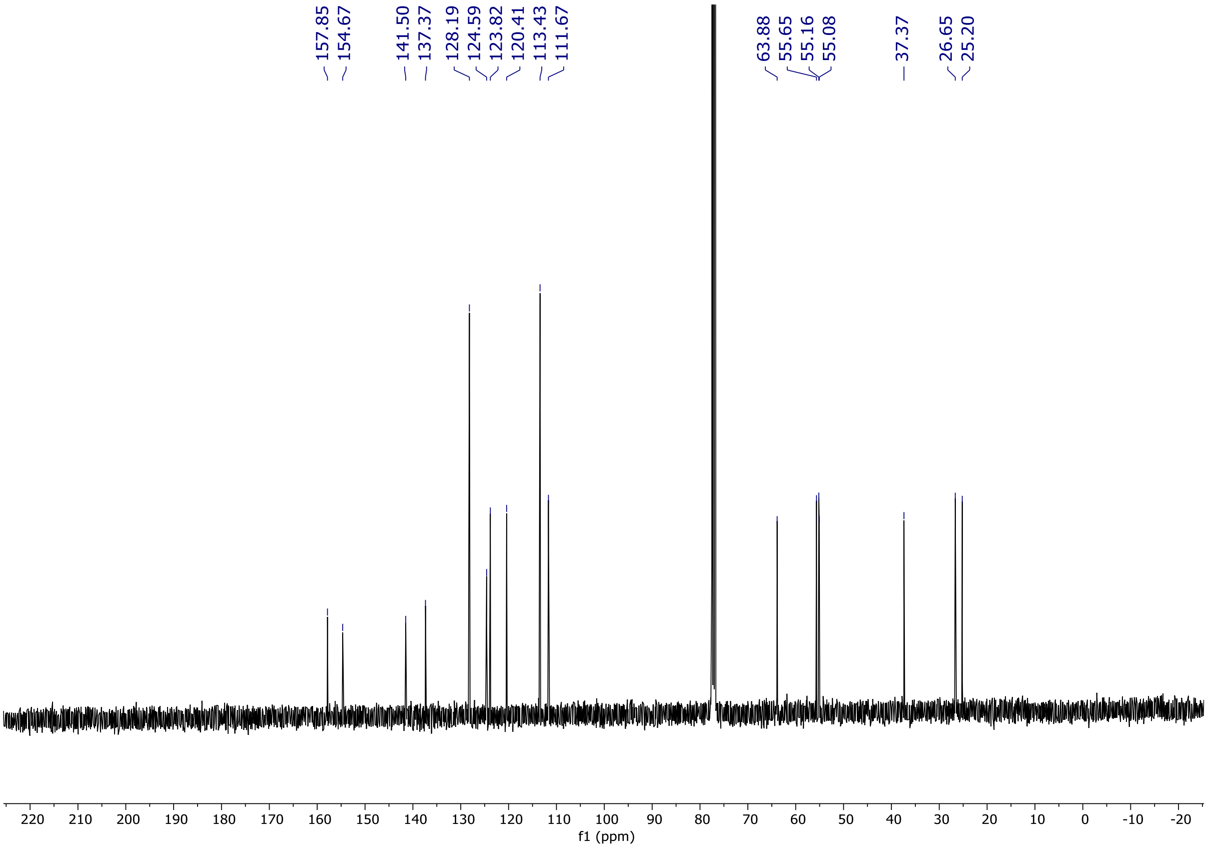

^{13}C{^{1}H} NMR (101 MHz, $CDCl_3$) spectrum of compound **50**

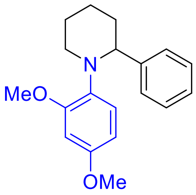

51

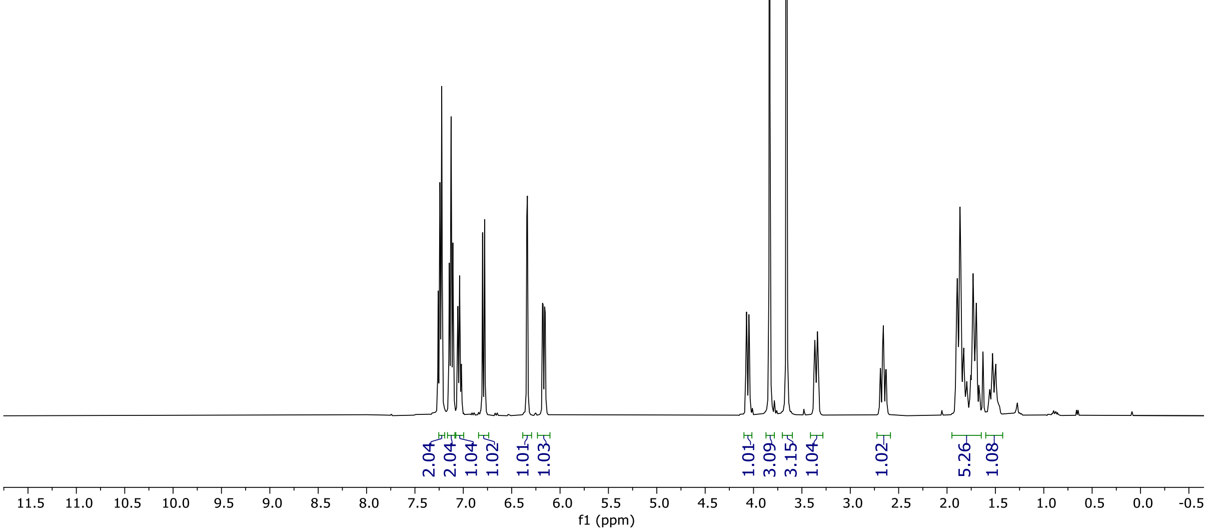

^{1}H NMR (400 MHz, $CDCl_3$) spectrum of compound **51**

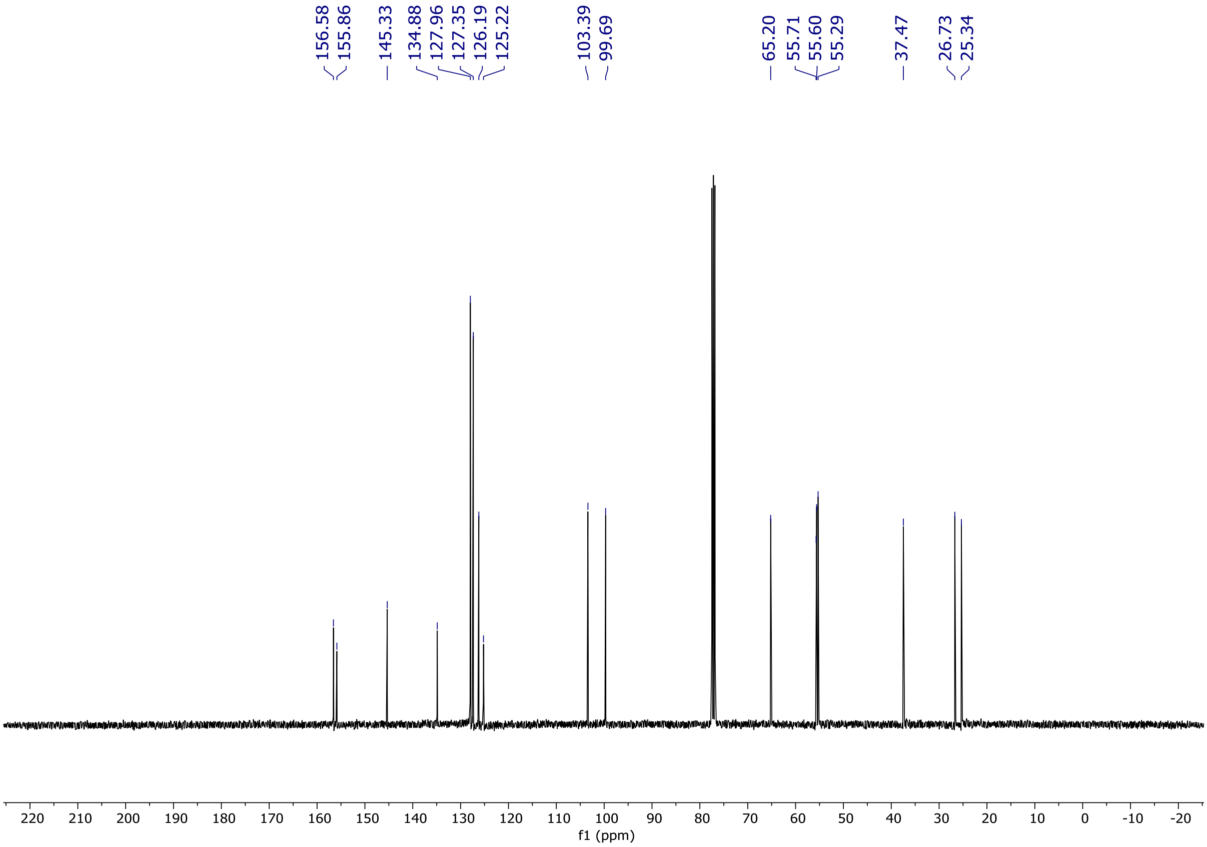

^{13}C{^{1}H} NMR (101 MHz, $CDCl_3$) spectrum of compound **51**

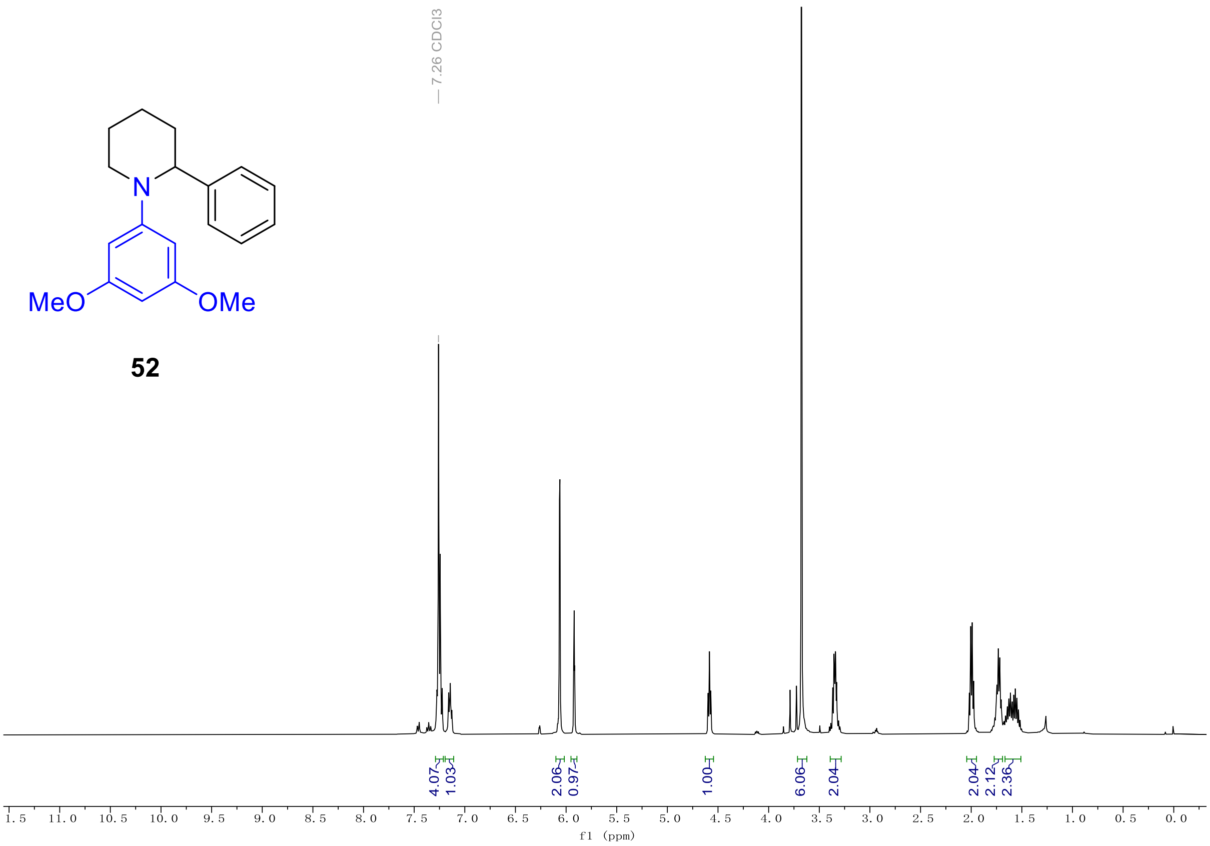

^{1}H NMR (400 MHz, $CDCl_3$) spectrum of compound **52**

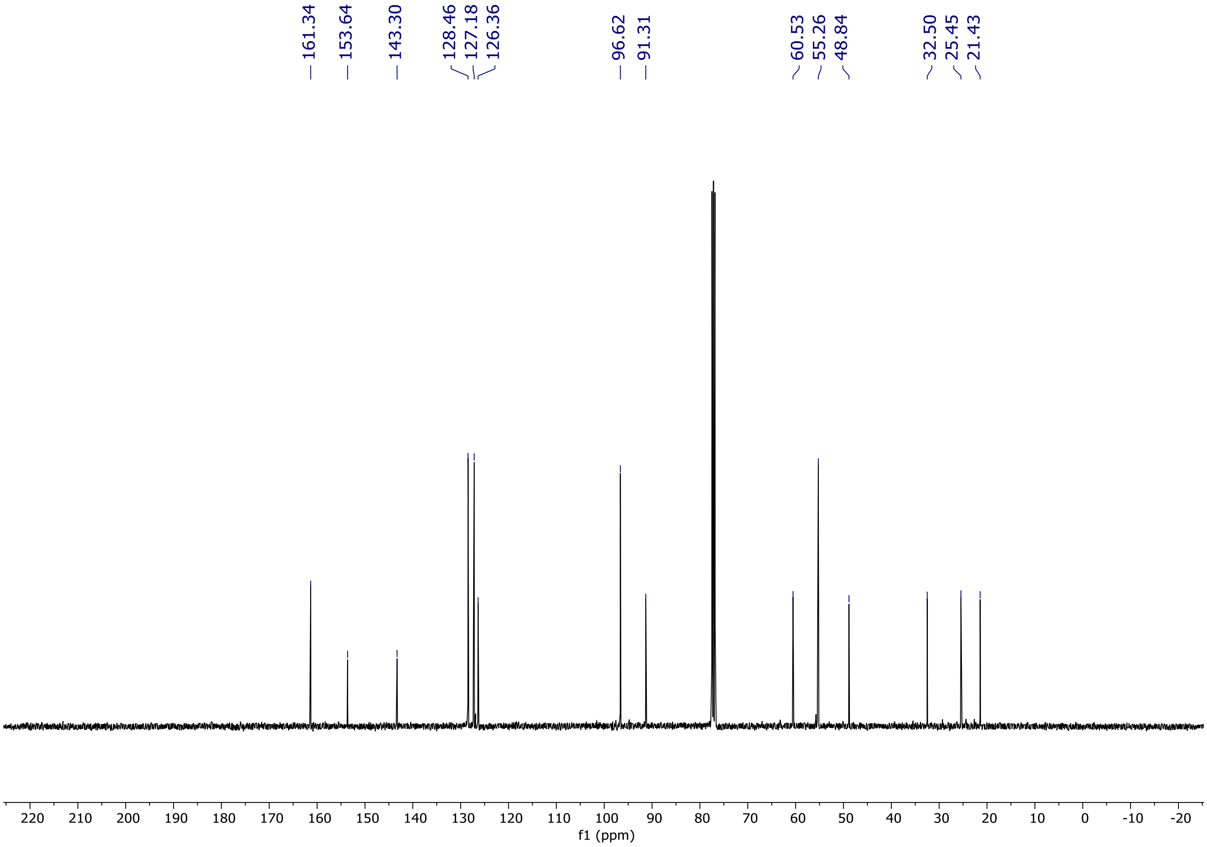

^{13}C{^{1}H} NMR (101 MHz, $CDCl_3$) spectrum of compound **52**

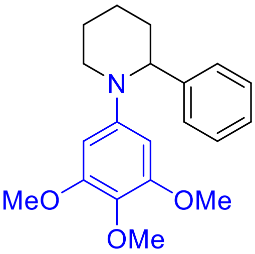

53

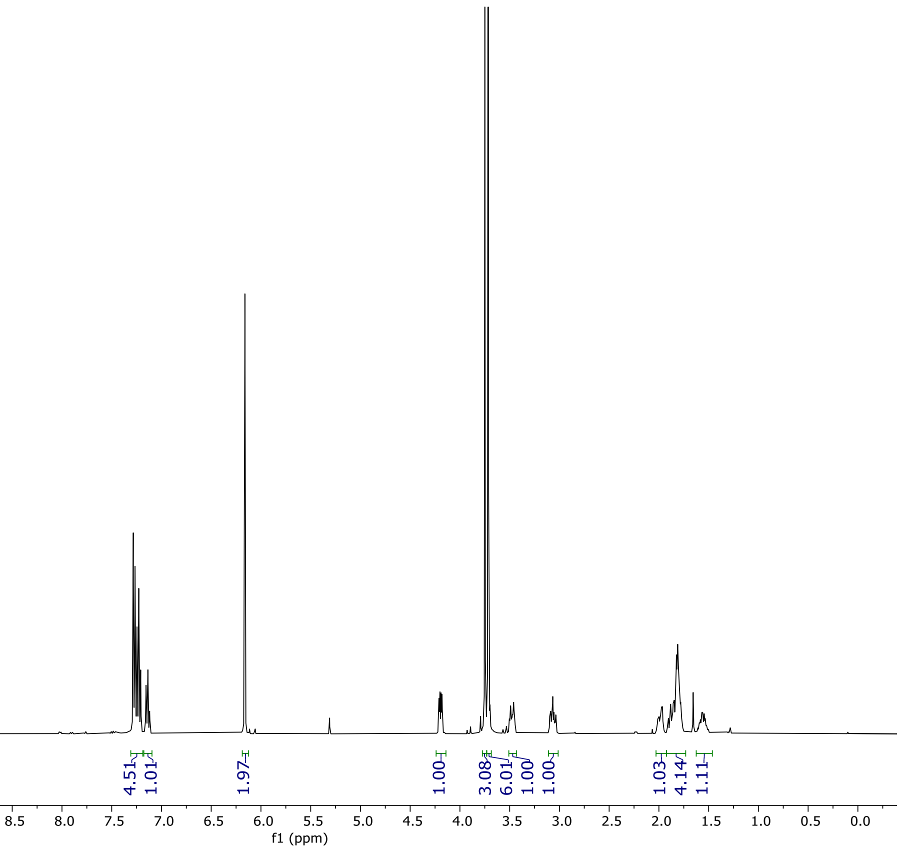

^{1}H NMR (400 MHz, $CDCl_3$) spectrum of compound **53**

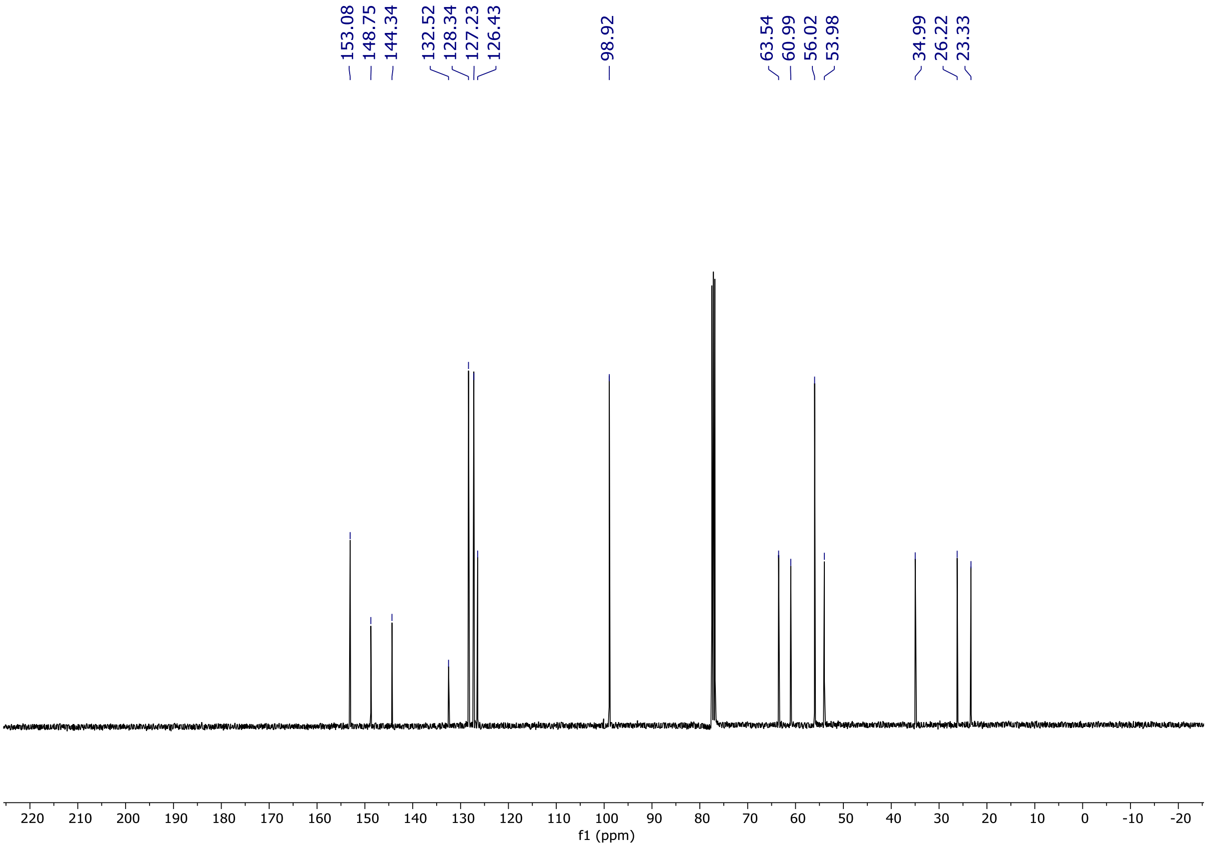

^{13}C{^{1}H} NMR (101 MHz, $CDCl_3$) spectrum of compound **53**

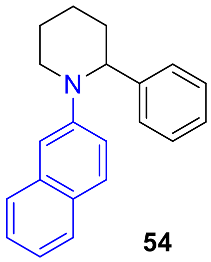

54

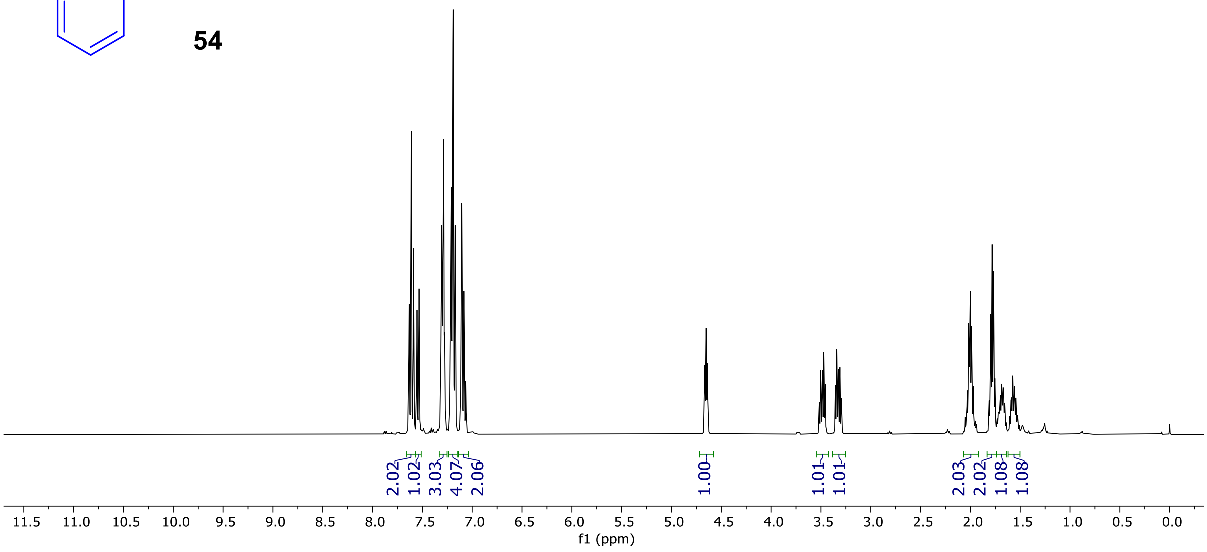

^{1}H NMR (400 MHz, $CDCl_3$) spectrum of compound **54**

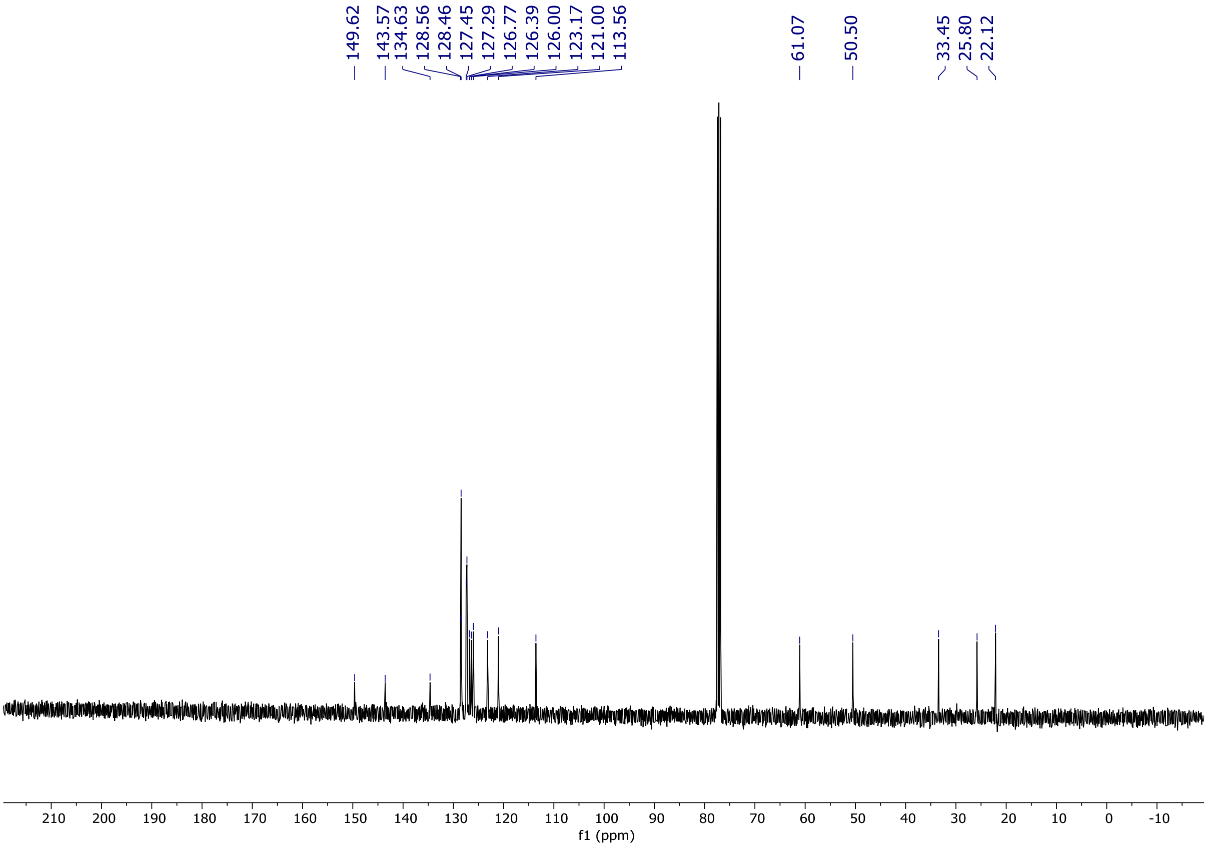

^{13}C{^{1}H} NMR (101 MHz, $CDCl_3$) spectrum of compound **54**

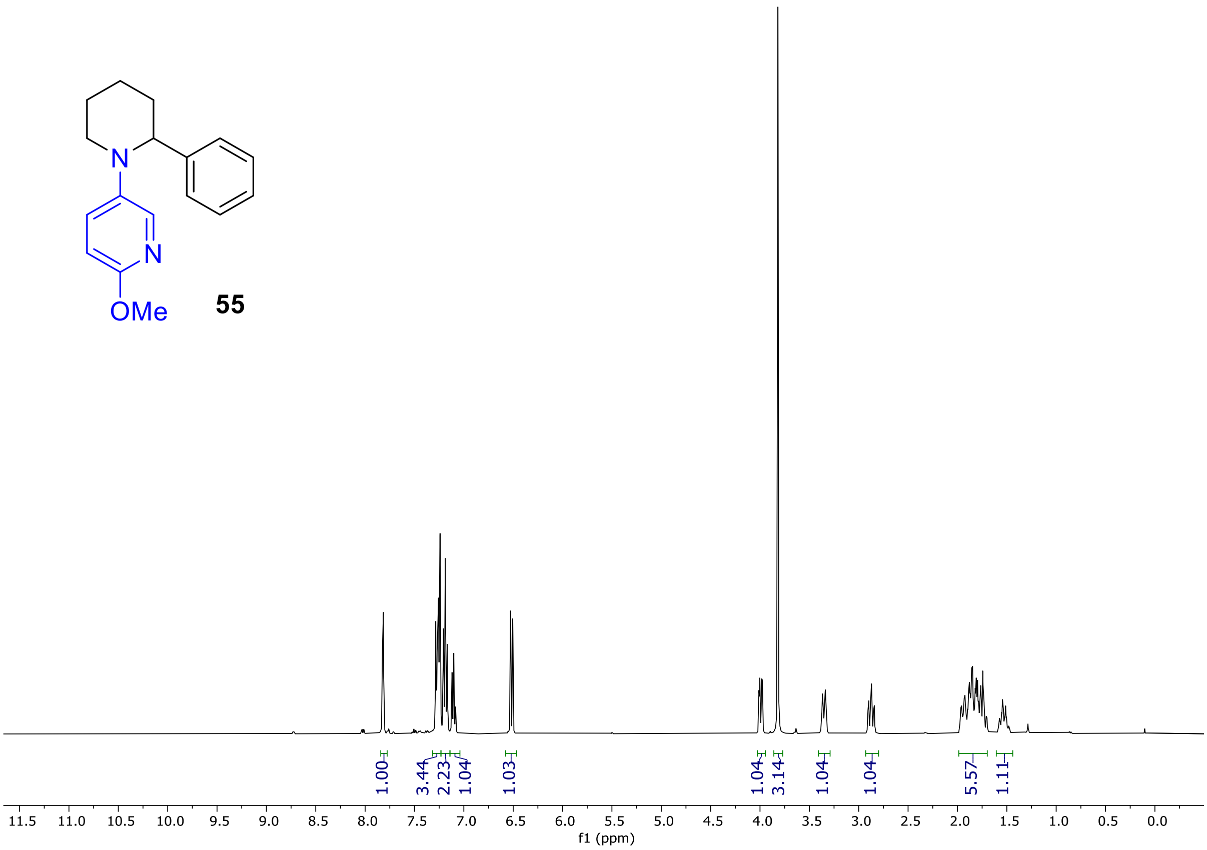

^{1}H NMR (400 MHz, $CDCl_3$) spectrum of compound **55**

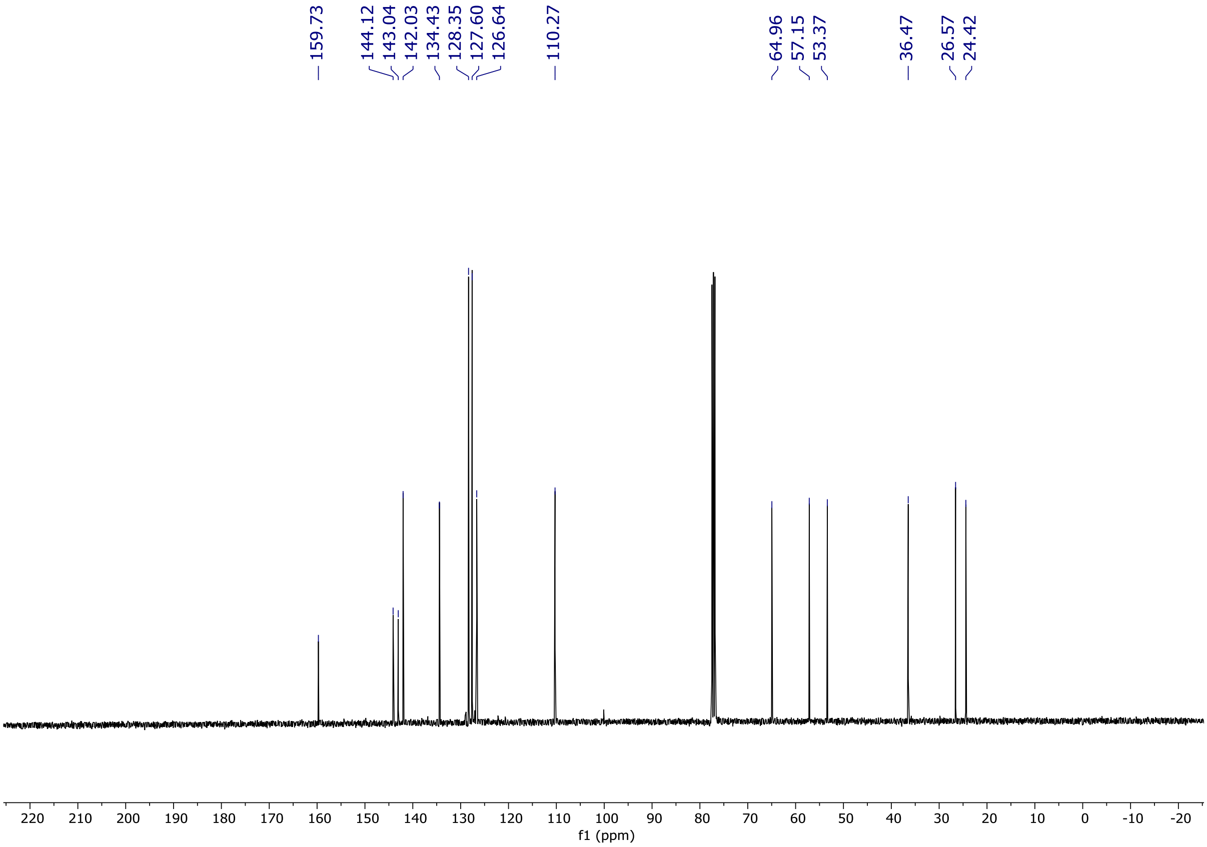

$^{13}C\{^1H\}$ NMR (101 MHz, $CDCl_3$) spectrum of compound **55**

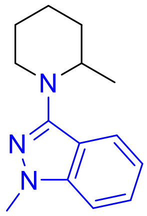

56

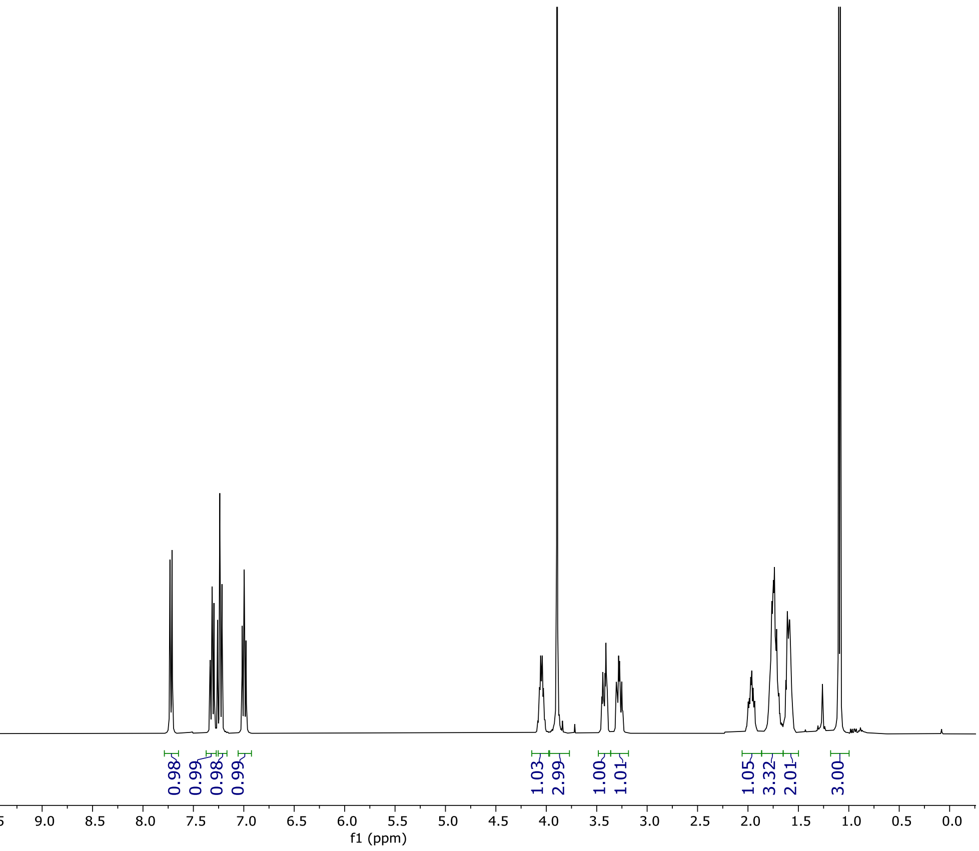

^{1}H NMR (400 MHz, $CDCl_3$) spectrum of compound **56**

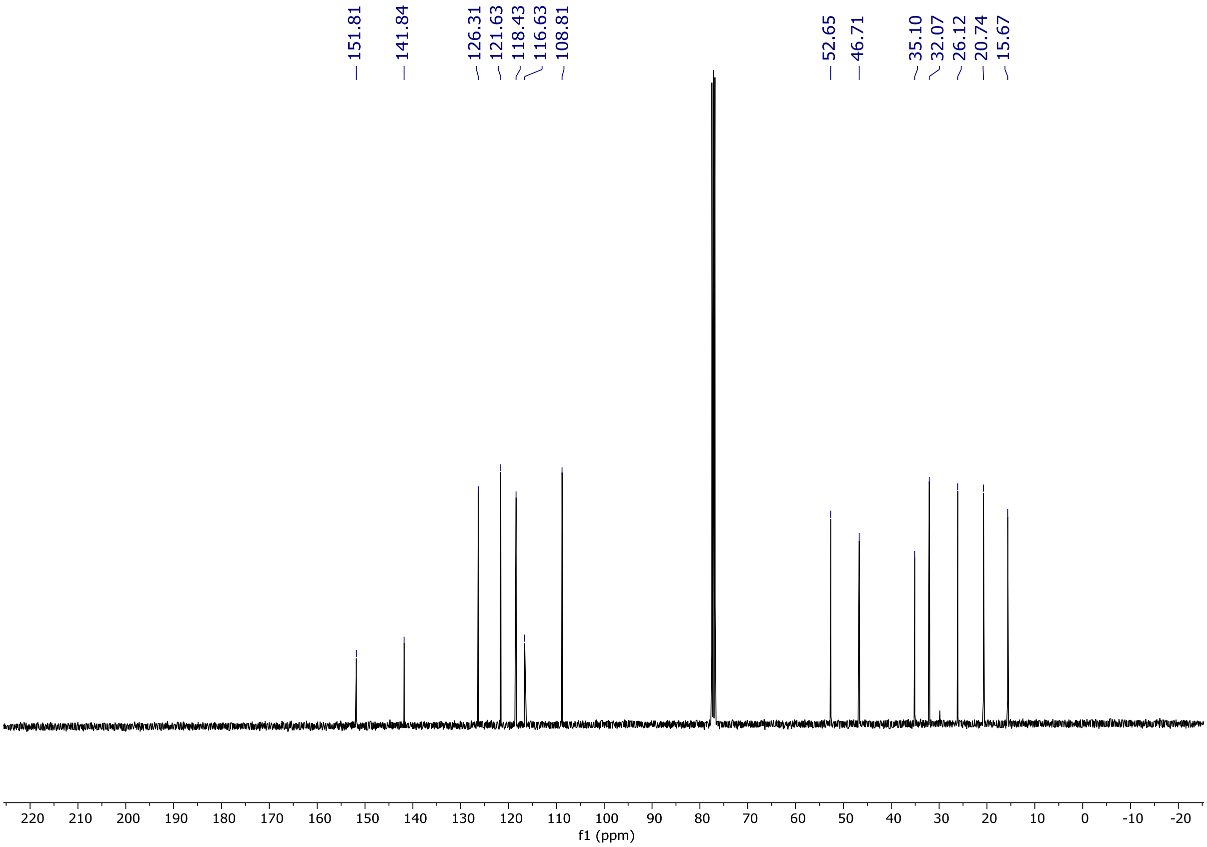

$^{13}C\{^{1}H\}$ NMR (101 MHz, $CDCl_3$) spectrum of compound **56**

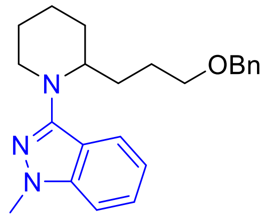

57

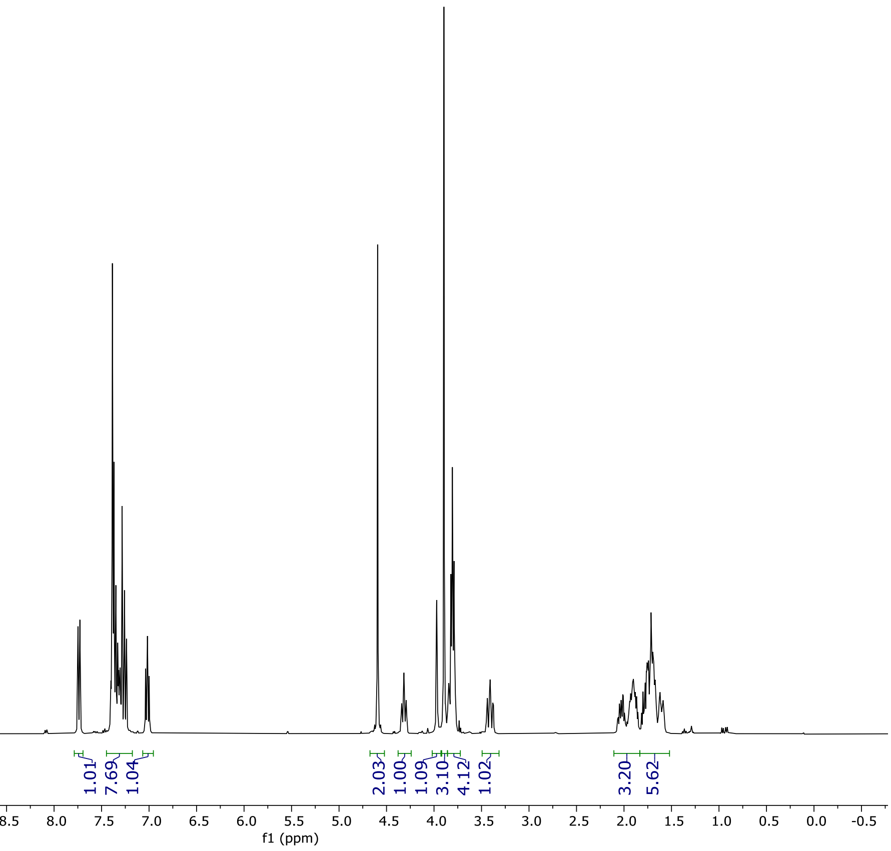

^{1}H NMR (400 MHz, $CDCl_3$) spectrum of compound **57**

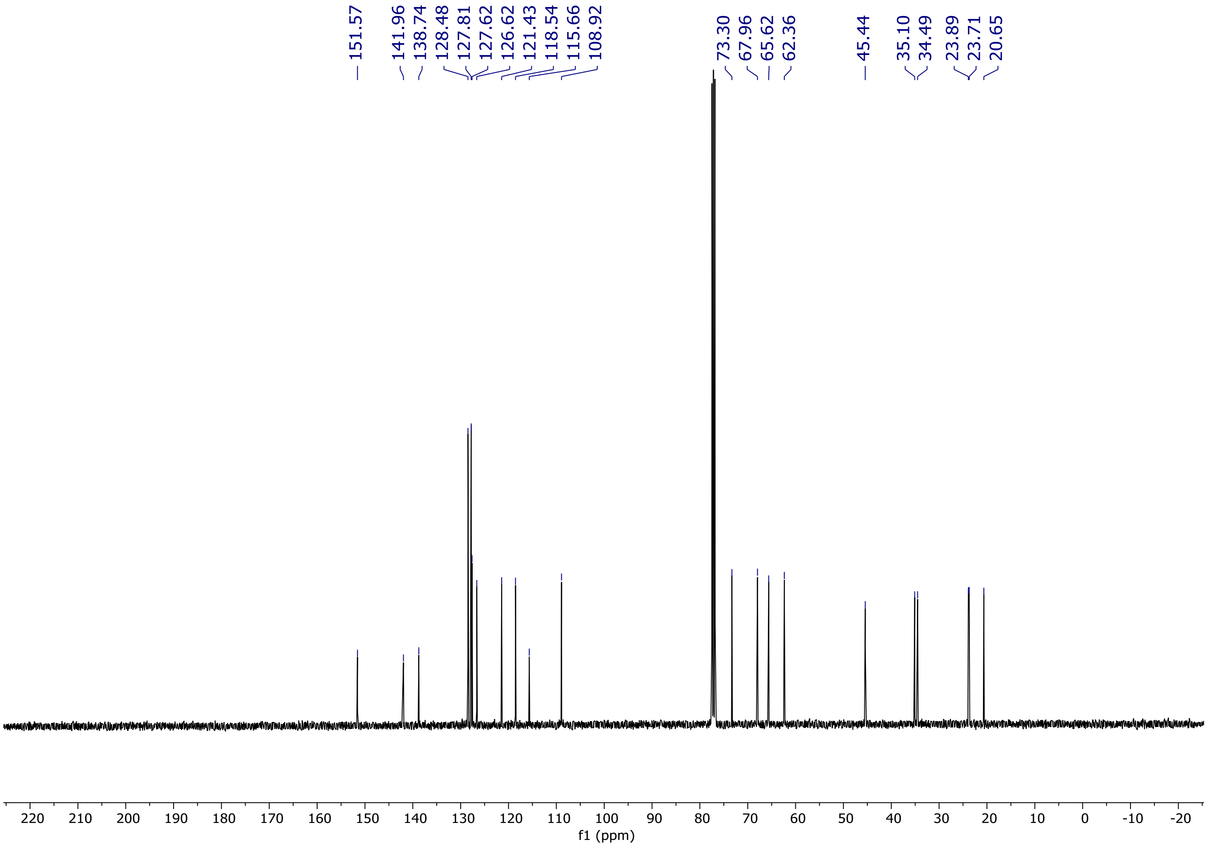

^{13}C{^{1}H} NMR (101 MHz, $CDCl_3$) spectrum of compound **57**

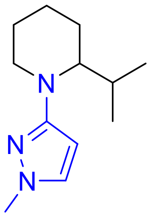

58

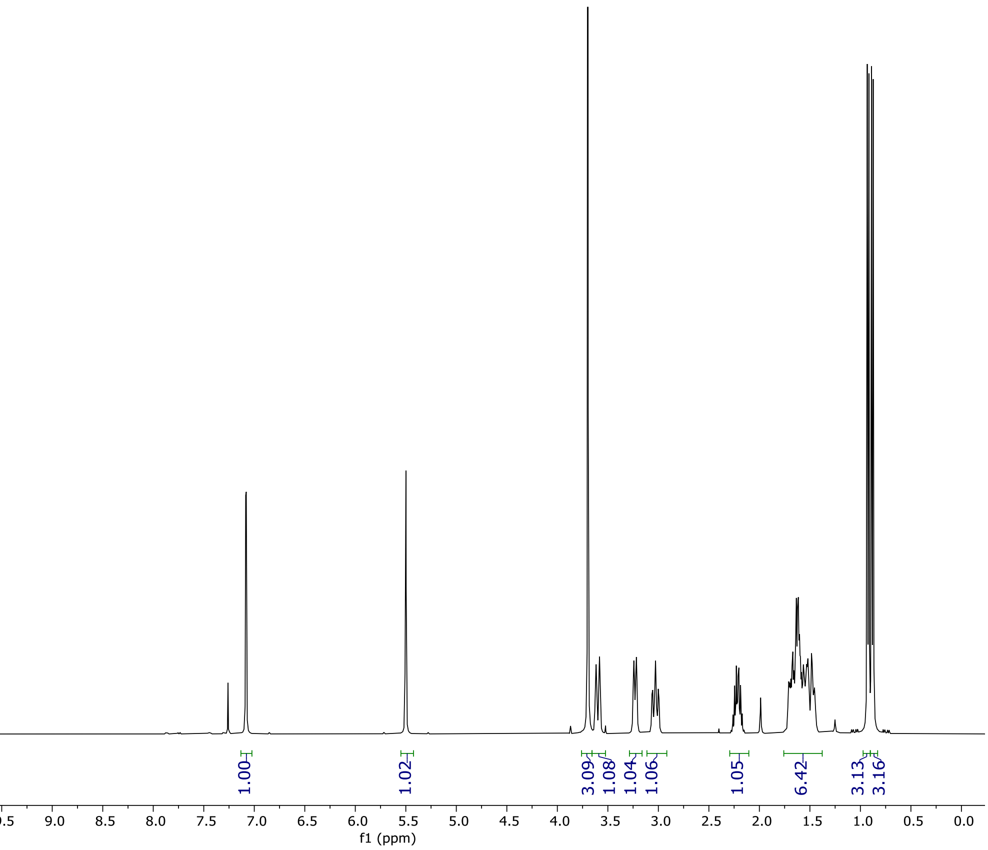

^{1}H NMR (400 MHz, $CDCl_3$) spectrum of compound **58**

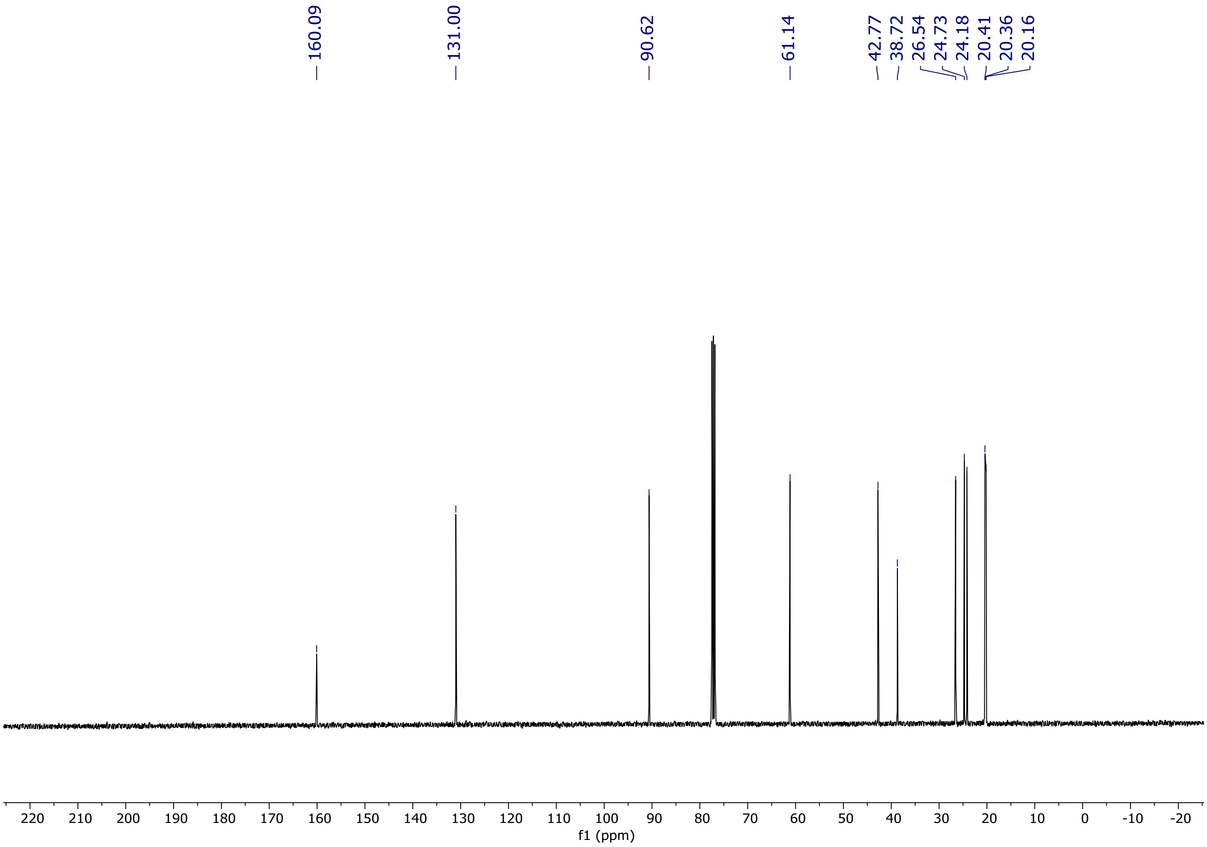

$^{13}C\{^{1}H\}$ NMR (101 MHz, $CDCl_3$) spectrum of compound **58**

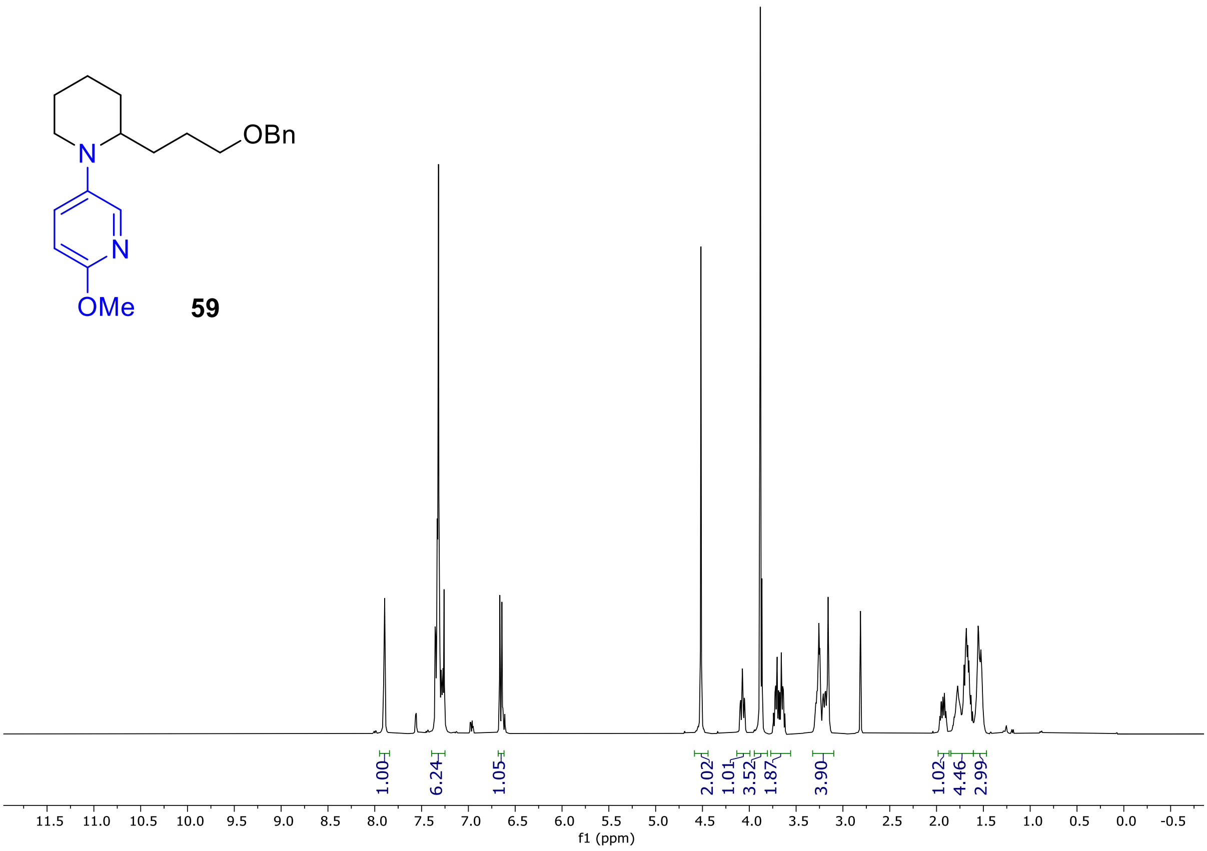

^{1}H NMR (400 MHz, $CDCl_3$) spectrum of compound **59**

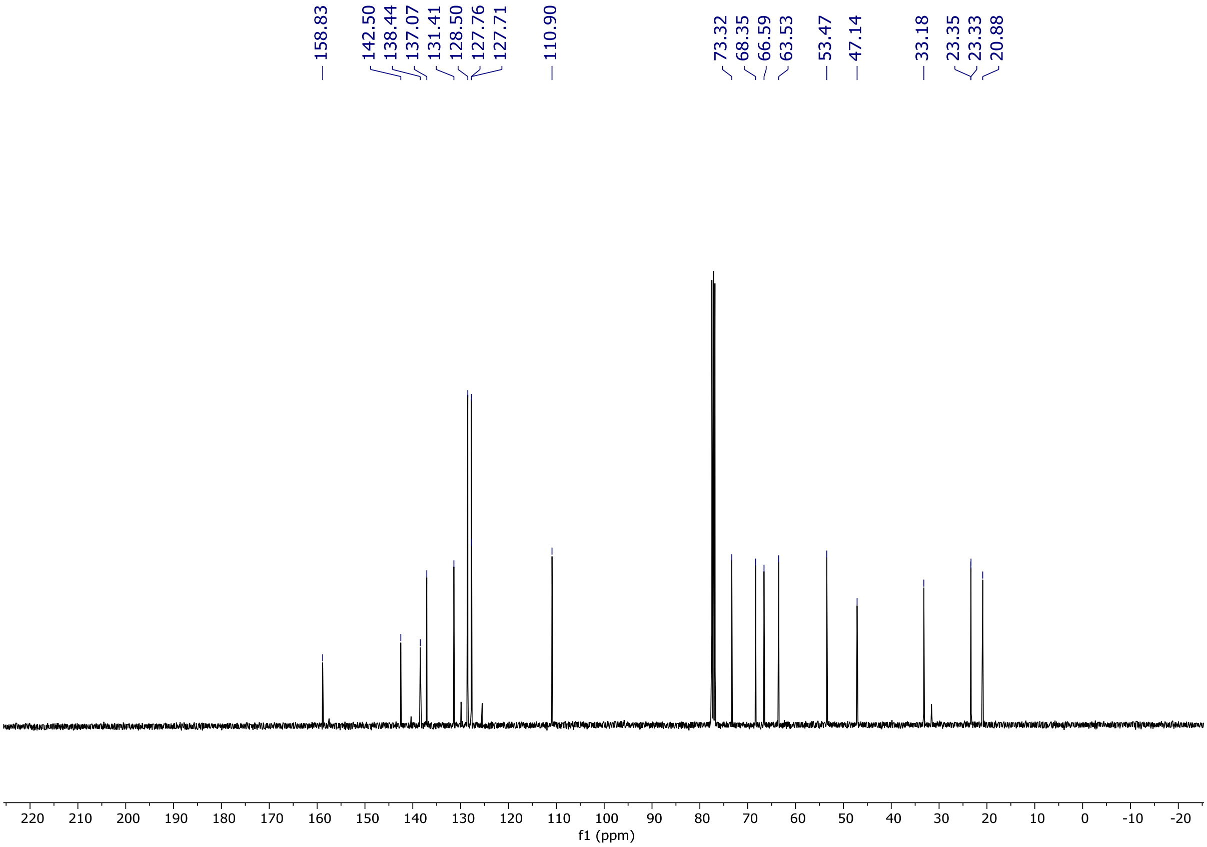

$^{13}C\{^{1}H\}$ NMR (101 MHz, $CDCl_3$) spectrum of compound **59**

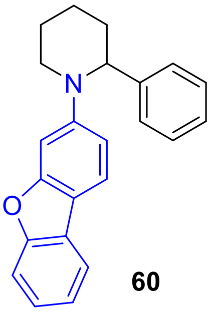

60

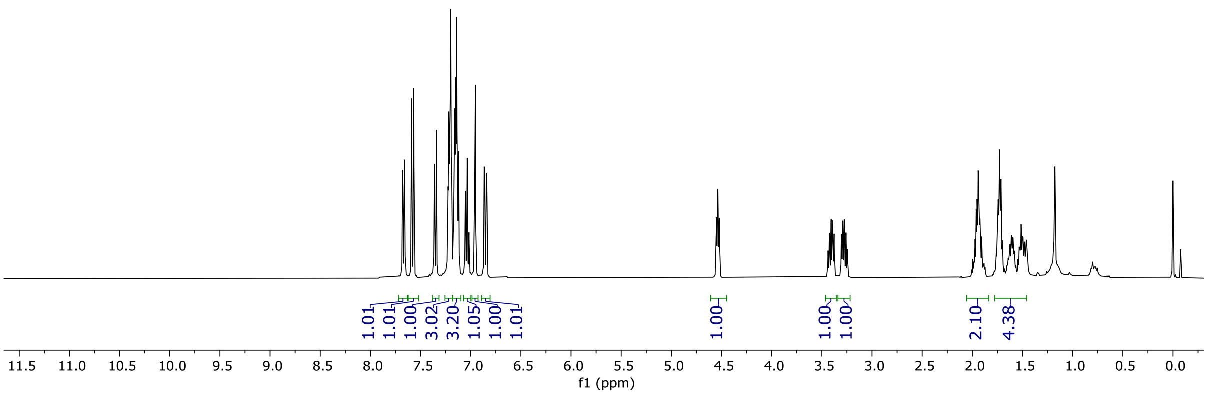

^{1}H NMR (400 MHz, CDCl$_3$) spectrum of compound **60**

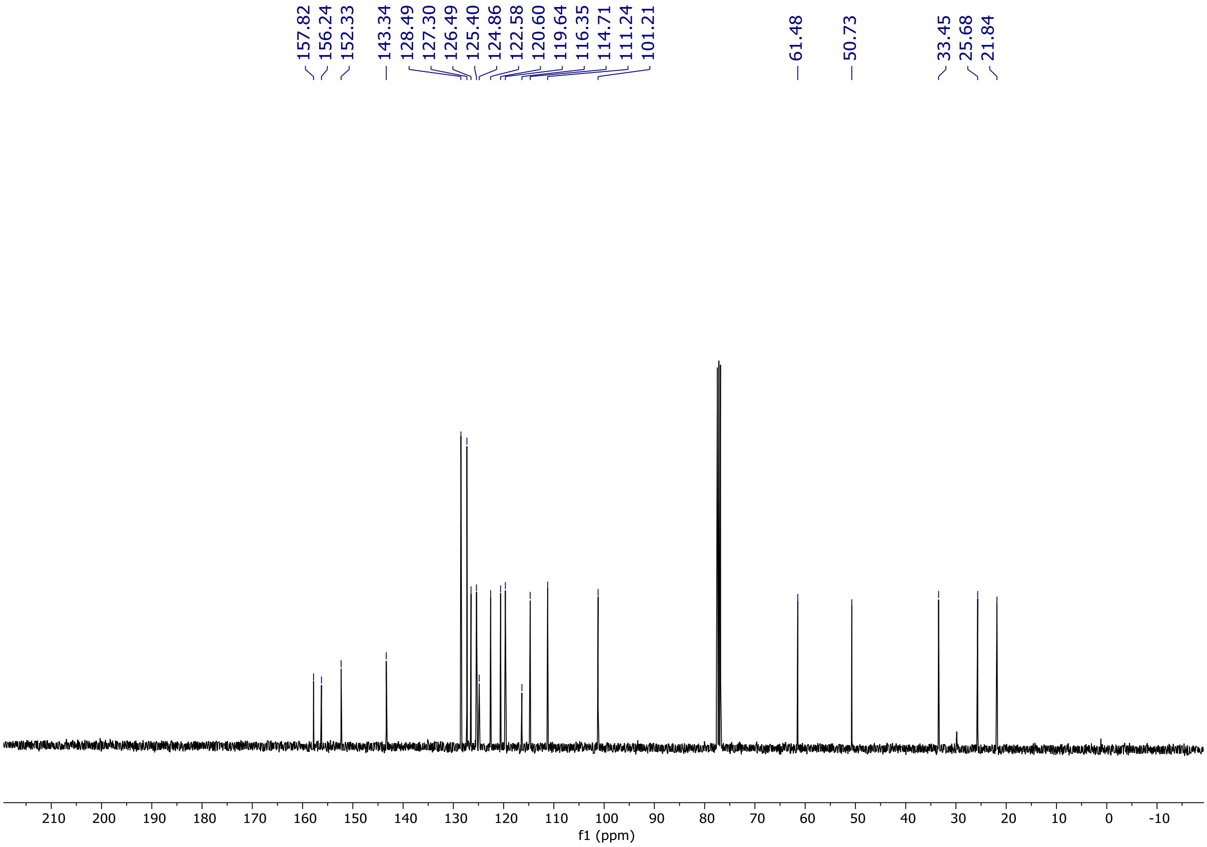

^{13}C{^{1}H} NMR (101 MHz, CDCl$_3$) spectrum of compound **60**

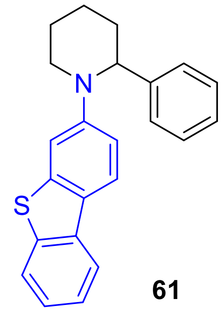

61

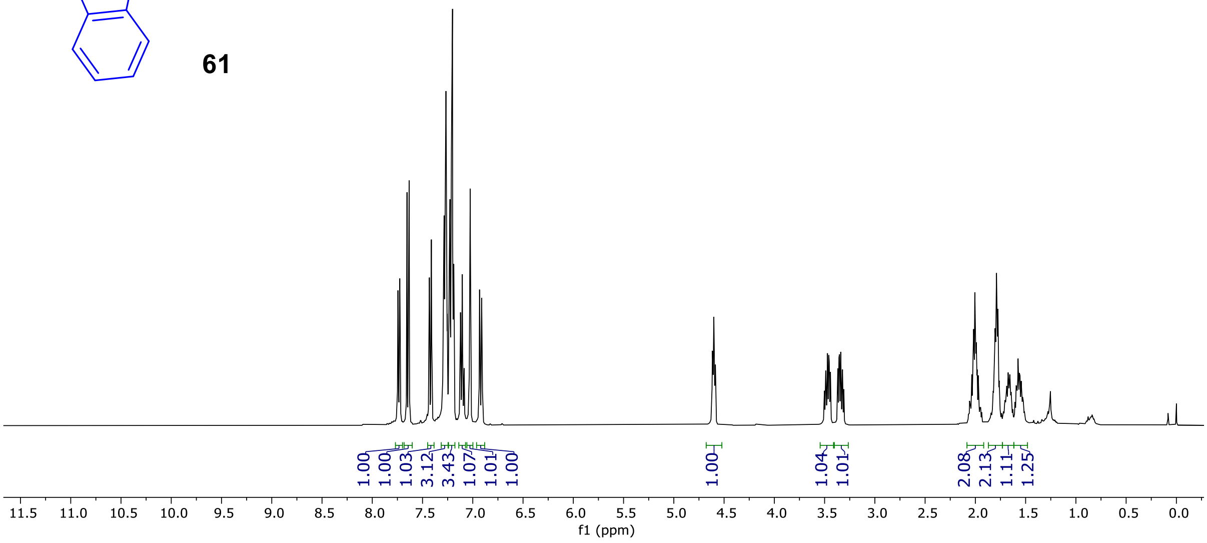

^{1}H NMR (400 MHz, $CDCl_3$) spectrum of compound **61**

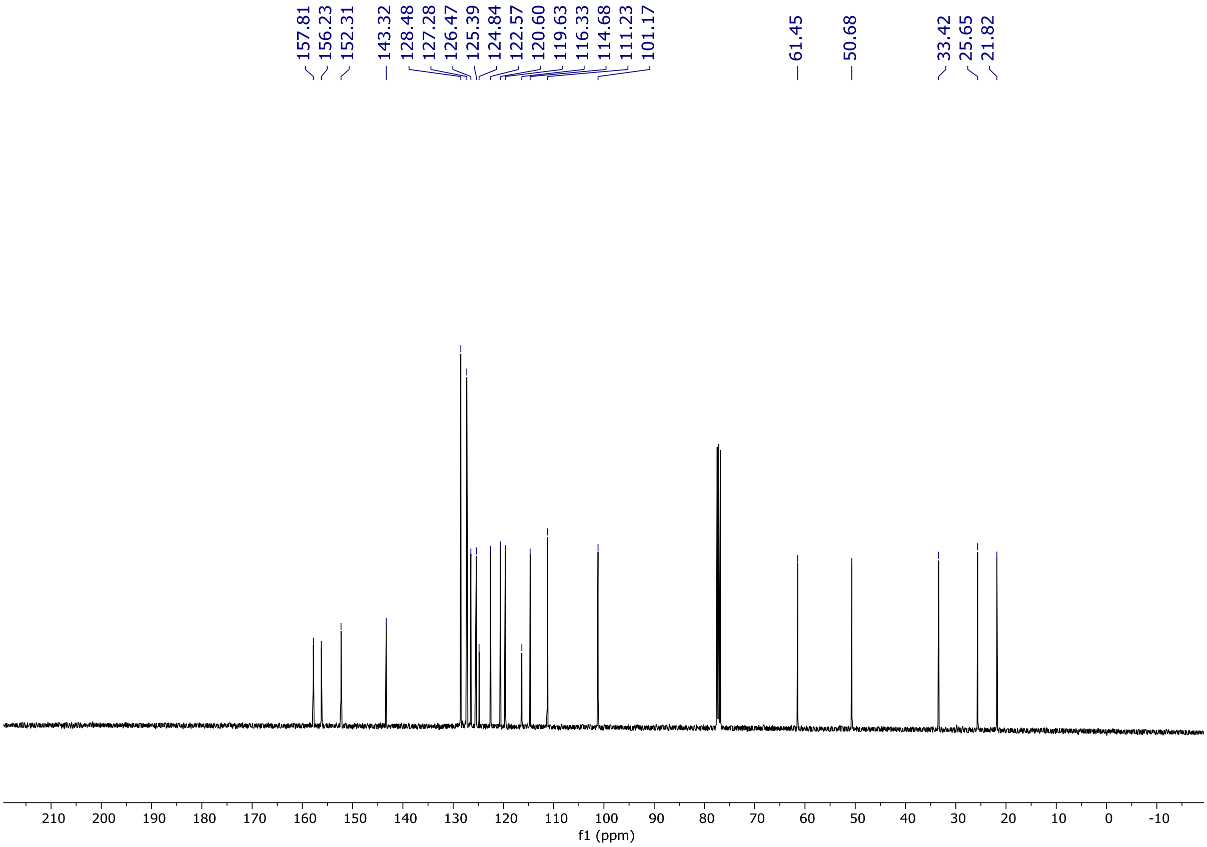

$^{13}C\{^{1}H\}$ NMR (101 MHz, $CDCl_3$) spectrum of compound **61**

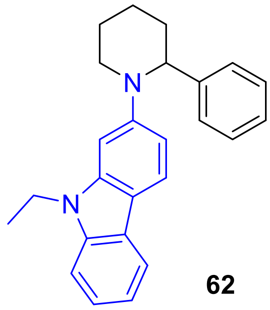

62

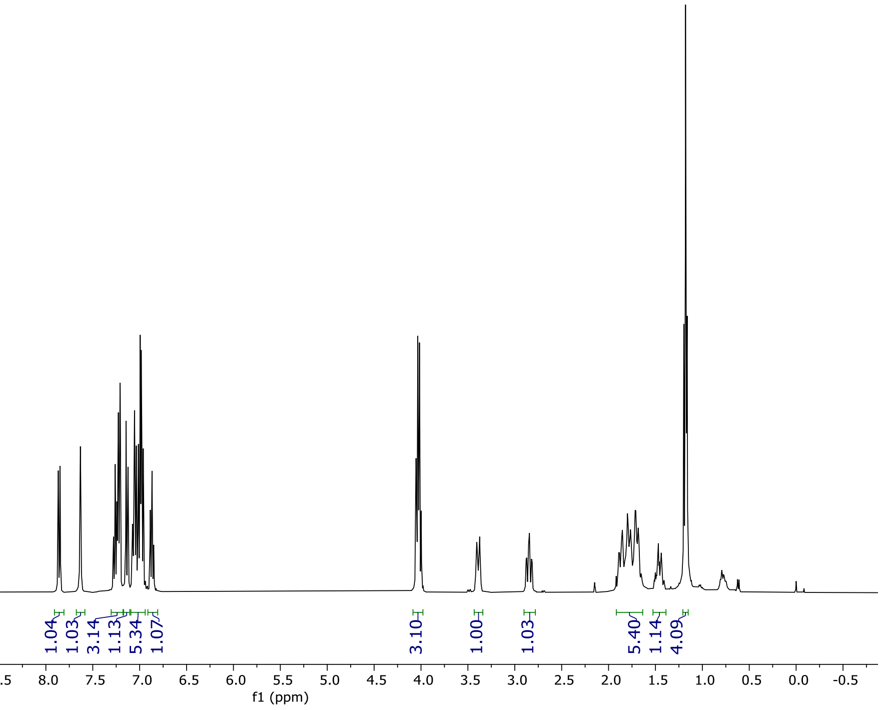

^{1}H NMR (400 MHz, $CDCl_3$) spectrum of compound **62**

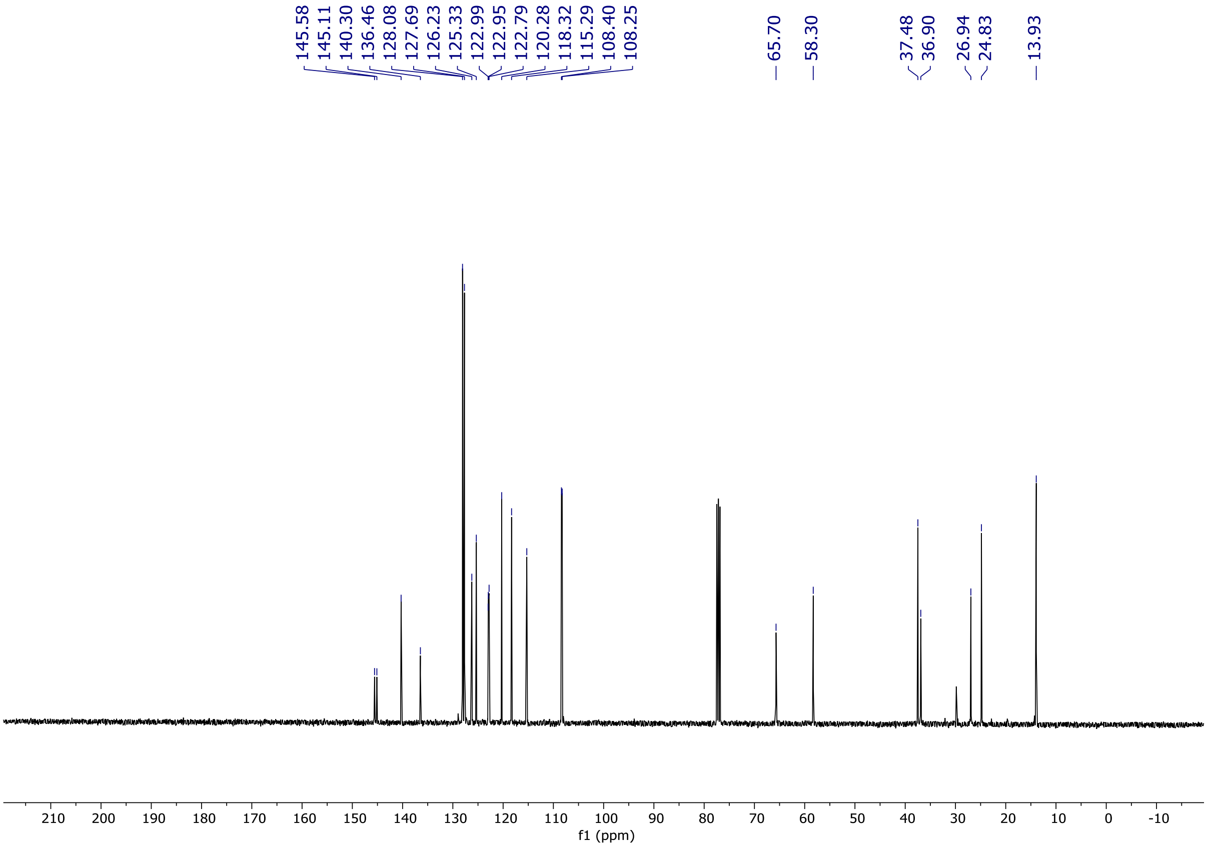

^{13}C{^{1}H} NMR (101 MHz, $CDCl_3$) spectrum of compound **62**

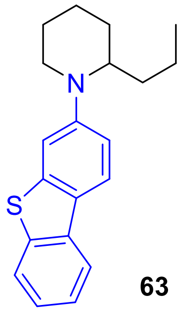

63

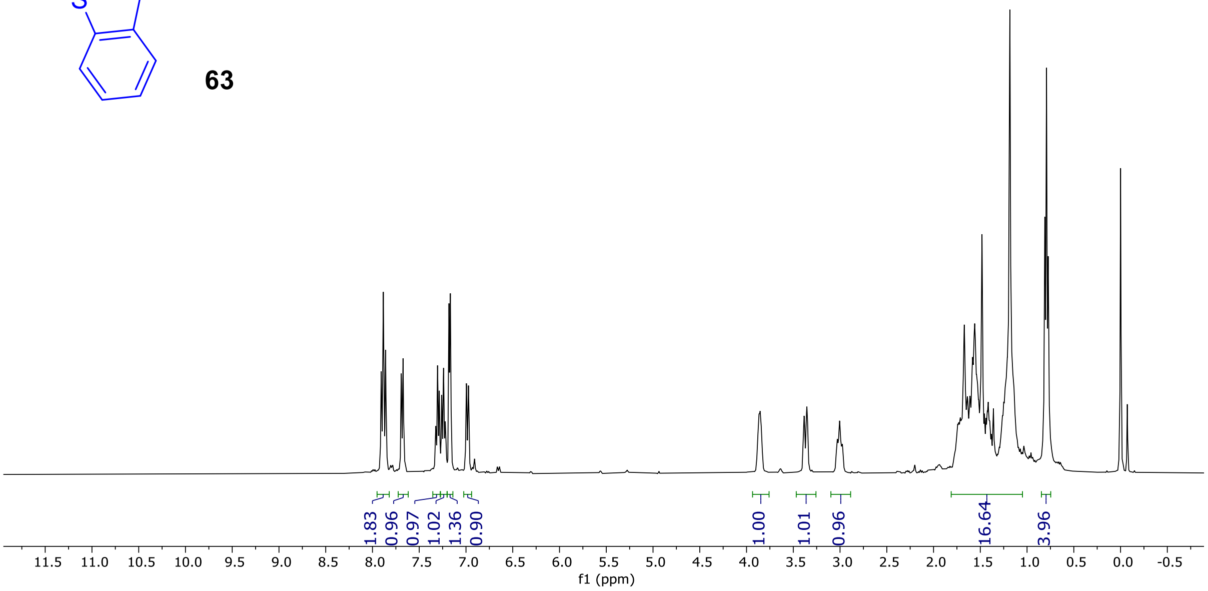

^{1}H NMR (400 MHz, $CDCl_3$) spectrum of compound **63**

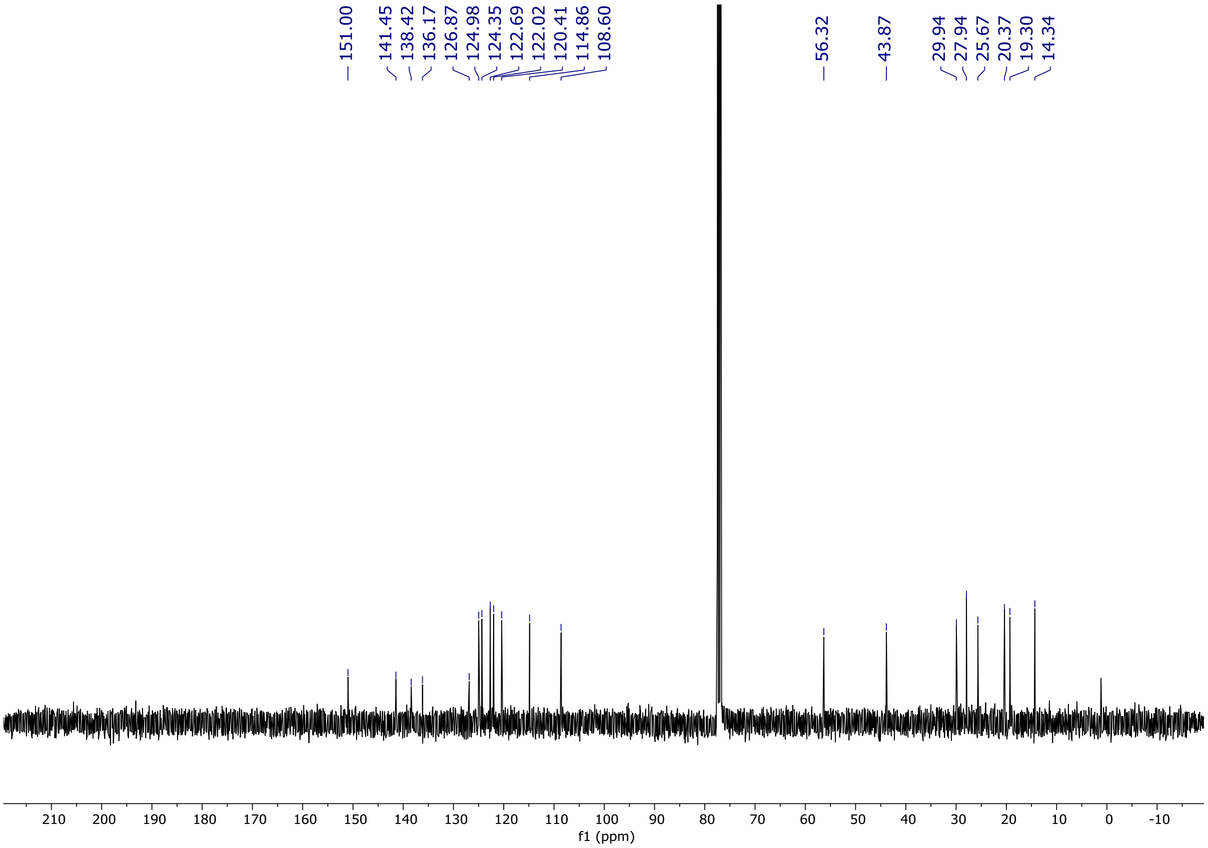

^{13}C{^{1}H} NMR (101 MHz, $CDCl_3$) spectrum of compound **63**

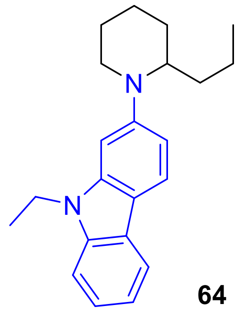

64

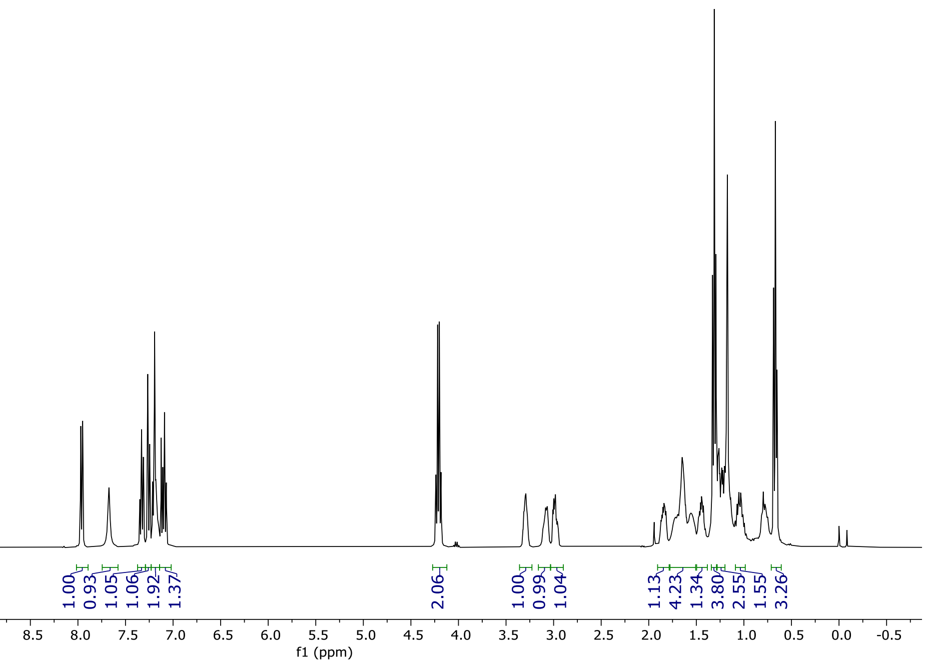

^{1}H NMR (400 MHz, $CDCl_3$) spectrum of compound **64**

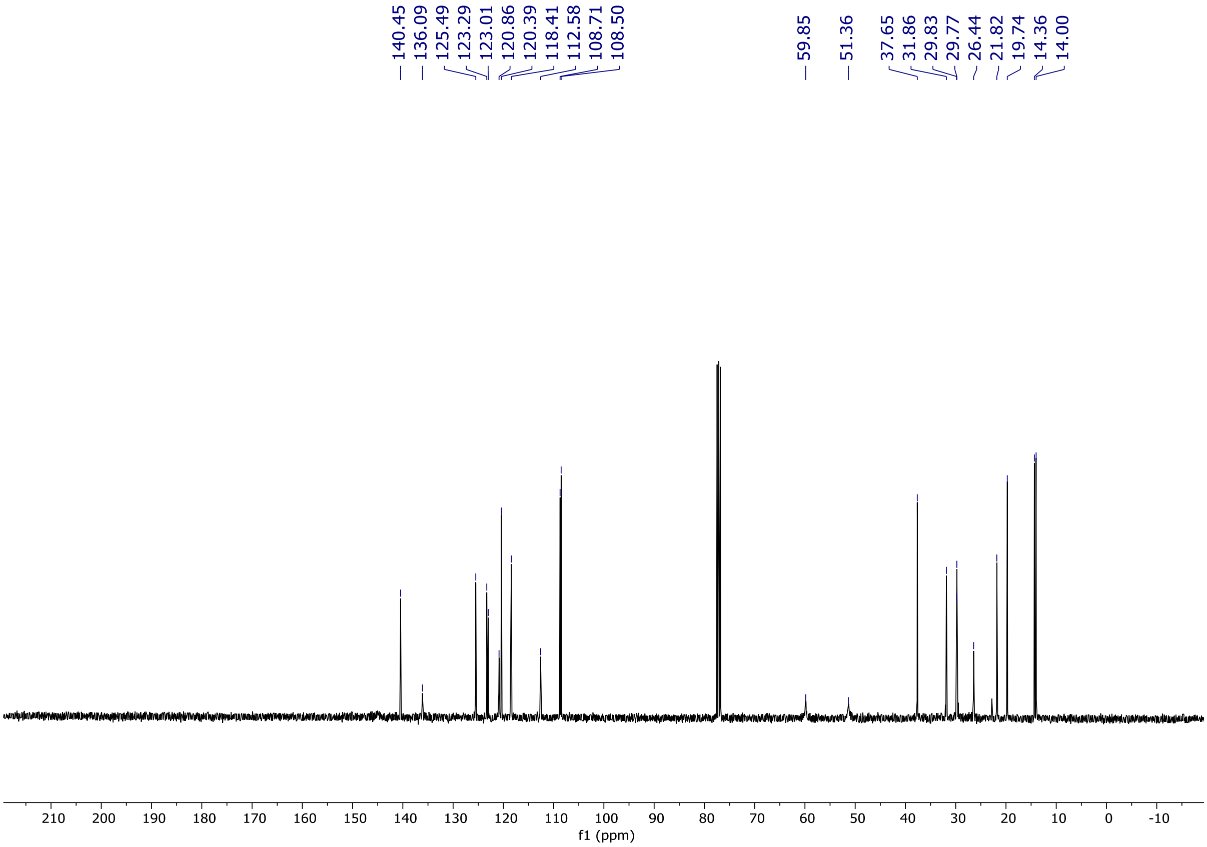

^{13}C{^{1}H} NMR (101 MHz, $CDCl_3$) spectrum of compound **64**

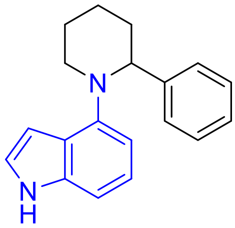

65

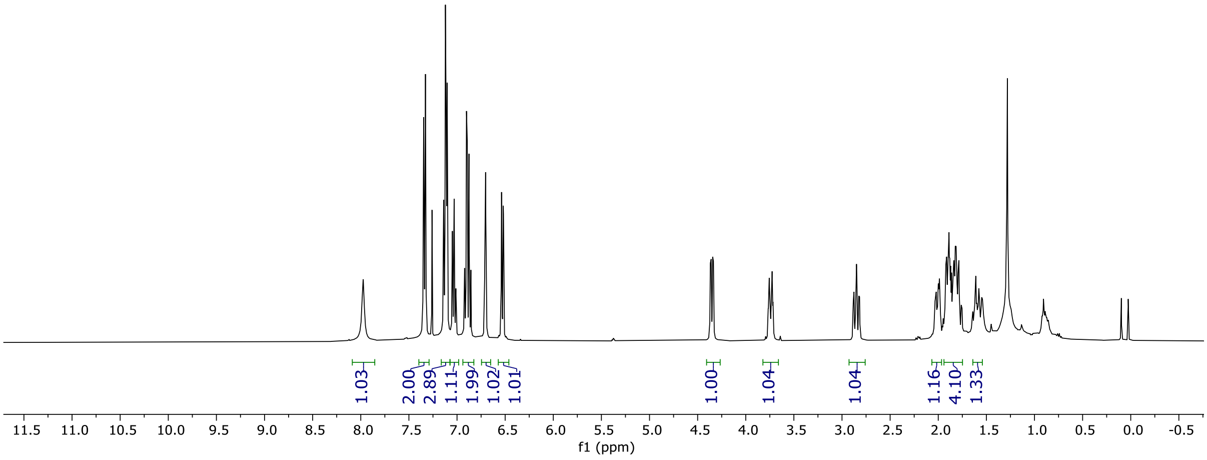

^{1}H NMR (400 MHz, $CDCl_3$) spectrum of compound **65**

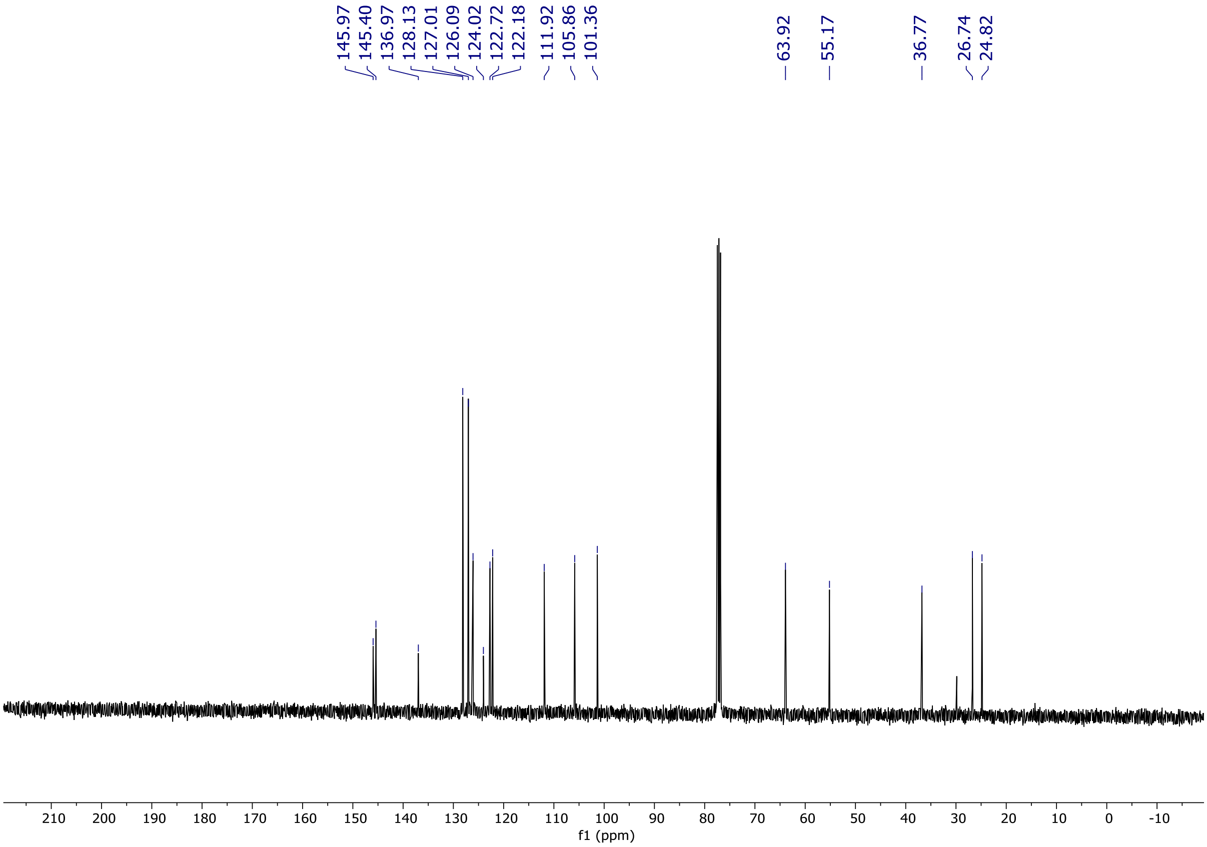

$^{13}C\{^{1}H\}$ NMR (101 MHz, $CDCl_3$) spectrum of compound **65**

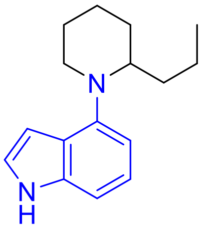

66

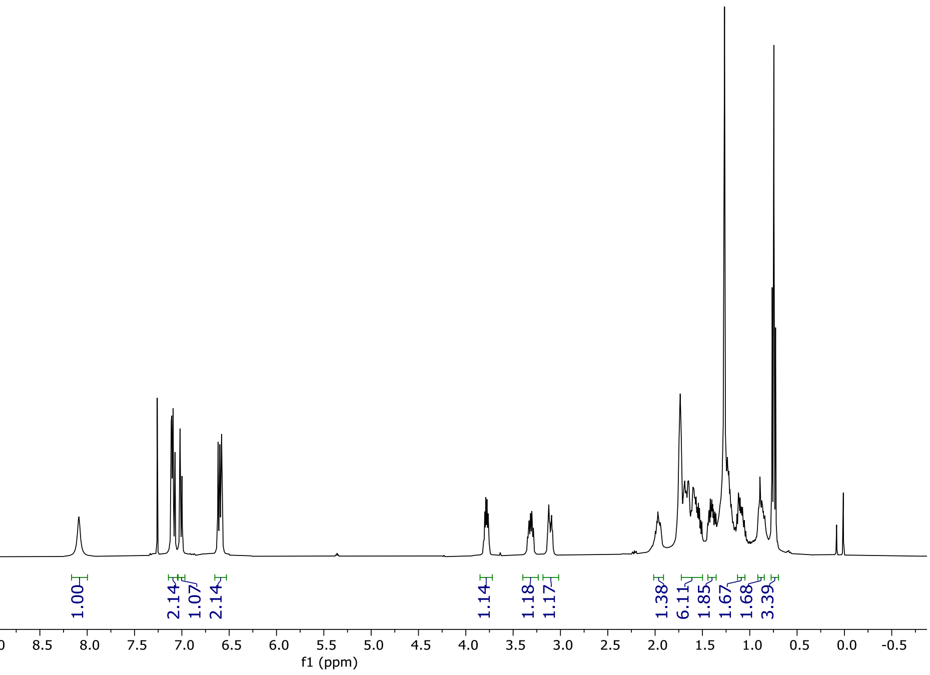

^{1}H NMR (400 MHz, $CDCl_3$) spectrum of compound **66**

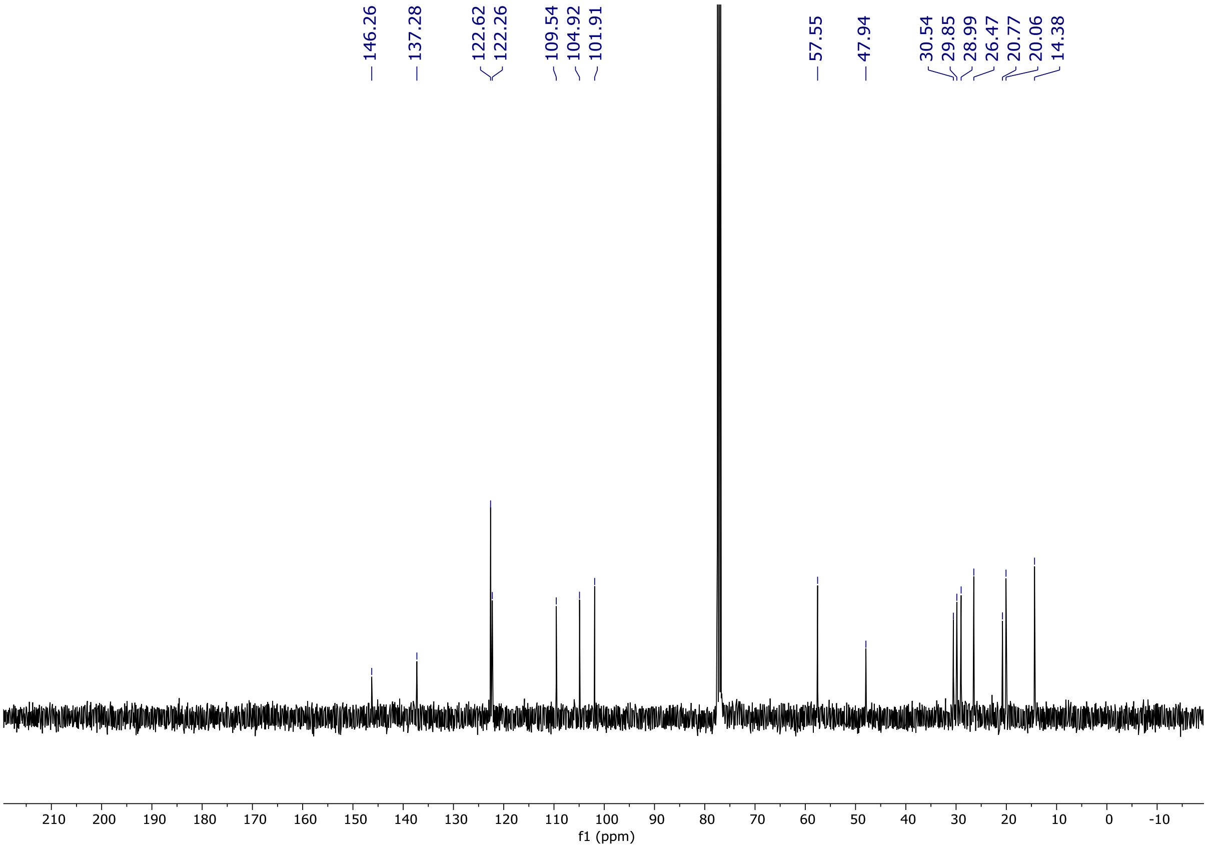

$^{13}C\{^{1}H\}$ NMR (101 MHz, $CDCl_3$) spectrum of compound **66**

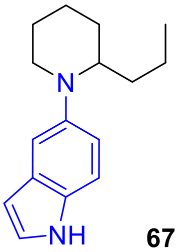

67

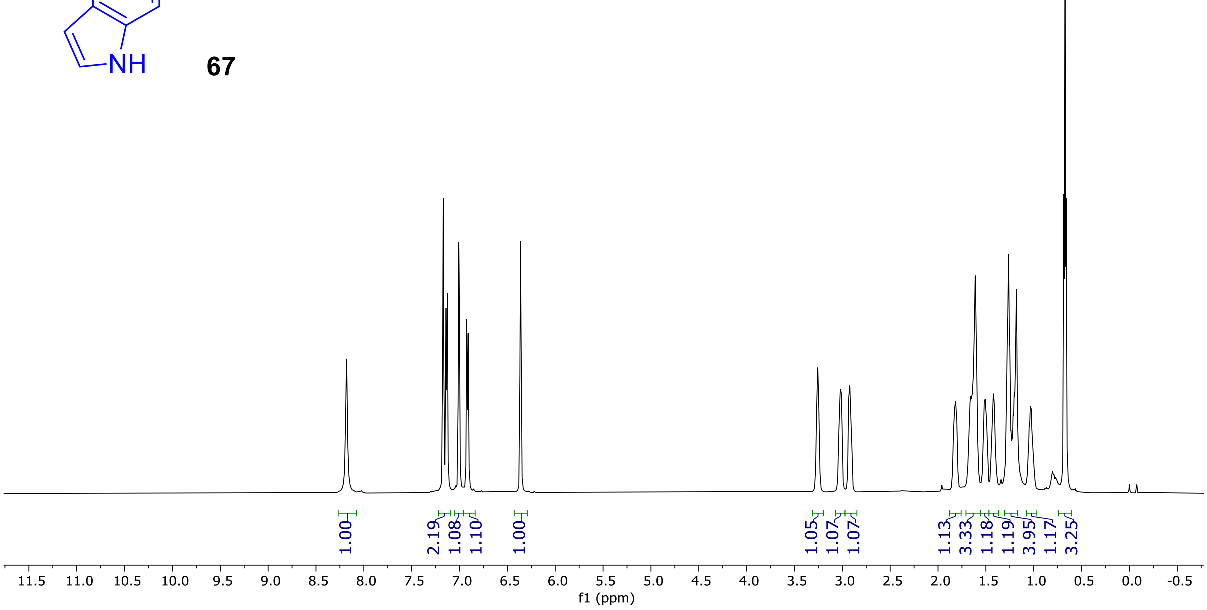

^{1}H NMR (400 MHz, $CDCl_3$) spectrum of compound **67**

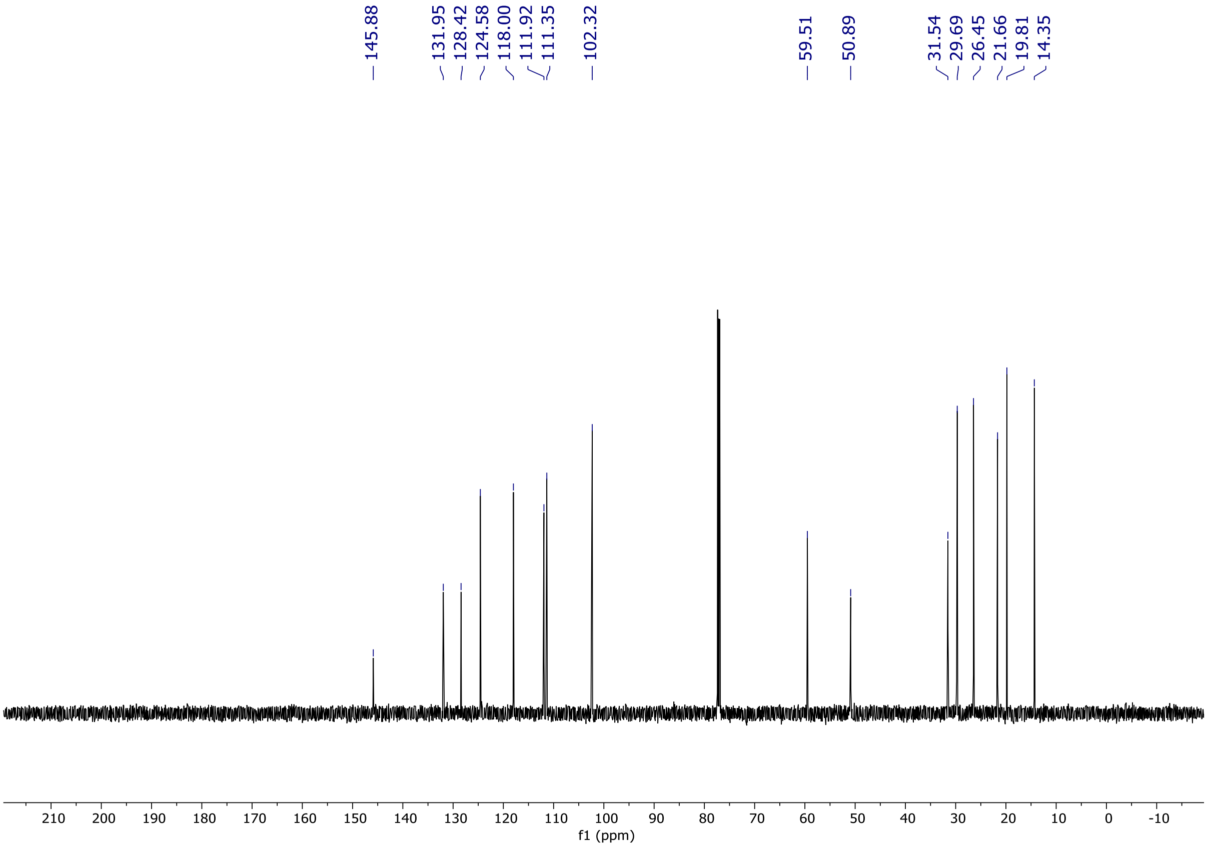

$^{13}C\{^{1}H\}$ NMR (101 MHz, $CDCl_3$) spectrum of compound **67**

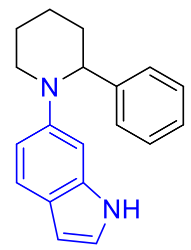

68

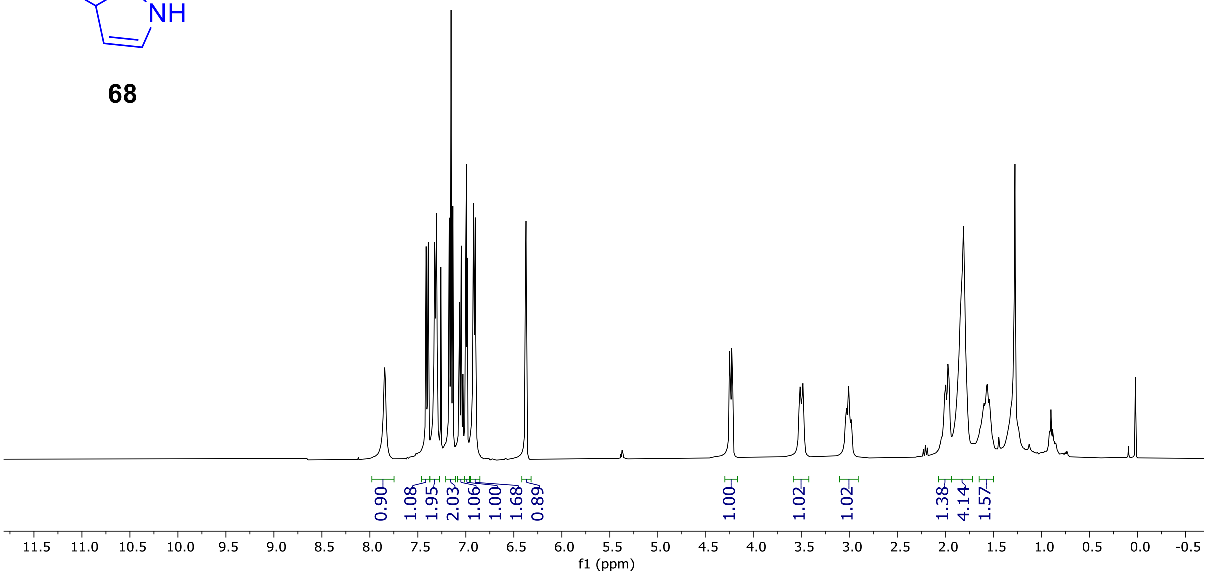

^{1}H NMR (400 MHz, $CDCl_3$) spectrum of compound **68**

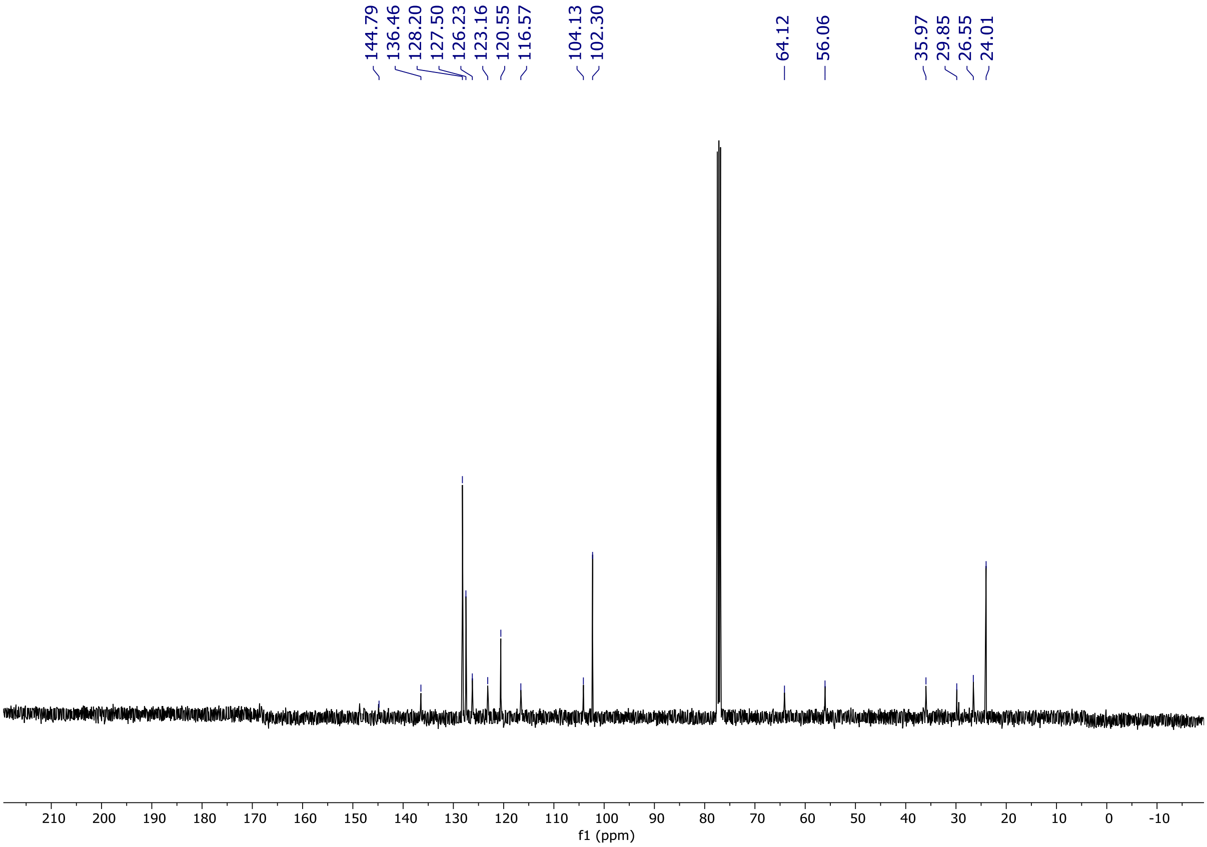

^{13}C{^{1}H} NMR (101 MHz, $CDCl_3$) spectrum of compound **68**

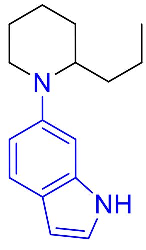

69

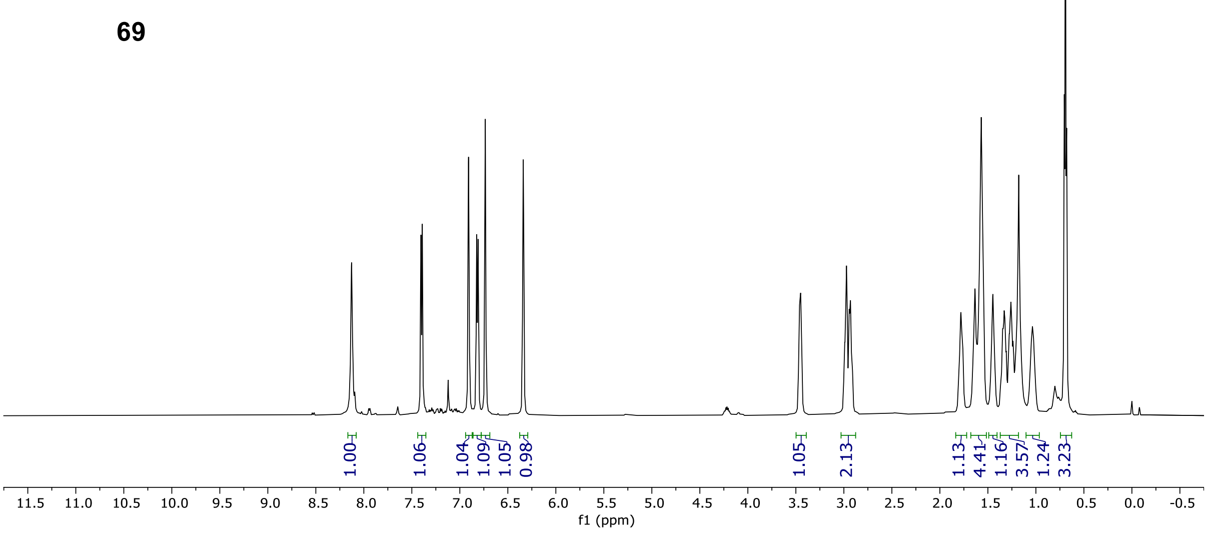

^{1}H NMR (400 MHz, $CDCl_3$) spectrum of compound **69**

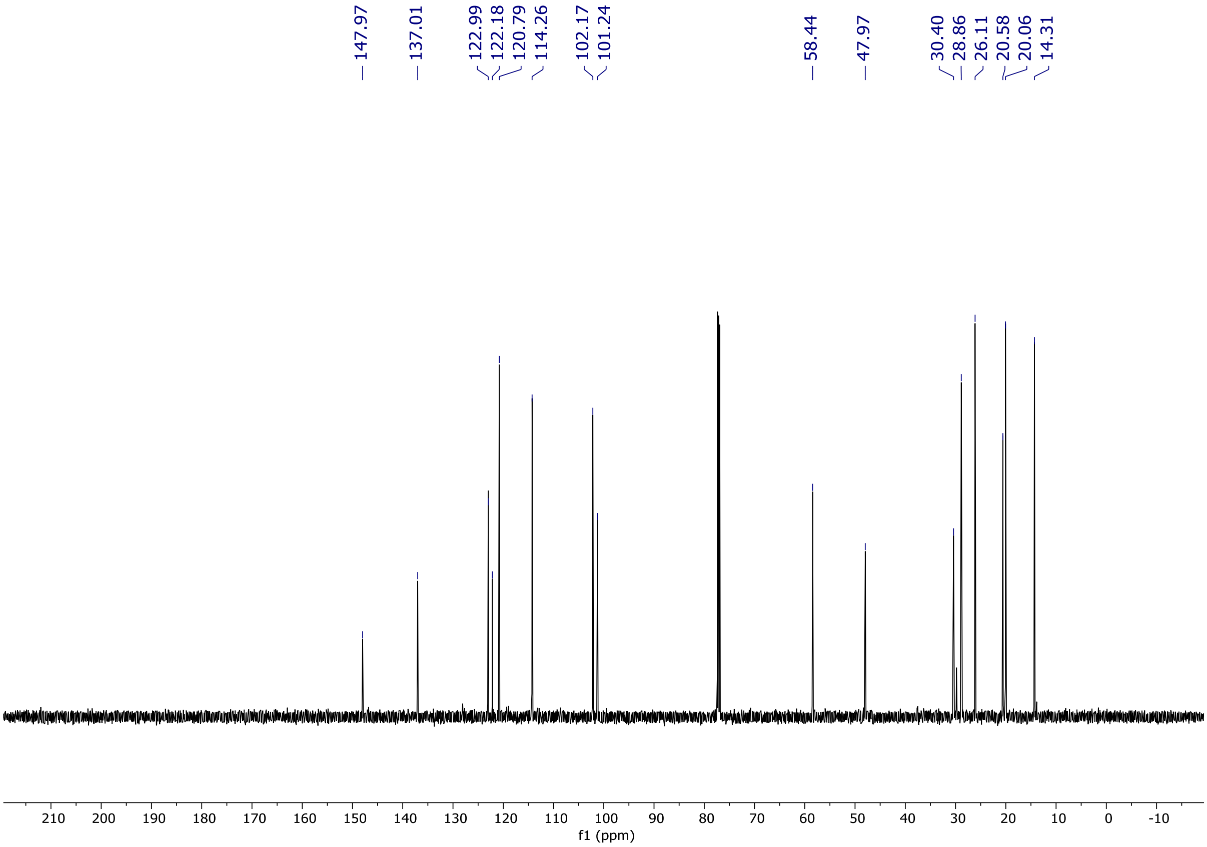

$^{13}C\{^{1}H\}$ NMR (101 MHz, $CDCl_3$) spectrum of compound **69**

8. X-Ray crystallographic data

Table S2. Crystallographic data of compounds 31 and 35

**Compound**	**31**	**35**
Chemical formula	$C_{19}H_{22}FNO_2$	$C_{19}H_{22}FNO_2$
Formula weight	315.37	315.37
Temperature (K)	200(2)	200(2)
Crystal system	Trigonal	Trigonal
Space group	$P3_121$	$P3_121$
a (Å)	8.0722(11)	8.1635(12)
b (Å)	8.0722(11)	8.1635(12)
c (Å)	21.809(4)	21.625(4)
α (°)	90	90
β (°)	90	90
γ (°)	120	120
V (Å^3)	1230.7(4)	1248.0(4)
Z	3	3
ρ (calc.) mg m^{-3}	1.277	1.259
μ (Mo Kα) mm^{-1}	0.090	0.089
R1 [F>4σ(F)]	0.0433	0.0469
wR2 (all data)	0.1113	0.1326
CCDC numbers	2259937	2259942

On the structural disorder of **31** and **35**

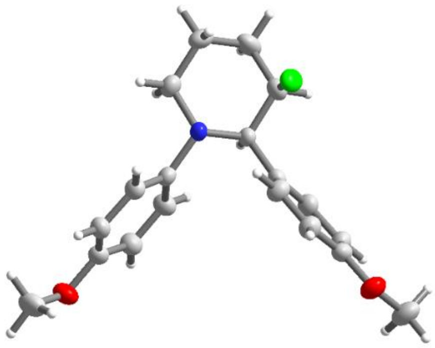

31

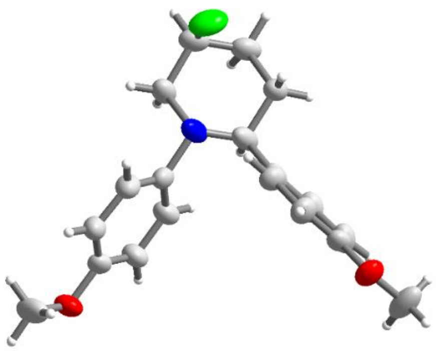

35

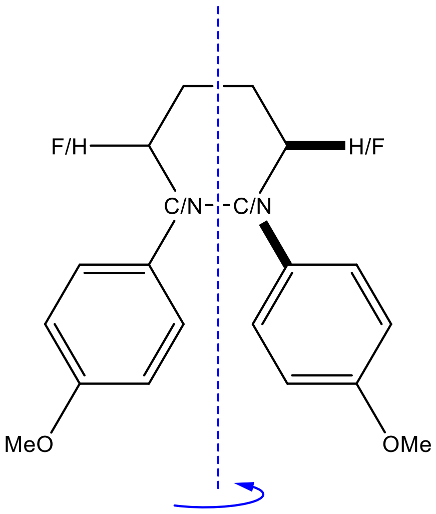

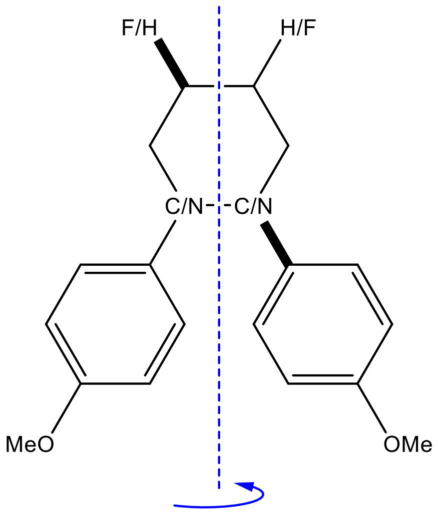

Figure S3. Crystal structures of **31** and **35** (top; C, grey; H, white, O, red; N, blue; F, green). Thermal ellipsoids are shown at the 50% probability level.

Both structures were refined in space group symmetry $P3_121$ (absolute structures could not be obtained reliably). They show a similar disorder about the crystallographically 2-fold rotation axis (marked by dashed blue line). This involves the superposition of C and N-atoms binding the anisidyl groups and of the F-atom with the symmetrically related H-atom.

9. References

1. Sheldrick, G. M., A short history of SHELX, *Acta Crystallogr*. **2008**, A64, 112.
2. Grozavu, A.; Hepburn, H.B.; Smith, P. J. *et al*. The reductive C3 functionalization of pyridinium and quinolinium salts through iridium-catalysed interrupted transfer hydrogenation. *Nat. Chem.* **2019**, 11, 242–247.
3. Gross, K. C.; Seybold, P. G.; Hadad, C. M., Comparison of different atomic charge schemes for predicting pKa variations in substituted anilines and phenols. *Int. J. Quantum Chem.* **2002**, 90 (1), 445-458.
4. Brotzel, F.; Chu, Y. C.; Mayr, H., Nucleophilicities of Primary and Secondary Amines in Water. *J. Org. Chem.* **2007**, 72 (10), 3679-3688.
5. Watile, R. A.; Bunrit, A.; Margalef, J.; Akkarasamiyo, S.; Ayub, R.; Lagerspets, E.; Biswas, S.; Repo, T.; Samec, J. S. M., Intramolecular substitutions of secondary and tertiary alcohols with chirality transfer by an iron(III) catalyst. *Nat Commun*. **2019**, 10 (1), 3826.
6. Nairoukh, Z.; Wollenburg, M.; Schlepphorst, C.; Bergander, K.; Glorius, F., The formation of all-*cis*-(multi)fluorinated piperidines by a dearomatization–hydrogenation process. *Nat. Chem.* **2019**, 11 (3), 264-270.
